# A Vibrio cholerae BolA-Like Protein Is Required for Proper Cell Shape and Cell Envelope Integrity

**DOI:** 10.1128/mBio.00790-19

**Published:** 2019-07-09

**Authors:** Aurore Fleurie, Abdelrahim Zoued, Laura Alvarez, Kelly M. Hines, Felipe Cava, Libin Xu, Brigid M. Davis, Matthew K. Waldor

**Affiliations:** a Department of Microbiology, Harvard Medical School, Boston, Massachusetts, USA; b Division of Infectious Diseases, Brigham and Women’s Hospital, Boston, Massachusetts, USA; c Laboratory for Molecular Infection Medicine Sweden, Department of Molecular Biology, Umeå Centre for Microbial Research, Umeå University, Umeå, Sweden; d Department of Medicinal Chemistry, University of Washington, Seattle, Washington, USA; e Howard Hughes Medical Institute, Boston, Massachusetts, USA; Princeton University

**Keywords:** BolA, IbaG, *Vibrio cholerae*, cell envelope, cell shape, iron-sulfur cluster

## Abstract

BolA family proteins are conserved in Gram-negative bacteria and many eukaryotes. While diverse cellular phenotypes have been linked to this protein family, the molecular pathways through which these proteins mediate their effects are not well described. Here, we investigated the roles of BolA family proteins in Vibrio cholerae, the cholera pathogen. Like Escherichia coli, V. cholerae encodes two BolA proteins, BolA and IbaG. However, in marked contrast to E. coli, where *bolA* is linked to cell shape and *ibaG* is not, in V. cholerae, *bolA* mutants lack morphological defects, whereas *ibaG* proved critical for the generation and/or maintenance of the pathogen’s morphology. Notably, the bizarre-shaped, multipolar, elongated, and wide cells that predominated in exponential-phase Δ*ibaG* V. cholerae cultures were not observed in stationary-phase cultures. The V. cholerae Δ*ibaG* mutant exhibited increased sensitivity to cell envelope stressors, including cell wall-acting antibiotics and bile, and was defective in intestinal colonization. Δ*ibaG* V. cholerae had reduced peptidoglycan and lipid II and altered outer membrane lipids, likely contributing to the mutant’s morphological defects and sensitivity to envelope stressors. Transposon insertion sequencing analysis of *ibaG*’s genetic interactions suggested that *ibaG* is involved in several processes involved in the generation and homeostasis of the cell envelope. Furthermore, copurification studies revealed that IbaG interacts with proteins containing iron-sulfur clusters or involved in their assembly. Collectively, our findings suggest that V. cholerae IbaG controls cell morphology and cell envelope integrity through its role in biogenesis or trafficking of iron-sulfur cluster proteins.

## Abstract (precis)

BolA-like proteins are conserved across prokaryotes and eukaryotes. These proteins have been linked to a variety of phenotypes, but the pathways and mechanisms through which they act have not been extensively characterized. Here, we unraveled the role of the BolA-like protein IbaG in the cholera pathogen Vibrio cholerae. The absence of IbaG was associated with dramatic changes in cell morphology, sensitivity to envelope stressors, and intestinal colonization defects. IbaG was found to be required for biogenesis of several components of the V. cholerae cell envelope and to interact with numerous iron-sulfur cluster-containing proteins and factors involved in their assembly. Thus, our findings suggest that IbaG governs V. cholerae cell shape and cell envelope homeostasis through its effects on iron-sulfur proteins and associated pathways. The diversity of processes involving iron-sulfur-containing proteins is likely a factor underlying the range of phenotypes associated with BolA family proteins.

## INTRODUCTION

The BolA protein family is widely conserved across Gram-negative bacteria and eukaryotes (1). These proteins have been linked to a range of cellular phenotypes, including cell morphology, membrane permeability, motility, and biofilm formation (2). BolA-like proteins have a class II KH fold related to that of the OsmC hyperperoxide reductase (3) that includes a helix-turn-helix (HTH) domain (1). The HTH domains of certain BolA proteins have been shown to bind DNA and modulate transcription (4). In several species, BolA family members have been linked to stress response pathways, and the absence or overexpression of BolA proteins can modulate bacterial viability in response to a variety of environmental challenges. Also, there is an emerging understanding of a role for BolA proteins in iron homeostasis and iron-sulfur cluster assembly and trafficking (5). The varied genomic context for genes encoding BolA family members, the range of phenotypes associated with BolA proteins, and the fact that some organisms carry genes that encode multiple BolA family members suggests that these proteins likely contribute to a variety of processes, both across species and within a single species. However, mechanisms underlying these proteins’ diverse effects on cell physiology have largely not been determined.

In Escherichia coli, *bolA* expression is induced in response to several stressors (6). Overexpression of *bolA* in E. coli induces formation of spherical cells (7), potentially due to associated upregulation of *dacA* and *dacC*, which encode the penicillin binding proteins (PBPs) PBP5 and PBP6, as well as to downregulation of *mreB* (8–10). Overexpression of *bolA* is also thought to decrease the permeability of the bacterial outer membrane, while the absence of *bolA* alters the accessibility of outer membrane proteins (11). Transcriptomic and ChIP analyses have shown that BolA overexpression directly modulates transcription (4). Finally, a role for BolA in biofilm formation has been demonstrated (4, 12, 13).

E. coli, like many other organisms, contains genes that encode more than one BolA family protein. In addition to the 105-amino-acid protein BolA, E. coli carries a gene that encodes IbaG (formerly YrbA), an 84-amino-acid protein that also contains the characteristic class II KH fold of BolA proteins (14). Unlike BolA, neither overexpression nor the absence of IbaG alters E. coli cell shape; however, overexpression of *ibaG* is deleterious to bacterial growth, while its deletion enhanced bacterial growth (14). *ibaG* expression is induced in response to acid, accounting for its name, *i*nfluenced *b*y *a*cid *g*ene, and the absence of *ibaG* also increases E. coli sensitivity to acid stress. Although IbaG, like BolA, is presumed to act as a transcription factor, it does not appear to recognize sequences bound by BolA (14). Thus, in E. coli, IbaG’s role is distinct from that of BolA.

BolA-like proteins have roles in genesis of iron-sulfur proteins through their partnerships with monothiol glutaredoxins (Grxs). Bioinformatics analysis of co-occurrence provided the first clue linking Grx proteins and BolA-like proteins; the simultaneous presence or absence in many genomes of genes encoding both these proteins suggested a functional interaction between them (1). Subsequently, it has been shown in E. coli and several eukaryotes that monothiol Grxs and BolA proteins form heterocomplexes implicated in iron-sulfur cluster assembly and trafficking (5). In particular, E. coli’s single monothiol Grx (Grx4) forms [2Fe-2S]-bridged heterodimers with BolA and IbaG (15, 16). Mutations in either *grxD* (encoding Grx4) or *ibaG* have been reported to increase the growth defects of strains with mutations in genes in the *isc* operon. This operon encodes several of the elements critical for the housekeeping iron-sulfur cluster assembly pathway, suggesting that Grx4 and IbaG may mediate an alternate process of iron-sulfur cluster assembly (17).

**FIG 1:**
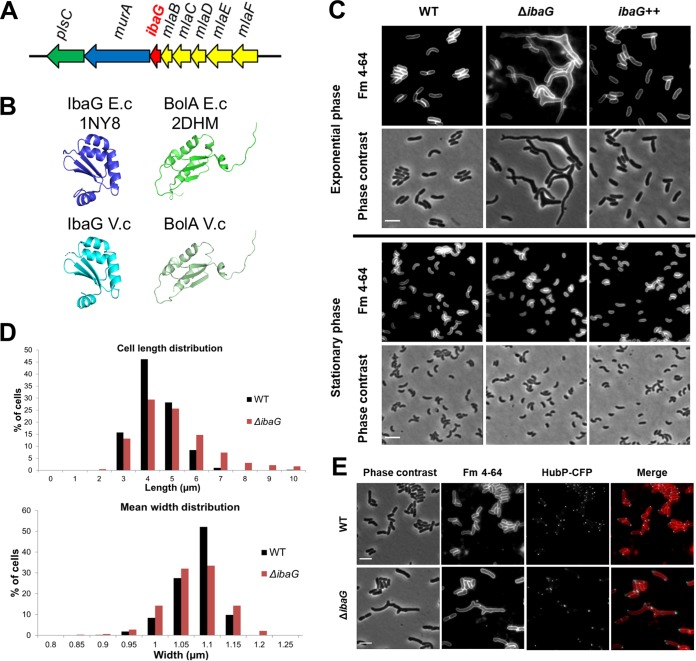
IbaG is required for Vibrio cholerae cell shape. (A) Schematic of the genomic neighborhood of *ibaG* (red), which includes *mlaBCDEF* (yellow), implicated in maintenance of outer membrane lipid asymmetry, *murA* (blue), involved in synthesis of peptidoglycan precursors, and *plsC* (green), implicated in phospholipid synthesis. (B) Comparison of structures of BolA and IbaG in E. coli and V. cholerae. Structure of IbaG (PDB accession code 1NY8; left) and BolA from E. coli (E.c) (PDB accession code 2DHM; right) (top panel) and the predicted models obtained with PHYRE2 for IbaG and BolA from V. cholerae (V.c) (bottom panel). (C) Phase contrast and fluorescence imaging of FM4-64-stained WT, Δ*ibaG*, and *ibaG* overexpressing (*ibaG*++) V. cholerae grown to exponential and stationary phase in M9 medium. Bars, 2 μm. (D) Cell length and mean width distribution of WT and Δ*ibaG* strains grown in M9 medium. At least 1,000 cells were measured for each condition using MicrobeTracker. Statistical significance was determined using a nonparametric Mann-Whitney U test. *P* value ≤0.001. (E) Fluorescence imaging of WT and Δ*ibaG* strains grown in M9 medium and expressing a chromosome-encoded HubP-CFP. Cells were also stained with FM4-64. Bars, 2 μm.

Like E. coli, the Gram-negative pathogen Vibrio cholerae carries genes that encode two members of the BolA protein family, BolA and IbaG. *ibaG* has a similar genomic context in both organisms; it is found downstream of *mlaBCDEF*, which encode components of an ABC transport system required for maintenance of outer membrane lipid asymmetry, and upstream of *murA*, whose product catalyzes the first step in peptidoglycan assembly (Fig. 1A). In contrast, genomic placement of *bolA* is not conserved between V. cholerae and E. coli. To date, no role has been reported for either BolA family member in V. cholerae.

Here, we explored the role of BolA family proteins in V. cholerae, the cholera pathogen. We found that in marked contrast to E. coli, V. cholerae *ibaG* is critical for the generation and/or maintenance of the pathogen’s morphology. V. cholerae Δ*ibaG* bacteria exhibited increased sensitivity to cell envelope stressors and were defective in intestinal colonization. These defects are likely attributable to the aberrant composition of the mutant’s cell envelope, including reduced peptidoglycan and altered outer membrane lipids. The results of genetic and protein interaction analyses suggest that IbaG may control V. cholerae cell morphology and envelope integrity through its role in biogenesis or trafficking of iron-sulfur cluster proteins.

## RESULTS

### IbaG is required for V. cholerae cell morphology and growth.

The predicted amino acid sequences of BolA and IbaG in V. cholerae and E. coli are highly similar (see Fig. S1A and B in the supplemental material). Furthermore, the predicted structures of homologous proteins are nearly identical for the two species (Fig. 1B). In contrast, the two V. cholerae BolA family proteins share only 24% amino acid similarity despite the conservation of their secondary structures (Fig. S1C and D). We constructed derivatives of V. cholerae N16961 in which either *ibaG* or *bolA* is deleted or overexpressed. Phase contrast and fluorescence microscopy of these cells revealed no effect of *bolA* on cell shape (Fig. S2A). In marked contrast, exponential-phase Δ*ibaG* cells had grossly distorted cell shapes (Fig. 1C and D), whereas overexpression of *ibaG* did not influence V. cholerae morphology (Fig. 1C and D). The morphological defects of the *ibaG* deletion mutant were observed in both LB and M9 media but were more pronounced in the latter (Fig. S2B). The Δ*ibaG* cells were generally longer and wider than the wild type (WT); moreover, many of the mutant cells exhibited branching and the presence of extra cell poles (Fig. 1C to E). HubP, a key regulator of V. cholerae pole development (18), was often detected at all poles (Fig. 1E), suggesting that the polar cell domain is intact at the supernumerary poles in branched Δ*ibaG* cells.

10.1128/mBio.00790-19.1 FIG S1 Comparison of secondary structures and amino acid sequences of BolA and IbaG in E. coli and V. cholerae. (A to C) ClustalW alignment of predicted amino acid sequences of BolA and IbaG from E. coli (E.c) and V. cholerae (V.c). The KEGG database was used to obtain the protein sequences, which were aligned using ClustalW NPSA. (D) Clustal Omega alignment of BolA and IbaG from V. cholerae. Predictions of secondary structures were generated using Ali2D and PSIpred. Alpha helices (H) and beta-sheets (E) are colored in red and blue, respectively. The helix-turn-helix motif (HTH) is annotated. Download FIG S1, TIF file, 1.5 MB. Copyright © 2019 Fleurie et al. 2019 Fleurie et al. This content is distributed under the terms of the Creative Commons Attribution 4.0 International license.

10.1128/mBio.00790-19.2 FIG S2 Morphology and growth of *bolA* and *ibaG* mutant cells. (A) Phase contrast and fluorescence imaging of FM4-64-stained Δ*bolA* and *bolA* overexpressing (*bolA*++) cells grown to exponential phase and stationary phase in M9 medium. Scale bars, 2 μm. (B) Phase contrast and fluorescence imaging of FM4-64-stained Δ*ibaG* cells grown to exponential phase in M9 and LB media. Scale bars, 2 μm. (C) Cell length and mean width distribution of WT and Δ*ibaG* strains grown to stationary phase in M9 medium. At least 1,000 cells were measured for each condition using MicrobeTracker; the differences in the distributions of lengths and widths (determined with a Mann-Whitney test) were not significant (*P* values of >0.15 for both). Download FIG S2, TIF file, 0.7 MB. Copyright © 2019 Fleurie et al. 2019 Fleurie et al. This content is distributed under the terms of the Creative Commons Attribution 4.0 International license.

Notably, the mutant’s morphological defects were observed only during exponential-phase growth; in stationary phase, Δ*ibaG* cells exhibited normal shape and size (Fig. 1C and Fig. S2C). These differences cannot be explained by changes in *ibaG* expression, which were very similar during exponential and stationary phase (Fig. S3A). The Δ*ibaG* morphological defects were eliminated by expression of *ibaG* in *trans*, indicating that shape changes are specifically linked to the absence of IbaG and not due to polar effects on other genes in the putative *ibaG* operon (Fig. S3B and C and Fig. S4B). Thus, in marked contrast to E. coli, *ibaG* has a pronounced influence on V. cholerae morphology*;* furthermore, *bolA* does not appear to modulate V. cholerae cell shape, whereas its overexpression in E. coli results in shape defects (7, 8). On the basis of these observations, additional studies were focused on deciphering the role(s) of *ibaG* in V. cholerae.

10.1128/mBio.00790-19.3 FIG S3 Complementation and *ibaG* expression analysis. (A) *ibaG* expression in WT V. cholerae in different conditions measured by quantitative PCR. WT cells were grown in LB until exponential phase (OD_600nm_ of ∼0.3), then diluted 20-fold in LB at pH 5.5 or LB at pH 7 and grown for 1 h before RNA samples were processed for qPCR. WT cells were also grown in LB and M9 until exponential phase or stationary phase before processing samples for qPCR. The data were analyzed by the ΔΔCT method using *rpoC* mRNA as an internal control. Log_2_ fold change was calculated from ΔΔCT results. The reference is expression of *ibaG* strain in LB at pH 7. Experiments were performed with biological triplicates, and standard deviations are shown. (B) Phase contrast and fluorescence imaging of FM4-64-stained Δ*ibaG* cells complemented with *ibaG* expressed from plasmid pBAD33. Cells were grown to exponential phase in M9 medium supplemented with 0.2% arabinose. Scale bars, 2 μm. (C) Growth curves of indicated strains cells grown in M9 medium supplemented with 0.2% arabinose. OD_600nm_ was measured at 10-min intervals. Experiments were done in biological triplicate; standard deviations are shown. (D) Growth curves of WT and Δ*ibaG* strains grown in LB at pH 7 and LB at pH 5.5. OD_600nm_ was measured at 10-min intervals. Experiments were performed in triplicate; error bars show standard deviations. Download FIG S3, TIF file, 0.6 MB. Copyright © 2019 Fleurie et al. 2019 Fleurie et al. This content is distributed under the terms of the Creative Commons Attribution 4.0 International license.

**FIG 2:**
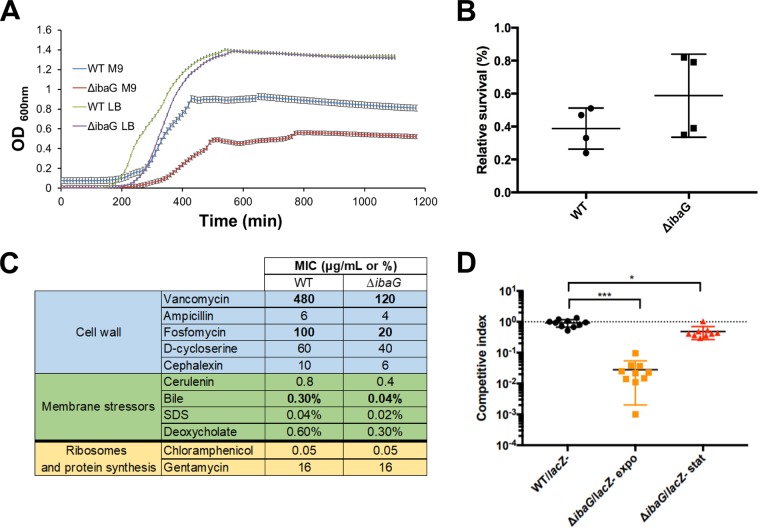
IbaG augments V. cholerae resistance to cell envelope stressors and promotes intestinal colonization. (A) Growth curves of WT and Δ*ibaG* V. cholerae grown in M9 medium. The OD_600_ was measured at 10-min intervals. Experiments were done in biological triplicate; error bars show standard deviations. (B) Acid resistance was determined by calculating the proportion of cells that survived during growth in acidic medium (LB at pH 5.5) versus in LB at pH 7. The numbers of colony**-**forming units per milliliter (CFU/ml) were determined for WT and Δ*ibaG* strains after 1-h growth in acidic medium from exponential-phase cultures. Experiments were performed in quadruplicate. (C) The MICs for the indicated agents were measured after 24 h growth in M9 medium at 37°C without shaking. The values shown represent the mean value obtained with two biological replicates done in technical quadruplicates for each strain. The concentrations are in micrograms per milliliter, except for bile, SDS, and deoxycholate which are shown as percentages. (D) Competitive indices for intestinal colonization for the indicated strain pairs. Suckling mice were inoculated with 1:1 mixture of Δ*ibaG* mutant and a *lacZ*-negative derivative of WT, made from log**-**phase (OD_600_ of ∼0.2) or overnight cultures. Competitive indices represent the output ratio (mutant strain CFU/*lacZ* mutant strain CFU) divided by the input ratio. Black horizontal lines represent geometric means, and colored horizontal lines show standard deviations. A Mann**-**Whitney U nonparametric test was used to assess statistical significance. Values that are significantly different are indicated by a bar and asterisks as follows: ***, *P* value of ≤0.0001; *, *P* value = 0.014.

10.1128/mBio.00790-19.4 FIG S4 Logarithmic scale growth curves. Representation of growth curves from Fig. 2A (panel A), Fig. S3C (panel B), Fig. S3D (panel C), and Fig. S6B (panel D) on logarithmic scale. Download FIG S4, TIF file, 0.7 MB. Copyright © 2019 Fleurie et al. 2019 Fleurie et al. This content is distributed under the terms of the Creative Commons Attribution 4.0 International license.

Growth analyses of Δ*ibaG* and WT V. cholerae revealed that the deletion markedly reduced the growth rate and terminal density of cells cultured in M9 medium (Fig. 2A and Fig. S4A). In LB medium, the effect was much less dramatic; the terminal densities of WT and Δ*ibaG* cultures were equivalent, but the mutant strain had a prolonged lag phase. The impaired growth of Δ*ibaG* V. cholerae contrasts with that of Δ*ibaG* E. coli, which displays enhanced growth (14), providing additional evidence that *ibaG* plays distinct roles in these organisms.

### IbaG promotes V. cholerae survival in the presence of factors that target the cell envelope.

Since BolA family proteins have been shown to participate in stress response pathways (2), we explored whether the absence of *ibaG* altered V. cholerae survival following exposure to a range of environmental stresses. Given the results of studies of E. coli IbaG, we first assessed whether V. cholerae IbaG modulates growth or survival under acidic conditions. Using a previously described acid resistance assay (19), we observed that the percentage survival of WT and Δ*ibaG* cells does not differ following a 1-h incubation in LB at pH 5.5 (Fig. 2B). Additionally, we found that the growth rate of WT and Δ*ibaG* V. cholerae in LB at pH 5.5 are very similar, although a longer lag period prior to growth was evident for the Δ*ibaG* cells (Fig. S3D and Fig. S4C). Furthermore, quantitative RT-PCR analysis revealed no change in *ibaG* expression following a 1-h exposure to acidified media (pH 5.5) (Fig. S3A). Thus, in contrast to E. coli *ibaG*, V. cholerae *ibaG* does not appear to promote bacterial resistance to acidic growth conditions.

To further evaluate the effect of IbaG on V. cholerae resistance to stressors, we determined the MICs of a wide variety of antimicrobial compounds for WT and *ΔibaG* cells. Notably, we observed that MICs for several antibiotics that target the cell wall (vancomycin, ampicillin, D-cycloserine, fosfomycin, cephalexin) were lower for the *ΔibaG* cells than for the WT (Fig. 2C). Additionally, we found that *ΔibaG* cells have increased sensitivity to bile, deoxycholate, SDS, and cerulenin (an inhibitor of fatty acid synthesis), all of which disrupt the outer membrane. In contrast, MICs of antibiotics that target the ribosomes and protein synthesis (chloramphenicol and gentamicin) were identical for the WT and the deletion strain. Collectively, these results suggest that the cell envelope of *ΔibaG* V. cholerae is more susceptible to disruption than that of WT cells, raising the possibility that IbaG regulates expression and/or activity of factors that contribute to envelope production or maintenance.

### Exponential-phase *ibaG* V. cholerae exhibit defective intestinal colonization.

Given the sensitivity of the *ΔibaG* V. cholerae to cell envelope stressors, including bile, we investigated whether *ibaG* contributes to the pathogen’s capacity to survive and proliferate in the intestines of suckling mice, a well-established model for studying V. cholerae intestinal colonization (20). Mice were orogastrically inoculated with 1:1 mixtures of WT and *ΔibaG* cells, and the relative abundance of the mutant cells within the small intestine was assessed at ∼24 h postinfection. Unexpectedly, the resulting competitive index for the *ΔibaG* cells was dependent upon the growth phase of cells used for the inoculum (Fig. 2D). When mice were infected with cells from stationary-phase cultures, colonization by the *ΔibaG* cells was minimally attenuated. In contrast, when mice were infected with cells from log-phase cultures, the *ΔibaG* cells exhibited an ∼50× decrease in colonization relative to the WT cells (Fig. 2D).

### *ΔibaG* cells have reduced peptidoglycan and phosphatidylethanolamine.

**FIG 3:**
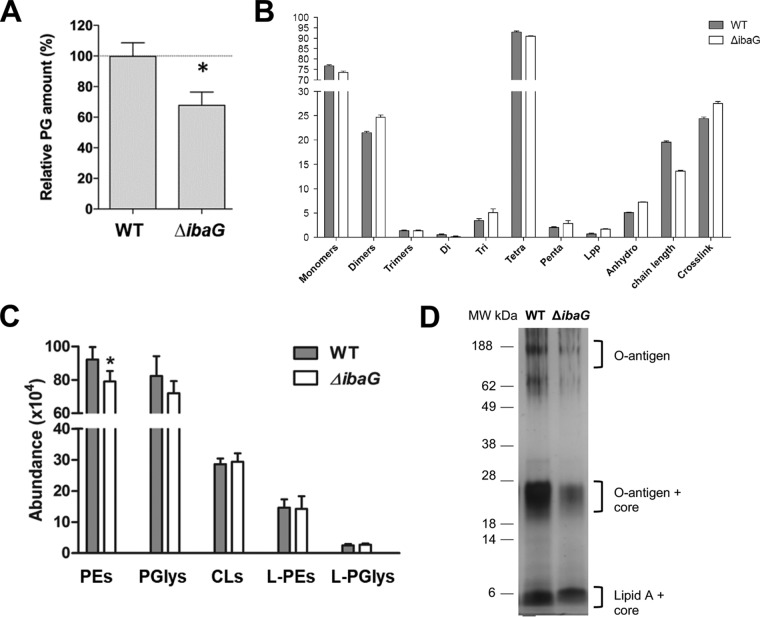
Comparison of cell envelope components in WT and *ΔibaG* V. cholerae. (A and B) Abundance (A) and composition (B) of peptidoglycan (PG) isolated from exponential-phase WT and *ΔibaG* V. cholerae. PG from each strain was analyzed in triplicate. Monomers, dimers, and trimers represent muropeptides, and Di (dimers), Tri (trimers), Tetra (tetramers), and Penta (pentamers) represent peptides. *,  *P* value of <0.01 by *t* test. (C) Quantification of different lengths and saturated forms of phosphatidylethanolamines (PEs), phosphatidylglycerols (PGlys), cardiolipins (CLs), lyso-PEs (L-PE), and lyso-PGs (L-PGlys) in whole-cell pellets from exponential-phase WT and *ΔibaG* V. cholerae. *,  *P* value of <0.01 by *t* test. (D) Silver stained SDS-PAGE of LPS isolated from exponential phase from WT and *ΔibaG* strains.

Our observation that *ΔibaG* cells are more sensitive than WT V. cholerae to agents that disrupt the cell wall or the outer membrane prompted us to further explore the structure and composition of these components in the *ΔibaG* background. Peptidoglycan (PG) was isolated from exponential-phase WT and *ΔibaG* cells, and its abundance and composition were measured. *ΔibaG* cells contained ∼25% less PG than WT cells did (Fig. 3A and Fig. S5A and B). Furthermore, there were differences in the abundance of several PG constituents (Fig. 3B). In particular, PG from *ΔibaG* cells had shorter average chain lengths, and it contained more than twice the WT level of Lpp, an outer membrane protein that is covalently linked to PG and helps anchor it to the outer membrane. Although the precise consequences of these changes are difficult to predict, it is likely that the reductions and alterations in *ΔibaG* PG contribute to the increased sensitivity of these cells to antibiotics that interfere with PG synthesis.

10.1128/mBio.00790-19.5 FIG S5 Chromatograms of peptidoglycan analysis. Chromatograms from analysis of peptidoglycan derived from WT and Δ*ibaG* strains in exponential phase (panel A). The muropeptides identified in each peak are described in the table (panel B). Download FIG S5, TIF file, 0.4 MB. Copyright © 2019 Fleurie et al. 2019 Fleurie et al. This content is distributed under the terms of the Creative Commons Attribution 4.0 International license.

Lipidomic analysis, using hydrophilic interaction liquid chromatography-ion mobility-mass spectrometry (HILIC-IM-MS) of exponential-phase WT and *ΔibaG* cells was also performed. The most obvious difference between the WT and Δ*ibaG* strains was an overall decrease in phosphatidylethanolamine (PE) (Fig. 3C), whereas phosphatidylglycerol (PGly) had a trend toward decrease and cardiolipin abundance was not significantly affected in the Δ*ibaG* strain (Fig. 3C). Since the biogenesis of 3-deoxy-d-manno-octulosonic acid (Kdo) requires PE (21, 22), and PE deficiency downregulates LPS biosynthesis in E. coli (21), we also quantified lipopolysaccharide (LPS) present in exponential-phase WT and mutant cells. The Δ*ibaG* strain contained significantly less LPS than the WT strain (Fig. 3D). Decreased LPS levels might contribute to the Δ*ibaG* mutant’s increased sensitivity to membrane-disrupting factors such as bile and SDS and contribute to its colonization defect.

### Transposon insertion site sequencing analysis links IbaG to cell envelope biogenesis.

**FIG 4:**
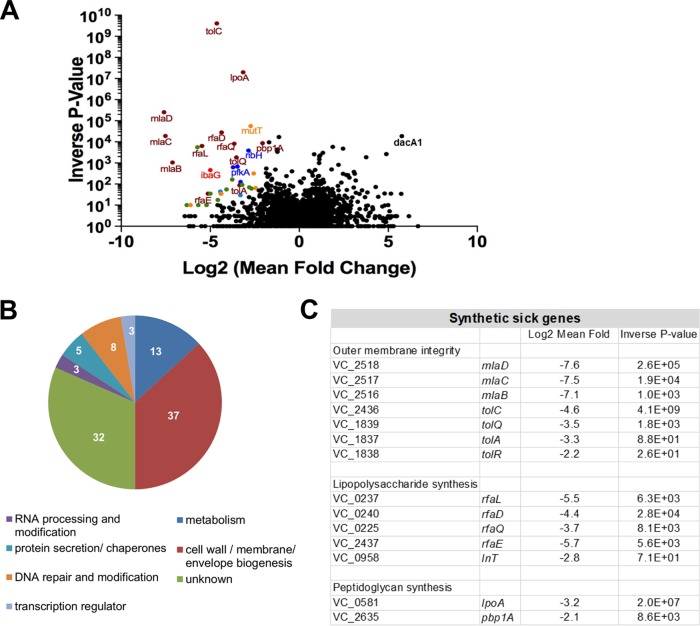
Transposon insertion sequence-based analyses of *ibaG* genetic interactions. (A) Volcano plots depicting the relative abundance of read counts mapped to individual genes in transposon libraries made in the Δ*ibaG* mutant versus WT. For each gene, the log_2_ mean fold change (*x* axis) and associated *P* value (*y* axis) are shown. Genes shown in color are considered significantly underrepresented compared to the WT (mean fold change of >2 and *P* value of <0.05), and the colors correspond to the functional classification represented in panel B. A comprehensive list of the genes over- or underrepresented in the Δ*ibaG* library with a mean fold change of >2 and a *P* value of <0.05 is shown in Table S1 in the supplemental material. (B) Functional classification of the genes classified as underrepresented in the Δ*ibaG* background. The numbers represent the percentage of genes (of 38 total) in each category. (C) Underrepresented genes in the Δ*ibaG* insertion library that are involved in cell envelope integrity and/or LPS and peptidoglycan synthesis.

To gain further insight into the pathways and processes affected by *ibaG*, we conducted a comparative transposon insertion sequencing analysis to identify transposon insertions that are underrepresented in the Δ*ibaG* background relative to the WT strain. Loci for which fewer insertions are identified in the Δ*ibaG* versus WT background are candidates for synthetic lethality with *ibaG* and may contribute to processes that are also impaired by the absence of *ibaG*. We identified 38 genes that were underrepresented at least twofold in the Δ*ibaG* insertion library with a *P* value of <0.05 (Fig. 4A and B). Notably, more than one third of these loci are involved in pathways linked to cell envelope integrity and/or LPS and PG synthesis (Fig. 4B). They include *mlaBCD*, which along with *mlaA* encode an ABC transport system involved in maintaining outer membrane lipid asymmetry, PG biosynthetic gene *pbp1A* and its activator *lpoA*, several loci in the *rfa* cluster, which contains many of the genes for LPS synthesis, and genes encoding components of the *tol* system, which regulates PBP1B and is important for outer membrane stability (23) (Fig. 4C). A Δ*mlaBCD* Δ*ibaG* double mutant exhibited a more severe growth defect than the Δ*ibaG* single mutant, consistent with the results of the transposon screen (Fig. S6B and Fig. S4D). Collectively, these results provide further support for the idea that *ibaG* is important for biogenesis and/or maintenance of the cell envelope, so that Δ*ibaG* cells are particularly sensitive to additional mutations that affect this structure.

10.1128/mBio.00790-19.6 FIG S6 Genetics and biochemical interactions of IbaG. (A) Bacterial adenylate cyclase two-hybrid analysis of IbaG and Grx4 interactions. Colonies of *cya*-negative strains producing T25 and T18 fusions of the respective proteins on LB medium supplemented with X-Gal and IPTG are shown. (B) Growth curves of indicated strains cells grown in LB medium (upper panel) or M9 medium (lower panel). OD_600nm_ was measured at 10-min intervals. Experiments were done in biological triplicate; standard deviations are shown. Download FIG S6, TIF file, 0.7 MB. Copyright © 2019 Fleurie et al. 2019 Fleurie et al. This content is distributed under the terms of the Creative Commons Attribution 4.0 International license.

10.1128/mBio.00790-19.8 TABLE S1 Transposon insertion sequencing analysis. Comprehensive list of the genes over- or underrepresented in the Δ*ibaG* library with a mean fold change of >2 and a *P* value of <0.05 and transposon insertion sequencing raw data with a list of the number of reads, log_2_ fold change, *P* value, and standard deviation obtained for all the genes for the Δ*ibaG* and WT libraries. Download Table S1, PDF file, 1.3 MB. Copyright © 2019 Fleurie et al. 2019 Fleurie et al. This content is distributed under the terms of the Creative Commons Attribution 4.0 International license.

We also identified 34 loci that are overrepresented at least two-fold in the Δ*ibaG* insertion library with a *P* value of <0.05 (Fig. 4A; see also Table S1 in the supplemental material). Intriguingly, these included *dacA1* (*pbp5*), which encodes a low-molecular-weight PG binding protein, which has been found to be necessary for normal V. cholerae growth and morphology (24). V. cholerae Δ*dacA1* cells exhibited branches and aberrant poles and are wider as well as elongated (24), phenotypes that are strikingly reminiscent of the morphology of Δ*ibaG* cells. Disruption of *dacA1* also impedes V. cholerae cell growth and viability. However, in the *ibaG* background, the effects of *dacA1* disruption may be less detrimental, perhaps because they affect processes that have already been disrupted.

### IbaG interacts with numerous iron-sulfur cluster proteins.

**FIG 5:**
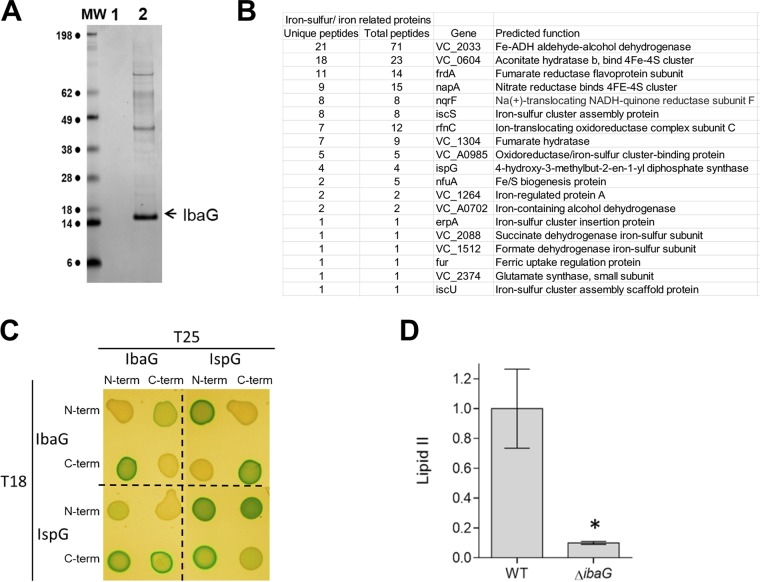
IbaG interacts with iron-sulfur cluster proteins. (A) Coomassie blue-stained gel of proteins recovered after TAP purification from cell extracts of V. cholerae producing TAP-TAG only (lane 1) or IbaG-TAP-TAG (lane 2). Bands of interest were analyzed by mass spectrometry. (B) IbaG-interacting proteins identified by mass spectrometry that are iron-sulfur-containing proteins or facilitate biogenesis of iron-sulfur proteins (a complete list of interacting proteins is presented in Table S2). (C) Bacterial adenylate cyclase two-hybrid analysis of IbaG and IspG interactions. Colonies of *cya*-negative strains producing T25 and T18 fusions of the respective proteins on LB medium supplemented with X-Gal and IPTG are shown. (D) Lipid II quantification in WT and Δ*ibaG* strains grown in M9 medium to exponential phase. *,  *P* value of <0.01 by *t* test.

In addition to the genetic interactions revealed by transposon insertion sequencing, we also identified proteins that interact with IbaG. In E. coli, IbaG interacts with Grx4, forming [2Fe-2S]-bridged heterodimers (16). Bacterial two-hybrid analysis demonstrated that the V. cholerae versions of these proteins also interact (Fig. S6A). To further our knowledge of IbaG’s partners, epitope-tagged IbaG was affinity purified, and copurified proteins were identified via tandem mass spectrometry analysis (Fig. 5A and Table S2). Notably, a third of the proteins that copurified with IbaG have roles in either iron-sulfur cluster biogenesis (e.g., IscS, IscU), use iron-sulfur clusters as cofactors (e.g., NqrF, IspG), or bind iron-sulfur clusters and serve as carriers to transfer them to other proteins (e.g., NfuA, ErpA) (Fig. 5B). These interactions suggest that V. cholerae IbaG contributes to iron trafficking and can bind iron-sulfur clusters as shown for E. coli IbaG (16). Consistent with this idea, we found that the activities of the iron-sulfur-containing enzymes succinate dehydrogenase, fumarase, and glutamate synthase were reduced in Δ*ibaG* versus WT cells (Fig. S7).

10.1128/mBio.00790-19.7 FIG S7 Iron-sulfur-containing enzymes exhibit reduced activities in the Δ*ibaG* mutant. Succinate dehydrogenase (SDH) (panel A), fumarase (panel B), and glutamate synthase (panel C) activities were measured in crude extracts from exponentially growing WT and Δ*ibaG* cells. The measurements were performed according to the manufacturer’s instructions. Experiments were done in biological triplicate; standard deviations are shown. *, *P* value of 0.06 Download FIG S7, TIF file, 0.2 MB. Copyright © 2019 Fleurie et al. 2019 Fleurie et al. This content is distributed under the terms of the Creative Commons Attribution 4.0 International license.

10.1128/mBio.00790-19.9 TABLE S2 Total proteins identified by mass spectrometry. List of the proteins identified by mass spectrometry and their functional classification represented on a pie chart (numbers represent the percentages of genes in each category). Download Table S2, PDF file, 0.1 MB. Copyright © 2019 Fleurie et al. 2019 Fleurie et al. This content is distributed under the terms of the Creative Commons Attribution 4.0 International license.

Factors involved in the synthesis of LPS and other lipids as well as the Tol-Pal system were also identified (Table S2), providing further support for the idea that *ibaG* is important for biogenesis and/or maintenance of the cell envelope.

IspG, one of the proteins that copurified with IbaG, contributes to the synthesis of precursors to lipid II, which mediates a critical early step in PG synthesis, suggesting a possible explanation for the reduced PG in the Δ*ibaG* cells. Bacterial two-hybrid analysis confirmed the interaction between IbaG and IspG (Fig. 5C). Furthermore, UPLC chromatography coupled to MS/MS analysis of lipid II levels in exponential-phase WT and Δ*ibaG* cells revealed markedly lower abundance (10-fold change) of lipid II in the Δ*ibaG* cells (Fig. 5D). Reduced lipid II levels (and subsequent effects on PG synthesis and homeostasis) could also contribute to the Δ*ibaG* mutant’s increased sensitivity to antibiotics that target cell wall synthesis.

## DISCUSSION

Here, we characterized the V. cholerae BolA-like protein IbaG. IbaG, which is encoded in the midst of loci that contribute to the biogenesis and maintenance of the cell envelope, likewise appears to modulate production and/or integrity of the V. cholerae envelope. Mutants lacking *ibaG* contain reduced amounts of peptidoglycan and LPS and have altered lipid profiles. Likely as a result of altered cellular barriers, Δ*ibaG* V. cholerae exhibit elevated sensitivity to antibiotics that target the cell wall and to detergents and other envelope-disrupting factors. The mutant also displays impaired capacity to colonize the intestine in an animal model of infection. Mutagenesis and biochemical analyses provided further support for the idea that V. cholerae *ibaG* contributes to cell envelope biogenesis and suggest that it may do so by modulating assembly and/or trafficking of iron-sulfur clusters.

Although E. coli and V. cholerae *ibaG* genes have significant homology and share genomic context, our findings revealed that deletion of *ibaG* has markedly distinct consequences in these two gammaproteobacteria. While no morphological defect was found for Δ*ibaG* E. coli (14), Δ*ibaG* V. cholerae cells were frequently elongated, branched, and wider than WT V. cholerae. Furthermore, E. coli *ibaG* is induced by acid stress and promotes survival in response to acid challenge (14), whereas neither phenotype was apparent in V. cholerae. Such diversity of function has previously been observed for a variety of E. coli/V. cholerae homolog pairs involved in cell wall regulation (e.g., DacA-1/PBP5, PBP1A, AmiB, and NlpD) (24–26). Similarly, BolA has been found to play distinct roles in Pseudomonas fluorescens and E. coli (27), suggesting that each factor may be adapted to meet the specific needs of the host organism.

Interestingly, the *ibaG* mutant exhibited aberrant morphology during exponential-phase growth but normal size and shape during stationary phase. Growth in minimal versus rich media also exacerbated the mutant’s distorted morphology. It is possible that the increased demand for cell wall components associated with cell growth and division, coupled with the reduced levels of PG, the PG biosynthetic factor lipid II, and LPS, may contribute to the *ibaG* mutant’s inability to maintain normal morphology during rapid growth. Potentially arguing against this hypothesis is the slower growth in minimal compared to LB media; furthermore, a recent analysis of E. coli revealed that nutrient limitation tended to reduce the effect of mutations on cell morphology (28). Given the apparent link between *ibaG* and iron-sulfur cluster-linked processes, it is possible that differences in iron availability in the minimal media contribute to the increased shape alterations rather than, or in addition to, the extent of nutrients.

In addition to its effect on cell morphology, the growth phase of the *ibaG* mutant also influenced its capacity to compete against WT V. cholerae in colonizing the intestine of a model animal host. When stationary-phase cultures were used to infect infant mice, the *ibaG* mutant exhibited a less than 2-fold deficit in colonization relative to the coinoculated WT strain; in contrast, log-phase cells exhibited an ∼50-fold deficit. We speculate that replicating *ibaG* cells may be particularly sensitive to host protective factors that are encountered early in the infection process (e.g., bile), and therefore may be preferentially eliminated at the beginning of the infection process. Such a disadvantage is consistent with the mutant’s increased susceptibility to cell envelope-disrupting factors *in vitro*. The normal morphology of the *ibaG* cells in stationary phase may be indicative of a relatively unperturbed cell envelope that is more able to withstand such host defenses. Although the stationary-phase inoculum gives rise to replicating (and presumably morphologically aberrant) cells *in vivo*, replication may occur after cells have reached intestinal sites where they are not exposed to high concentrations of agents such as bile.

Our analysis of proteins that copurify with IbaG provided possible explanations for the reduced levels of cell envelope components observed in the Δ*ibaG* mutant. Several members of the RfB family, responsible for O-antigen synthesis, were found to interact directly or indirectly with IbaG; the absence of such interactions may contribute to Δ*ibaG* V. cholerae’s LPS deficiency. Similarly, an interaction between IbaG and IspG, which contributes to the synthesis of precursors to lipid II, may underlie the reduction in lipid II and PG that was observed in the *ibaG* mutant. Deficiencies in PG and LPS likely lead to formation of a cell wall and outer membrane that are defective in cell division and maintenance of turgor pressure and sensitive to membrane-disrupting factors, accounting for some of the mutant’s phenotypes.

Notably, analysis of factors that copurify with IbaG also suggests that V. cholerae IbaG is linked to production or trafficking of iron-sulfur clusters. We found that IbaG is able to interact directly or indirectly with several proteins involved in iron-sulfur biogenesis or containing iron-sulfur clusters, including IscU, IscS, and NfuA. Given the pivotal role of iron-sulfur-containing proteins in numerous cellular processes, including central carbon metabolism, DNA/RNA metabolism, signal transduction, and stress responses, their interactions with IbaG suggest multiple ways that *ibaG* deletion might disrupt cellular physiology, which may account for its pleotropic effects. Finally, perhaps even more remarkable than the extreme distortion of the shape and size of exponential-phase Δ*ibaG* V. cholerae is their capacity to regain normal shape and size; unraveling the mechanisms that enable this recovery should yield insight into the plasticity of bacterial shape-determining pathways.

## MATERIALS AND METHODS

### Strains, media, and growth conditions.

All V. cholerae strains described in this study are derivatives of V. cholerae El Tor strain N16961 (29). E. coli DH5α λpir was used for general cloning purposes. E. coli SM10 λpir was used for conjugation. Cells were grown at 37°C in Luria-Bertani (LB) medium, or M9 medium supplemented with 0.2% glucose (M9). Media were supplemented when needed with 200 μg/ml streptomycin, 50 μg/ml carbenicillin (V. cholerae), or 20 μg/ml chloramphenicol (E. coli). For induction of genes under the control of arabinose-inducible promoters, strains were grown in media supplemented with 0.2% l-arabinose.

For growth curves, at least three replicates per strain and condition were grown in 200 μl medium in a 100-well honeycomb plate inoculated 1:100 from an exponentially growing preculture (optical density at 600 nm [OD_600_] of ∼0.02) and analyzed in a BioScreen C growth plate reader at 10 min intervals. Data were analyzed using Microsoft Excel.

### Construction of plasmids and strains.

Plasmids and strains are described in Table S3 in the supplemental material. Plasmids were generated with Gibson assembly (30). In-frame deletions were introduced using sucrose-based counterselection with *sacB*-containing suicide vector pCVD442 (31). Proteins were overproduced by placing the respective gene under the control of the *araC* (P_BAD_) promoter using vector pBAD33 (32).

10.1128/mBio.00790-19.10 TABLE S3 Strains, plasmids, and oligonucleotides used in this study. Download Table S3, PDF file, 0.1 MB. Copyright © 2019 Fleurie et al. 2019 Fleurie et al. This content is distributed under the terms of the Creative Commons Attribution 4.0 International license.

### Microscopy.

Microscopy was performed using exponentially growing cells (OD_600_ of ∼0.2 to 0.4) or stationary-phase cells. Bacteria were immobilized on 1% agarose pads and visualized using a Nikon Eclipse Ti microscope equipped with an Andor NeoZyla camera and a 100× oil phase 3 1.4-numerical-aperture (NA) objective. Images were processed using ImageJ (http://rsb.info.nih.gov/ij/) and MicrobeTracker (33) to generate cell length and width distribution histograms. The mean width, which is the average of the width over the entire cell, was measured instead of the maximum width, given the variation in width for *ibaG* cells. A nonparametric statistical analysis (Mann-Whitney U test) was performed using Prism because of the nonnormal distribution of cell sizes in the mutant strain (34). Staining with FM4-64 was performed as described previously (35). Briefly, cells were grown to exponential phase or stationary phase in LB or M9 medium, and 1 μg/ml of FM4-64 was added to the cultures and incubated for 5 min at room temperature and imaged as described above.

### Bacterial two-hybrid assay.

The adenylate cyclase-based bacterial two-hybrid technique was used as previously published (36). Briefly, IbaG, IspG, and Grx4 were fused to the isolated T18 and T25 catalytic domains of the *Bordetella* adenylate cyclase. After transformation of the two plasmids producing the fusion proteins into the reporter BTH101 strain, plates were incubated at 30°C for 48 h. Three independent colonies for each transformation were inoculated into 600 μl of LB medium supplemented with ampicillin, kanamycin, and isopropyl-β-d-thiogalactopyranoside (IPTG) (0.5 mM). After overnight growth at 30°C, 10-μl portions from each culture were dropped onto LB plates supplemented with ampicillin, kanamycin, bromo-chloro-indolyl-galactopyranoside (X-Gal) (40 μg/ml) and IPTG (0.5 mM) and incubated for 16 h at 30°C. The experiments were performed at least in triplicate, and a representative result is shown.

### Purification and visualization of lipopolysaccharide.

Lipopolysaccharide (LPS) was extracted following the protocol described by Davis and Goldberg (37). Briefly, pelleted bacteria harvested from exponential-phase cultures were resuspended in 200 μl of SDS buffer (2% β-mercaptoethanol, 2% SDS, and 10% glycerol in 0.05 M Tris-HCl [pH 6.8]) and boiled for 15 min. The samples were treated first with 5 μl of DNase and RNase (10 mg/ml) for 30 min at 37°C and then with 10 μl of proteinase K (10 mg/ml) for 3 h at 59°C. Two hundred microliters of ice-cold Tris-saturated phenol was then added, and the samples were incubated for 15 min at 65°C, with occasional vortexing. One milliliter of diethyl ether was added before centrifugation for 10 min at 20,600 × *g*, and the bottom blue layer was extracted. The extractions with Tris-saturated phenol and diethyl ether were repeated twice before adding 2× SDS buffer to the samples. The samples (15 μl) were run on SDS-polyacrylamide gels. LPS was visualized using the Pro-Q Emerald 300 Lipopolysaccharide Gel Stain kit (Molecular Probes) according to the manufacturer’s instructions.

### Acid resistance assay.

Bacterial cultures were grown in LB until they reached an OD_600_ of ∼0.3, then diluted 20-fold in LB at pH 5.5, and incubated for 1 h before plating serial dilutions to determine the number of CFU per milliliter for each strain. The numbers of CFU per milliliter were similarly determined for growth in LB at pH 7 prior to the acid challenge, and the relative survival (CFU/ml at pH 5.5/CFU/ml at pH 7) was calculated to determine acid resistance for both strains. The pH of the LB broth was adjusted using 1 mM HCl.

### Quantitative PCR.

Cells from overnight (stationary-phase) cultures were inoculated in triplicate into 5 ml LB or M9 and grown at 37°C until exponential phase (OD_600_ of ∼0.3) or stationary phase. Total RNA was extracted from harvested cells with TRIzol reagent (Life Technologies). RNA was treated with Turbo DNase I for 30 min (Life Technologies) and subjected to quantitative reverse transcriptase PCR (qRT-PCR) as previously described (38). Briefly, 1 μg total RNA was used for the reverse transcription reaction with Superscript III first strand synthesis system with random hexamers (Life Technologies). Real-time PCR amplification of the synthesized cDNA was conducted using the Fast SYBR green Master Mix kit (Life Technologies). Each of the three biological replicates was analyzed in technical triplicate on the StepOnePlus platform (Life Technologies) using primers shown in Table S3. The data were analyzed by the ΔΔ*C_T_* method using *rpoC* mRNA as an internal control. Log_2_ fold change was calculated from the ΔΔ*C_T_* results.

### Transposon mutant library construction and sequencing.

Transposon insertion sequencing was performed as described previously (39). Transposon libraries were created in WT and Δ*ibaG* V. cholerae using the transposon delivery vector pSC189. A total of ∼600,000 transposon mutants were generated for each strain. Genomic DNA was purified and sequenced on an Illumina MiSeq benchtop sequencer (Illumina, San Diego, CA). Sequenced reads were mapped onto the V. cholerae N16961 reference genome, and all TA sites were tallied and assigned to annotated genes as previously described (40). Insertion sites were identified as described previously (39), and significance was determined using the Con-Artist pipeline.

### MIC assay.

MIC assays were performed using an adaptation of a standard methodology with exponential-phase cultures (41). In short, serial twofold dilutions of the antimicrobial agents were prepared in 50 μl of LB in a 96-well plate. Then, to each well was added 50 μl of a culture prepared by diluting an overnight culture 1,000-fold into fresh LB broth, growing it for 1 h at 37°C, and again diluting it 1,000-fold into fresh medium. The plates were then incubated without shaking for 24 h at 37°C.

### Peptidoglycan purification and analysis.

Peptidoglycan (PG) samples were prepared and analyzed in triplicate as described previously (42, 43). Briefly, 1 liter of exponential WT and Δ*ibaG* strains grown in LB were harvested and boiled in 5% SDS for 2 h. Sacculi were repeatedly washed with MilliQ water by ultracentrifugation (110,000 rpm, 10 min, 20°C) until total removal of the detergent, followed by digestion with pronase E (100 μg/ml) for 1 h at 60°C. Finally, samples were treated with muramidase (100 μg/ml) for 16 h at 37°C. Muramidase digestion was stopped by boiling, and coagulated proteins were removed by centrifugation (10 min, 14,000 rpm). For sample reduction, the pH of the supernatants was adjusted to pH 8.5 to 9.0 with sodium borate buffer, and sodium borohydride was added to a final concentration of 10 mg/ml. After incubating for 30 min at room temperature, the pH of the samples was adjusted to pH 3.5 with orthophosphoric acid.

Ultrahigh performance liquid chromatography (UPLC) analyses of muropeptides were performed on a Waters UPLC system (Waters Corporation, USA) equipped with an Acquity UPLC BEH C_18_ column (130 Å; 1.7 μm; 2.1 mm by 150 mm) (Waters, USA) and a dual wavelength absorbance detector. Elution of muropeptides was detected at 204 nm. Muropeptides were separated at 35°C using a linear gradient from buffer A (50 mM phosphate buffer [pH 4.35]) to buffer B (50 mM phosphate buffer [pH 4.95], 15% [vol/vol] methanol) in a 20 min run, with a 0.25-ml/min flow rate.

Relative total PG amounts were calculated by comparison of the total intensities of the chromatograms (total area) from three biological replicates normalized to the same OD_600_ and extracted with the same volumes. Muropeptide identity was confirmed by MS/MS analysis, using a Xevo G2-XS QTof system (Waters Corporation, USA). Quantification of muropeptides was based on their relative abundances (relative area of the corresponding peak) normalized to their molar ratio.

### *In vivo* colonization assay.

Intestinal colonization in infant mice was conducted as described previously (44). Cells for the exponential-phase inoculum were grown separately to an OD_600_ of ∼0.3 in LB and then diluted 1:100 in the same medium prior to mixing 1:1. Cells for the stationary-phase inoculum were grown separately overnight in LB at 37°C and then diluted 1:1000 in LB prior to mixing. Infant mice were gavaged with 50 μl of the 1:1 inoculum mixture and then sacrificed after 24 h. Dilutions of homogenized small intestines were plated on LB agar to enumerate CFU. Competitive indices (CI) were calculated as the ratio of mutant to WT bacteria isolated from intestines normalized to the input ratio. Statistical significance was determined using a Mann-Whitney U *t* test (*P* value of <0.01).

### Lipid II quantification.

Precursor extraction was performed as described previously and performed in triplicate (45). Briefly, 500 ml of WT and Δ*ibaG* strains were grown in LB to an OD_600_ of 0.45. Cells were harvested, resuspended in 5 ml phosphate-buffered saline (PBS) in 50-ml flasks containing 20 ml CHCl_3_-methanol (1:2). The mixture was stirred for 1 h at room temperature and centrifuged for 10 min at 4,000 × *g* at 4°C. The supernatant was transferred to 250-ml flasks containing 12 ml CHCl_3_ and 9 ml PBS, stirred for 1 h at room temperature, and centrifuged for 10 min at 4,000 × *g* at 4°C. The interface fraction (between the top aqueous and bottom organic layers) was collected and vacuum dried. To remove the lipid tail, samples were resuspended in 100 μl dimethyl sulfoxide (DMSO), 800 μl H_2_O, and 100 μl ammonium acetate (100 mM; pH 4.2). This mixture was boiled for 30 min, dried in a vacuum, and resuspended in 300 μl H_2_O.

Samples were analyzed by UPLC chromatography coupled to MS/MS analysis, using a Xevo G2- XS QTof system (Waters Corporation, USA). Precursors were separated at 45°C using a linear gradient from buffer A (0.1% formic acid in water) to buffer B (0.1% formic acid in acetonitrile) in an 18-min run, with a 0.25 ml/min flow rate. A library of compounds was used to target the identification of peptidoglycan precursors and possible intermediates, although only lipid II was detected. Lipid II amounts were calculated based on the integration of the peaks (total area), normalized to the culture OD.

### Tandem affinity purification assay and mass spectrometry analysis.

IbaG was purified using a standard tandem affinity purification (TAP) protocol. Briefly, an overnight culture of V. cholerae encoding IbaG C-terminally tagged with calmodulin binding protein, a tobacco etch virus (TEV) cleavage site, and protein A was used to inoculate 500 ml of LB (1/100 dilution), which was grown for 5.5 h at 37°C with shaking, and then the cells were pelleted and washed in cold PBS. Tandem affinity purification was then performed as described before (46, 47). Then, the cells were broken using an Emulsilfex C3 (Avestin) in the presence of protease inhibitor (Complete; Roche). The lysate was used to bind to 200 μl of IgG Sepharose beads (Amersham Biosciences) for 2 h at 4°C using a disposable chromatography column (Bio-Rad). The IgG-Sepharose column was washed with 35 ml of protein A binding buffer (10 mM Tris-HCl [pH 8], 150 mM NaCl, 0.1% NP-40), followed by 10 ml of the TEV cleavage buffer (10 mM Tris-HCl [pH 8], 150 mM NaCl, 0.1% NP-40, 0.5 mM EDTA, 1 mM dithiothreitol [DTT]). Cleavage with TEV was performed using 10 μl (100 U) of AcTEV (Invitrogen) in 1 ml of cleavage buffer for 2 h at 4°C. Calmodulin-Sepharose (Stratagene) purification was performed as described previously (47). Independent tandem affinity purifications followed by mass spectrometry analysis was performed at least twice.

### Homology alignments and structural predictions.

The 3D homology models of BolA and IbaG from V. cholerae were constructed using the Phyre2 Server (48) (www.sbg.bio.ic.ac.uk/~phyre2). The program used BolA (PDB ID 2DHM) and YrbA (PDB ID 1NY8) from E. coli as the template to generate the models. PyMOL (The PyMOL Molecular Graphics System, version 1.2r3pre; Schrödinger, LLC.) was used to generate the figure. Multiple sequence alignments were assembled from selected pairwise alignments and converted to clustal format (49) and uploaded to Ali2D (https://toolkit.tuebingen.mpg.de/#/tools/ali2d) to generate images for secondary structure similarity (50).

### Lipid quantification.

Extraction of lipids from V. cholerae pellets was performed using the method of Bligh and Dyer, as described previously (51–54). Briefly, dried pellets (4.1 ± 6.0 mg for the Δ*ibaG* mutant and 71.9 ± 4.2 mg for strain N16961; *P = *0.62 by *t* test) in 10 ml glass centrifuge tubes (Fisher) were reconstituted in 1 ml of H_2_O (Fisher Optima LC-MS) and sonicated for 30 min in an ice bath, followed by the addition of 4 ml of chilled 1:2 chloroform-methanol (Fisher Optima LC-MS) extraction solution. Following mixing and centrifugation, the organic phase of the two-layer extraction was collected into fresh glass centrifuge tubes and dried in a vacuum concentrator. Extracts were reconstituted with 500 μl of 1:1 chloroform-methanol solution. For analysis, 5 μl of extract was transferred to an LC vial, dried, and reconstituted with 100 μl of 2:1 acetonitrile-methanol solution. A pooled quality control sample was prepared from 15 μl of each sample.

Characterization of the V. cholerae lipidome was performed by hydrophilic interaction liquid chromatography (HILIC) coupled to ion mobility-mass spectrometry (IM-MS), as described previously (51). Data were acquired for each sample in both positive and negative electrospray ionization modes over the range of 50 to 1200 *m/z*. Alignment of HILIC-IM-MS data and peak detection were performed in Progenesis QI (Nonlinear Dynamics) with the default “All Compounds” normalization method. The negative-mode data set was filtered by analysis of variance (ANOVA) *P* ≤ 0.1, which retained 528 features. The top 10 features for phosphatidylethanolamines (PEs) and phosphatidylglycerols (PGlys) and the top 5 features for lyso-phosphatidylethanolamines (L-PEs) and lyso-phosphatidylglycerols (L-PGlys) that meet the ANOVA *P* threshold were summed in the figure. Student’s *t* tests for two samples were performed using a two-tailed distribution and equal variance. Identification of lipid species was performed against the METLIN database within 15 ppm mass accuracy (55, 56).

### Enzyme assays.

Succinate dehydrogenase and fumarate and glutamate synthase activities were measured according to the manufacturer’s protocols (Sigma-Aldrich). Briefly, 4 ml of exponential-phase cultures of WT and Δ*ibaG* strains were pelleted and homogenized in 100 μl of the succinate dehydrogenase, fumarase, or glutamate synthase assay buffer. Samples were then centrifuged at 10,000 × *g* for 5 min to remove insoluble material. 50 μl portions of the samples were then used for the assays. Experiments were performed in biological triplicate.
