# Supplementary figures and images for: A Vibrio cholerae BolA-Like Protein Is Required for Proper Cell Shape and Cell Envelope Integrity
Source: mBio. 2019 Jul 9;10(4):e00790-19. doi: 10.1128/mBio.00790-19 (PMC6747721; doi:10.1128/mBio.00790-19)

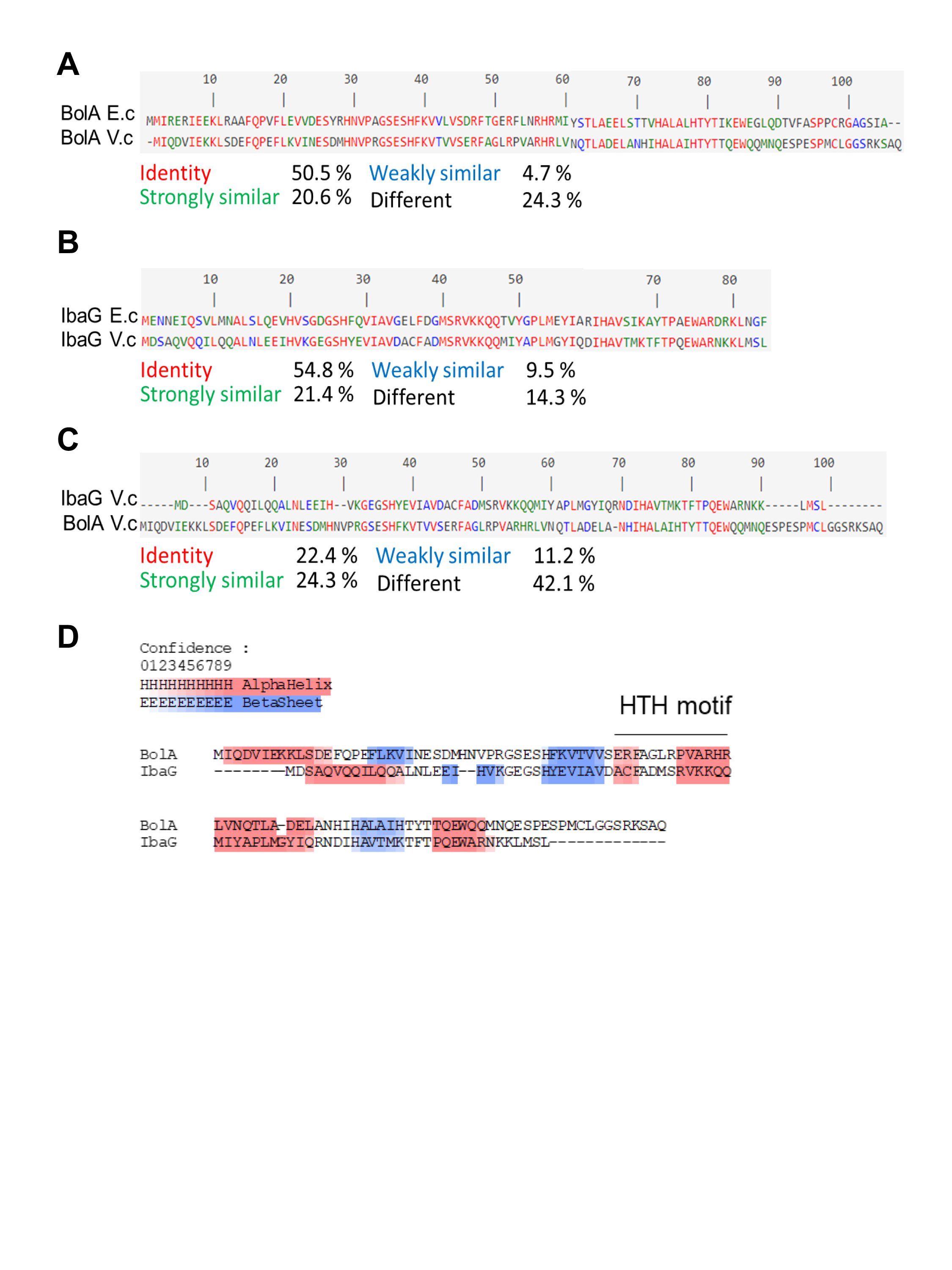

Supplement: FIG S1 [file mBio.00790-19-sf001.tif]

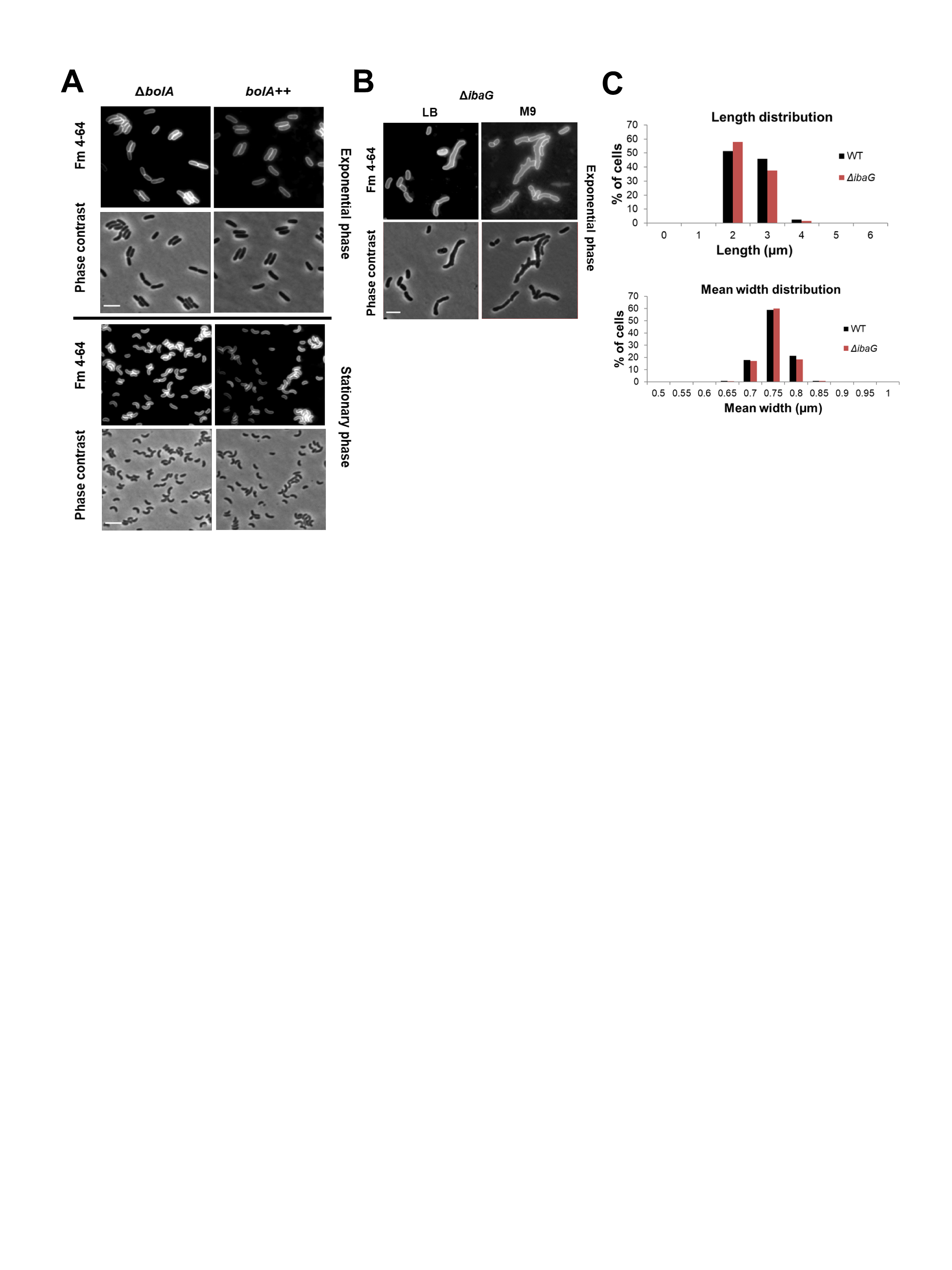

Supplement: FIG S2 [file mBio.00790-19-sf002.tif]

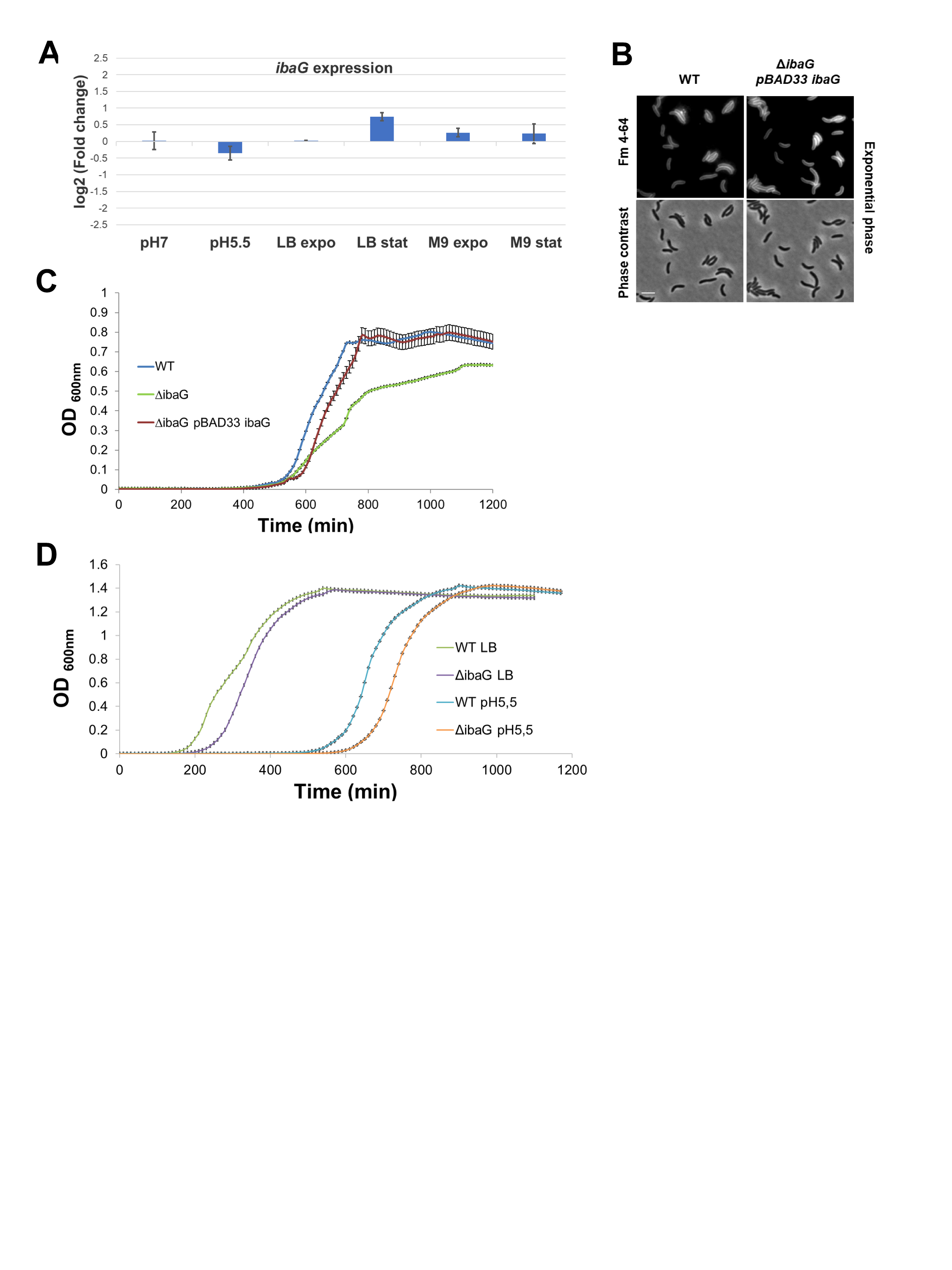

Supplement: FIG S3 [file mBio.00790-19-sf003.tif]

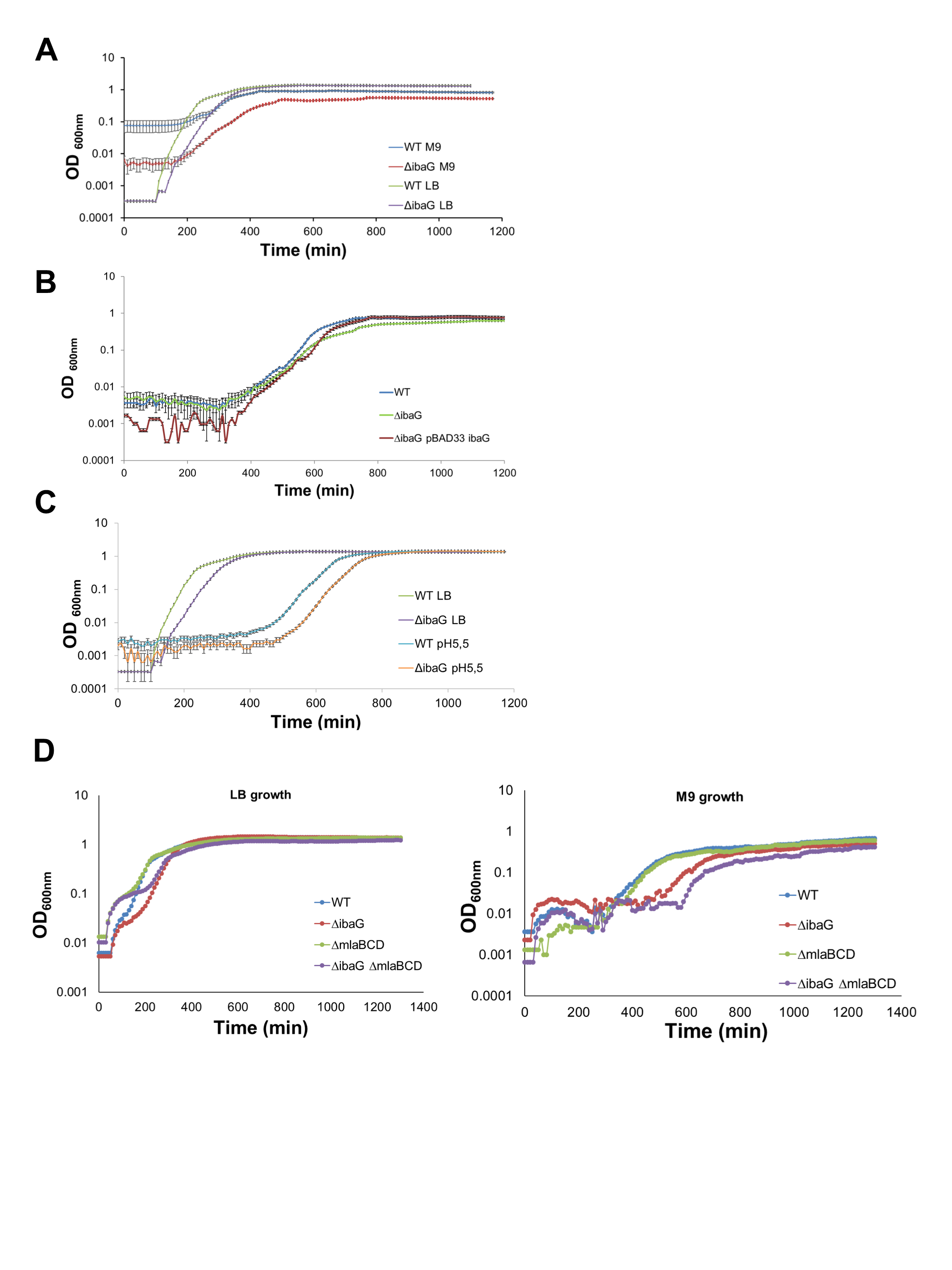

Supplement: FIG S4 [file mBio.00790-19-sf004.tif]

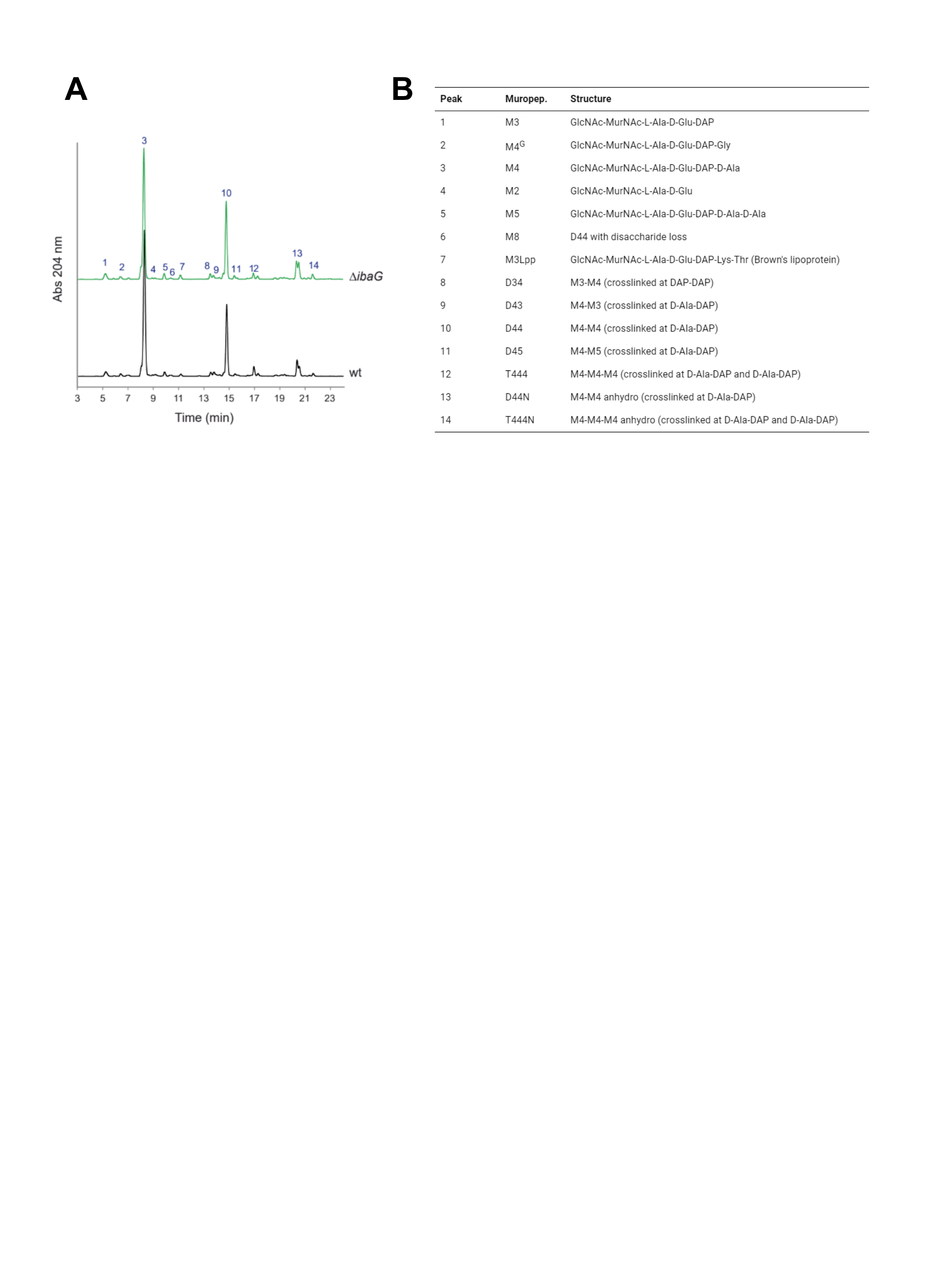

Supplement: FIG S5 [file mBio.00790-19-sf005.tif]

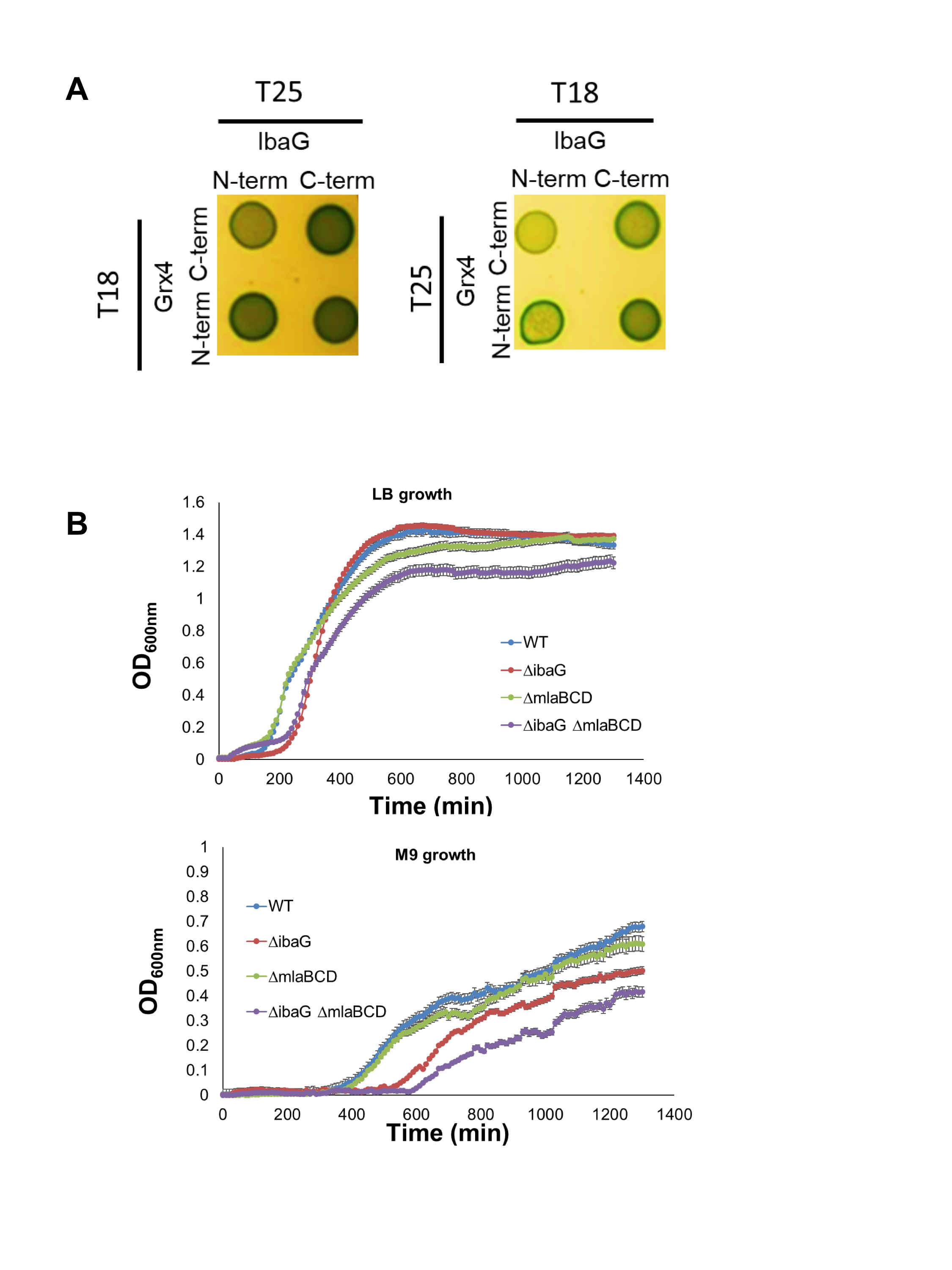

Supplement: FIG S6 [file mBio.00790-19-sf006.tif]

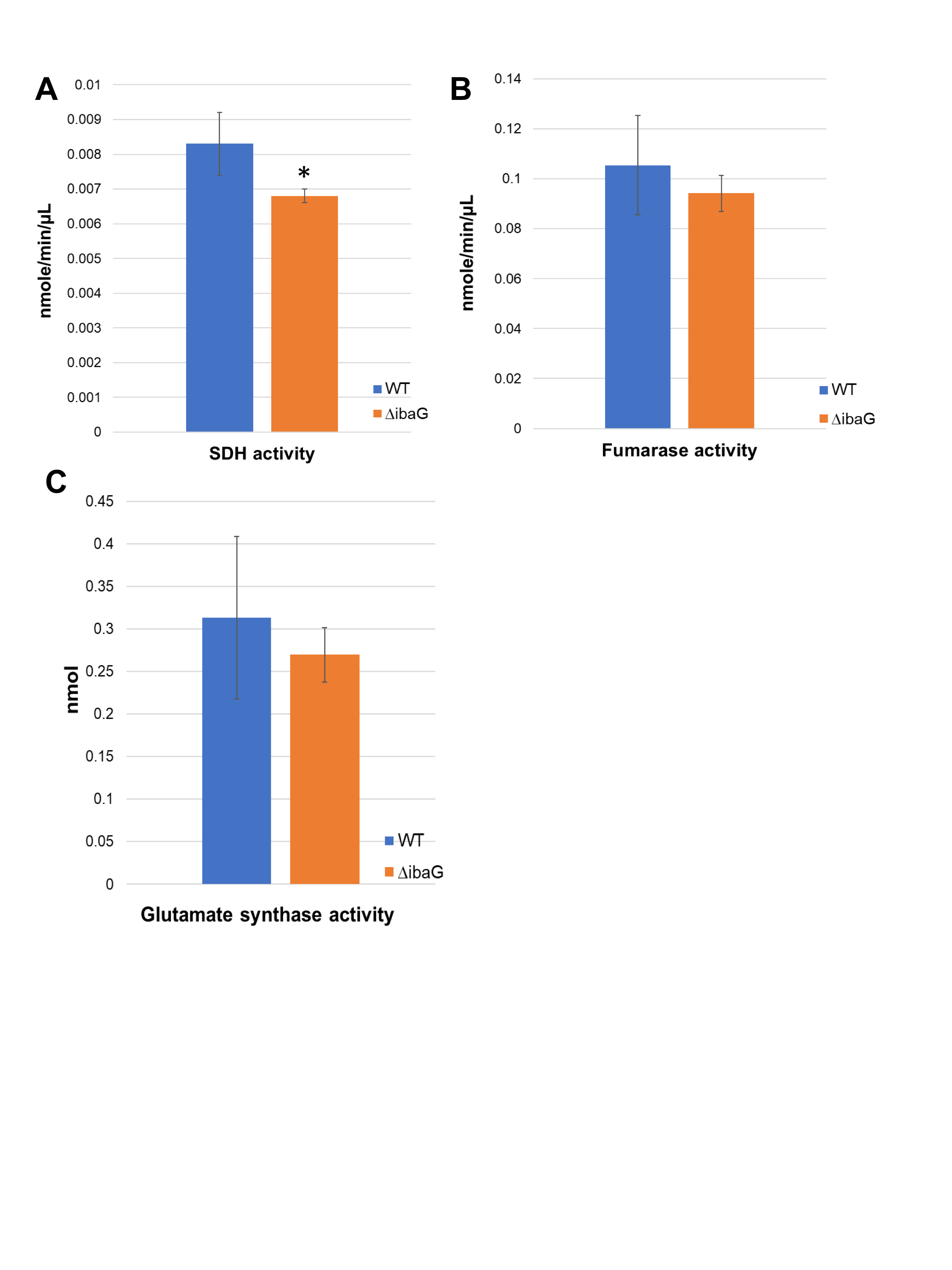

Supplement: FIG S7 [file mBio.00790-19-sf007.tif]
