# Supplementary material for: A Vibrio cholerae BolA-Like Protein Is Required for Proper Cell Shape and Cell Envelope Integrity
Source: mBio. 2019 Jul 9;10(4):e00790-19. doi: 10.1128/mBio.00790-19 (PMC6747721; doi:10.1128/mBio.00790-19)
Supplement: TABLE S1 [file mBio.00790-19-st001.pdf]

# Table S1. Transposon-insertion sequencing analysis

| | Under-represented genes in Tn-seq analysis* | | |
|---|---|---|---|
| Gene | log2 mean fold change ∆ibag/wt | inverse p-value | Predicted function |
| VC_2518 | -7.59 | 2.56E+05 | MlaD |
| VC_2517 | -7.52 | 1.89E+04 | MlaC |
| VC_2516 | -7.12 | 1.04E+03 | MlaB |
| VC_2437 | -5.72 | 5.60E+03 | pseudogene near TolC vc2436 |
| VC_0237 | -5.46 | 6.32E+03 | RfaL O-antigen ligase |
| VC_2317 | -5.13 | 3.50E+01 | RfaE |
| VC_2515 | -5.00 | 4.62E+02 | IbaG |
| VC_1613 | -5.00 | 3.50E+01 | unknown |
| VC_2436 | -4.63 | 4.12E+09 | TolC |
| VC_0761 | -4.43 | 4.42E+01 | YfgM in E. coli, periplasmic chaperone, ancillary SecYEG translocon subunit |
| VC_2670 | -4.39 | 3.50E+01 | TpiA triosephosphate isomerase |
| VC_2409 | -4.39 | 3.50E+01 | MraW S-adenosyl-methyltransferase, 16S rRNA (cytosine1402-N4)-methyltransferase |
| VC_0240 | -4.36 | 2.80E+04 | RfaD |
| VC_A0509 | -4.08 | 5.52E+01 | unknown |
| VC_A0105 | -3.76 | 1.64E+02 | unknown |
| VC_0263 | -3.71 | 6.26E+02 | galactosyl transferase |
| VC_0225 | -3.65 | 8.10E+03 | RfaQ lipopolysaccharide biosynthesis protein, heptosyl transferase I |
| VC_1839 | -3.52 | 1.84E+03 | TolQ |
| VC_2689 | -3.47 | 6.69E+02 | PfkA, 6 phosphofructokinase |
| VC_1837 | -3.35 | 8.82E+01 | TolA |
| VC_0247 | -3.30 | 2.97E+01 | YbhF, lipopolysaccharide transport system ATP-binding protein, efflux pomp |
| VC_1339 | -3.30 | 1.28E+02 | 2-methylaconitate isomerase PrpF |
| VC_0025 | -3.21 | 9.17E+01 | unknown |
| VC_0581 | -3.15 | 1.99E+07 | LpoA |
| VC_2268 | -2.85 | 3.87E+03 | RibH riboflavine metabolism |
| VC_A0821 | -2.81 | 2.38E+01 | unknown |
| VC_0958 | -2.80 | 7.06E+01 | LnT, apolipoprotein N-acyltransferase, lipoprotein synthesis, essential in E. coli |
| VC_1342 | -2.72 | 5.54E+04 | MutT/nudix family protein |
| VC_A0408 | -2.69 | 6.17E+01 | unknown |
| VC_1038 | -2.57 | 3.21E+02 | uridine kinase |
| VC_A0298 | -2.45 | 6.50E+01 | unknown |
| VC_1845 | -2.26 | 4.98E+01 | RuvB Holliday junction DNA helicase |
| VC_1838 | -2.23 | 2.59E+01 | TolR |
| VC_A0600 | -2.22 | 3.08E+01 | PhnR repressor protein |
| VC_A0866 | -2.14 | 4.65E+01 | unknown |
| VC_A0806 | -2.09 | 5.60E+01 | unknown |
| VC_A0651 | -2.06 | 2.55E+01 | unknown |
| VC_2635 | -2.06 | 8.60E+03 | PBP1a |

| | Over-represented genes in Tn-seq analysis* | | |
|---|---|---|---|
| Gene | log2 mean fold change ∆ibag/wt | inverse p-value | Predicted function |
| VC_0947 | 5.76 | 1.86E+04 | DacA1 PBP5 |
| VC_1257 | 5.73 | 3.45E+01 | 3-demethylubiquinone-9 3-methyltransferase |
| VC_A0660 | 5.15 | 3.53E+01 | unknown |
| VC_2770 | 4.89 | 2.66E+03 | ATP synthase F0F1 subunit A |
| VC_A0677 | 4.79 | 3.57E+01 | NapD periplasmic nitrate reductase |
| VC_A1022 | 4.44 | 2.50E+01 | unknown |
| VC_A0953 | 4.30 | 7.29E+01 | peptidyl-prolyl cis-trans isomerase C |
| VC_1681 | 3.83 | 4.02E+01 | SapB antimicrobial peptide ABC transporter permease |
| VC_0108 | 3.76 | 1.87E+03 | DNA polymerase 1 |
| VC_1662 | 3.69 | 2.40E+01 | unknown |
| VC_0742 | 3.53 | 5.02E+01 | YajC preprotein translocase subunit Sec translocon module |
| VC_A0752 | 3.38 | 3.36E+01 | Thioredoxin 2 |
| VC_2567 | 3.18 | 3.85E+01 | unknown membrane protein |
| VC_2765 | 3.17 | 1.49E+02 | F-type H+-transporting ATPase subunit gamma \| (RefSeq) ATP synthase F0F1 subunit gamma |
| VC_A0607 | 3.12 | 3.10E+02 | ATP synthase F0F1 subunit alpha |
| VC_1869 | 3.07 | 3.79E+02 | PflA pyruvate formate lyase-activating enzyme 1 |
| VC_0105 | 3.06 | 6.89E+01 | delta-aminolevulinic acid dehydratase |
| VC_A0838 | 2.98 | 2.37E+01 | unknown |
| VC_1290 | 2.82 | 2.02E+02 | DNA polymerase III subunit epsilon |
| VC_2565 | 2.74 | 2.63E+02 | ElaA protein putative N-acetyltransferase |
| VC_1490 | 2.69 | 2.20E+01 | cell division protein ZapC |
| VC_1689 | 2.68 | 5.29E+01 | pseudogene |
| VC_1358 | 2.65 | 2.67E+02 | YccA Modulator of FtsH protease, inner membrane protein |
| VC_A0255 | 2.56 | 2.85E+01 | unknown |
| VC_1904 | 2.46 | 3.07E+02 | leucine-responsive transcriptional regulator |
| VC_2766 | 2.46 | 4.81E+03 | ATP synthase F0F1 subunit alpha |
| VC_1355 | 2.36 | 1.17E+02 | acylphosphatase |
| VC_0731 | 2.34 | 2.00E+01 | peroxiredoxin (alkyl hydroperoxide reductase subunit C), anti-oxidant AhpCTSA family protein |
| VC_0842 | 2.22 | 2.87E+01 | unknown |
| VC_A0021 | 2.21 | 8.10E+02 | unknown |
| VC_2106 | 2.20 | 5.01E+01 | Fur ferric uptake regulator |
| VC_2724 | 2.17 | 2.25E+01 | cholera toxin secretion protein EpsM |
| VC_0549 | 2.17 | 8.14E+01 | oxaloacetate decarboxylase subunit gamma |
| VC_A0478 | 2.03 | 2.06E+01 | mRNA interferase RelE/StbE |

* list of the genes over or under-represented in the Δ*ibaG* library with a mean fold change >2 and a p-value <0.05

# Tn-seq raw data

STD : standard deviation

NaN : not applicable

| genes | mean number of informative sites | STD | reads in ∆ibag | reads in WT | Log 2 mean fold change ∆ibag/wt | STD of fold change | p-value |
|---|---|---|---|---|---|---|---|
| VC_A1115 | 0.00 | 0.00 | 1.00 | 1.00 | #VALEUR! | NaN | NaN |
| VC_A1114 | 0.59 | 0.49 | 1.28 | 1.00 | 0.28 | 0.26 | 1.000 |
| VC_A1113 | 28.35 | 0.63 | 239.55 | 140.00 | 0.77 | 0.04 | 0.352 |
| VC_A1112 | 47.15 | 0.80 | 439.70 | 312.00 | 0.49 | 0.04 | 0.270 |
| VC_A1111 | 11.28 | 0.68 | 164.72 | 153.00 | 0.10 | 0.07 | 0.632 |
| VC_A1110 | 28.14 | 1.44 | 201.82 | 150.00 | 0.42 | 0.06 | 0.161 |
| VC_A1109 | 17.67 | 0.47 | 274.74 | 284.00 | -0.05 | 0.06 | 0.843 |
| VC_A1108 | 13.61 | 0.49 | 104.51 | 222.00 | -1.10 | 0.22 | 0.379 |
| VC_A1107 | 5.53 | 0.50 | 25.49 | 28.00 | -0.20 | 0.27 | 0.691 |
| VC_A1106 | 4.85 | 0.36 | 17.67 | 19.00 | -0.19 | 0.29 | 0.748 |
| VC_A1105 | 21.66 | 0.48 | 287.00 | 413.00 | -0.53 | 0.09 | 0.367 |
| VC_A1104 | 32.44 | 0.69 | 157.51 | 299.00 | -0.93 | 0.14 | 0.124 |
| VC_A1103 | 3.95 | 0.22 | 123.04 | 69.00 | 0.82 | 0.05 | 0.899 |
| VC_A1102 | 51.38 | 1.08 | 710.87 | 441.00 | 0.69 | 0.02 | 0.183 |
| VC_A1101 | 31.86 | 0.89 | 248.05 | 195.00 | 0.34 | 0.05 | 0.414 |
| VC_A1100 | 18.64 | 0.48 | 157.20 | 171.00 | -0.13 | 0.10 | 0.806 |
| VC_A1099 | 25.79 | 0.41 | 257.44 | 434.00 | -0.76 | 0.09 | 0.086 |
| VC_A1098 | 34.10 | 0.77 | 376.02 | 450.00 | -0.26 | 0.05 | 0.910 |
| VC_A1097 | 9.99 | 0.10 | 106.29 | 133.00 | -0.34 | 0.13 | 0.320 |
| VC_A1096 | 7.66 | 0.48 | 53.31 | 29.00 | 0.85 | 0.09 | 0.603 |
| VC_A1095 | 39.28 | 0.68 | 413.56 | 285.00 | 0.53 | 0.03 | 0.662 |
| VC_A1094 | 9.70 | 0.46 | 51.13 | 64.00 | -0.36 | 0.20 | 0.814 |
| VC_A1093 | 12.91 | 0.29 | 248.30 | 236.00 | 0.07 | 0.06 | 0.687 |
| VC_A1092 | 46.16 | 0.69 | 443.90 | 596.00 | -0.43 | 0.06 | 0.240 |
| VC_A1091 | 15.95 | 0.22 | 193.03 | 266.00 | -0.47 | 0.10 | 0.356 |
| VC_A1090 | 19.27 | 0.68 | 193.46 | 205.00 | -0.09 | 0.08 | 0.784 |
| VC_A1089 | 25.14 | 0.68 | 242.98 | 440.00 | -0.86 | 0.11 | 0.168 |
| VC_A1088 | 22.61 | 0.49 | 141.05 | 190.00 | -0.44 | 0.11 | 0.169 |
| VC_A1087 | 10.00 | 0.00 | 118.16 | 146.00 | -0.32 | 0.12 | 0.810 |
| VC_A1086 | 42.66 | 1.22 | 371.72 | 464.00 | -0.32 | 0.07 | 0.587 |
| VC_A1085 | 38.83 | 1.09 | 306.25 | 384.00 | -0.33 | 0.07 | 0.538 |
| VC_A1084 | 51.92 | 0.87 | 579.24 | 517.00 | 0.16 | 0.04 | 0.878 |

| | | | | | | | |
|---|---|---|---|---|---|---|---|
| VC_A1083 | 21.92 | 0.27 | 160.65 | 292.00 | -0.87 | 0.15 | 0.169 |
| VC_A1082 | 31.23 | 0.68 | 457.32 | 341.00 | 0.42 | 0.03 | 0.247 |
| VC_A1081 | 20.04 | 0.85 | 258.08 | 173.00 | 0.57 | 0.05 | 0.711 |
| VC_A1080 | 33.51 | 0.90 | 232.18 | 281.00 | -0.28 | 0.09 | 0.875 |
| VC_A1079 | 0.00 | 0.00 | 1.00 | 1.00 | #VALEUR! | NaN | NaN |
| VC_A1078 | 20.29 | 0.78 | 127.85 | 102.00 | 0.31 | 0.08 | 0.755 |
| VC_A1077 | 41.13 | 1.11 | 610.40 | 292.00 | 1.06 | 0.02 | 0.004 |
| VC_A1076 | 2.00 | 0.00 | 9.59 | 1.00 | 2.98 | 0.10 | 1.000 |
| VC_A1075 | 29.54 | 1.01 | 563.55 | 367.00 | 0.62 | 0.03 | 0.587 |
| VC_A1074 | 22.00 | 0.00 | 439.94 | 355.00 | 0.31 | 0.04 | 0.685 |
| VC_A1073 | 56.46 | 1.01 | 483.31 | 640.00 | -0.41 | 0.06 | 0.874 |
| VC_A1072 | 16.00 | 0.00 | 341.38 | 315.00 | 0.11 | 0.05 | 0.490 |
| VC_A1071 | 30.23 | 1.11 | 145.35 | 191.00 | -0.40 | 0.12 | 0.850 |
| VC_A1070 | 6.00 | 0.00 | 129.82 | 44.00 | 1.55 | 0.03 | 0.281 |
| VC_A1069 | 33.14 | 0.73 | 327.12 | 324.00 | 0.01 | 0.06 | 0.397 |
| VC_A1068 | 8.17 | 0.71 | 63.09 | 18.00 | 1.79 | 0.04 | 0.264 |
| VC_A1067 | 25.52 | 1.03 | 404.80 | 175.00 | 1.21 | 0.02 | 0.046 |
| VC_A1066 | 9.50 | 0.50 | 78.22 | 80.00 | -0.05 | 0.12 | 0.645 |
| VC_A1065 | 3.00 | 0.00 | 10.92 | 8.00 | 0.28 | 0.40 | 0.991 |
| VC_A1064 | 1.69 | 0.46 | 1.53 | 4.00 | -1.69 | 1.10 | 1.000 |
| VC_A1063 | 52.81 | 0.93 | 526.57 | 647.00 | -0.30 | 0.06 | 0.346 |
| VC_A1062 | 32.00 | 0.00 | 379.56 | 412.00 | -0.12 | 0.06 | 0.866 |
| VC_A1061 | 3.72 | 0.45 | 5.17 | 20.00 | -2.25 | 2.86 | 0.764 |
| VC_A1060 | 18.28 | 0.70 | 193.15 | 150.00 | 0.36 | 0.06 | 0.117 |
| VC_A1059 | 8.00 | 0.00 | 67.68 | 130.00 | -0.96 | 0.25 | 0.118 |
| VC_A1058 | 22.32 | 0.74 | 207.11 | 165.00 | 0.32 | 0.05 | 0.827 |
| VC_A1057 | 14.61 | 0.51 | 126.71 | 186.00 | -0.57 | 0.14 | 0.461 |
| VC_A1056 | 62.75 | 1.10 | 570.22 | 764.00 | -0.42 | 0.06 | 0.125 |
| VC_A1055 | 23.25 | 0.70 | 281.21 | 208.00 | 0.43 | 0.05 | 0.339 |
| VC_A1054 | 14.45 | 0.66 | 160.74 | 207.00 | -0.38 | 0.12 | 0.442 |
| VC_A1053 | 12.00 | 0.00 | 91.27 | 140.00 | -0.64 | 0.19 | 0.331 |
| VC_A1052 | 13.00 | 0.00 | 253.70 | 88.00 | 1.52 | 0.02 | 0.571 |
| VC_A1051 | 9.63 | 0.53 | 73.21 | 112.00 | -0.63 | 0.15 | 0.686 |
| VC_A1050 | 23.50 | 0.59 | 120.89 | 196.00 | -0.71 | 0.17 | 0.685 |
| VC_A1049 | 25.64 | 1.07 | 311.65 | 166.00 | 0.90 | 0.03 | 0.257 |
| VC_A1048 | 26.79 | 0.43 | 437.42 | 301.00 | 0.54 | 0.04 | 0.298 |
| VC_A1047 | 7.00 | 0.00 | 77.03 | 39.00 | 0.96 | 0.06 | 0.803 |
| VC_A1046 | 19.25 | 0.76 | 257.91 | 307.00 | -0.26 | 0.08 | 0.777 |
| VC_A1045 | 57.27 | 1.14 | 756.53 | 872.00 | -0.21 | 0.04 | 0.898 |
| VC_A1044 | 2.00 | 0.00 | 4.19 | 1.00 | 1.63 | 0.22 | 1.000 |

| | | | | | | | |
|---|---|---|---|---|---|---|---|
| VC_A1043 | 35.61 | 0.94 | 238.32 | 239.00 | -0.01 | 0.07 | 0.817 |
| VC_A1042 | 12.61 | 0.51 | 46.69 | 53.00 | -0.22 | 0.18 | 0.723 |
| VC_A1041 | 30.29 | 0.73 | 282.89 | 266.00 | 0.08 | 0.06 | 0.594 |
| VC_A1040 | 31.62 | 0.58 | 201.99 | 395.00 | -0.98 | 0.14 | 0.146 |
| VC_A1039 | 12.00 | 0.00 | 117.56 | 192.00 | -0.72 | 0.16 | 0.186 |
| VC_A1038 | 21.62 | 0.49 | 490.11 | 248.00 | 0.98 | 0.02 | 0.472 |
| VC_A1037 | 12.14 | 0.70 | 80.66 | 200.00 | -1.33 | 0.28 | 0.271 |
| VC_A1036 | 26.09 | 0.79 | 169.35 | 354.00 | -1.07 | 0.17 | 0.161 |
| VC_A1035 | 15.82 | 0.39 | 248.02 | 95.00 | 1.38 | 0.03 | 0.008 |
| VC_A1034 | 44.80 | 0.40 | 389.81 | 546.00 | -0.49 | 0.07 | 0.830 |
| VC_A1033 | 32.26 | 0.75 | 385.72 | 357.00 | 0.11 | 0.05 | 0.671 |
| VC_A1032 | 9.95 | 0.22 | 99.38 | 88.00 | 0.16 | 0.11 | 0.476 |
| VC_A1031 | 33.23 | 0.75 | 506.64 | 468.00 | 0.11 | 0.04 | 0.624 |
| VC_A1030 | 4.91 | 0.29 | 35.88 | 49.00 | -0.49 | 0.26 | 0.925 |
| VC_A1029 | 61.35 | 0.70 | 825.22 | 567.00 | 0.54 | 0.02 | 0.460 |
| VC_A1028 | 55.22 | 1.07 | 515.04 | 608.00 | -0.24 | 0.05 | 0.581 |
| VC_A1027 | 32.56 | 0.86 | 336.15 | 375.00 | -0.16 | 0.06 | 0.412 |
| VC_A1026 | 29.79 | 0.46 | 451.93 | 219.00 | 1.04 | 0.02 | 0.205 |
| VC_A1025 | 22.52 | 0.59 | 291.16 | 264.00 | 0.14 | 0.05 | 0.557 |
| VC_A1024 | 10.00 | 0.00 | 75.09 | 130.00 | -0.82 | 0.23 | 0.467 |
| VC_A1023 | 57.87 | 0.85 | 583.24 | 378.00 | 0.62 | 0.03 | 0.049 |
| VC_A1022 | 5.62 | 0.49 | 44.78 | 2.00 | 4.44 | 0.01 | 0.040 |
| VC_A1021 | 8.65 | 0.50 | 52.04 | 102.00 | -1.00 | 0.31 | 0.602 |
| VC_A1020 | 18.61 | 0.57 | 200.52 | 154.00 | 0.37 | 0.06 | 0.798 |
| VC_A1019 | 13.94 | 0.24 | 110.28 | 117.00 | -0.10 | 0.11 | 0.237 |
| VC_A1018 | 29.21 | 0.74 | 329.87 | 179.00 | 0.88 | 0.03 | 0.065 |
| VC_A1017 | 14.84 | 0.37 | 56.66 | 118.00 | -1.09 | 0.31 | 0.556 |
| VC_A1016 | 11.87 | 0.34 | 148.35 | 139.00 | 0.08 | 0.08 | 0.853 |
| VC_A1015 | 36.18 | 0.99 | 480.17 | 538.00 | -0.17 | 0.06 | 0.900 |
| VC_A1014 | 1.00 | 0.00 | 1.00 | 1.00 | 0.00 | 0.00 | 1.000 |
| VC_A1013 | 21.00 | 0.00 | 323.68 | 428.00 | -0.41 | 0.07 | 0.828 |
| VC_A1012 | 17.87 | 0.34 | 72.19 | 160.00 | -1.17 | 0.28 | 0.125 |
| VC_A1011 | 23.86 | 0.40 | 108.51 | 218.00 | -1.02 | 0.19 | 0.052 |
| VC_A1010 | 17.67 | 0.47 | 331.99 | 147.00 | 1.17 | 0.02 | 0.363 |
| VC_A1009 | 2.66 | 0.48 | 11.55 | 21.00 | -0.99 | 0.62 | 0.762 |
| VC_A1008 | 22.02 | 0.79 | 218.61 | 272.00 | -0.32 | 0.10 | 0.663 |
| VC_A1007 | 3.00 | 0.00 | 55.44 | 24.00 | 1.18 | 0.06 | 0.503 |
| VC_A1006 | 6.72 | 0.45 | 39.96 | 88.00 | -1.17 | 0.36 | 0.529 |
| VC_A1005 | 8.99 | 0.10 | 59.56 | 84.00 | -0.52 | 0.20 | 0.910 |
| VC_A1004 | 12.86 | 0.35 | 189.31 | 147.00 | 0.36 | 0.06 | 0.255 |

| | | | | | | | |
|---|---|---|---|---|---|---|---|
| VC_A1003 | 8.93 | 0.26 | 112.43 | 218.00 | -0.97 | 0.19 | 0.332 |
| VC_A1002 | 21.20 | 0.83 | 140.98 | 239.00 | -0.77 | 0.15 | 0.688 |
| VC_A1001 | 23.77 | 0.45 | 157.10 | 156.00 | 0.00 | 0.08 | 0.718 |
| VC_A1000 | 13.80 | 0.85 | 74.39 | 179.00 | -1.29 | 0.31 | 0.729 |
| VC_A0999 | 12.95 | 0.77 | 28.98 | 58.00 | -1.04 | 0.36 | 0.167 |
| VC_A0998 | 21.25 | 0.77 | 201.10 | 124.00 | 0.69 | 0.05 | 0.562 |
| VC_A0997 | 9.00 | 0.00 | 78.53 | 72.00 | 0.11 | 0.11 | 0.692 |
| VC_A0996 | 40.04 | 0.78 | 375.98 | 544.00 | -0.54 | 0.07 | 0.290 |
| VC_A0995 | 2.00 | 0.00 | 27.16 | 24.00 | 0.12 | 0.21 | 0.957 |
| VC_A0994 | 25.89 | 0.99 | 171.62 | 95.00 | 0.85 | 0.04 | 0.054 |
| VC_A0993 | 24.11 | 0.74 | 205.03 | 300.00 | -0.56 | 0.10 | 0.515 |
| VC_A0992 | 6.89 | 0.31 | 14.30 | 56.00 | -2.11 | 1.63 | 0.152 |
| VC_A0991 | 15.36 | 0.64 | 102.44 | 84.00 | 0.27 | 0.09 | 0.427 |
| VC_A0990 | 21.82 | 0.89 | 315.65 | 124.00 | 1.34 | 0.02 | 0.423 |
| VC_A0989 | 38.64 | 0.52 | 367.11 | 671.00 | -0.87 | 0.10 | 0.101 |
| VC_A0988 | 36.63 | 0.63 | 402.67 | 375.00 | 0.10 | 0.05 | 0.876 |
| VC_A0987 | 46.34 | 0.71 | 982.03 | 1077.00 | -0.13 | 0.03 | 0.580 |
| VC_A0986 | 16.38 | 0.65 | 234.03 | 116.00 | 1.01 | 0.03 | 0.880 |
| VC_A0985 | 47.56 | 1.29 | 608.72 | 269.00 | 1.18 | 0.02 | 0.010 |
| VC_A0984 | 18.92 | 0.27 | 266.92 | 207.00 | 0.36 | 0.05 | 0.810 |
| VC_A0983 | 26.58 | 0.55 | 356.12 | 339.00 | 0.07 | 0.04 | 0.562 |
| VC_A0982 | 17.67 | 0.47 | 123.29 | 244.00 | -1.00 | 0.18 | 0.139 |
| VC_A0981 | 23.05 | 0.91 | 193.22 | 184.00 | 0.06 | 0.07 | 0.773 |
| VC_A0980 | 23.00 | 0.00 | 109.79 | 157.00 | -0.53 | 0.14 | 0.271 |
| VC_A0979 | 13.55 | 1.01 | 162.99 | 59.00 | 1.46 | 0.03 | 0.285 |
| VC_A0978 | 15.00 | 0.00 | 65.93 | 172.00 | -1.40 | 0.33 | 0.119 |
| VC_A0977 | 27.33 | 0.71 | 303.96 | 121.00 | 1.32 | 0.03 | 0.440 |
| VC_A0976 | 15.95 | 0.22 | 113.81 | 232.00 | -1.04 | 0.19 | 0.236 |
| VC_A0975 | 50.52 | 0.58 | 650.31 | 719.00 | -0.15 | 0.04 | 0.534 |
| VC_A0974 | 30.38 | 0.68 | 400.47 | 297.00 | 0.43 | 0.04 | 0.588 |
| VC_A0973 | 0.00 | 0.00 | 1.00 | 1.00 | #VALEUR! | NaN | NaN |
| VC_A0972 | 32.72 | 0.51 | 182.21 | 325.00 | -0.84 | 0.14 | 0.283 |
| VC_A0971 | 10.60 | 0.49 | 103.78 | 136.00 | -0.41 | 0.15 | 0.257 |
| VC_A0970 | 5.90 | 0.30 | 66.28 | 37.00 | 0.82 | 0.07 | 0.777 |
| VC_A0969 | 13.52 | 0.59 | 322.98 | 142.00 | 1.18 | 0.02 | 0.422 |
| VC_A0968 | 2.59 | 0.51 | 7.51 | 1.00 | 2.73 | 0.05 | 0.200 |
| VC_A0967 | 1.00 | 0.00 | 8.49 | 1.00 | 2.86 | 0.06 | 1.000 |
| VC_A0966 | 12.00 | 0.00 | 222.83 | 117.00 | 0.92 | 0.04 | 0.890 |
| VC_A0965 | 44.65 | 0.54 | 365.21 | 381.00 | -0.07 | 0.06 | 0.545 |
| VC_A0964 | 18.92 | 0.27 | 141.92 | 206.00 | -0.55 | 0.14 | 0.400 |

| | | | | | | | |
|---|---|---|---|---|---|---|---|
| VC_A0963 | 30.50 | 0.94 | 176.75 | 245.00 | -0.48 | 0.10 | 0.555 |
| VC_A0962 | 31.96 | 0.20 | 362.68 | 502.00 | -0.47 | 0.08 | 0.192 |
| VC_A0961 | 13.70 | 0.48 | 54.94 | 73.00 | -0.44 | 0.19 | 0.445 |
| VC_A0960 | 56.46 | 0.66 | 524.67 | 559.00 | -0.10 | 0.06 | 0.750 |
| VC_A0959 | 0.00 | 0.00 | 1.00 | 1.00 | #VALEUR! | NaN | NaN |
| VC_A0958 | 27.97 | 0.83 | 127.56 | 256.00 | -1.02 | 0.18 | 0.793 |
| VC_A0957 | 18.27 | 0.75 | 239.03 | 167.00 | 0.51 | 0.04 | 0.744 |
| VC_A0956 | 27.38 | 0.68 | 118.57 | 302.00 | -1.36 | 0.24 | 0.049 |
| VC_A0955 | 17.20 | 0.72 | 270.10 | 248.00 | 0.12 | 0.06 | 0.857 |
| VC_A0954 | 20.81 | 0.46 | 160.26 | 164.00 | -0.04 | 0.08 | 0.350 |
| VC_A0953 | 5.23 | 0.68 | 40.51 | 2.00 | 4.30 | 0.01 | 0.014 |
| VC_A0952 | 21.19 | 0.72 | 96.14 | 85.00 | 0.16 | 0.10 | 0.807 |
| VC_A0951 | 5.00 | 0.00 | 119.62 | 131.00 | -0.14 | 0.11 | 0.807 |
| VC_A0950 | 2.00 | 0.00 | 24.44 | 15.00 | 0.63 | 0.15 | 1.000 |
| VC_A0949 | 14.62 | 0.55 | 37.35 | 72.00 | -0.99 | 0.35 | 0.591 |
| VC_A0948 | 8.54 | 0.61 | 149.30 | 33.00 | 2.17 | 0.02 | 0.501 |
| VC_A0947 | 20.60 | 0.62 | 274.19 | 119.00 | 1.20 | 0.02 | 0.022 |
| VC_A0946 | 21.38 | 0.68 | 395.44 | 339.00 | 0.22 | 0.04 | 0.123 |
| VC_A0945 | 25.70 | 0.46 | 283.22 | 286.00 | -0.02 | 0.06 | 0.628 |
| VC_A0944 | 37.59 | 0.51 | 455.46 | 384.00 | 0.24 | 0.04 | 0.391 |
| VC_A0943 | 26.35 | 0.66 | 348.83 | 197.00 | 0.82 | 0.03 | 0.201 |
| VC_A0942 | 13.79 | 0.43 | 78.54 | 111.00 | -0.52 | 0.15 | 0.211 |
| VC_A0941 | 11.67 | 0.47 | 87.75 | 79.00 | 0.14 | 0.10 | 0.908 |
| VC_A0940 | 12.30 | 0.72 | 127.80 | 74.00 | 0.78 | 0.05 | 0.875 |
| VC_A0939 | 22.61 | 0.57 | 164.47 | 266.00 | -0.70 | 0.13 | 0.752 |
| VC_A0938 | 22.97 | 0.17 | 268.92 | 507.00 | -0.92 | 0.11 | 0.729 |
| VC_A0937 | 15.83 | 0.38 | 102.03 | 121.00 | -0.26 | 0.12 | 0.622 |
| VC_A0936 | 24.49 | 0.64 | 279.42 | 411.00 | -0.56 | 0.10 | 0.749 |
| VC_A0935 | 9.00 | 0.00 | 49.35 | 24.00 | 1.01 | 0.07 | 0.934 |
| VC_A0934 | 0.00 | 0.00 | 1.00 | 1.00 | #VALEUR! | NaN | NaN |
| VC_A0933 | 6.64 | 0.50 | 45.42 | 7.00 | 2.66 | 0.03 | 0.135 |
| VC_A0932 | 4.00 | 0.00 | 2.27 | 21.00 | -3.66 | 6.68 | 0.090 |
| VC_A0931 | 38.51 | 0.56 | 300.90 | 447.00 | -0.58 | 0.08 | 0.069 |
| VC_A0930 | 20.67 | 0.47 | 232.75 | 385.00 | -0.73 | 0.11 | 0.637 |
| VC_A0929 | 21.59 | 0.55 | 194.16 | 164.00 | 0.24 | 0.06 | 0.758 |
| VC_A0928 | 9.32 | 0.68 | 64.57 | 70.00 | -0.14 | 0.14 | 0.798 |
| VC_A0927 | 8.98 | 0.14 | 38.79 | 76.00 | -1.00 | 0.30 | 0.564 |
| VC_A0926 | 18.93 | 0.82 | 259.39 | 162.00 | 0.67 | 0.03 | 0.766 |
| VC_A0925 | 15.51 | 0.56 | 182.69 | 196.00 | -0.11 | 0.07 | 0.643 |
| VC_A0924 | 43.70 | 0.52 | 510.86 | 263.00 | 0.95 | 0.03 | 0.009 |

| | | | | | | | |
|---|---|---|---|---|---|---|---|
| VC_A0923 | 46.16 | 0.84 | 382.17 | 416.00 | -0.13 | 0.06 | 0.487 |
| VC_A0922 | 14.69 | 0.53 | 111.73 | 184.00 | -0.73 | 0.14 | 0.506 |
| VC_A0921 | 5.00 | 0.00 | 127.89 | 37.00 | 1.78 | 0.02 | 0.745 |
| VC_A0920 | 11.90 | 0.30 | 154.09 | 122.00 | 0.33 | 0.06 | 0.933 |
| VC_A0919 | 8.00 | 0.00 | 84.72 | 78.00 | 0.10 | 0.10 | 0.886 |
| VC_A0918 | 5.54 | 0.56 | 48.19 | 54.00 | -0.20 | 0.19 | 0.479 |
| VC_A0917 | 5.00 | 0.00 | 22.65 | 15.00 | 0.52 | 0.18 | 0.783 |
| VC_A0916 | 2.00 | 0.00 | 25.36 | 4.00 | 2.60 | 0.04 | 1.000 |
| VC_A0915 | 13.98 | 0.14 | 288.76 | 176.00 | 0.71 | 0.03 | 0.876 |
| VC_A0914 | 29.58 | 0.50 | 226.35 | 370.00 | -0.71 | 0.10 | 0.831 |
| VC_A0913 | 18.37 | 0.69 | 261.15 | 83.00 | 1.65 | 0.02 | 0.048 |
| VC_A0912 | 8.64 | 0.48 | 35.55 | 30.00 | 0.21 | 0.14 | 0.839 |
| VC_A0911 | 16.95 | 0.22 | 300.26 | 231.00 | 0.37 | 0.04 | 0.857 |
| VC_A0910 | 16.32 | 0.75 | 158.77 | 225.00 | -0.51 | 0.12 | 0.792 |
| VC_A0909 | 23.32 | 0.69 | 130.01 | 479.00 | -1.89 | 0.35 | 0.030 |
| VC_A0908 | 16.74 | 0.44 | 198.32 | 149.00 | 0.41 | 0.05 | 0.749 |
| VC_A0907 | 8.30 | 0.66 | 57.19 | 17.00 | 1.72 | 0.04 | 0.592 |
| VC_A0906 | 50.13 | 0.75 | 741.84 | 605.00 | 0.29 | 0.03 | 0.223 |
| VC_A0905 | 17.58 | 0.57 | 309.08 | 193.00 | 0.67 | 0.04 | 0.566 |
| VC_A0904 | 45.32 | 1.22 | 595.80 | 384.00 | 0.63 | 0.03 | 0.075 |
| VC_A0903 | 19.85 | 0.87 | 181.35 | 87.00 | 1.05 | 0.04 | 0.402 |
| VC_A0902 | 21.92 | 0.82 | 97.74 | 141.00 | -0.55 | 0.17 | 0.843 |
| VC_A0901 | 15.62 | 0.49 | 392.70 | 146.00 | 1.42 | 0.02 | 0.162 |
| VC_A0900 | 19.78 | 0.46 | 278.83 | 197.00 | 0.49 | 0.05 | 0.137 |
| VC_A0899 | 3.00 | 0.00 | 15.41 | 41.00 | -1.54 | 1.08 | 0.363 |
| VC_A0898 | 28.85 | 0.91 | 304.61 | 274.00 | 0.15 | 0.05 | 0.336 |
| VC_A0897 | 12.63 | 0.51 | 125.51 | 152.00 | -0.29 | 0.13 | 0.765 |
| VC_A0896 | 36.31 | 0.72 | 386.34 | 510.00 | -0.41 | 0.08 | 0.745 |
| VC_A0895 | 74.98 | 0.88 | 894.92 | 853.00 | 0.07 | 0.03 | 0.668 |
| VC_A0894 | 10.58 | 0.50 | 51.31 | 132.00 | -1.39 | 0.39 | 0.700 |
| VC_A0893 | 8.92 | 0.27 | 42.40 | 29.00 | 0.51 | 0.12 | 0.728 |
| VC_A0892 | 15.00 | 0.00 | 124.24 | 112.00 | 0.14 | 0.08 | 0.686 |
| VC_A0891 | 34.43 | 0.67 | 351.79 | 363.00 | -0.05 | 0.05 | 0.429 |
| VC_A0890 | 12.88 | 0.33 | 113.28 | 87.00 | 0.37 | 0.07 | 0.919 |
| VC_A0889 | 16.98 | 0.14 | 66.13 | 204.00 | -1.64 | 0.35 | 0.038 |
| VC_A0888 | 25.82 | 0.44 | 244.33 | 164.00 | 0.57 | 0.05 | 0.227 |
| VC_A0887 | 20.59 | 0.49 | 172.67 | 136.00 | 0.33 | 0.07 | 0.288 |
| VC_A0886 | 18.50 | 0.58 | 200.62 | 241.00 | -0.27 | 0.09 | 0.619 |
| VC_A0885 | 17.78 | 0.44 | 160.80 | 221.00 | -0.47 | 0.11 | 0.174 |
| VC_A0884 | 37.03 | 1.31 | 363.43 | 208.00 | 0.80 | 0.03 | 0.408 |

| | | | | | | | |
|---|---|---|---|---|---|---|---|
| VC_A0883 | 34.02 | 0.85 | 372.52 | 333.00 | 0.16 | 0.04 | 0.447 |
| VC_A0882 | 40.79 | 0.84 | 451.50 | 458.00 | -0.02 | 0.06 | 0.799 |
| VC_A0881 | 11.67 | 0.47 | 53.07 | 58.00 | -0.15 | 0.15 | 0.595 |
| VC_A0880 | 9.00 | 0.00 | 86.97 | 140.00 | -0.70 | 0.17 | 0.046 |
| VC_A0879 | 0.00 | 0.00 | 1.00 | 1.00 | #VALEUR! | NaN | NaN |
| VC_A0878 | 2.69 | 0.46 | 34.34 | 201.00 | -2.58 | 0.95 | 0.448 |
| VC_A0877 | 26.61 | 0.57 | 291.31 | 408.00 | -0.49 | 0.08 | 0.787 |
| VC_A0876 | 31.43 | 0.66 | 114.90 | 252.00 | -1.15 | 0.21 | 0.158 |
| VC_A0875 | 31.03 | 0.89 | 305.88 | 173.00 | 0.82 | 0.03 | 0.069 |
| VC_A0874 | 1.53 | 0.56 | 3.18 | 1.00 | 1.26 | 0.26 | 0.615 |
| VC_A0873 | 37.05 | 1.09 | 243.00 | 279.00 | -0.21 | 0.08 | 0.734 |
| VC_A0872 | 51.72 | 0.47 | 442.81 | 491.00 | -0.15 | 0.06 | 0.527 |
| VC_A0871 | 44.13 | 0.82 | 325.91 | 319.00 | 0.03 | 0.05 | 0.389 |
| VC_A0870 | 17.89 | 0.31 | 239.01 | 123.00 | 0.95 | 0.03 | 0.441 |
| VC_A0869 | 2.93 | 0.26 | 20.66 | 21.00 | -0.09 | 0.23 | 0.931 |
| VC_A0868 | 4.00 | 0.00 | 60.61 | 47.00 | 0.34 | 0.10 | 0.941 |
| VC_A0867 | 32.79 | 0.41 | 318.75 | 159.00 | 1.00 | 0.03 | 0.038 |
| VC_A0866 | 6.00 | 0.00 | 22.01 | 92.00 | -2.14 | 1.11 | 0.022 |
| VC_A0865 | 56.65 | 1.05 | 1240.95 | 847.00 | 0.55 | 0.02 | 0.759 |
| VC_A0864 | 44.60 | 0.55 | 321.27 | 543.00 | -0.76 | 0.08 | 0.357 |
| VC_A0863 | 71.67 | 1.25 | 856.56 | 872.00 | -0.03 | 0.04 | 0.478 |
| VC_A0862 | 22.00 | 0.84 | 334.01 | 188.00 | 0.82 | 0.03 | 0.021 |
| VC_A0861 | 4.00 | 0.00 | 49.28 | 62.00 | -0.37 | 0.20 | 0.775 |
| VC_A0860 | 41.98 | 0.84 | 579.66 | 429.00 | 0.43 | 0.03 | 0.023 |
| VC_A0859 | 13.99 | 0.10 | 367.27 | 194.00 | 0.92 | 0.03 | 0.218 |
| VC_A0858 | 5.57 | 0.50 | 38.93 | 17.00 | 1.14 | 0.09 | 0.226 |
| VC_A0857 | 3.00 | 0.00 | 24.56 | 5.00 | 2.22 | 0.06 | 0.145 |
| VC_A0856 | 21.64 | 0.52 | 142.75 | 354.00 | -1.32 | 0.20 | 0.177 |
| VC_A0855 | 15.62 | 0.53 | 159.65 | 177.00 | -0.16 | 0.09 | 0.888 |
| VC_A0854 | 26.24 | 0.73 | 189.85 | 353.00 | -0.90 | 0.14 | 0.110 |
| VC_A0853 | 15.47 | 0.58 | 152.68 | 172.00 | -0.19 | 0.11 | 0.399 |
| VC_A0852 | 24.81 | 0.42 | 149.07 | 206.00 | -0.47 | 0.11 | 0.313 |
| VC_A0851 | 33.03 | 0.77 | 197.04 | 250.00 | -0.35 | 0.09 | 0.038 |
| VC_A0850 | 16.72 | 0.45 | 82.72 | 209.00 | -1.36 | 0.30 | 0.295 |
| VC_A0849 | 206.37 | 1.76 | 2454.28 | 2056.00 | 0.25 | 0.02 | 0.169 |
| VC_A0848 | 22.10 | 0.75 | 252.59 | 206.00 | 0.29 | 0.05 | 0.522 |
| VC_A0847 | 31.44 | 0.62 | 154.10 | 302.00 | -0.98 | 0.16 | 0.404 |
| VC_A0846 | 17.66 | 0.54 | 133.03 | 96.00 | 0.46 | 0.07 | 0.565 |
| VC_A0845 | 6.94 | 0.24 | 99.51 | 39.00 | 1.34 | 0.04 | 0.206 |
| VC_A0844 | 2.00 | 0.00 | 1.00 | 2.00 | -1.00 | 0.00 | 0.333 |

| | | | | | | | |
|---|---|---|---|---|---|---|---|
| VC_A0843 | 38.59 | 0.95 | 323.11 | 279.00 | 0.21 | 0.05 | 0.881 |
| VC_A0842 | 5.00 | 0.00 | 20.82 | 54.00 | -1.47 | 0.76 | 0.491 |
| VC_A0841 | 4.00 | 0.00 | 80.60 | 43.00 | 0.89 | 0.06 | 0.595 |
| VC_A0840 | 8.92 | 0.27 | 52.15 | 70.00 | -0.45 | 0.20 | 0.142 |
| VC_A0839 | 3.00 | 0.00 | 27.35 | 77.00 | -1.53 | 0.52 | 0.411 |
| VC_A0838 | 6.67 | 0.49 | 95.72 | 12.00 | 2.98 | 0.01 | 0.042 |
| VC_A0837 | 30.77 | 0.93 | 285.77 | 273.00 | 0.06 | 0.06 | 0.095 |
| VC_A0836 | 7.84 | 0.37 | 21.22 | 133.00 | -2.72 | 1.63 | 0.353 |
| VC_A0835 | 21.14 | 0.77 | 245.86 | 335.00 | -0.45 | 0.08 | 0.869 |
| VC_A0834 | 22.56 | 0.54 | 142.50 | 240.00 | -0.76 | 0.15 | 0.114 |
| VC_A0833 | 21.69 | 0.49 | 251.75 | 305.00 | -0.28 | 0.07 | 0.301 |
| VC_A0832 | 12.82 | 0.39 | 73.99 | 143.00 | -0.97 | 0.25 | 0.123 |
| VC_A0831 | 2.00 | 0.00 | 10.20 | 2.00 | 2.23 | 0.07 | 0.383 |
| VC_A0830 | 28.38 | 0.69 | 162.22 | 370.00 | -1.20 | 0.21 | 0.735 |
| VC_A0829 | 40.56 | 0.56 | 502.41 | 332.00 | 0.59 | 0.03 | 0.245 |
| VC_A0828 | 24.37 | 0.66 | 215.64 | 358.00 | -0.74 | 0.12 | 0.686 |
| VC_A0827 | 7.77 | 0.45 | 41.41 | 75.00 | -0.89 | 0.31 | 0.754 |
| VC_A0826 | 1.00 | 0.00 | 2.38 | 9.00 | -2.40 | 2.82 | 1.000 |
| VC_A0825 | 9.67 | 0.47 | 90.27 | 66.00 | 0.43 | 0.09 | 0.710 |
| VC_A0824 | 27.91 | 0.29 | 272.26 | 317.00 | -0.22 | 0.06 | 0.636 |
| VC_A0823 | 13.20 | 0.85 | 195.47 | 182.00 | 0.10 | 0.07 | 0.836 |
| VC_A0822 | 30.38 | 0.74 | 307.45 | 200.00 | 0.62 | 0.04 | 0.087 |
| VC_A0821 | 6.00 | 0.00 | 21.85 | 144.00 | -2.81 | 1.92 | 0.042 |
| VC_A0820 | 18.31 | 0.65 | 190.71 | 182.00 | 0.06 | 0.07 | 0.557 |
| VC_A0819 | 4.60 | 0.51 | 83.38 | 8.00 | 3.36 | 0.01 | 0.307 |
| VC_A0818 | 31.84 | 0.39 | 451.07 | 281.00 | 0.68 | 0.03 | 0.297 |
| VC_A0817 | 20.97 | 0.17 | 311.83 | 141.00 | 1.14 | 0.03 | 0.179 |
| VC_A0816 | 1.55 | 0.50 | 1.29 | 6.00 | -2.39 | 1.35 | 1.000 |
| VC_A0815 | 45.23 | 0.74 | 570.75 | 461.00 | 0.31 | 0.03 | 0.516 |
| VC_A0814 | 17.69 | 0.46 | 291.22 | 376.00 | -0.37 | 0.08 | 0.921 |
| VC_A0813 | 39.73 | 0.91 | 374.72 | 425.00 | -0.19 | 0.07 | 0.430 |
| VC_A0812 | 34.90 | 0.33 | 288.44 | 461.00 | -0.68 | 0.09 | 0.258 |
| VC_A0811 | 33.16 | 0.77 | 471.29 | 342.00 | 0.46 | 0.04 | 0.440 |
| VC_A0810 | 4.00 | 0.00 | 13.99 | 40.00 | -1.64 | 1.03 | 0.335 |
| VC_A0809 | 40.13 | 0.75 | 562.62 | 540.00 | 0.06 | 0.04 | 0.904 |
| VC_A0808 | 10.88 | 0.33 | 43.15 | 144.00 | -1.77 | 0.54 | 0.564 |
| VC_A0807 | 24.44 | 0.67 | 190.86 | 256.00 | -0.43 | 0.09 | 0.605 |
| VC_A0806 | 12.96 | 0.20 | 41.43 | 173.00 | -2.09 | 0.61 | 0.018 |
| VC_A0805 | 37.33 | 0.71 | 406.76 | 412.00 | -0.02 | 0.05 | 0.214 |
| VC_A0804 | 26.26 | 1.03 | 142.93 | 462.00 | -1.70 | 0.27 | 0.019 |

| | | | | | | | |
|---|---|---|---|---|---|---|---|
| VC_A0803 | 40.19 | 1.06 | 450.11 | 340.00 | 0.40 | 0.03 | 0.508 |
| VC_A0802 | 48.57 | 1.30 | 388.80 | 320.00 | 0.28 | 0.04 | 0.673 |
| VC_A0801 | 40.60 | 0.55 | 453.05 | 597.00 | -0.40 | 0.07 | 0.299 |
| VC_A0800 | 7.62 | 0.49 | 23.37 | 41.00 | -0.87 | 0.43 | 0.238 |
| VC_A0799 | 6.00 | 0.00 | 65.52 | 121.00 | -0.91 | 0.24 | 0.309 |
| VC_A0798 | 9.61 | 0.49 | 129.98 | 131.00 | -0.02 | 0.10 | 0.817 |
| VC_A0797 | 3.54 | 0.50 | 15.80 | 10.00 | 0.54 | 0.23 | 0.645 |
| VC_A0796 | 1.98 | 0.14 | 4.83 | 4.00 | -0.21 | 0.87 | 1.000 |
| VC_A0795 | 44.61 | 0.53 | 783.09 | 374.00 | 1.06 | 0.02 | 0.052 |
| VC_A0794 | 12.00 | 0.00 | 68.05 | 53.00 | 0.34 | 0.10 | 0.415 |
| VC_A0793 | 19.74 | 0.96 | 218.67 | 135.00 | 0.69 | 0.04 | 0.127 |
| VC_A0792 | 17.03 | 0.77 | 123.16 | 52.00 | 1.23 | 0.04 | 0.113 |
| VC_A0791 | 40.63 | 1.03 | 299.60 | 302.00 | -0.02 | 0.06 | 0.809 |
| VC_A0790 | 56.39 | 1.20 | 500.40 | 384.00 | 0.38 | 0.04 | 0.216 |
| VC_A0789 | 21.62 | 0.49 | 251.28 | 313.00 | -0.32 | 0.07 | 0.549 |
| VC_A0788 | 14.83 | 0.38 | 122.49 | 135.00 | -0.15 | 0.10 | 0.826 |
| VC_A0787 | 0.00 | 0.00 | 1.00 | 1.00 | #VALEUR! | NaN | NaN |
| VC_A0786 | 2.00 | 0.00 | 1.00 | 25.00 | -4.64 | 0.00 | 0.333 |
| VC_A0785 | 29.98 | 0.14 | 284.49 | 363.00 | -0.36 | 0.07 | 0.343 |
| VC_A0784 | 28.81 | 0.98 | 217.43 | 300.00 | -0.47 | 0.09 | 0.861 |
| VC_A0783 | 16.79 | 0.41 | 279.62 | 197.00 | 0.50 | 0.04 | 0.645 |
| VC_A0782 | 13.67 | 0.47 | 154.17 | 136.00 | 0.17 | 0.07 | 0.290 |
| VC_A0781 | 49.21 | 0.80 | 577.16 | 647.00 | -0.17 | 0.05 | 0.879 |
| VC_A0780 | 32.35 | 0.70 | 358.11 | 375.00 | -0.07 | 0.06 | 0.608 |
| VC_A0779 | 16.87 | 0.34 | 301.22 | 189.00 | 0.67 | 0.03 | 0.270 |
| VC_A0778 | 20.65 | 0.50 | 196.93 | 232.00 | -0.24 | 0.09 | 0.885 |
| VC_A0777 | 15.62 | 0.49 | 329.07 | 242.00 | 0.44 | 0.04 | 0.565 |
| VC_A0776 | 17.67 | 0.47 | 333.96 | 295.00 | 0.18 | 0.05 | 0.593 |
| VC_A0775 | 6.00 | 0.00 | 64.16 | 13.00 | 2.28 | 0.02 | 0.143 |
| VC_A0774 | 31.36 | 0.63 | 299.36 | 235.00 | 0.34 | 0.05 | 0.095 |
| VC_A0773 | 21.40 | 0.67 | 172.30 | 228.00 | -0.41 | 0.10 | 0.590 |
| VC_A0772 | 41.15 | 0.80 | 361.31 | 338.00 | 0.09 | 0.05 | 0.604 |
| VC_A0771 | 3.00 | 0.00 | 24.33 | 1.00 | 4.55 | 0.01 | 0.400 |
| VC_A0770 | 3.00 | 0.00 | 20.25 | 28.00 | -0.54 | 0.36 | 0.794 |
| VC_A0769 | 13.85 | 0.81 | 203.76 | 110.00 | 0.88 | 0.04 | 0.029 |
| VC_A0768 | 32.64 | 0.56 | 333.82 | 429.00 | -0.37 | 0.08 | 0.148 |
| VC_A0767 | 23.00 | 0.00 | 333.48 | 288.00 | 0.21 | 0.05 | 0.139 |
| VC_A0766 | 12.98 | 0.14 | 70.19 | 102.00 | -0.56 | 0.17 | 0.602 |
| VC_A0765 | 21.62 | 0.51 | 201.74 | 193.00 | 0.06 | 0.07 | 0.511 |
| VC_A0764 | 7.19 | 0.75 | 24.47 | 18.00 | 0.38 | 0.16 | 0.771 |

| | | | | | | | |
|---|---|---|---|---|---|---|---|
| VC_A0763 | 37.69 | 0.46 | 514.49 | 666.00 | -0.38 | 0.06 | 0.166 |
| VC_A0762 | 36.37 | 1.10 | 386.59 | 644.00 | -0.74 | 0.09 | 0.633 |
| VC_A0761 | 1.00 | 0.00 | 2.22 | 7.00 | -2.11 | 2.20 | 1.000 |
| VC_A0760 | 10.75 | 0.46 | 76.05 | 70.00 | 0.10 | 0.10 | 0.349 |
| VC_A0759 | 14.76 | 0.45 | 53.85 | 118.00 | -1.16 | 0.34 | 0.648 |
| VC_A0758 | 15.62 | 0.53 | 76.64 | 127.00 | -0.75 | 0.21 | 0.576 |
| VC_A0757 | 17.98 | 0.14 | 222.29 | 98.00 | 1.18 | 0.03 | 0.088 |
| VC_A0756 | 22.32 | 1.02 | 108.64 | 191.00 | -0.83 | 0.19 | 0.830 |
| VC_A0755 | 13.49 | 0.58 | 137.22 | 106.00 | 0.36 | 0.08 | 0.545 |
| VC_A0754 | 18.96 | 0.20 | 238.25 | 119.00 | 1.00 | 0.03 | 0.574 |
| VC_A0753 | 33.18 | 1.09 | 218.44 | 306.00 | -0.49 | 0.10 | 0.692 |
| VC_A0752 | 4.68 | 0.51 | 21.65 | 2.00 | 3.38 | 0.02 | 0.030 |
| VC_A0751 | 24.82 | 0.41 | 246.12 | 170.00 | 0.53 | 0.04 | 0.865 |
| VC_A0750 | 4.00 | 0.00 | 2.37 | 40.00 | -4.57 | 12.59 | 0.056 |
| VC_A0749 | 25.55 | 0.63 | 240.48 | 360.00 | -0.59 | 0.09 | 0.373 |
| VC_A0748 | 14.90 | 0.30 | 126.47 | 83.00 | 0.60 | 0.06 | 0.232 |
| VC_A0747 | 26.68 | 0.49 | 299.62 | 113.00 | 1.40 | 0.02 | 0.037 |
| VC_A0746 | 6.89 | 0.31 | 22.93 | 24.00 | -0.15 | 0.29 | 0.646 |
| VC_A0745 | 15.45 | 1.18 | 148.74 | 82.00 | 0.85 | 0.04 | 0.143 |
| VC_A0744 | 35.62 | 0.56 | 238.75 | 414.00 | -0.80 | 0.11 | 0.022 |
| VC_A0743 | 8.93 | 0.26 | 51.84 | 166.00 | -1.70 | 0.42 | 0.074 |
| VC_A0742 | 1.00 | 0.00 | 6.44 | 1.00 | 2.38 | 0.13 | 1.000 |
| VC_A0741 | 5.00 | 0.00 | 11.64 | 157.00 | -3.93 | 6.36 | 0.130 |
| VC_A0740 | 15.75 | 0.44 | 153.00 | 130.00 | 0.23 | 0.06 | 0.367 |
| VC_A0739 | 6.00 | 0.00 | 26.80 | 192.00 | -2.91 | 1.74 | 0.127 |
| VC_A0738 | 32.29 | 0.67 | 242.19 | 252.00 | -0.06 | 0.06 | 0.453 |
| VC_A0737 | 23.85 | 0.36 | 130.84 | 164.00 | -0.34 | 0.11 | 0.284 |
| VC_A0736 | 46.86 | 1.29 | 313.03 | 441.00 | -0.50 | 0.08 | 0.706 |
| VC_A0735 | 18.61 | 0.49 | 256.00 | 288.00 | -0.18 | 0.08 | 0.636 |
| VC_A0734 | 16.00 | 0.00 | 160.71 | 72.00 | 1.15 | 0.04 | 0.417 |
| VC_A0733 | 2.00 | 0.00 | 1.00 | 2.00 | -1.00 | 0.00 | 0.333 |
| VC_A0732 | 8.67 | 0.47 | 101.19 | 118.00 | -0.24 | 0.13 | 0.772 |
| VC_A0731 | 8.15 | 0.73 | 78.38 | 30.00 | 1.37 | 0.05 | 0.122 |
| VC_A0730 | 75.63 | 1.24 | 667.40 | 637.00 | 0.07 | 0.04 | 0.319 |
| VC_A0729 | 22.00 | 0.00 | 246.30 | 137.00 | 0.84 | 0.03 | 0.157 |
| VC_A0728 | 74.64 | 1.04 | 583.56 | 492.00 | 0.24 | 0.04 | 0.886 |
| VC_A0727 | 24.55 | 0.59 | 156.07 | 332.00 | -1.10 | 0.18 | 0.457 |
| VC_A0726 | 13.00 | 0.00 | 178.53 | 143.00 | 0.31 | 0.06 | 0.485 |
| VC_A0725 | 10.00 | 0.00 | 77.90 | 88.00 | -0.19 | 0.13 | 0.496 |
| VC_A0724 | 14.59 | 0.57 | 129.34 | 194.00 | -0.60 | 0.13 | 0.713 |

| | | | | | | | |
|---|---|---|---|---|---|---|---|
| VC_A0723 | 22.47 | 0.56 | 239.82 | 183.00 | 0.38 | 0.05 | 0.810 |
| VC_A0722 | 4.62 | 0.49 | 31.58 | 105.00 | -1.78 | 0.65 | 0.888 |
| VC_A0721 | 5.00 | 0.00 | 26.79 | 58.00 | -1.17 | 0.49 | 0.657 |
| VC_A0720 | 16.91 | 0.29 | 110.96 | 148.00 | -0.43 | 0.12 | 0.408 |
| VC_A0719 | 18.13 | 0.92 | 171.44 | 102.00 | 0.74 | 0.05 | 0.016 |
| VC_A0718 | 39.37 | 0.96 | 548.25 | 597.00 | -0.13 | 0.05 | 0.810 |
| VC_A0717 | 41.34 | 0.83 | 447.76 | 572.00 | -0.36 | 0.06 | 0.712 |
| VC_A0716 | 10.00 | 0.00 | 176.95 | 138.00 | 0.35 | 0.06 | 0.869 |
| VC_A0715 | 12.99 | 0.10 | 35.75 | 38.00 | -0.14 | 0.22 | 0.703 |
| VC_A0714 | 9.69 | 0.49 | 34.29 | 48.00 | -0.55 | 0.37 | 0.643 |
| VC_A0713 | 2.70 | 0.48 | 4.87 | 9.00 | -1.25 | 1.61 | 1.000 |
| VC_A0712 | 9.85 | 0.36 | 66.23 | 38.00 | 0.78 | 0.08 | 0.315 |
| VC_A0711 | 8.00 | 0.00 | 53.11 | 164.00 | -1.66 | 0.56 | 0.480 |
| VC_A0710 | 17.67 | 0.53 | 183.69 | 235.00 | -0.36 | 0.10 | 0.307 |
| VC_A0709 | 49.49 | 0.99 | 488.11 | 598.00 | -0.30 | 0.06 | 0.418 |
| VC_A0708 | 22.87 | 0.34 | 271.84 | 376.00 | -0.47 | 0.08 | 0.190 |
| VC_A0707 | 32.86 | 0.93 | 390.75 | 491.00 | -0.33 | 0.06 | 0.439 |
| VC_A0706 | 27.72 | 0.49 | 333.09 | 277.00 | 0.26 | 0.05 | 0.860 |
| VC_A0705 | 33.82 | 0.87 | 312.51 | 410.00 | -0.40 | 0.07 | 0.195 |
| VC_A0704 | 28.41 | 0.62 | 382.92 | 447.00 | -0.23 | 0.06 | 0.688 |
| VC_A0703 | 21.66 | 0.59 | 178.47 | 244.00 | -0.46 | 0.12 | 0.327 |
| VC_A0702 | 21.45 | 1.07 | 263.27 | 289.00 | -0.14 | 0.07 | 0.570 |
| VC_A0701 | 1.00 | 0.00 | 1.00 | 1.00 | 0.00 | 0.00 | 1.000 |
| VC_A0700 | 71.67 | 1.10 | 1495.09 | 1199.00 | 0.32 | 0.02 | 0.110 |
| VC_A0699 | 28.98 | 1.12 | 559.70 | 250.00 | 1.16 | 0.02 | 0.084 |
| VC_A0698 | 3.00 | 0.00 | 4.00 | 18.00 | -2.61 | 4.31 | 0.405 |
| VC_A0697 | 51.41 | 1.34 | 546.04 | 519.00 | 0.07 | 0.04 | 0.348 |
| VC_A0696 | 5.57 | 0.52 | 26.89 | 8.00 | 1.70 | 0.06 | 0.151 |
| VC_A0695 | 17.39 | 0.67 | 104.06 | 148.00 | -0.52 | 0.13 | 0.092 |
| VC_A0694 | 10.80 | 0.40 | 215.76 | 163.00 | 0.40 | 0.05 | 0.591 |
| VC_A0693 | 30.44 | 1.00 | 303.66 | 342.00 | -0.18 | 0.07 | 0.515 |
| VC_A0692 | 16.06 | 0.84 | 81.12 | 165.00 | -1.04 | 0.21 | 0.266 |
| VC_A0691 | 19.83 | 0.38 | 132.12 | 226.00 | -0.79 | 0.15 | 0.201 |
| VC_A0690 | 22.07 | 0.62 | 247.89 | 256.00 | -0.05 | 0.07 | 0.399 |
| VC_A0689 | 7.00 | 0.00 | 59.68 | 46.00 | 0.35 | 0.11 | 0.844 |
| VC_A0688 | 36.39 | 0.60 | 412.55 | 545.00 | -0.40 | 0.06 | 0.575 |
| VC_A0687 | 27.18 | 0.81 | 440.78 | 202.00 | 1.12 | 0.02 | 0.016 |
| VC_A0686 | 52.62 | 0.58 | 483.54 | 406.00 | 0.25 | 0.04 | 0.672 |
| VC_A0685 | 21.44 | 0.62 | 148.52 | 215.00 | -0.54 | 0.12 | 0.673 |
| VC_A0684 | 31.79 | 0.41 | 263.53 | 173.00 | 0.60 | 0.04 | 0.185 |

| | | | | | | | |
|---|---|---|---|---|---|---|---|
| VC_A0683 | 29.86 | 0.91 | 181.16 | 207.00 | -0.20 | 0.10 | 0.514 |
| VC_A0682 | 14.11 | 0.72 | 185.82 | 158.00 | 0.23 | 0.07 | 0.286 |
| VC_A0681 | 29.61 | 0.49 | 342.45 | 332.00 | 0.04 | 0.05 | 0.808 |
| VC_A0680 | 7.00 | 0.00 | 104.94 | 46.00 | 1.18 | 0.04 | 0.706 |
| VC_A0679 | 3.00 | 0.00 | 17.68 | 45.00 | -1.42 | 0.59 | 0.532 |
| VC_A0678 | 42.93 | 1.22 | 402.14 | 453.00 | -0.18 | 0.06 | 0.129 |
| VC_A0677 | 5.92 | 0.27 | 83.76 | 3.00 | 4.79 | 0.00 | 0.028 |
| VC_A0676 | 5.99 | 0.10 | 61.48 | 125.00 | -1.04 | 0.23 | 0.951 |
| VC_A0675 | 34.70 | 0.94 | 350.78 | 369.00 | -0.08 | 0.06 | 0.502 |
| VC_A0674 | 10.60 | 0.60 | 101.20 | 89.00 | 0.17 | 0.09 | 0.876 |
| VC_A0673 | 25.66 | 0.82 | 151.51 | 179.00 | -0.25 | 0.10 | 0.632 |
| VC_A0672 | 4.71 | 0.46 | 9.83 | 26.00 | -1.58 | 1.25 | 0.394 |
| VC_A0671 | 23.05 | 0.93 | 181.55 | 164.00 | 0.14 | 0.06 | 0.100 |
| VC_A0670 | 4.00 | 0.00 | 22.57 | 45.00 | -1.07 | 0.50 | 0.240 |
| VC_A0669 | 28.22 | 0.76 | 311.12 | 202.00 | 0.62 | 0.04 | 0.681 |
| VC_A0668 | 2.00 | 0.00 | 35.75 | 18.00 | 0.94 | 0.10 | 0.417 |
| VC_A0667 | 45.59 | 1.06 | 437.09 | 275.00 | 0.67 | 0.03 | 0.213 |
| VC_A0666 | 23.24 | 0.74 | 149.09 | 249.00 | -0.75 | 0.15 | 0.199 |
| VC_A0665 | 44.57 | 0.50 | 705.14 | 639.00 | 0.14 | 0.03 | 0.512 |
| VC_A0664 | 4.00 | 0.00 | 71.46 | 12.00 | 2.56 | 0.02 | 0.165 |
| VC_A0663 | 34.83 | 0.85 | 336.34 | 337.00 | -0.01 | 0.05 | 0.383 |
| VC_A0662 | 12.36 | 0.70 | 142.78 | 30.00 | 2.24 | 0.02 | 0.089 |
| VC_A0661 | 13.71 | 0.50 | 128.16 | 57.00 | 1.16 | 0.04 | 0.399 |
| VC_A0660 | 5.84 | 0.37 | 71.76 | 2.00 | 5.15 | 0.00 | 0.028 |
| VC_A0659 | 16.33 | 0.70 | 90.29 | 195.00 | -1.13 | 0.25 | 0.367 |
| VC_A0658 | 36.26 | 1.21 | 342.30 | 436.00 | -0.35 | 0.07 | 0.781 |
| VC_A0657 | 28.23 | 1.00 | 337.08 | 178.00 | 0.92 | 0.03 | 0.072 |
| VC_A0656 | 17.18 | 0.74 | 126.25 | 182.00 | -0.54 | 0.14 | 0.855 |
| VC_A0655 | 33.53 | 0.63 | 380.22 | 283.00 | 0.42 | 0.04 | 0.106 |
| VC_A0654 | 13.70 | 0.46 | 111.21 | 152.00 | -0.46 | 0.11 | 0.708 |
| VC_A0653 | 22.88 | 0.33 | 220.54 | 424.00 | -0.95 | 0.12 | 0.062 |
| VC_A0652 | 6.00 | 0.00 | 51.02 | 47.00 | 0.08 | 0.17 | 0.859 |
| VC_A0651 | 15.65 | 0.48 | 78.54 | 324.00 | -2.06 | 0.50 | 0.039 |
| VC_A0650 | 23.95 | 0.22 | 362.90 | 377.00 | -0.06 | 0.06 | 0.705 |
| VC_A0649 | 12.89 | 0.31 | 199.35 | 234.00 | -0.24 | 0.09 | 0.913 |
| VC_A0648 | 10.89 | 0.31 | 162.33 | 95.00 | 0.76 | 0.05 | 0.211 |
| VC_A0647 | 8.99 | 0.10 | 22.92 | 106.00 | -2.28 | 1.12 | 0.124 |
| VC_A0646 | 42.97 | 0.81 | 578.37 | 658.00 | -0.19 | 0.05 | 0.190 |
| VC_A0645 | 10.64 | 0.48 | 32.67 | 72.00 | -1.19 | 0.47 | 0.030 |
| VC_A0644 | 35.19 | 1.07 | 291.91 | 175.00 | 0.73 | 0.04 | 0.533 |

| | | | | | | | |
|---|---|---|---|---|---|---|---|
| VC_A0643 | 8.00 | 0.00 | 60.94 | 69.00 | -0.21 | 0.16 | 0.429 |
| VC_A0642 | 2.00 | 0.00 | 8.97 | 5.00 | 0.70 | 0.21 | 1.000 |
| VC_A0641 | 4.00 | 0.00 | 75.43 | 58.00 | 0.35 | 0.11 | 0.819 |
| VC_A0640 | 5.62 | 0.49 | 76.45 | 40.00 | 0.92 | 0.06 | 0.375 |
| VC_A0639 | 20.63 | 0.49 | 227.29 | 248.00 | -0.13 | 0.08 | 0.407 |
| VC_A0638 | 54.37 | 1.01 | 660.08 | 917.00 | -0.48 | 0.05 | 0.707 |
| VC_A0637 | 11.64 | 0.48 | 136.59 | 106.00 | 0.36 | 0.06 | 0.903 |
| VC_A0636 | 4.97 | 0.17 | 34.62 | 33.00 | 0.02 | 0.19 | 0.762 |
| VC_A0635 | 16.31 | 1.13 | 145.86 | 218.00 | -0.59 | 0.12 | 0.748 |
| VC_A0634 | 13.38 | 0.63 | 72.61 | 201.00 | -1.49 | 0.32 | 0.219 |
| VC_A0633 | 8.00 | 0.00 | 43.48 | 50.00 | -0.24 | 0.19 | 0.282 |
| VC_A0632 | 5.63 | 0.51 | 22.07 | 18.00 | 0.20 | 0.26 | 0.748 |
| VC_A0631 | 12.00 | 0.00 | 177.86 | 101.00 | 0.81 | 0.04 | 0.595 |
| VC_A0630 | 16.36 | 0.69 | 237.52 | 99.00 | 1.26 | 0.03 | 0.237 |
| VC_A0629 | 16.40 | 0.65 | 175.18 | 173.00 | 0.01 | 0.07 | 0.775 |
| VC_A0628 | 18.23 | 1.47 | 55.67 | 30.00 | 0.87 | 0.07 | 0.154 |
| VC_A0627 | 11.98 | 0.14 | 222.53 | 131.00 | 0.76 | 0.04 | 0.247 |
| VC_A0626 | 3.00 | 0.00 | 35.07 | 47.00 | -0.46 | 0.24 | 1.000 |
| VC_A0625 | 50.78 | 1.54 | 498.35 | 358.00 | 0.47 | 0.03 | 0.715 |
| VC_A0624 | 34.59 | 0.51 | 541.07 | 708.00 | -0.39 | 0.06 | 0.455 |
| VC_A0623 | 13.59 | 0.49 | 108.03 | 233.00 | -1.12 | 0.20 | 0.396 |
| VC_A0622 | 3.00 | 0.00 | 53.60 | 6.00 | 3.13 | 0.02 | 0.504 |
| VC_A0621 | 11.30 | 0.66 | 220.27 | 225.00 | -0.04 | 0.07 | 0.752 |
| VC_A0620 | 11.85 | 0.36 | 238.79 | 197.00 | 0.27 | 0.05 | 0.670 |
| VC_A0619 | 5.00 | 0.00 | 102.51 | 20.00 | 2.34 | 0.02 | 0.220 |
| VC_A0618 | 12.88 | 0.33 | 83.70 | 158.00 | -0.93 | 0.20 | 0.565 |
| VC_A0617 | 14.90 | 0.30 | 202.38 | 128.00 | 0.65 | 0.05 | 0.282 |
| VC_A0616 | 0.88 | 0.33 | 2.18 | 1.00 | 0.78 | 0.31 | 1.000 |
| VC_A0615 | 32.80 | 0.90 | 245.35 | 243.00 | 0.01 | 0.07 | 0.885 |
| VC_A0614 | 25.91 | 0.29 | 224.72 | 206.00 | 0.12 | 0.07 | 0.270 |
| VC_A0613 | 1.99 | 0.10 | 6.24 | 39.00 | -3.02 | 5.86 | 1.000 |
| VC_A0612 | 9.38 | 0.62 | 94.40 | 36.00 | 1.37 | 0.05 | 0.593 |
| VC_A0611 | 3.00 | 0.00 | 1.00 | 51.00 | -5.67 | 0.00 | 0.100 |
| VC_A0610 | 10.43 | 0.56 | 182.88 | 55.00 | 1.73 | 0.02 | 0.084 |
| VC_A0609 | 6.00 | 0.00 | 70.13 | 50.00 | 0.47 | 0.09 | 0.762 |
| VC_A0608 | 21.34 | 1.08 | 152.24 | 107.00 | 0.50 | 0.06 | 0.039 |
| VC_A0607 | 7.98 | 0.14 | 140.17 | 16.00 | 3.12 | 0.01 | 0.003 |
| VC_A0606 | 13.91 | 0.29 | 101.66 | 129.00 | -0.36 | 0.13 | 0.644 |
| VC_A0605 | 27.08 | 0.81 | 286.25 | 199.00 | 0.52 | 0.04 | 0.422 |
| VC_A0604 | 22.12 | 0.76 | 198.29 | 320.00 | -0.70 | 0.14 | 0.879 |

| | | | | | | | |
|---|---|---|---|---|---|---|---|
| VC_A0603 | 19.59 | 0.88 | 233.23 | 323.00 | -0.48 | 0.11 | 0.360 |
| VC_A0602 | 19.61 | 0.57 | 197.69 | 231.00 | -0.23 | 0.08 | 0.571 |
| VC_A0601 | 42.14 | 1.13 | 380.27 | 433.00 | -0.19 | 0.07 | 0.490 |
| VC_A0600 | 16.79 | 0.41 | 52.59 | 240.00 | -2.22 | 0.65 | 0.032 |
| VC_A0599 | 28.24 | 0.79 | 226.29 | 247.00 | -0.13 | 0.08 | 0.809 |
| VC_A0598 | 2.00 | 0.00 | 7.04 | 9.00 | -0.65 | 1.01 | 0.790 |
| VC_A0596 | 27.90 | 0.89 | 224.82 | 242.00 | -0.11 | 0.07 | 0.801 |
| VC_A0595 | 20.64 | 0.50 | 244.45 | 200.00 | 0.28 | 0.06 | 0.568 |
| VC_A0594 | 7.62 | 0.58 | 77.51 | 107.00 | -0.49 | 0.18 | 0.643 |
| VC_A0593 | 21.98 | 0.14 | 194.00 | 230.00 | -0.25 | 0.09 | 0.205 |
| VC_A0592 | 15.75 | 0.50 | 130.27 | 105.00 | 0.30 | 0.07 | 0.157 |
| VC_A0591 | 33.17 | 0.77 | 255.32 | 243.00 | 0.07 | 0.06 | 0.638 |
| VC_A0590 | 20.63 | 0.49 | 256.30 | 229.00 | 0.16 | 0.06 | 0.873 |
| VC_A0589 | 28.12 | 0.88 | 431.48 | 228.00 | 0.92 | 0.03 | 0.860 |
| VC_A0588 | 31.23 | 0.66 | 275.32 | 333.00 | -0.28 | 0.08 | 0.176 |
| VC_A0587 | 7.00 | 0.00 | 53.14 | 151.00 | -1.54 | 0.43 | 0.769 |
| VC_A0586 | 7.74 | 0.44 | 81.17 | 55.00 | 0.54 | 0.08 | 0.613 |
| VC_A0585 | 9.90 | 0.30 | 114.54 | 61.00 | 0.90 | 0.05 | 0.556 |
| VC_A0584 | 9.13 | 0.79 | 35.00 | 220.00 | -2.69 | 1.09 | 0.734 |
| VC_A0583 | 20.50 | 0.58 | 233.17 | 147.00 | 0.66 | 0.04 | 0.373 |
| VC_A0582 | 4.63 | 0.51 | 11.17 | 33.00 | -1.74 | 1.43 | 0.909 |
| VC_A0581 | 18.90 | 0.30 | 144.92 | 152.00 | -0.08 | 0.09 | 0.662 |
| VC_A0580 | 10.65 | 0.48 | 59.75 | 175.00 | -1.57 | 0.35 | 0.012 |
| VC_A0579 | 3.00 | 0.00 | 17.45 | 22.00 | -0.41 | 0.30 | 0.754 |
| VC_A0578 | 30.12 | 1.18 | 144.65 | 127.00 | 0.17 | 0.08 | 0.756 |
| VC_A0577 | 2.86 | 0.35 | 2.02 | 5.00 | -1.69 | 1.50 | 0.682 |
| VC_A0576 | 72.38 | 1.42 | 535.62 | 397.00 | 0.43 | 0.03 | 0.854 |
| VC_A0575 | 24.01 | 0.81 | 277.56 | 266.00 | 0.06 | 0.06 | 0.287 |
| VC_A0574 | 69.39 | 0.97 | 784.59 | 667.00 | 0.23 | 0.04 | 0.906 |
| VC_A0573 | 11.84 | 0.90 | 210.07 | 114.00 | 0.87 | 0.04 | 0.634 |
| VC_A0572 | 2.59 | 0.49 | 34.57 | 1.00 | 5.07 | 0.01 | 0.196 |
| VC_A0571 | 5.88 | 0.33 | 4.30 | 42.00 | -3.71 | 8.94 | 0.151 |
| VC_A0570 | 7.97 | 0.17 | 20.48 | 38.00 | -0.98 | 0.57 | 0.810 |
| VC_A0569 | 1.00 | 0.00 | 1.00 | 11.00 | -3.46 | 0.00 | 1.000 |
| VC_A0568 | 38.34 | 0.70 | 377.90 | 172.00 | 1.13 | 0.02 | 0.001 |
| VC_A0567 | 22.67 | 0.99 | 130.05 | 128.00 | 0.01 | 0.08 | 0.843 |
| VC_A0566 | 0.00 | 0.00 | 1.00 | 1.00 | #VALEUR! | NaN | NaN |
| VC_A0565 | 4.65 | 0.48 | 1.32 | 15.00 | -3.72 | 3.39 | 0.075 |
| VC_A0564 | 31.48 | 0.58 | 312.54 | 408.00 | -0.39 | 0.07 | 0.298 |
| VC_A0563 | 41.17 | 1.14 | 790.53 | 334.00 | 1.24 | 0.02 | 0.111 |

| | | | | | | | |
|---|---|---|---|---|---|---|---|
| VC_A0562 | 4.96 | 0.20 | 27.97 | 2.00 | 3.74 | 0.02 | 0.158 |
| VC_A0561 | 9.00 | 0.00 | 80.36 | 197.00 | -1.31 | 0.26 | 0.233 |
| VC_A0560 | 42.47 | 0.54 | 584.71 | 510.00 | 0.19 | 0.04 | 0.877 |
| VC_A0559 | 28.61 | 0.53 | 308.69 | 345.00 | -0.17 | 0.07 | 0.626 |
| VC_A0558 | 33.83 | 0.87 | 191.46 | 424.00 | -1.15 | 0.16 | 0.029 |
| VC_A0557 | 46.02 | 0.91 | 597.81 | 475.00 | 0.33 | 0.03 | 0.380 |
| VC_A0556 | 15.24 | 0.73 | 222.95 | 148.00 | 0.58 | 0.05 | 0.507 |
| VC_A0555 | 1.94 | 0.24 | 3.08 | 1.00 | 1.13 | 0.30 | 1.000 |
| VC_A0554 | 29.60 | 0.55 | 389.13 | 459.00 | -0.24 | 0.06 | 0.195 |
| VC_A0553 | 1.00 | 0.00 | 3.17 | 3.00 | -0.41 | 0.86 | 1.000 |
| VC_A0552 | 40.19 | 0.71 | 588.32 | 537.00 | 0.13 | 0.04 | 0.900 |
| VC_A0551 | 4.66 | 0.48 | 88.26 | 91.00 | -0.06 | 0.11 | 0.895 |
| VC_A0550 | 25.27 | 0.76 | 148.76 | 426.00 | -1.53 | 0.23 | 0.252 |
| VC_A0549 | 15.28 | 0.71 | 190.70 | 194.00 | -0.03 | 0.08 | 0.660 |
| VC_A0548 | 3.61 | 0.49 | 15.86 | 18.00 | -0.32 | 0.42 | 0.790 |
| VC_A0547 | 3.00 | 0.00 | 133.03 | 67.00 | 0.98 | 0.05 | 0.700 |
| VC_A0546 | 20.33 | 0.65 | 143.63 | 164.00 | -0.20 | 0.11 | 0.220 |
| VC_A0545 | 36.59 | 0.53 | 776.04 | 467.00 | 0.73 | 0.02 | 0.908 |
| VC_A0544 | 24.15 | 0.76 | 433.03 | 317.00 | 0.45 | 0.03 | 0.151 |
| VC_A0543 | 11.69 | 0.46 | 59.03 | 145.00 | -1.32 | 0.33 | 0.607 |
| VC_A0542 | 21.57 | 0.90 | 283.95 | 170.00 | 0.74 | 0.03 | 0.282 |
| VC_A0541 | 0.96 | 0.20 | 3.18 | 1.00 | 1.29 | 0.25 | 1.000 |
| VC_A0540 | 33.47 | 0.58 | 319.73 | 380.00 | -0.25 | 0.07 | 0.873 |
| VC_A0539 | 8.38 | 0.63 | 45.06 | 130.00 | -1.56 | 0.44 | 0.218 |
| VC_A0538 | 15.58 | 0.55 | 143.89 | 202.00 | -0.50 | 0.11 | 0.896 |
| VC_A0537 | 13.93 | 0.26 | 174.96 | 129.00 | 0.43 | 0.05 | 0.922 |
| VC_A0536 | 30.59 | 0.53 | 352.58 | 252.00 | 0.48 | 0.04 | 0.667 |
| VC_A0535 | 7.93 | 0.26 | 101.73 | 116.00 | -0.20 | 0.12 | 0.219 |
| VC_A0534 | 18.62 | 0.49 | 97.16 | 335.00 | -1.80 | 0.34 | 0.028 |
| VC_A0533 | 4.00 | 0.00 | 17.20 | 47.00 | -1.54 | 0.72 | 0.795 |
| VC_A0532 | 14.96 | 0.20 | 208.53 | 142.00 | 0.55 | 0.05 | 0.583 |
| VC_A0531 | 35.45 | 0.59 | 372.09 | 307.00 | 0.27 | 0.04 | 0.361 |
| VC_A0530 | 72.08 | 1.15 | 1055.93 | 789.00 | 0.42 | 0.02 | 0.125 |
| VC_A0529 | 44.95 | 1.53 | 742.49 | 661.00 | 0.17 | 0.04 | 0.487 |
| VC_A0528 | 1.00 | 0.00 | 1.00 | 4.00 | -2.00 | 0.00 | 1.000 |
| VC_A0527 | 17.95 | 0.22 | 291.11 | 134.00 | 1.11 | 0.03 | 0.203 |
| VC_A0526 | 36.23 | 0.69 | 366.52 | 395.00 | -0.11 | 0.06 | 0.568 |
| VC_A0525 | 5.00 | 0.00 | 6.30 | 47.00 | -3.19 | 4.61 | 0.078 |
| VC_A0524 | 25.61 | 1.04 | 134.06 | 236.00 | -0.83 | 0.16 | 0.474 |
| VC_A0523 | 27.71 | 1.23 | 332.09 | 273.00 | 0.28 | 0.05 | 0.596 |

| | | | | | | | |
|---|---|---|---|---|---|---|---|
| VC_A0522 | 61.85 | 0.95 | 577.80 | 445.00 | 0.37 | 0.03 | 0.474 |
| VC_A0521 | 49.11 | 0.75 | 374.85 | 364.00 | 0.04 | 0.05 | 0.723 |
| VC_A0520 | 19.00 | 0.00 | 190.05 | 242.00 | -0.36 | 0.10 | 0.486 |
| VC_A0519 | 15.99 | 0.10 | 191.75 | 138.00 | 0.47 | 0.05 | 0.944 |
| VC_A0518 | 27.98 | 0.14 | 496.61 | 426.00 | 0.22 | 0.03 | 0.942 |
| VC_A0517 | 16.59 | 0.49 | 152.03 | 250.00 | -0.73 | 0.15 | 0.869 |
| VC_A0516 | 30.72 | 0.94 | 320.32 | 393.00 | -0.30 | 0.07 | 0.394 |
| VC_A0515 | 5.74 | 0.82 | 18.73 | 17.00 | 0.07 | 0.24 | 0.843 |
| VC_A0514 | 20.98 | 0.14 | 173.61 | 148.00 | 0.22 | 0.07 | 0.480 |
| VC_A0513 | 23.58 | 0.52 | 299.18 | 429.00 | -0.52 | 0.08 | 0.912 |
| VC_A0512 | 18.65 | 0.48 | 123.49 | 206.00 | -0.75 | 0.15 | 0.226 |
| VC_A0511 | 43.39 | 0.62 | 516.29 | 729.00 | -0.50 | 0.07 | 0.189 |
| VC_A0510 | 83.61 | 1.85 | 1385.58 | 1086.00 | 0.35 | 0.02 | 0.146 |
| VC_A0509 | 7.66 | 0.50 | 9.64 | 142.00 | -4.08 | 8.45 | 0.018 |
| VC_A0508 | 43.64 | 0.93 | 486.31 | 563.00 | -0.21 | 0.06 | 0.366 |
| VC_A0507 | 16.98 | 0.14 | 166.88 | 81.00 | 1.03 | 0.04 | 0.111 |
| VC_A0506 | 12.29 | 0.73 | 45.69 | 42.00 | 0.08 | 0.16 | 0.717 |
| VC_A0505 | 28.27 | 1.06 | 193.63 | 170.00 | 0.18 | 0.06 | 0.621 |
| VC_A0504 | 7.00 | 0.00 | 48.31 | 43.00 | 0.14 | 0.14 | 0.903 |
| VC_A0503 | 5.97 | 0.17 | 44.23 | 16.00 | 1.43 | 0.06 | 0.547 |
| VC_A0502 | 5.00 | 0.00 | 81.54 | 41.00 | 0.97 | 0.06 | 0.476 |
| VC_A0501 | 9.30 | 0.64 | 49.98 | 137.00 | -1.49 | 0.43 | 0.593 |
| VC_A0500 | 3.00 | 0.00 | 16.58 | 75.00 | -2.27 | 1.22 | 0.214 |
| VC_A0499 | 2.99 | 0.10 | 5.65 | 19.00 | -2.02 | 2.27 | 0.473 |
| VC_A0498 | 6.97 | 0.17 | 39.88 | 90.00 | -1.21 | 0.42 | 0.425 |
| VC_A0497 | 16.91 | 0.32 | 101.12 | 216.00 | -1.11 | 0.22 | 0.355 |
| VC_A0496 | 19.29 | 0.71 | 243.90 | 269.00 | -0.15 | 0.08 | 0.775 |
| VC_A0495 | 38.62 | 0.49 | 186.79 | 230.00 | -0.31 | 0.10 | 0.049 |
| VC_A0494 | 4.99 | 0.10 | 19.19 | 13.00 | 0.49 | 0.17 | 0.785 |
| VC_A0493 | 23.69 | 0.46 | 216.51 | 289.00 | -0.42 | 0.09 | 0.256 |
| VC_A0492 | 4.95 | 0.22 | 20.97 | 11.00 | 0.85 | 0.14 | 0.704 |
| VC_A0491 | 23.90 | 0.88 | 164.76 | 78.00 | 1.07 | 0.04 | 0.287 |
| VC_A0490 | 34.94 | 0.86 | 296.34 | 403.00 | -0.45 | 0.08 | 0.885 |
| VC_A0489 | 4.93 | 0.26 | 57.02 | 104.00 | -0.90 | 0.27 | 0.756 |
| VC_A0488 | 0.00 | 0.00 | 1.00 | 1.00 | #VALEUR! | NaN | NaN |
| VC_A0487 | 15.66 | 0.48 | 84.35 | 272.00 | -1.71 | 0.36 | 0.011 |
| VC_A0486 | 2.95 | 0.22 | 10.70 | 41.00 | -2.09 | 1.51 | 0.731 |
| VC_A0485 | 14.28 | 0.68 | 74.12 | 131.00 | -0.84 | 0.21 | 0.824 |
| VC_A0484 | 19.57 | 0.52 | 138.42 | 125.00 | 0.14 | 0.08 | 0.291 |
| VC_A0483 | 16.92 | 0.27 | 66.96 | 64.00 | 0.04 | 0.13 | 0.675 |

| | | | | | | | |
|---|---|---|---|---|---|---|---|
| VC_A0482 | 11.59 | 0.53 | 117.93 | 67.00 | 0.80 | 0.06 | 0.352 |
| VC_A0481 | 17.31 | 0.68 | 111.17 | 104.00 | 0.08 | 0.09 | 0.508 |
| VC_A0480 | 2.93 | 0.26 | 3.15 | 16.00 | -2.79 | 4.37 | 0.674 |
| VC_A0479 | 13.32 | 0.75 | 65.52 | 120.00 | -0.90 | 0.24 | 0.797 |
| VC_A0478 | 8.60 | 0.49 | 135.73 | 33.00 | 2.03 | 0.02 | 0.048 |
| VC_A0477 | 2.61 | 0.49 | 8.53 | 4.00 | 0.85 | 0.28 | 0.861 |
| VC_A0476 | 15.58 | 0.54 | 86.29 | 78.00 | 0.13 | 0.10 | 0.394 |
| VC_A0475 | 7.67 | 0.47 | 58.32 | 21.00 | 1.45 | 0.05 | 0.832 |
| VC_A0474 | 23.07 | 0.79 | 103.49 | 135.00 | -0.40 | 0.14 | 0.576 |
| VC_A0473 | 15.00 | 0.00 | 208.54 | 372.00 | -0.84 | 0.12 | 0.698 |
| VC_A0472 | 20.95 | 0.22 | 266.75 | 464.00 | -0.80 | 0.11 | 0.324 |
| VC_A0471 | 3.00 | 0.00 | 49.30 | 16.00 | 1.59 | 0.05 | 0.224 |
| VC_A0470 | 19.90 | 0.30 | 54.23 | 89.00 | -0.75 | 0.26 | 0.361 |
| VC_A0469 | 6.61 | 0.51 | 12.90 | 63.00 | -2.40 | 1.76 | 0.724 |
| VC_A0468 | 9.69 | 0.49 | 86.48 | 59.00 | 0.54 | 0.07 | 0.698 |
| VC_A0467 | 4.00 | 0.00 | 115.22 | 40.00 | 1.52 | 0.03 | 0.224 |
| VC_A0466 | 5.67 | 0.47 | 42.07 | 65.00 | -0.67 | 0.30 | 0.681 |
| VC_A0465 | 21.96 | 0.79 | 83.62 | 76.00 | 0.12 | 0.11 | 0.320 |
| VC_A0464 | 10.00 | 0.00 | 132.27 | 117.00 | 0.17 | 0.07 | 0.580 |
| VC_A0463 | 8.95 | 0.22 | 43.55 | 74.00 | -0.79 | 0.26 | 0.465 |
| VC_A0462 | 3.00 | 0.00 | 110.28 | 9.00 | 3.60 | 0.01 | 0.500 |
| VC_A0461 | 1.00 | 0.00 | 40.67 | 9.00 | 2.14 | 0.03 | 1.000 |
| VC_A0460 | 16.79 | 0.43 | 52.55 | 137.00 | -1.41 | 0.35 | 0.163 |
| VC_A0459 | 16.47 | 0.94 | 216.21 | 191.00 | 0.17 | 0.06 | 0.731 |
| VC_A0458 | 26.83 | 0.96 | 124.08 | 119.00 | 0.05 | 0.09 | 0.155 |
| VC_A0457 | 18.95 | 0.82 | 133.47 | 50.00 | 1.41 | 0.03 | 0.136 |
| VC_A0456 | 1.00 | 0.00 | 38.08 | 9.00 | 2.03 | 0.05 | 1.000 |
| VC_A0455 | 20.34 | 0.73 | 183.90 | 264.00 | -0.53 | 0.11 | 0.871 |
| VC_A0454 | 20.85 | 0.39 | 162.34 | 122.00 | 0.40 | 0.06 | 0.217 |
| VC_A0453 | 6.56 | 0.50 | 13.07 | 48.00 | -2.03 | 1.81 | 0.424 |
| VC_A0452 | 2.00 | 0.00 | 23.64 | 1.00 | 4.51 | 0.01 | 0.333 |
| VC_A0451 | 14.49 | 0.59 | 129.58 | 49.00 | 1.39 | 0.04 | 0.296 |
| VC_A0450 | 20.87 | 0.76 | 79.40 | 103.00 | -0.39 | 0.15 | 0.779 |
| VC_A0449 | 10.57 | 0.50 | 161.14 | 64.00 | 1.32 | 0.03 | 0.270 |
| VC_A0448 | 19.49 | 0.97 | 51.86 | 83.00 | -0.70 | 0.22 | 0.641 |
| VC_A0447 | 15.67 | 0.47 | 131.24 | 90.00 | 0.53 | 0.07 | 0.698 |
| VC_A0446 | 5.00 | 0.00 | 56.02 | 34.00 | 0.69 | 0.09 | 0.703 |
| VC_A0445 | 4.00 | 0.00 | 29.80 | 28.00 | 0.02 | 0.22 | 0.785 |
| VC_A0444 | 8.95 | 0.22 | 38.30 | 51.00 | -0.45 | 0.22 | 0.392 |
| VC_A0443 | 21.41 | 0.81 | 109.90 | 186.00 | -0.77 | 0.15 | 0.597 |

| | | | | | | | |
|---|---|---|---|---|---|---|---|
| VC_A0442 | 20.79 | 0.46 | 91.49 | 126.00 | -0.48 | 0.16 | 0.314 |
| VC_A0441 | 35.06 | 0.94 | 256.71 | 139.00 | 0.88 | 0.04 | 0.058 |
| VC_A0440 | 21.73 | 0.53 | 131.15 | 320.00 | -1.30 | 0.24 | 0.822 |
| VC_A0439 | 43.81 | 0.42 | 370.92 | 445.00 | -0.27 | 0.06 | 0.397 |
| VC_A0438 | 0.00 | 0.00 | 1.00 | 1.00 | #VALEUR! | NaN | NaN |
| VC_A0437 | 3.00 | 0.00 | 33.16 | 1.00 | 4.99 | 0.01 | 0.100 |
| VC_A0436 | 28.62 | 0.55 | 220.73 | 227.00 | -0.05 | 0.08 | 0.810 |
| VC_A0435 | 1.72 | 0.55 | 4.27 | 1.00 | 1.76 | 0.19 | 0.460 |
| VC_A0434 | 2.00 | 0.00 | 10.83 | 2.00 | 2.31 | 0.06 | 1.000 |
| VC_A0433 | 25.60 | 0.59 | 135.77 | 186.00 | -0.47 | 0.12 | 0.859 |
| VC_A0432 | 19.96 | 0.20 | 422.61 | 571.00 | -0.44 | 0.06 | 0.934 |
| VC_A0431 | 8.66 | 0.54 | 20.20 | 32.00 | -0.74 | 0.40 | 0.653 |
| VC_A0430 | 1.00 | 0.00 | 20.13 | 5.00 | 1.91 | 0.08 | 1.000 |
| VC_A0429 | 2.00 | 0.00 | 14.77 | 12.00 | 0.21 | 0.22 | 0.837 |
| VC_A0428 | 18.57 | 0.70 | 72.09 | 67.00 | 0.09 | 0.11 | 0.357 |
| VC_A0427 | 5.00 | 0.00 | 89.31 | 46.00 | 0.94 | 0.05 | 0.448 |
| VC_A0426 | 10.00 | 0.00 | 152.26 | 233.00 | -0.62 | 0.13 | 0.679 |
| VC_A0425 | 12.84 | 0.37 | 181.82 | 199.00 | -0.14 | 0.09 | 0.057 |
| VC_A0424 | 9.77 | 0.45 | 122.99 | 53.00 | 1.21 | 0.03 | 0.111 |
| VC_A0423 | 8.92 | 0.27 | 214.49 | 90.00 | 1.25 | 0.03 | 0.778 |
| VC_A0422 | 8.91 | 0.29 | 116.54 | 58.00 | 0.99 | 0.05 | 0.219 |
| VC_A0421 | 10.64 | 0.56 | 54.28 | 14.00 | 1.93 | 0.04 | 0.065 |
| VC_A0420 | 10.00 | 0.00 | 79.39 | 42.00 | 0.90 | 0.06 | 0.746 |
| VC_A0419 | 40.22 | 0.87 | 369.98 | 640.00 | -0.80 | 0.10 | 0.252 |
| VC_A0418 | 2.00 | 0.00 | 5.56 | 2.00 | 1.20 | 0.21 | 1.000 |
| VC_A0417 | 23.86 | 0.35 | 98.15 | 207.00 | -1.09 | 0.23 | 0.170 |
| VC_A0416 | 7.97 | 0.17 | 60.10 | 68.00 | -0.20 | 0.14 | 0.376 |
| VC_A0415 | 7.85 | 0.36 | 65.89 | 99.00 | -0.62 | 0.23 | 0.806 |
| VC_A0414 | 12.96 | 0.20 | 168.57 | 215.00 | -0.36 | 0.09 | 0.387 |
| VC_A0413 | 18.70 | 0.50 | 217.67 | 202.00 | 0.10 | 0.07 | 0.720 |
| VC_A0412 | 4.00 | 0.00 | 54.19 | 9.00 | 2.56 | 0.02 | 0.131 |
| VC_A0411 | 1.00 | 0.00 | 9.91 | 20.00 | -1.19 | 0.99 | 1.000 |
| VC_A0410 | 24.88 | 0.33 | 507.74 | 499.00 | 0.02 | 0.05 | 0.885 |
| VC_A0409 | 16.59 | 0.71 | 153.15 | 35.00 | 2.12 | 0.02 | 0.058 |
| VC_A0408 | 16.62 | 0.65 | 28.44 | 176.00 | -2.69 | 1.32 | 0.016 |
| VC_A0407 | 3.00 | 0.00 | 10.58 | 14.00 | -0.54 | 0.51 | 0.969 |
| VC_A0406 | 19.75 | 0.46 | 180.24 | 232.00 | -0.37 | 0.09 | 0.302 |
| VC_A0405 | 21.19 | 0.83 | 108.99 | 170.00 | -0.65 | 0.14 | 0.212 |
| VC_A0404 | 1.00 | 0.00 | 36.97 | 10.00 | 1.85 | 0.05 | 1.000 |
| VC_A0403 | 6.00 | 0.00 | 61.18 | 83.00 | -0.46 | 0.19 | 0.832 |

| | | | | | | | |
|---|---|---|---|---|---|---|---|
| VC_A0402 | 20.66 | 0.54 | 180.55 | 248.00 | -0.47 | 0.10 | 0.674 |
| VC_A0401 | 15.92 | 0.27 | 97.70 | 95.00 | 0.02 | 0.11 | 0.414 |
| VC_A0400 | 12.74 | 0.46 | 72.54 | 119.00 | -0.74 | 0.21 | 0.722 |
| VC_A0399 | 29.56 | 0.67 | 590.49 | 407.00 | 0.53 | 0.03 | 0.059 |
| VC_A0398 | 1.00 | 0.00 | 27.23 | 8.00 | 1.70 | 0.07 | 1.000 |
| VC_A0397 | 20.54 | 0.64 | 225.37 | 73.00 | 1.62 | 0.02 | 0.147 |
| VC_A0396 | 27.62 | 0.55 | 281.74 | 326.00 | -0.22 | 0.07 | 0.463 |
| VC_A0395 | 26.64 | 0.54 | 250.80 | 360.00 | -0.53 | 0.09 | 0.253 |
| VC_A0394 | 0.00 | 0.00 | 1.00 | 1.00 | #VALEUR! | NaN | NaN |
| VC_A0393 | 2.95 | 0.22 | 13.86 | 3.00 | 2.09 | 0.08 | 0.198 |
| VC_A0392 | 1.00 | 0.00 | 1.00 | 10.00 | -3.32 | 0.00 | 1.000 |
| VC_A0391 | 11.68 | 0.51 | 129.83 | 188.00 | -0.55 | 0.15 | 0.348 |
| VC_A0390 | 1.00 | 0.00 | 1.00 | 20.00 | -4.32 | 0.00 | 1.000 |
| VC_A0389 | 6.00 | 0.00 | 97.64 | 153.00 | -0.66 | 0.16 | 0.339 |
| VC_A0388 | 23.84 | 0.37 | 259.55 | 104.00 | 1.31 | 0.02 | 0.110 |
| VC_A0387 | 18.94 | 0.24 | 299.13 | 424.00 | -0.51 | 0.08 | 0.810 |
| VC_A0386 | 8.00 | 0.00 | 162.59 | 80.00 | 1.01 | 0.04 | 0.157 |
| VC_A0385 | 11.00 | 0.00 | 119.15 | 113.00 | 0.06 | 0.08 | 0.816 |
| VC_A0384 | 0.00 | 0.00 | 1.00 | 1.00 | #VALEUR! | NaN | NaN |
| VC_A0383 | 0.00 | 0.00 | 1.00 | 1.00 | #VALEUR! | NaN | NaN |
| VC_A0382 | 10.00 | 0.00 | 154.77 | 218.00 | -0.50 | 0.12 | 0.676 |
| VC_A0381 | 3.00 | 0.00 | 31.74 | 9.00 | 1.78 | 0.05 | 0.388 |
| VC_A0380 | 16.86 | 0.35 | 196.50 | 109.00 | 0.84 | 0.04 | 0.646 |
| VC_A0379 | 22.85 | 0.36 | 183.14 | 171.00 | 0.09 | 0.08 | 0.354 |
| VC_A0378 | 3.00 | 0.00 | 21.41 | 14.00 | 0.54 | 0.15 | 0.656 |
| VC_A0377 | 2.62 | 0.58 | 6.73 | 1.00 | 2.47 | 0.09 | 0.210 |
| VC_A0376 | 19.95 | 0.22 | 232.33 | 184.00 | 0.33 | 0.05 | 0.938 |
| VC_A0375 | 9.88 | 0.33 | 146.74 | 160.00 | -0.13 | 0.07 | 0.427 |
| VC_A0374 | 34.34 | 0.86 | 471.93 | 215.00 | 1.13 | 0.02 | 0.000 |
| VC_A0373 | 3.80 | 0.40 | 22.96 | 3.00 | 2.86 | 0.04 | 0.100 |
| VC_A0372 | 16.90 | 0.30 | 221.17 | 139.00 | 0.66 | 0.05 | 0.536 |
| VC_A0371 | 42.63 | 0.58 | 564.31 | 568.00 | -0.01 | 0.04 | 0.750 |
| VC_A0370 | 12.77 | 0.45 | 101.89 | 49.00 | 1.04 | 0.05 | 0.324 |
| VC_A0369 | 24.84 | 0.37 | 214.81 | 211.00 | 0.02 | 0.07 | 0.849 |
| VC_A0368 | 21.49 | 0.64 | 211.32 | 175.00 | 0.27 | 0.06 | 0.751 |
| VC_A0367 | 33.15 | 0.82 | 227.47 | 268.00 | -0.24 | 0.08 | 0.211 |
| VC_A0366 | 30.68 | 0.53 | 252.17 | 153.00 | 0.71 | 0.04 | 0.064 |
| VC_A0365 | 14.72 | 0.51 | 176.36 | 275.00 | -0.65 | 0.12 | 0.413 |
| VC_A0364 | 12.73 | 0.45 | 92.01 | 114.00 | -0.33 | 0.15 | 0.392 |
| VC_A0363 | 10.82 | 0.39 | 44.91 | 13.00 | 1.76 | 0.04 | 0.753 |

| | | | | | | | |
|---|---|---|---|---|---|---|---|
| VC_A0362 | 3.95 | 0.22 | 77.52 | 5.00 | 3.94 | 0.01 | 0.239 |
| VC_A0361 | 14.83 | 0.38 | 179.24 | 153.00 | 0.22 | 0.07 | 0.539 |
| VC_A0360 | 6.88 | 0.33 | 54.15 | 74.00 | -0.48 | 0.20 | 0.916 |
| VC_A0359 | 6.86 | 0.35 | 46.14 | 40.00 | 0.17 | 0.14 | 0.896 |
| VC_A0358 | 10.00 | 0.00 | 52.63 | 38.00 | 0.44 | 0.11 | 0.820 |
| VC_A0357 | 2.00 | 0.00 | 71.20 | 12.00 | 2.55 | 0.02 | 0.337 |
| VC_A0356 | 36.62 | 0.56 | 340.22 | 385.00 | -0.18 | 0.06 | 0.109 |
| VC_A0355 | 33.97 | 0.17 | 507.92 | 422.00 | 0.26 | 0.04 | 0.893 |
| VC_A0354 | 23.37 | 0.77 | 394.77 | 292.00 | 0.43 | 0.03 | 0.899 |
| VC_A0353 | 3.00 | 0.00 | 21.43 | 7.00 | 1.54 | 0.08 | 0.519 |
| VC_A0352 | 1.00 | 0.00 | 22.73 | 1.00 | 4.43 | 0.01 | 1.000 |
| VC_A0351 | 27.73 | 0.45 | 337.63 | 254.00 | 0.41 | 0.04 | 0.575 |
| VC_A0350 | 20.69 | 0.46 | 168.15 | 209.00 | -0.32 | 0.10 | 0.460 |
| VC_A0349 | 7.00 | 0.00 | 91.18 | 59.00 | 0.61 | 0.06 | 0.757 |
| VC_A0348 | 9.97 | 0.17 | 108.06 | 82.00 | 0.38 | 0.08 | 0.328 |
| VC_A0347 | 11.92 | 0.27 | 61.57 | 50.00 | 0.28 | 0.10 | 0.807 |
| VC_A0346 | 16.91 | 0.32 | 191.43 | 168.00 | 0.18 | 0.06 | 0.417 |
| VC_A0345 | 26.93 | 0.26 | 227.59 | 227.00 | 0.00 | 0.06 | 0.922 |
| VC_A0344 | 18.91 | 0.29 | 90.49 | 86.00 | 0.06 | 0.11 | 0.874 |
| VC_A0343 | 9.71 | 0.50 | 21.61 | 69.00 | -1.75 | 0.80 | 0.080 |
| VC_A0342 | 11.70 | 0.48 | 202.87 | 72.00 | 1.49 | 0.02 | 0.058 |
| VC_A0341 | 10.00 | 0.00 | 54.87 | 69.00 | -0.35 | 0.15 | 0.564 |
| VC_A0340 | 24.00 | 0.00 | 212.17 | 456.00 | -1.11 | 0.16 | 0.066 |
| VC_A0339 | 5.00 | 0.00 | 143.61 | 209.00 | -0.55 | 0.13 | 0.952 |
| VC_A0338 | 8.00 | 0.00 | 47.14 | 133.00 | -1.52 | 0.38 | 0.066 |
| VC_A0337 | 45.89 | 0.31 | 376.89 | 418.00 | -0.15 | 0.06 | 0.261 |
| VC_A0336 | 2.00 | 0.00 | 17.09 | 3.00 | 2.41 | 0.05 | 0.337 |
| VC_A0335 | 1.00 | 0.00 | 36.33 | 1.00 | 5.15 | 0.00 | 1.000 |
| VC_A0334 | 12.68 | 0.53 | 18.34 | 52.00 | -1.57 | 0.63 | 0.076 |
| VC_A0333 | 0.00 | 0.00 | 1.00 | 1.00 | #VALEUR! | NaN | NaN |
| VC_A0332 | 6.86 | 0.35 | 17.56 | 75.00 | -2.19 | 1.29 | 0.281 |
| VC_A0331 | 36.75 | 0.52 | 331.22 | 413.00 | -0.32 | 0.07 | 0.625 |
| VC_A0330 | 9.96 | 0.20 | 130.31 | 35.00 | 1.88 | 0.03 | 0.091 |
| VC_A0329 | 8.95 | 0.22 | 21.19 | 71.00 | -1.84 | 1.08 | 0.165 |
| VC_A0328 | 10.78 | 0.48 | 52.00 | 64.00 | -0.33 | 0.18 | 0.655 |
| VC_A0327 | 3.00 | 0.00 | 13.52 | 48.00 | -1.93 | 1.09 | 0.143 |
| VC_A0326 | 4.99 | 0.10 | 20.54 | 7.00 | 1.47 | 0.10 | 0.394 |
| VC_A0325 | 11.90 | 0.30 | 446.51 | 265.00 | 0.75 | 0.03 | 0.348 |
| VC_A0324 | 2.00 | 0.00 | 11.09 | 15.00 | -0.61 | 0.76 | 1.000 |
| VC_A0323 | 15.79 | 0.50 | 192.80 | 139.00 | 0.47 | 0.05 | 0.919 |

| | | | | | | | |
|---|---|---|---|---|---|---|---|
| VC_A0322 | 19.97 | 0.17 | 239.75 | 187.00 | 0.35 | 0.06 | 0.148 |
| VC_A0321 | 2.00 | 0.00 | 1.00 | 19.00 | -4.25 | 0.00 | 0.333 |
| VC_A0320 | 3.00 | 0.00 | 7.50 | 26.00 | -2.03 | 2.00 | 0.236 |
| VC_A0319 | 11.89 | 0.31 | 173.66 | 67.00 | 1.37 | 0.03 | 0.339 |
| VC_A0318 | 15.98 | 0.14 | 119.39 | 117.00 | 0.02 | 0.09 | 0.663 |
| VC_A0317 | 21.98 | 0.14 | 100.95 | 238.00 | -1.25 | 0.23 | 0.009 |
| VC_A0316 | 23.70 | 0.48 | 130.27 | 226.00 | -0.81 | 0.15 | 0.276 |
| VC_A0315 | 5.85 | 0.36 | 55.94 | 78.00 | -0.50 | 0.16 | 0.603 |
| VC_A0314 | 12.86 | 0.35 | 202.87 | 273.00 | -0.44 | 0.10 | 0.867 |
| VC_A0313 | 10.00 | 0.00 | 176.58 | 210.00 | -0.26 | 0.10 | 0.429 |
| VC_A0312 | 7.78 | 0.42 | 140.51 | 78.00 | 0.84 | 0.05 | 0.390 |
| VC_A0311 | 13.84 | 0.37 | 105.32 | 125.00 | -0.26 | 0.12 | 0.722 |
| VC_A0310 | 11.89 | 0.31 | 179.36 | 150.00 | 0.25 | 0.06 | 0.901 |
| VC_A0309 | 25.00 | 0.00 | 330.75 | 296.00 | 0.15 | 0.06 | 0.824 |
| VC_A0308 | 55.36 | 0.72 | 602.85 | 747.00 | -0.31 | 0.05 | 0.649 |
| VC_A0307 | 34.17 | 0.91 | 382.16 | 355.00 | 0.10 | 0.05 | 0.691 |
| VC_A0306 | 2.00 | 0.00 | 2.18 | 8.00 | -2.32 | 2.54 | 0.467 |
| VC_A0305 | 0.00 | 0.00 | 1.00 | 1.00 | #VALEUR! | NaN | NaN |
| VC_A0304 | 2.00 | 0.00 | 12.98 | 24.00 | -1.06 | 0.91 | 1.000 |
| VC_A0303 | 22.52 | 0.76 | 112.46 | 92.00 | 0.28 | 0.08 | 0.295 |
| VC_A0302 | 3.00 | 0.00 | 7.16 | 87.00 | -3.98 | 11.03 | 0.318 |
| VC_A0301 | 22.70 | 0.54 | 292.40 | 403.00 | -0.47 | 0.09 | 0.535 |
| VC_A0300 | 24.90 | 0.30 | 371.84 | 409.00 | -0.14 | 0.06 | 0.351 |
| VC_A0299 | 21.83 | 0.40 | 453.61 | 716.00 | -0.66 | 0.08 | 0.811 |
| VC_A0298 | 9.89 | 0.31 | 14.52 | 74.00 | -2.45 | 1.54 | 0.015 |
| VC_A0297 | 3.00 | 0.00 | 17.95 | 8.00 | 1.07 | 0.13 | 0.636 |
| VC_A0296 | 1.00 | 0.00 | 39.53 | 1.00 | 5.26 | 0.00 | 1.000 |
| VC_A0295 | 2.80 | 0.43 | 11.64 | 1.00 | 3.40 | 0.03 | 0.151 |
| VC_A0294 | 8.00 | 0.00 | 64.23 | 59.00 | 0.09 | 0.13 | 0.585 |
| VC_A0293 | 25.33 | 0.82 | 242.84 | 333.00 | -0.46 | 0.10 | 0.796 |
| VC_A0292 | 11.89 | 0.31 | 72.97 | 94.00 | -0.39 | 0.16 | 0.468 |
| VC_A0291 | 29.60 | 0.59 | 485.34 | 349.00 | 0.47 | 0.03 | 0.905 |
| VC_A0290 | 0.00 | 0.00 | 1.00 | 1.00 | #VALEUR! | NaN | NaN |
| VC_A0289 | 0.00 | 0.00 | 1.00 | 1.00 | #VALEUR! | NaN | NaN |
| VC_A0288 | 0.00 | 0.00 | 1.00 | 1.00 | #VALEUR! | NaN | NaN |
| VC_A0287 | 0.00 | 0.00 | 1.00 | 1.00 | #VALEUR! | NaN | NaN |
| VC_A0286 | 16.69 | 0.46 | 132.81 | 203.00 | -0.63 | 0.15 | 0.888 |
| VC_A0285 | 30.21 | 0.78 | 434.42 | 421.00 | 0.04 | 0.05 | 0.007 |
| VC_A0284 | 22.60 | 0.60 | 369.01 | 339.00 | 0.12 | 0.05 | 0.686 |
| VC_A0283 | 41.98 | 0.92 | 407.45 | 396.00 | 0.04 | 0.05 | 0.232 |

| | | | | | | | |
|---|---|---|---|---|---|---|---|
| VC_A0282 | 19.79 | 0.41 | 318.49 | 454.00 | -0.52 | 0.08 | 0.584 |
| VC_A0281 | 26.64 | 0.52 | 287.52 | 634.00 | -1.15 | 0.13 | 0.089 |
| VC_A0280 | 14.51 | 0.54 | 229.48 | 288.00 | -0.33 | 0.08 | 0.100 |
| VC_A0279 | 9.36 | 0.67 | 72.70 | 112.00 | -0.64 | 0.18 | 0.479 |
| VC_A0278 | 26.84 | 0.39 | 396.15 | 300.00 | 0.40 | 0.04 | 0.382 |
| VC_A0277 | 9.67 | 0.51 | 86.84 | 83.00 | 0.05 | 0.11 | 0.610 |
| VC_A0276 | 41.29 | 0.73 | 533.07 | 551.00 | -0.05 | 0.05 | 0.468 |
| VC_A0275 | 24.00 | 0.00 | 126.18 | 165.00 | -0.40 | 0.10 | 0.093 |
| VC_A0274 | 18.78 | 0.42 | 95.61 | 187.00 | -0.98 | 0.21 | 0.224 |
| VC_A0273 | 9.93 | 0.26 | 48.63 | 126.00 | -1.40 | 0.40 | 0.646 |
| VC_A0272 | 9.56 | 0.50 | 88.00 | 123.00 | -0.50 | 0.15 | 0.801 |
| VC_A0271 | 9.52 | 0.58 | 122.97 | 323.00 | -1.41 | 0.26 | 0.721 |
| VC_A0270 | 22.86 | 0.35 | 193.26 | 253.00 | -0.40 | 0.09 | 0.325 |
| VC_A0269 | 38.36 | 0.63 | 329.81 | 255.00 | 0.37 | 0.05 | 0.591 |
| VC_A0268 | 47.28 | 0.71 | 569.57 | 594.00 | -0.06 | 0.04 | 0.724 |
| VC_A0267 | 45.49 | 0.59 | 284.61 | 623.00 | -1.13 | 0.11 | 0.000 |
| VC_A0266 | 19.24 | 0.67 | 118.69 | 120.00 | -0.03 | 0.09 | 0.865 |
| VC_A0265 | 10.88 | 0.33 | 144.66 | 93.00 | 0.63 | 0.06 | 0.228 |
| VC_A0264 | 27.04 | 0.78 | 139.95 | 213.00 | -0.62 | 0.14 | 0.523 |
| VC_A0263 | 9.19 | 0.69 | 38.32 | 12.00 | 1.64 | 0.05 | 0.112 |
| VC_A0262 | 10.69 | 0.46 | 294.74 | 121.00 | 1.28 | 0.02 | 0.043 |
| VC_A0261 | 3.00 | 0.00 | 21.41 | 59.00 | -1.53 | 0.65 | 0.768 |
| VC_A0260 | 1.00 | 0.00 | 31.59 | 14.00 | 1.13 | 0.08 | 1.000 |
| VC_A0259 | 5.00 | 0.00 | 23.50 | 24.00 | -0.09 | 0.23 | 0.864 |
| VC_A0258 | 11.67 | 0.47 | 52.36 | 128.00 | -1.32 | 0.36 | 0.071 |
| VC_A0257 | 26.66 | 0.50 | 377.10 | 339.00 | 0.15 | 0.05 | 0.877 |
| VC_A0256 | 7.98 | 0.14 | 81.90 | 133.00 | -0.71 | 0.17 | 0.901 |
| VC_A0255 | 7.71 | 0.50 | 65.81 | 11.00 | 2.56 | 0.02 | 0.035 |
| VC_A0254 | 32.35 | 0.99 | 316.48 | 288.00 | 0.13 | 0.05 | 0.537 |
| VC_A0253 | 23.55 | 0.54 | 180.93 | 175.00 | 0.04 | 0.09 | 0.489 |
| VC_A0252 | 2.00 | 0.00 | 13.59 | 7.00 | 0.82 | 0.20 | 1.000 |
| VC_A0251 | 6.00 | 0.00 | 84.24 | 29.00 | 1.53 | 0.03 | 0.265 |
| VC_A0250 | 52.32 | 0.68 | 864.60 | 399.00 | 1.11 | 0.02 | 0.001 |
| VC_A0249 | 15.00 | 0.00 | 267.62 | 220.00 | 0.28 | 0.05 | 0.428 |
| VC_A0248 | 18.60 | 0.49 | 176.07 | 382.00 | -1.12 | 0.16 | 0.382 |
| VC_A0247 | 13.00 | 0.00 | 234.40 | 81.00 | 1.53 | 0.02 | 0.369 |
| VC_A0246 | 30.51 | 0.93 | 708.14 | 429.00 | 0.72 | 0.02 | 0.116 |
| VC_A0245 | 7.94 | 0.24 | 139.80 | 51.00 | 1.45 | 0.03 | 0.209 |
| VC_A0244 | 12.68 | 0.51 | 86.99 | 173.00 | -1.01 | 0.23 | 0.035 |
| VC_A0243 | 14.93 | 0.86 | 230.27 | 311.00 | -0.44 | 0.10 | 0.540 |

| | | | | | | | |
|---|---|---|---|---|---|---|---|
| VC_A0242 | 8.84 | 0.37 | 46.87 | 98.00 | -1.10 | 0.35 | 0.268 |
| VC_A0241 | 14.86 | 0.38 | 142.83 | 158.00 | -0.16 | 0.09 | 0.435 |
| VC_A0240 | 10.45 | 0.63 | 63.19 | 47.00 | 0.40 | 0.10 | 0.591 |
| VC_A0239 | 15.97 | 0.17 | 134.10 | 104.00 | 0.36 | 0.07 | 0.260 |
| VC_A0238 | 37.80 | 0.45 | 173.11 | 248.00 | -0.53 | 0.12 | 0.167 |
| VC_A0237 | 18.46 | 0.66 | 158.04 | 234.00 | -0.58 | 0.12 | 0.764 |
| VC_A0236 | 14.88 | 0.33 | 59.25 | 185.00 | -1.67 | 0.42 | 0.347 |
| VC_A0235 | 20.65 | 0.52 | 233.18 | 266.00 | -0.20 | 0.07 | 0.844 |
| VC_A0234 | 2.00 | 0.00 | 42.29 | 4.00 | 3.35 | 0.02 | 0.333 |
| VC_A0233 | 6.00 | 0.00 | 49.00 | 7.00 | 2.78 | 0.02 | 0.108 |
| VC_A0232 | 56.04 | 0.83 | 863.42 | 717.00 | 0.27 | 0.03 | 0.404 |
| VC_A0231 | 23.13 | 0.72 | 265.09 | 141.00 | 0.91 | 0.03 | 0.242 |
| VC_A0230 | 15.71 | 0.50 | 172.28 | 173.00 | -0.01 | 0.08 | 0.332 |
| VC_A0229 | 22.97 | 0.17 | 127.90 | 430.00 | -1.76 | 0.32 | 0.313 |
| VC_A0228 | 24.19 | 0.72 | 181.23 | 69.00 | 1.38 | 0.03 | 0.530 |
| VC_A0227 | 26.52 | 0.61 | 175.25 | 251.00 | -0.53 | 0.11 | 0.829 |
| VC_A0226 | 6.81 | 0.42 | 17.60 | 29.00 | -0.80 | 0.44 | 0.929 |
| VC_A0225 | 25.56 | 0.54 | 419.55 | 512.00 | -0.29 | 0.06 | 0.685 |
| VC_A0224 | 7.99 | 0.10 | 39.72 | 18.00 | 1.09 | 0.09 | 0.727 |
| VC_A0223 | 60.97 | 1.23 | 780.83 | 509.00 | 0.62 | 0.02 | 0.885 |
| VC_A0222 | 22.00 | 0.00 | 236.83 | 222.00 | 0.09 | 0.07 | 0.899 |
| VC_A0221 | 23.94 | 0.24 | 241.41 | 188.00 | 0.35 | 0.06 | 0.122 |
| VC_A0220 | 29.87 | 0.34 | 351.15 | 404.00 | -0.21 | 0.06 | 0.858 |
| VC_A0219 | 60.78 | 0.80 | 554.72 | 964.00 | -0.80 | 0.07 | 0.298 |
| VC_A0218 | 46.96 | 0.80 | 629.60 | 611.00 | 0.04 | 0.04 | 0.517 |
| VC_A0217 | 24.57 | 0.56 | 277.50 | 322.00 | -0.22 | 0.08 | 0.846 |
| VC_A0216 | 7.97 | 0.17 | 207.66 | 181.00 | 0.19 | 0.06 | 0.802 |
| VC_A0215 | 3.18 | 0.67 | 4.67 | 15.00 | -2.05 | 2.44 | 0.830 |
| VC_A0214 | 36.53 | 0.61 | 371.46 | 541.00 | -0.55 | 0.08 | 0.106 |
| VC_A0213 | 35.33 | 0.70 | 240.81 | 460.00 | -0.94 | 0.12 | 0.170 |
| VC_A0212 | 27.76 | 0.97 | 289.29 | 204.00 | 0.50 | 0.04 | 0.179 |
| VC_A0211 | 52.19 | 0.65 | 580.26 | 510.00 | 0.18 | 0.03 | 0.226 |
| VC_A0210 | 39.90 | 0.30 | 581.10 | 398.00 | 0.54 | 0.03 | 0.316 |
| VC_A0209 | 4.00 | 0.00 | 16.31 | 26.00 | -0.79 | 0.57 | 0.435 |
| VC_A0208 | 0.00 | 0.00 | 1.00 | 1.00 | #VALEUR! | NaN | NaN |
| VC_A0207 | 0.00 | 0.00 | 1.00 | 1.00 | #VALEUR! | NaN | NaN |
| VC_A0206 | 2.00 | 0.00 | 11.43 | 38.00 | -1.94 | 1.65 | 0.463 |
| VC_A0205 | 29.77 | 1.00 | 382.22 | 263.00 | 0.54 | 0.04 | 0.392 |
| VC_A0204 | 26.48 | 0.67 | 302.58 | 244.00 | 0.31 | 0.04 | 0.879 |
| VC_A0203 | 13.58 | 0.50 | 45.96 | 93.00 | -1.06 | 0.34 | 0.196 |

| | | | | | | | |
|---|---|---|---|---|---|---|---|
| VC_A0202 | 19.53 | 0.61 | 134.29 | 118.00 | 0.18 | 0.07 | 0.582 |
| VC_A0201 | 14.45 | 0.93 | 33.91 | 28.00 | 0.24 | 0.15 | 0.524 |
| VC_A0200 | 33.36 | 1.07 | 350.84 | 125.00 | 1.48 | 0.02 | 0.053 |
| VC_A0199 | 40.78 | 1.27 | 184.92 | 164.00 | 0.17 | 0.06 | 0.356 |
| VC_A0198 | 26.00 | 1.04 | 44.31 | 109.00 | -1.33 | 0.39 | 0.143 |
| VC_A0197 | 25.18 | 0.77 | 461.47 | 268.00 | 0.78 | 0.03 | 0.369 |
| VC_A0196 | 9.42 | 0.68 | 69.77 | 113.00 | -0.72 | 0.19 | 0.059 |
| VC_A0195 | 15.25 | 0.69 | 141.32 | 41.00 | 1.78 | 0.02 | 0.178 |
| VC_A0194 | 19.68 | 0.47 | 254.99 | 305.00 | -0.26 | 0.08 | 0.545 |
| VC_A0193 | 22.52 | 0.54 | 256.39 | 228.00 | 0.16 | 0.06 | 0.376 |
| VC_A0192 | 14.74 | 0.44 | 154.08 | 146.00 | 0.06 | 0.10 | 0.446 |
| VC_A0191 | 39.83 | 0.38 | 467.58 | 501.00 | -0.10 | 0.05 | 0.277 |
| VC_A0190 | 9.99 | 0.10 | 97.68 | 68.00 | 0.50 | 0.08 | 0.187 |
| VC_A0189 | 21.75 | 0.77 | 162.56 | 146.00 | 0.15 | 0.07 | 0.479 |
| VC_A0188 | 8.88 | 0.33 | 106.21 | 119.00 | -0.18 | 0.12 | 0.808 |
| VC_A0187 | 5.00 | 0.00 | 16.23 | 45.00 | -1.56 | 0.75 | 0.134 |
| VC_A0186 | 5.91 | 0.29 | 64.62 | 63.00 | 0.01 | 0.14 | 0.468 |
| VC_A0185 | 9.90 | 0.30 | 93.74 | 59.00 | 0.65 | 0.06 | 0.746 |
| VC_A0184 | 1.61 | 0.51 | 5.09 | 1.00 | 2.01 | 0.15 | 0.582 |
| VC_A0183 | 33.54 | 0.52 | 300.04 | 406.00 | -0.44 | 0.09 | 0.416 |
| VC_A0182 | 32.19 | 0.73 | 325.25 | 337.00 | -0.06 | 0.07 | 0.790 |
| VC_A0181 | 15.27 | 0.68 | 130.73 | 211.00 | -0.70 | 0.12 | 0.237 |
| VC_A0180 | 32.49 | 1.11 | 329.17 | 359.00 | -0.13 | 0.06 | 0.886 |
| VC_A0179 | 26.74 | 0.46 | 294.91 | 328.00 | -0.16 | 0.07 | 0.301 |
| VC_A0178 | 13.71 | 0.50 | 82.17 | 188.00 | -1.21 | 0.26 | 0.755 |
| VC_A0177 | 2.00 | 0.00 | 1.37 | 7.00 | -2.58 | 1.71 | 0.367 |
| VC_A0176 | 41.96 | 0.86 | 365.32 | 492.00 | -0.43 | 0.07 | 0.284 |
| VC_A0175 | 20.49 | 0.61 | 230.97 | 253.00 | -0.14 | 0.08 | 0.276 |
| VC_A0174 | 16.64 | 0.48 | 225.25 | 234.00 | -0.06 | 0.07 | 0.504 |
| VC_A0173 | 8.80 | 0.43 | 110.33 | 76.00 | 0.52 | 0.07 | 0.358 |
| VC_A0172 | 23.30 | 0.73 | 270.26 | 284.00 | -0.08 | 0.06 | 0.294 |
| VC_A0171 | 37.29 | 0.73 | 647.62 | 479.00 | 0.43 | 0.03 | 0.103 |
| VC_A0170 | 11.72 | 0.49 | 39.85 | 153.00 | -1.98 | 0.72 | 0.840 |
| VC_A0169 | 13.57 | 0.50 | 166.73 | 112.00 | 0.56 | 0.06 | 0.400 |
| VC_A0168 | 11.97 | 0.17 | 165.83 | 187.00 | -0.18 | 0.09 | 0.810 |
| VC_A0167 | 18.60 | 0.57 | 232.81 | 84.00 | 1.46 | 0.03 | 0.416 |
| VC_A0166 | 1.59 | 0.49 | 1.46 | 7.00 | -2.53 | 1.87 | 1.000 |
| VC_A0165 | 57.96 | 0.91 | 699.03 | 600.00 | 0.22 | 0.03 | 0.407 |
| VC_A0164 | 21.22 | 0.81 | 183.61 | 233.00 | -0.35 | 0.09 | 0.592 |
| VC_A0163 | 4.00 | 0.00 | 7.56 | 4.00 | 0.67 | 0.31 | 0.853 |

| | | | | | | | |
|---|---|---|---|---|---|---|---|
| VC_A0162 | 3.94 | 0.24 | 6.60 | 7.00 | -0.27 | 0.48 | 0.955 |
| VC_A0161.1 | 1.00 | 0.00 | 1.00 | 6.00 | -2.58 | 0.00 | 1.000 |
| VC_A0161 | 38.28 | 0.74 | 320.26 | 334.00 | -0.07 | 0.06 | 0.681 |
| VC_A0160 | 32.50 | 1.16 | 338.36 | 238.00 | 0.50 | 0.04 | 0.665 |
| VC_A0159 | 26.24 | 0.75 | 226.18 | 209.00 | 0.11 | 0.06 | 0.835 |
| VC_A0158 | 4.00 | 0.00 | 88.98 | 29.00 | 1.60 | 0.03 | 0.249 |
| VC_A0157 | 71.01 | 1.10 | 704.56 | 746.00 | -0.08 | 0.04 | 0.261 |
| VC_A0156 | 8.00 | 0.00 | 86.05 | 97.00 | -0.19 | 0.14 | 0.904 |
| VC_A0155 | 31.92 | 0.81 | 234.69 | 369.00 | -0.66 | 0.12 | 0.307 |
| VC_A0154 | 7.00 | 0.00 | 64.16 | 48.00 | 0.39 | 0.10 | 0.613 |
| VC_A0153 | 13.67 | 0.47 | 90.03 | 108.00 | -0.28 | 0.13 | 0.743 |
| VC_A0152 | 12.23 | 0.74 | 89.27 | 209.00 | -1.24 | 0.24 | 0.185 |
| VC_A0151 | 33.16 | 0.79 | 380.47 | 341.00 | 0.15 | 0.05 | 0.831 |
| VC_A0150 | 15.72 | 0.47 | 149.15 | 292.00 | -0.98 | 0.16 | 0.612 |
| VC_A0149 | 1.00 | 0.00 | 32.90 | 1.00 | 5.00 | 0.01 | 1.000 |
| VC_A0148 | 99.75 | 1.02 | 1517.96 | 1067.00 | 0.51 | 0.02 | 0.227 |
| VC_A0147 | 27.90 | 0.30 | 260.45 | 299.00 | -0.21 | 0.08 | 0.564 |
| VC_A0146 | 45.51 | 0.56 | 518.88 | 500.00 | 0.05 | 0.04 | 0.909 |
| VC_A0145 | 0.00 | 0.00 | 1.00 | 1.00 | #VALEUR! | NaN | NaN |
| VC_A0144 | 23.22 | 0.73 | 209.85 | 358.00 | -0.78 | 0.12 | 0.250 |
| VC_A0143 | 2.00 | 0.00 | 32.62 | 1.00 | 4.99 | 0.01 | 0.333 |
| VC_A0142 | 14.64 | 0.48 | 85.86 | 240.00 | -1.50 | 0.35 | 0.040 |
| VC_A0141 | 39.66 | 1.02 | 376.22 | 331.00 | 0.18 | 0.05 | 0.235 |
| VC_A0140 | 31.15 | 0.74 | 330.06 | 288.00 | 0.19 | 0.05 | 0.177 |
| VC_A0139 | 12.00 | 0.00 | 49.81 | 214.00 | -2.13 | 0.63 | 0.084 |
| VC_A0138 | 2.89 | 0.31 | 21.34 | 3.00 | 2.76 | 0.03 | 0.197 |
| VC_A0137 | 36.19 | 0.69 | 452.48 | 300.00 | 0.59 | 0.03 | 0.107 |
| VC_A0136 | 31.35 | 0.69 | 389.02 | 227.00 | 0.77 | 0.03 | 0.356 |
| VC_A0135 | 11.84 | 0.39 | 59.17 | 113.00 | -0.96 | 0.28 | 0.488 |
| VC_A0134 | 11.63 | 0.49 | 132.69 | 94.00 | 0.49 | 0.06 | 0.708 |
| VC_A0133 | 17.99 | 0.10 | 207.14 | 109.00 | 0.92 | 0.04 | 0.407 |
| VC_A0132 | 22.17 | 0.75 | 75.36 | 197.00 | -1.41 | 0.37 | 0.198 |
| VC_A0131 | 22.00 | 0.84 | 183.42 | 206.00 | -0.17 | 0.08 | 0.869 |
| VC_A0130 | 11.26 | 0.65 | 230.09 | 126.00 | 0.86 | 0.03 | 0.468 |
| VC_A0129 | 26.23 | 0.63 | 233.93 | 289.00 | -0.31 | 0.08 | 0.112 |
| VC_A0128 | 19.86 | 0.35 | 184.29 | 139.00 | 0.40 | 0.06 | 0.355 |
| VC_A0127 | 10.64 | 0.48 | 296.63 | 304.00 | -0.04 | 0.06 | 0.549 |
| VC_A0126 | 7.97 | 0.17 | 59.41 | 32.00 | 0.87 | 0.08 | 0.806 |
| VC_A0125 | 8.00 | 0.00 | 70.43 | 24.00 | 1.53 | 0.04 | 0.883 |
| VC_A0124 | 10.36 | 1.14 | 36.61 | 21.00 | 0.75 | 0.11 | 0.274 |

| | | | | | | | |
|---|---|---|---|---|---|---|---|
| VC_A0123 | 81.48 | 1.53 | 733.32 | 728.00 | 0.01 | 0.04 | 0.474 |
| VC_A0122 | 6.91 | 0.29 | 70.25 | 83.00 | -0.26 | 0.14 | 0.812 |
| VC_A0121 | 29.40 | 1.09 | 172.26 | 165.00 | 0.05 | 0.08 | 0.416 |
| VC_A0120 | 95.27 | 1.14 | 732.40 | 1104.00 | -0.59 | 0.06 | 0.409 |
| VC_A0119 | 16.48 | 0.58 | 169.21 | 204.00 | -0.28 | 0.09 | 0.450 |
| VC_A0118 | 7.64 | 0.48 | 96.28 | 38.00 | 1.33 | 0.04 | 0.115 |
| VC_A0117 | 30.76 | 0.97 | 488.54 | 183.00 | 1.41 | 0.02 | 0.171 |
| VC_A0116 | 53.69 | 0.93 | 449.46 | 456.00 | -0.02 | 0.05 | 0.866 |
| VC_A0115 | 17.43 | 0.66 | 341.56 | 287.00 | 0.25 | 0.05 | 0.587 |
| VC_A0114 | 32.22 | 0.72 | 319.78 | 471.00 | -0.56 | 0.08 | 0.644 |
| VC_A0113 | 15.00 | 0.00 | 104.79 | 139.00 | -0.42 | 0.13 | 0.571 |
| VC_A0112 | 35.50 | 0.63 | 600.97 | 436.00 | 0.46 | 0.03 | 0.230 |
| VC_A0111 | 15.74 | 0.48 | 95.84 | 144.00 | -0.60 | 0.16 | 0.812 |
| VC_A0110 | 41.15 | 1.18 | 502.36 | 397.00 | 0.34 | 0.04 | 0.277 |
| VC_A0109 | 11.59 | 0.49 | 87.67 | 100.00 | -0.21 | 0.13 | 0.448 |
| VC_A0108 | 35.41 | 0.55 | 526.23 | 258.00 | 1.03 | 0.02 | 0.302 |
| VC_A0107 | 10.63 | 0.49 | 87.89 | 71.00 | 0.29 | 0.09 | 0.823 |
| VC_A0106 | 23.29 | 0.73 | 95.48 | 186.00 | -0.98 | 0.24 | 0.769 |
| VC_A0105 | 6.00 | 0.00 | 7.40 | 84.00 | -3.76 | 8.55 | 0.006 |
| VC_A0104 | 39.60 | 0.64 | 519.46 | 363.00 | 0.51 | 0.03 | 0.058 |
| VC_A0103 | 34.03 | 0.76 | 483.73 | 315.00 | 0.62 | 0.03 | 0.231 |
| VC_A0102 | 18.97 | 0.17 | 159.48 | 308.00 | -0.96 | 0.14 | 0.816 |
| VC_A0101 | 45.04 | 0.79 | 487.16 | 161.00 | 1.59 | 0.01 | 0.015 |
| VC_A0100 | 5.94 | 0.24 | 51.43 | 23.00 | 1.13 | 0.07 | 0.138 |
| VC_A0099 | 24.90 | 0.78 | 251.51 | 305.00 | -0.28 | 0.07 | 0.794 |
| VC_A0098 | 24.46 | 0.67 | 251.75 | 235.00 | 0.09 | 0.06 | 0.911 |
| VC_A0097 | 8.61 | 0.57 | 29.37 | 46.00 | -0.70 | 0.30 | 0.669 |
| VC_A0096 | 38.94 | 0.24 | 549.32 | 559.00 | -0.03 | 0.04 | 0.881 |
| VC_A0095 | 11.27 | 0.68 | 26.11 | 48.00 | -0.94 | 0.41 | 0.235 |
| VC_A0094 | 9.85 | 0.36 | 35.35 | 72.00 | -1.07 | 0.39 | 0.418 |
| VC_A0093 | 5.91 | 0.29 | 26.69 | 11.00 | 1.21 | 0.10 | 0.861 |
| VC_A0092 | 22.34 | 0.67 | 296.93 | 223.00 | 0.41 | 0.05 | 0.246 |
| VC_A0091 | 5.00 | 0.00 | 5.47 | 31.00 | -2.95 | 5.00 | 0.147 |
| VC_A0090 | 21.87 | 0.34 | 145.11 | 211.00 | -0.55 | 0.13 | 0.482 |
| VC_A0089 | 1.91 | 0.29 | 8.50 | 1.00 | 2.86 | 0.07 | 0.393 |
| VC_A0088 | 23.41 | 0.65 | 145.86 | 556.00 | -1.94 | 0.29 | 0.089 |
| VC_A0087 | 5.00 | 0.00 | 13.60 | 18.00 | -0.53 | 0.45 | 0.696 |
| VC_A0086 | 8.52 | 0.64 | 82.35 | 244.00 | -1.59 | 0.35 | 0.755 |
| VC_A0085 | 37.54 | 0.56 | 288.89 | 341.00 | -0.24 | 0.07 | 0.258 |
| VC_A0084 | 11.44 | 0.62 | 53.90 | 76.00 | -0.53 | 0.22 | 0.859 |

| | | | | | | | |
|---|---|---|---|---|---|---|---|
| VC_A0083 | 38.56 | 0.56 | 456.14 | 568.00 | -0.32 | 0.06 | 0.193 |
| VC_A0082 | 20.82 | 0.39 | 141.67 | 206.00 | -0.56 | 0.16 | 0.574 |
| VC_A0081 | 4.99 | 0.10 | 34.95 | 32.00 | 0.08 | 0.17 | 0.702 |
| VC_A0080 | 67.40 | 1.01 | 567.55 | 600.00 | -0.08 | 0.04 | 0.886 |
| VC_A0079 | 24.40 | 0.95 | 142.85 | 211.00 | -0.57 | 0.11 | 0.085 |
| VC_A0078 | 7.15 | 0.70 | 51.84 | 55.00 | -0.12 | 0.16 | 0.547 |
| VC_A0077 | 41.70 | 0.94 | 451.60 | 352.00 | 0.36 | 0.03 | 0.901 |
| VC_A0076 | 31.62 | 0.91 | 384.67 | 341.00 | 0.17 | 0.04 | 0.315 |
| VC_A0075 | 17.29 | 0.69 | 283.02 | 94.00 | 1.59 | 0.02 | 0.084 |
| VC_A0074 | 29.38 | 1.04 | 226.21 | 194.00 | 0.21 | 0.06 | 0.450 |
| VC_A0073 | 15.66 | 0.48 | 306.39 | 161.00 | 0.92 | 0.03 | 0.534 |
| VC_A0072 | 17.13 | 0.75 | 184.19 | 89.00 | 1.04 | 0.04 | 0.115 |
| VC_A0071 | 25.27 | 0.75 | 569.72 | 330.00 | 0.79 | 0.02 | 0.619 |
| VC_A0070 | 27.02 | 0.89 | 156.97 | 237.00 | -0.60 | 0.12 | 0.829 |
| VC_A0069 | 3.00 | 0.00 | 3.48 | 22.00 | -3.07 | 5.17 | 0.122 |
| VC_A0068 | 35.53 | 1.09 | 384.53 | 179.00 | 1.10 | 0.03 | 0.017 |
| VC_A0067 | 16.90 | 0.30 | 414.47 | 152.00 | 1.44 | 0.02 | 0.030 |
| VC_A0066 | 31.66 | 0.48 | 303.18 | 236.00 | 0.36 | 0.05 | 0.481 |
| VC_A0065 | 61.80 | 0.91 | 592.92 | 698.00 | -0.24 | 0.05 | 0.168 |
| VC_A0064 | 49.80 | 0.88 | 640.14 | 443.00 | 0.53 | 0.03 | 0.322 |
| VC_A0063 | 48.16 | 0.71 | 374.34 | 437.00 | -0.23 | 0.06 | 0.077 |
| VC_A0062 | 3.99 | 0.10 | 44.82 | 62.00 | -0.50 | 0.23 | 0.983 |
| VC_A0061 | 4.24 | 0.78 | 19.96 | 5.00 | 1.94 | 0.06 | 0.407 |
| VC_A0060 | 9.00 | 0.00 | 132.85 | 148.00 | -0.17 | 0.10 | 0.930 |
| VC_A0059 | 15.00 | 0.00 | 104.87 | 192.00 | -0.89 | 0.18 | 0.320 |
| VC_A0058 | 27.56 | 0.61 | 182.57 | 365.00 | -1.01 | 0.15 | 0.071 |
| VC_A0057 | 32.00 | 0.80 | 384.19 | 565.00 | -0.56 | 0.08 | 0.632 |
| VC_A0056 | 11.66 | 0.54 | 159.17 | 67.00 | 1.24 | 0.03 | 0.342 |
| VC_A0055 | 24.69 | 0.56 | 248.43 | 191.00 | 0.37 | 0.04 | 0.619 |
| VC_A0054 | 4.00 | 0.00 | 13.81 | 16.00 | -0.36 | 0.46 | 0.226 |
| VC_A0053 | 17.68 | 0.47 | 487.06 | 371.00 | 0.39 | 0.03 | 0.165 |
| VC_A0052 | 44.57 | 0.59 | 640.57 | 649.00 | -0.02 | 0.04 | 0.878 |
| VC_A0051 | 25.87 | 0.34 | 223.73 | 268.00 | -0.27 | 0.08 | 0.167 |
| VC_A0050 | 22.83 | 0.38 | 383.48 | 553.00 | -0.53 | 0.07 | 0.363 |
| VC_A0049 | 20.70 | 0.46 | 186.68 | 392.00 | -1.08 | 0.16 | 0.816 |
| VC_A0048 | 8.00 | 0.00 | 42.36 | 117.00 | -1.50 | 0.48 | 0.202 |
| VC_A0047 | 29.14 | 0.86 | 220.22 | 348.00 | -0.67 | 0.10 | 0.528 |
| VC_A0046 | 3.00 | 0.00 | 30.16 | 18.00 | 0.69 | 0.12 | 0.681 |
| VC_A0044 | 85.21 | 1.13 | 1213.14 | 1080.00 | 0.17 | 0.02 | 0.092 |
| VC_A0043 | 7.89 | 0.31 | 130.99 | 67.00 | 0.96 | 0.05 | 0.296 |

| | | | | | | | |
|---|---|---|---|---|---|---|---|
| VC_A0042 | 14.65 | 0.50 | 83.35 | 232.00 | -1.50 | 0.36 | 0.050 |
| VC_A0041 | 33.84 | 0.93 | 253.58 | 262.00 | -0.05 | 0.07 | 0.565 |
| VC_A0040 | 18.92 | 0.27 | 140.91 | 166.00 | -0.25 | 0.11 | 0.600 |
| VC_A0039 | 14.94 | 0.24 | 139.75 | 113.00 | 0.30 | 0.07 | 0.900 |
| VC_A0038 | 16.77 | 0.45 | 275.67 | 147.00 | 0.90 | 0.04 | 0.141 |
| VC_A0037 | 7.99 | 0.10 | 57.44 | 81.00 | -0.52 | 0.21 | 0.471 |
| VC_A0036 | 33.18 | 0.74 | 320.37 | 398.00 | -0.32 | 0.07 | 0.826 |
| VC_A0035 | 21.31 | 0.68 | 120.80 | 221.00 | -0.88 | 0.17 | 0.208 |
| VC_A0034 | 15.62 | 0.49 | 161.96 | 98.00 | 0.72 | 0.04 | 0.857 |
| VC_A0033 | 41.52 | 0.94 | 501.88 | 693.00 | -0.47 | 0.07 | 0.719 |
| VC_A0032 | 15.82 | 0.39 | 115.72 | 116.00 | -0.01 | 0.09 | 0.756 |
| VC_A0031 | 45.25 | 0.81 | 452.96 | 470.00 | -0.06 | 0.05 | 0.864 |
| VC_A0030 | 23.00 | 0.00 | 419.51 | 432.00 | -0.05 | 0.05 | 0.251 |
| VC_A0029 | 17.68 | 0.51 | 231.01 | 177.00 | 0.38 | 0.06 | 0.376 |
| VC_A0028 | 5.95 | 0.22 | 45.34 | 71.00 | -0.68 | 0.25 | 0.581 |
| VC_A0027 | 80.62 | 0.56 | 994.94 | 1033.00 | -0.06 | 0.03 | 0.265 |
| VC_A0026 | 14.00 | 0.00 | 96.54 | 106.00 | -0.15 | 0.12 | 0.062 |
| VC_A0025 | 30.89 | 0.84 | 187.19 | 235.00 | -0.34 | 0.09 | 0.763 |
| VC_A0024 | 26.48 | 0.56 | 152.57 | 248.00 | -0.71 | 0.16 | 0.161 |
| VC_A0023 | 33.56 | 0.54 | 353.91 | 247.00 | 0.51 | 0.04 | 0.299 |
| VC_A0022 | 17.87 | 0.34 | 241.52 | 239.00 | 0.01 | 0.07 | 0.435 |
| VC_A0021 | 19.60 | 1.03 | 70.10 | 15.00 | 2.21 | 0.02 | 0.001 |
| VC_A0020 | 92.86 | 0.91 | 727.86 | 1071.00 | -0.56 | 0.05 | 0.313 |
| VC_A0019 | 20.64 | 0.50 | 148.43 | 140.00 | 0.07 | 0.10 | 0.862 |
| VC_A0018 | 47.18 | 0.88 | 599.29 | 592.00 | 0.02 | 0.04 | 0.885 |
| VC_A0017 | 10.99 | 0.10 | 139.03 | 78.00 | 0.82 | 0.05 | 0.535 |
| VC_A0016 | 51.14 | 0.74 | 406.06 | 731.00 | -0.85 | 0.09 | 0.260 |
| VC_A0015 | 2.00 | 0.00 | 58.39 | 25.00 | 1.19 | 0.06 | 0.667 |
| VC_A0014 | 42.49 | 0.59 | 431.22 | 363.00 | 0.24 | 0.04 | 0.376 |
| VC_A0013 | 38.35 | 0.73 | 516.92 | 270.00 | 0.93 | 0.02 | 0.020 |
| VC_A0012 | 1.00 | 0.00 | 1.00 | 1.00 | 0.00 | 0.00 | 1.000 |
| VC_A0011 | 57.19 | 1.03 | 466.35 | 551.00 | -0.24 | 0.05 | 0.755 |
| VC_A0010 | 10.97 | 0.17 | 130.00 | 134.00 | -0.05 | 0.08 | 0.343 |
| VC_A0009 | 10.50 | 0.64 | 115.19 | 96.00 | 0.25 | 0.08 | 0.557 |
| VC_A0008 | 46.47 | 0.54 | 469.74 | 372.00 | 0.33 | 0.04 | 0.892 |
| VC_A0007 | 17.13 | 0.73 | 277.94 | 228.00 | 0.28 | 0.05 | 0.844 |
| VC_A0006 | 8.63 | 0.49 | 60.71 | 75.00 | -0.33 | 0.17 | 0.893 |
| VC_A0005 | 11.44 | 0.59 | 120.65 | 116.00 | 0.04 | 0.11 | 0.906 |
| VC_A0004 | 5.00 | 0.00 | 153.90 | 51.00 | 1.59 | 0.02 | 0.641 |
| VC_A0003 | 14.60 | 0.57 | 114.20 | 211.00 | -0.90 | 0.19 | 0.665 |

| | | | | | | | |
|---|---|---|---|---|---|---|---|
| VC_A0002 | 7.00 | 0.00 | 49.70 | 59.00 | -0.28 | 0.20 | 0.841 |
| VC_A0001 | 4.00 | 0.00 | 9.76 | 8.00 | 0.15 | 0.33 | 0.394 |
| VC_2775 | 50.06 | 0.78 | 474.32 | 659.00 | -0.48 | 0.06 | 0.205 |
| VC_2774 | 15.00 | 0.00 | 218.47 | 218.00 | -0.01 | 0.08 | 0.903 |
| VC_2773 | 33.55 | 0.56 | 975.69 | 774.00 | 0.33 | 0.02 | 0.028 |
| VC_2772 | 17.00 | 0.00 | 372.89 | 335.00 | 0.15 | 0.05 | 0.806 |
| VC_2771 | 11.26 | 0.68 | 47.29 | 27.00 | 0.78 | 0.09 | 0.202 |
| VC_2770 | 7.78 | 0.85 | 30.94 | 1.00 | 4.89 | 0.01 | 0.000 |
| VC_2769 | 2.71 | 0.48 | 8.35 | 3.00 | 1.12 | 0.40 | 0.546 |
| VC_2768 | 3.65 | 0.50 | 26.79 | 1.00 | 4.68 | 0.01 | 0.116 |
| VC_2767 | 2.62 | 0.49 | 7.46 | 2.00 | 1.59 | 0.26 | 0.736 |
| VC_2766 | 16.15 | 1.33 | 78.06 | 14.00 | 2.46 | 0.02 | 0.000 |
| VC_2765 | 10.24 | 0.82 | 55.35 | 6.00 | 3.17 | 0.02 | 0.007 |
| VC_2764 | 16.93 | 0.87 | 91.64 | 69.00 | 0.39 | 0.08 | 0.077 |
| VC_2763 | 4.80 | 0.40 | 17.63 | 9.00 | 0.89 | 0.14 | 0.345 |
| VC_2762 | 0.00 | 0.00 | 1.00 | 1.00 | #VALEUR! | NaN | NaN |
| VC_2761 | 39.71 | 0.48 | 670.93 | 744.00 | -0.15 | 0.05 | 0.276 |
| VC_2760 | 28.50 | 0.58 | 619.58 | 834.00 | -0.43 | 0.06 | 0.763 |
| VC_2759 | 20.84 | 0.37 | 435.60 | 844.00 | -0.96 | 0.09 | 0.087 |
| VC_2758 | 55.43 | 0.62 | 1475.69 | 1429.00 | 0.05 | 0.03 | 0.136 |
| VC_2757 | 21.95 | 0.22 | 448.67 | 616.00 | -0.46 | 0.07 | 0.251 |
| VC_2756 | 54.26 | 0.76 | 1021.50 | 1207.00 | -0.24 | 0.04 | 0.890 |
| VC_2755 | 1.93 | 0.26 | 7.95 | 1.00 | 2.72 | 0.10 | 0.380 |
| VC_2754 | 2.00 | 0.00 | 1.00 | 73.00 | -6.19 | 0.00 | 0.333 |
| VC_2753 | 4.00 | 0.00 | 21.54 | 27.00 | -0.42 | 0.36 | 0.750 |
| VC_2752 | 6.00 | 0.00 | 24.47 | 41.00 | -0.79 | 0.31 | 0.366 |
| VC_2751 | 26.50 | 0.63 | 303.97 | 554.00 | -0.87 | 0.10 | 0.055 |
| VC_2750 | 98.92 | 1.19 | 2803.02 | 2598.00 | 0.11 | 0.02 | 0.538 |
| VC_2749 | 41.83 | 0.93 | 1006.13 | 671.00 | 0.58 | 0.02 | 0.064 |
| VC_2748 | 23.15 | 0.73 | 470.48 | 336.00 | 0.48 | 0.04 | 0.786 |
| VC_2747 | 14.92 | 0.27 | 568.94 | 285.00 | 0.99 | 0.02 | 0.138 |
| VC_2746 | 39.74 | 0.48 | 396.53 | 265.00 | 0.58 | 0.04 | 0.129 |
| VC_2745 | 6.62 | 0.49 | 56.33 | 74.00 | -0.42 | 0.20 | 0.854 |
| VC_2744 | 49.33 | 0.62 | 952.59 | 720.00 | 0.40 | 0.03 | 0.037 |
| VC_2743 | 16.47 | 0.59 | 582.79 | 270.00 | 1.11 | 0.02 | 0.376 |
| VC_2742 | 34.81 | 0.39 | 702.24 | 506.00 | 0.47 | 0.02 | 0.339 |
| VC_2741 | 11.96 | 0.20 | 135.64 | 160.00 | -0.25 | 0.12 | 0.643 |
| VC_2740 | 35.57 | 0.52 | 783.74 | 734.00 | 0.09 | 0.03 | 0.918 |
| VC_2739 | 54.64 | 0.52 | 835.73 | 1167.00 | -0.48 | 0.05 | 0.491 |
| VC_2738 | 45.98 | 0.14 | 1474.27 | 1044.00 | 0.50 | 0.02 | 0.556 |

| | | | | | | | |
|---|---|---|---|---|---|---|---|
| VC_2737 | 12.58 | 0.50 | 296.16 | 161.00 | 0.87 | 0.04 | 0.062 |
| VC_2736 | 26.96 | 0.20 | 549.14 | 416.00 | 0.40 | 0.03 | 0.285 |
| VC_2735 | 6.99 | 0.10 | 64.61 | 50.00 | 0.35 | 0.09 | 0.884 |
| VC_2734 | 17.96 | 0.20 | 802.72 | 600.00 | 0.42 | 0.03 | 0.446 |
| VC_2733 | 13.83 | 0.87 | 27.62 | 111.00 | -2.06 | 0.91 | 0.333 |
| VC_2732 | 3.86 | 0.38 | 5.69 | 20.00 | -2.21 | 3.22 | 0.957 |
| VC_2731 | 6.84 | 0.37 | 80.92 | 46.00 | 0.80 | 0.06 | 0.338 |
| VC_2730 | 5.86 | 0.88 | 8.51 | 30.00 | -2.06 | 2.00 | 0.762 |
| VC_2729 | 6.88 | 0.33 | 46.80 | 23.00 | 1.00 | 0.07 | 0.685 |
| VC_2728 | 2.84 | 0.37 | 117.88 | 126.00 | -0.11 | 0.11 | 0.876 |
| VC_2727 | 11.32 | 0.66 | 66.10 | 174.00 | -1.42 | 0.33 | 0.804 |
| VC_2726 | 16.04 | 0.83 | 69.03 | 54.00 | 0.33 | 0.11 | 0.265 |
| VC_2725 | 10.15 | 1.06 | 46.11 | 18.00 | 1.33 | 0.06 | 0.793 |
| VC_2724 | 8.97 | 0.17 | 207.53 | 46.00 | 2.17 | 0.01 | 0.044 |
| VC_2723 | 17.00 | 0.00 | 119.20 | 168.00 | -0.51 | 0.14 | 0.182 |
| VC_2722 | 19.95 | 0.22 | 408.79 | 466.00 | -0.19 | 0.06 | 0.935 |
| VC_2721 | 14.00 | 0.00 | 138.94 | 258.00 | -0.90 | 0.16 | 0.136 |
| VC_2720 | 14.33 | 0.77 | 209.32 | 123.00 | 0.76 | 0.04 | 0.010 |
| VC_2719 | 17.97 | 0.17 | 311.09 | 366.00 | -0.24 | 0.06 | 0.844 |
| VC_2718 | 21.27 | 0.68 | 293.37 | 538.00 | -0.88 | 0.10 | 0.199 |
| VC_2717 | 13.94 | 0.24 | 467.37 | 463.00 | 0.01 | 0.05 | 0.711 |
| VC_2716 | 44.30 | 0.76 | 769.44 | 626.00 | 0.30 | 0.03 | 0.172 |
| VC_2715 | 14.17 | 0.82 | 151.13 | 103.00 | 0.55 | 0.05 | 0.823 |
| VC_2714 | 13.40 | 0.64 | 89.33 | 154.00 | -0.81 | 0.21 | 0.502 |
| VC_2713 | 25.28 | 0.70 | 451.44 | 476.00 | -0.08 | 0.05 | 0.652 |
| VC_2712 | 42.84 | 0.91 | 855.75 | 744.00 | 0.20 | 0.03 | 0.383 |
| VC_2711 | 34.80 | 0.88 | 528.97 | 310.00 | 0.77 | 0.02 | 0.375 |
| VC_2710 | 19.42 | 0.62 | 255.52 | 320.00 | -0.33 | 0.08 | 0.432 |
| VC_2709 | 5.00 | 0.00 | 44.23 | 52.00 | -0.27 | 0.21 | 0.512 |
| VC_2708 | 5.96 | 0.20 | 15.77 | 41.00 | -1.46 | 0.74 | 0.321 |
| VC_2707 | 0.72 | 0.45 | 1.30 | 1.00 | 0.33 | 0.24 | 1.000 |
| VC_2706 | 21.85 | 0.88 | 210.94 | 315.00 | -0.58 | 0.09 | 0.378 |
| VC_2705 | 59.26 | 0.63 | 1227.85 | 1400.00 | -0.19 | 0.04 | 0.597 |
| VC_2704 | 6.59 | 0.49 | 79.87 | 174.00 | -1.14 | 0.27 | 0.910 |
| VC_2703 | 43.29 | 0.67 | 560.44 | 483.00 | 0.21 | 0.04 | 0.757 |
| VC_2702 | 13.98 | 0.14 | 138.02 | 305.00 | -1.16 | 0.21 | 0.326 |
| VC_2701 | 39.32 | 0.68 | 801.97 | 357.00 | 1.17 | 0.02 | 0.252 |
| VC_2700 | 89.18 | 0.81 | 1374.58 | 1359.00 | 0.02 | 0.03 | 0.722 |
| VC_2699 | 26.91 | 0.84 | 615.62 | 424.00 | 0.54 | 0.03 | 0.269 |
| VC_2698 | 38.60 | 0.99 | 420.95 | 647.00 | -0.62 | 0.07 | 0.138 |

| | | | | | | | |
|---|---|---|---|---|---|---|---|
| VC_2697 | 20.62 | 0.92 | 503.01 | 479.00 | 0.07 | 0.04 | 0.887 |
| VC_2696 | 12.29 | 0.64 | 165.38 | 209.00 | -0.35 | 0.10 | 0.567 |
| VC_2695 | 10.55 | 0.52 | 54.27 | 120.00 | -1.17 | 0.31 | 0.582 |
| VC_2694 | 29.41 | 0.62 | 869.87 | 858.00 | 0.02 | 0.04 | 0.792 |
| VC_2693 | 36.60 | 0.49 | 767.01 | 499.00 | 0.62 | 0.02 | 0.184 |
| VC_2692 | 12.64 | 0.48 | 109.16 | 171.00 | -0.66 | 0.15 | 0.324 |
| VC_2691 | 3.00 | 0.00 | 31.49 | 88.00 | -1.53 | 0.53 | 0.300 |
| VC_2690 | 22.95 | 0.82 | 345.40 | 600.00 | -0.80 | 0.11 | 0.318 |
| VC_2689 | 12.93 | 0.26 | 6.42 | 60.00 | -3.47 | 6.55 | 0.001 |
| VC_2688 | 23.21 | 0.67 | 475.42 | 169.00 | 1.49 | 0.02 | 0.095 |
| VC_2687 | 2.57 | 0.50 | 33.64 | 12.00 | 1.43 | 0.08 | 0.829 |
| VC_2686 | 3.67 | 0.47 | 1.41 | 24.00 | -4.33 | 6.12 | 0.131 |
| VC_2685 | 26.91 | 0.29 | 602.24 | 653.00 | -0.12 | 0.05 | 0.621 |
| VC_2684 | 56.52 | 0.88 | 995.23 | 1092.00 | -0.13 | 0.03 | 0.790 |
| VC_2683 | 33.00 | 0.90 | 522.99 | 606.00 | -0.22 | 0.05 | 0.838 |
| VC_2682 | 8.64 | 0.48 | 117.03 | 180.00 | -0.63 | 0.14 | 0.762 |
| VC_2681 | 31.29 | 0.69 | 749.51 | 713.00 | 0.07 | 0.04 | 0.382 |
| VC_2680 | 6.97 | 0.17 | 61.54 | 24.00 | 1.34 | 0.05 | 0.728 |
| VC_2679 | 3.95 | 0.22 | 5.48 | 21.00 | -2.24 | 3.05 | 0.756 |
| VC_2678 | 2.00 | 0.00 | 1.00 | 11.00 | -3.46 | 0.00 | 0.333 |
| VC_2677 | 15.97 | 0.17 | 299.28 | 259.00 | 0.20 | 0.05 | 0.250 |
| VC_2676 | 0.00 | 0.00 | 1.00 | 1.00 | #VALEUR! | NaN | NaN |
| VC_2675 | 18.31 | 0.63 | 291.55 | 444.00 | -0.61 | 0.08 | 0.453 |
| VC_2674 | 20.14 | 0.68 | 355.61 | 317.00 | 0.16 | 0.05 | 0.854 |
| VC_2673 | 22.94 | 0.81 | 197.35 | 276.00 | -0.49 | 0.10 | 0.591 |
| VC_2672 | 15.60 | 0.49 | 396.81 | 281.00 | 0.50 | 0.03 | 0.683 |
| VC_2671 | 44.15 | 0.77 | 882.17 | 710.00 | 0.31 | 0.03 | 0.862 |
| VC_2670 | 4.00 | 0.00 | 1.00 | 21.00 | -4.39 | 0.00 | 0.029 |
| VC_2669 | 8.95 | 0.22 | 100.72 | 65.00 | 0.61 | 0.07 | 0.180 |
| VC_2668 | 11.00 | 0.00 | 205.84 | 106.00 | 0.95 | 0.04 | 0.387 |
| VC_2667 | 9.66 | 0.48 | 139.97 | 126.00 | 0.14 | 0.08 | 0.737 |
| VC_2666 | 12.00 | 0.00 | 204.79 | 84.00 | 1.28 | 0.03 | 0.135 |
| VC_2665 | 1.00 | 0.00 | 1.00 | 9.00 | -3.17 | 0.00 | 1.000 |
| VC_2664 | 1.00 | 0.00 | 154.28 | 104.00 | 0.56 | 0.06 | 1.000 |
| VC_2663 | 7.00 | 0.00 | 301.35 | 65.00 | 2.21 | 0.01 | 0.745 |
| VC_2662 | 16.00 | 0.00 | 239.37 | 200.00 | 0.25 | 0.06 | 0.324 |
| VC_2661 | 14.45 | 0.64 | 154.21 | 66.00 | 1.21 | 0.04 | 0.041 |
| VC_2660 | 14.35 | 0.73 | 122.73 | 60.00 | 1.02 | 0.04 | 0.016 |
| VC_2659 | 12.00 | 0.00 | 272.59 | 175.00 | 0.63 | 0.04 | 0.510 |
| VC_2658 | 13.58 | 0.55 | 201.19 | 404.00 | -1.01 | 0.16 | 0.303 |

| | | | | | | | |
|---|---|---|---|---|---|---|---|
| VC_2657 | 23.02 | 0.78 | 420.99 | 526.00 | -0.33 | 0.06 | 0.110 |
| VC_2656 | 47.72 | 0.51 | 1177.66 | 784.00 | 0.59 | 0.02 | 0.123 |
| VC_2655 | 15.37 | 0.68 | 56.32 | 136.00 | -1.30 | 0.35 | 0.348 |
| VC_2654 | 9.99 | 0.10 | 140.18 | 114.00 | 0.29 | 0.07 | 0.376 |
| VC_2653 | 5.00 | 0.00 | 133.92 | 106.00 | 0.33 | 0.07 | 0.874 |
| VC_2652 | 4.96 | 0.20 | 6.52 | 81.00 | -3.96 | 10.11 | 0.398 |
| VC_2651 | 0.00 | 0.00 | 1.00 | 1.00 | #VALEUR! | NaN | NaN |
| VC_2650 | 4.92 | 0.27 | 126.72 | 77.00 | 0.71 | 0.06 | 0.315 |
| VC_2649 | 5.60 | 0.49 | 8.21 | 36.00 | -2.36 | 2.77 | 0.266 |
| VC_2648 | 1.93 | 0.26 | 33.63 | 37.00 | -0.19 | 0.22 | 0.957 |
| VC_2647 | 20.28 | 0.71 | 208.13 | 136.00 | 0.61 | 0.04 | 0.365 |
| VC_2646 | 53.33 | 0.97 | 1235.61 | 1035.00 | 0.25 | 0.02 | 0.273 |
| VC_2645 | 30.57 | 0.56 | 406.48 | 536.00 | -0.40 | 0.08 | 0.888 |
| VC_2644 | 27.64 | 0.48 | 375.42 | 326.00 | 0.20 | 0.05 | 0.691 |
| VC_2643 | 13.63 | 0.49 | 232.25 | 127.00 | 0.87 | 0.03 | 0.511 |
| VC_2642 | 31.58 | 0.57 | 442.83 | 409.00 | 0.11 | 0.05 | 0.896 |
| VC_2641 | 28.61 | 0.49 | 487.09 | 501.00 | -0.04 | 0.05 | 0.881 |
| VC_2640 | 8.54 | 0.58 | 154.53 | 83.00 | 0.89 | 0.04 | 0.506 |
| VC_2639 | 0.00 | 0.00 | 1.00 | 1.00 | #VALEUR! | NaN | NaN |
| VC_2638 | 35.45 | 0.61 | 669.94 | 742.00 | -0.15 | 0.04 | 0.771 |
| VC_2637 | 22.09 | 0.74 | 164.92 | 394.00 | -1.26 | 0.18 | 0.030 |
| VC_2636 | 25.98 | 0.14 | 212.45 | 305.00 | -0.53 | 0.09 | 0.299 |
| VC_2635 | 49.58 | 1.25 | 97.89 | 403.00 | -2.06 | 0.45 | 0.000 |
| VC_2634 | 26.19 | 0.80 | 324.22 | 340.00 | -0.07 | 0.06 | 0.091 |
| VC_2633 | 11.00 | 0.00 | 248.28 | 283.00 | -0.19 | 0.07 | 0.450 |
| VC_2632 | 17.32 | 0.60 | 253.73 | 215.00 | 0.23 | 0.06 | 0.626 |
| VC_2631 | 13.97 | 0.17 | 242.05 | 267.00 | -0.15 | 0.08 | 0.323 |
| VC_2630 | 47.72 | 0.91 | 609.65 | 456.00 | 0.42 | 0.03 | 0.530 |
| VC_2629 | 18.24 | 0.65 | 212.08 | 227.00 | -0.10 | 0.08 | 0.152 |
| VC_2628 | 26.30 | 0.67 | 547.18 | 451.00 | 0.28 | 0.03 | 0.890 |
| VC_2627 | 36.39 | 0.60 | 544.88 | 436.00 | 0.32 | 0.03 | 0.325 |
| VC_2626 | 0.00 | 0.00 | 1.00 | 1.00 | #VALEUR! | NaN | NaN |
| VC_2625 | 10.89 | 0.31 | 61.24 | 48.00 | 0.33 | 0.10 | 0.400 |
| VC_2624 | 21.59 | 0.49 | 154.45 | 345.00 | -1.17 | 0.19 | 0.028 |
| VC_2623 | 1.00 | 0.00 | 11.18 | 1.00 | 3.33 | 0.04 | 1.000 |
| VC_2622 | 23.33 | 0.67 | 220.41 | 556.00 | -1.34 | 0.16 | 0.015 |
| VC_2621 | 74.72 | 0.47 | 1221.15 | 1666.00 | -0.45 | 0.04 | 0.149 |
| VC_2620 | 26.87 | 0.84 | 700.12 | 319.00 | 1.13 | 0.02 | 0.041 |
| VC_2619 | 12.00 | 0.00 | 348.84 | 353.00 | -0.02 | 0.05 | 0.655 |
| VC_2618 | 19.85 | 0.36 | 404.65 | 502.00 | -0.32 | 0.07 | 0.870 |

| | | | | | | | |
|---|---|---|---|---|---|---|---|
| VC_2617 | 32.61 | 0.58 | 918.66 | 329.00 | 1.48 | 0.01 | 0.019 |
| VC_2616 | 33.52 | 0.52 | 505.55 | 478.00 | 0.08 | 0.04 | 0.184 |
| VC_2615 | 25.00 | 0.00 | 283.50 | 298.00 | -0.08 | 0.07 | 0.312 |
| VC_2614 | 12.41 | 1.03 | 40.27 | 63.00 | -0.68 | 0.25 | 0.303 |
| VC_2613 | 25.75 | 0.50 | 265.77 | 341.00 | -0.36 | 0.07 | 0.567 |
| VC_2612 | 5.00 | 0.00 | 147.85 | 106.00 | 0.47 | 0.06 | 0.979 |
| VC_2611 | 3.00 | 0.00 | 18.35 | 28.00 | -0.69 | 0.41 | 0.554 |
| VC_2610 | 25.28 | 0.67 | 244.23 | 474.00 | -0.96 | 0.11 | 0.055 |
| VC_2609 | 11.00 | 0.00 | 111.02 | 434.00 | -1.98 | 0.41 | 0.036 |
| VC_2608 | 45.37 | 0.63 | 702.25 | 532.00 | 0.40 | 0.03 | 0.248 |
| VC_2607 | 12.65 | 0.48 | 588.46 | 417.00 | 0.49 | 0.03 | 0.622 |
| VC_2606 | 40.47 | 0.97 | 478.95 | 707.00 | -0.56 | 0.06 | 0.028 |
| VC_2605 | 5.06 | 0.80 | 92.85 | 59.00 | 0.64 | 0.06 | 0.227 |
| VC_2604 | 13.00 | 0.00 | 268.93 | 198.00 | 0.44 | 0.05 | 0.900 |
| VC_2603 | 15.00 | 0.00 | 246.30 | 378.00 | -0.62 | 0.09 | 0.574 |
| VC_2602 | 0.00 | 0.00 | 1.00 | 1.00 | #VALEUR! | NaN | NaN |
| VC_2601 | 26.00 | 0.80 | 353.30 | 515.00 | -0.55 | 0.09 | 0.640 |
| VC_2600 | 59.83 | 0.88 | 1164.38 | 1222.00 | -0.07 | 0.03 | 0.166 |
| VC_2599 | 58.48 | 0.64 | 1146.97 | 1157.00 | -0.01 | 0.03 | 0.302 |
| VC_2598 | 17.96 | 0.20 | 240.30 | 342.00 | -0.51 | 0.09 | 0.928 |
| VC_2597 | 0.00 | 0.00 | 1.00 | 1.00 | #VALEUR! | NaN | NaN |
| VC_2596 | 0.00 | 0.00 | 1.00 | 1.00 | #VALEUR! | NaN | NaN |
| VC_2595 | 0.00 | 0.00 | 1.00 | 1.00 | #VALEUR! | NaN | NaN |
| VC_2594 | 0.00 | 0.00 | 1.00 | 1.00 | #VALEUR! | NaN | NaN |
| VC_2593 | 0.00 | 0.00 | 1.00 | 1.00 | #VALEUR! | NaN | NaN |
| VC_2592 | 0.00 | 0.00 | 1.00 | 1.00 | #VALEUR! | NaN | NaN |
| VC_2591 | 0.00 | 0.00 | 1.00 | 1.00 | #VALEUR! | NaN | NaN |
| VC_2590 | 0.00 | 0.00 | 1.00 | 1.00 | #VALEUR! | NaN | NaN |
| VC_2589 | 0.00 | 0.00 | 1.00 | 1.00 | #VALEUR! | NaN | NaN |
| VC_2588 | 0.00 | 0.00 | 1.00 | 1.00 | #VALEUR! | NaN | NaN |
| VC_2587 | 0.98 | 0.14 | 4.36 | 1.00 | 1.74 | 0.20 | 1.000 |
| VC_2586 | 0.00 | 0.00 | 1.00 | 1.00 | #VALEUR! | NaN | NaN |
| VC_2585 | 0.00 | 0.00 | 1.00 | 1.00 | #VALEUR! | NaN | NaN |
| VC_2584 | 0.00 | 0.00 | 1.00 | 1.00 | #VALEUR! | NaN | NaN |
| VC_2583 | 0.00 | 0.00 | 1.00 | 1.00 | #VALEUR! | NaN | NaN |
| VC_2582 | 0.00 | 0.00 | 1.00 | 1.00 | #VALEUR! | NaN | NaN |
| VC_2581 | 0.00 | 0.00 | 1.00 | 1.00 | #VALEUR! | NaN | NaN |
| VC_2580 | 0.00 | 0.00 | 1.00 | 1.00 | #VALEUR! | NaN | NaN |
| VC_2579 | 0.00 | 0.00 | 1.00 | 1.00 | #VALEUR! | NaN | NaN |
| VC_2578 | 0.00 | 0.00 | 1.00 | 1.00 | #VALEUR! | NaN | NaN |

| | | | | | | | |
|---|---|---|---|---|---|---|---|
| VC_2577 | 0.00 | 0.00 | 1.00 | 1.00 | #VALEUR! | NaN | NaN |
| VC_2576 | 0.00 | 0.00 | 1.00 | 1.00 | #VALEUR! | NaN | NaN |
| VC_2575 | 0.00 | 0.00 | 1.00 | 1.00 | #VALEUR! | NaN | NaN |
| VC_2574 | 0.00 | 0.00 | 1.00 | 1.00 | #VALEUR! | NaN | NaN |
| VC_2573 | 0.00 | 0.00 | 1.00 | 1.00 | #VALEUR! | NaN | NaN |
| VC_2572 | 0.00 | 0.00 | 1.00 | 1.00 | #VALEUR! | NaN | NaN |
| VC_2571 | 0.00 | 0.00 | 1.00 | 1.00 | #VALEUR! | NaN | NaN |
| VC_2570 | 0.00 | 0.00 | 1.00 | 1.00 | #VALEUR! | NaN | NaN |
| VC_2569 | 16.65 | 0.52 | 365.43 | 315.00 | 0.21 | 0.05 | 0.818 |
| VC_2568 | 18.71 | 0.46 | 354.68 | 163.00 | 1.12 | 0.03 | 0.262 |
| VC_2567 | 12.00 | 0.00 | 291.36 | 32.00 | 3.18 | 0.01 | 0.026 |
| VC_2566 | 20.69 | 0.46 | 219.78 | 294.00 | -0.43 | 0.09 | 0.700 |
| VC_2565 | 13.95 | 0.22 | 415.32 | 62.00 | 2.74 | 0.01 | 0.004 |
| VC_2564 | 43.67 | 0.47 | 608.44 | 1006.00 | -0.73 | 0.07 | 0.034 |
| VC_2563 | 18.95 | 0.22 | 164.08 | 441.00 | -1.44 | 0.23 | 0.113 |
| VC_2562 | 58.38 | 0.94 | 679.66 | 717.00 | -0.08 | 0.04 | 0.374 |
| VC_2561 | 17.50 | 0.69 | 300.36 | 306.00 | -0.03 | 0.05 | 0.667 |
| VC_2560 | 17.00 | 0.00 | 368.85 | 197.00 | 0.90 | 0.03 | 0.120 |
| VC_2559 | 36.04 | 0.80 | 788.36 | 439.00 | 0.84 | 0.02 | 0.000 |
| VC_2558 | 14.00 | 0.00 | 333.89 | 169.00 | 0.98 | 0.03 | 0.511 |
| VC_2557 | 19.00 | 0.00 | 284.39 | 126.00 | 1.17 | 0.03 | 0.100 |
| VC_2556 | 7.99 | 0.10 | 76.70 | 110.00 | -0.54 | 0.16 | 0.547 |
| VC_2555 | 21.98 | 0.14 | 394.67 | 334.00 | 0.24 | 0.04 | 0.370 |
| VC_2554 | 29.88 | 0.33 | 479.84 | 596.00 | -0.32 | 0.05 | 0.657 |
| VC_2553 | 20.68 | 0.47 | 267.18 | 362.00 | -0.44 | 0.08 | 0.222 |
| VC_2552 | 17.70 | 0.46 | 366.77 | 379.00 | -0.05 | 0.05 | 0.905 |
| VC_2551 | 6.00 | 0.00 | 198.73 | 123.00 | 0.68 | 0.05 | 0.934 |
| VC_2550 | 12.97 | 0.17 | 256.33 | 294.00 | -0.20 | 0.07 | 0.634 |
| VC_2549 | 16.00 | 0.00 | 180.66 | 248.00 | -0.47 | 0.11 | 0.383 |
| VC_2548 | 49.14 | 0.74 | 383.39 | 673.00 | -0.81 | 0.07 | 0.090 |
| VC_2547 | 83.31 | 1.01 | 776.78 | 1277.00 | -0.72 | 0.06 | 0.109 |
| VC_2546 | 13.60 | 0.49 | 167.88 | 127.00 | 0.39 | 0.06 | 0.845 |
| VC_2545 | 1.00 | 0.00 | 12.52 | 1.00 | 3.51 | 0.03 | 1.000 |
| VC_2544 | 26.16 | 0.79 | 471.62 | 421.00 | 0.16 | 0.04 | 0.126 |
| VC_2543 | 5.00 | 0.00 | 28.23 | 78.00 | -1.53 | 0.64 | 0.187 |
| VC_2542 | 31.94 | 1.08 | 351.93 | 464.00 | -0.40 | 0.08 | 0.805 |
| VC_2541 | 4.96 | 0.20 | 53.54 | 12.00 | 2.12 | 0.04 | 0.082 |
| VC_2540 | 5.89 | 0.31 | 76.22 | 10.00 | 2.91 | 0.02 | 0.067 |
| VC_2539 | 20.20 | 0.72 | 132.24 | 241.00 | -0.88 | 0.15 | 0.340 |
| VC_2538 | 42.92 | 0.97 | 168.65 | 444.00 | -1.41 | 0.22 | 0.043 |

| | | | | | | | |
|---|---|---|---|---|---|---|---|
| VC_2537 | 18.99 | 0.10 | 122.54 | 269.00 | -1.15 | 0.19 | 0.260 |
| VC_2536 | 7.86 | 0.35 | 124.93 | 50.00 | 1.31 | 0.04 | 0.155 |
| VC_2535 | 42.98 | 0.77 | 870.10 | 829.00 | 0.07 | 0.03 | 0.680 |
| VC_2534 | 28.78 | 0.42 | 806.51 | 403.00 | 1.00 | 0.02 | 0.617 |
| VC_2533 | 5.00 | 0.00 | 55.89 | 32.00 | 0.77 | 0.09 | 0.141 |
| VC_2532 | 16.00 | 0.00 | 375.10 | 210.00 | 0.83 | 0.03 | 0.093 |
| VC_2531 | 11.96 | 0.94 | 154.36 | 60.00 | 1.35 | 0.03 | 0.449 |
| VC_2530 | 10.95 | 0.22 | 173.76 | 147.00 | 0.23 | 0.07 | 0.821 |
| VC_2529 | 47.57 | 0.56 | 1225.18 | 890.00 | 0.46 | 0.02 | 0.302 |
| VC_2528 | 0.00 | 0.00 | 1.00 | 1.00 | #VALEUR! | NaN | NaN |
| VC_2527 | 4.00 | 0.00 | 19.85 | 30.00 | -0.68 | 0.43 | 0.823 |
| VC_2525 | 0.00 | 0.00 | 1.00 | 1.00 | #VALEUR! | NaN | NaN |
| VC_2524 | 3.91 | 0.29 | 12.50 | 13.00 | -0.25 | 0.52 | 0.577 |
| VC_2523 | 5.00 | 0.00 | 56.89 | 55.00 | 0.02 | 0.16 | 0.814 |
| VC_2522 | 27.83 | 0.85 | 542.85 | 315.00 | 0.78 | 0.02 | 0.438 |
| VC_2521 | 6.96 | 0.20 | 50.92 | 91.00 | -0.86 | 0.25 | 0.876 |
| VC_2520 | 21.62 | 0.49 | 94.09 | 230.00 | -1.31 | 0.26 | 0.036 |
| VC_2519 | 11.19 | 0.73 | 51.52 | 236.00 | -2.24 | 0.81 | 0.136 |
| VC_2518 | 13.00 | 0.00 | 1.00 | 193.00 | -7.59 | 0.00 | 0.000 |
| VC_2517 | 11.00 | 0.00 | 1.47 | 222.00 | -7.52 | 59.28 | 0.000 |
| VC_2516 | 7.00 | 0.00 | 1.38 | 164.00 | -7.12 | 40.28 | 0.001 |
| VC_2515 | 6.00 | 0.00 | 1.00 | 32.00 | -5.00 | 0.00 | 0.002 |
| VC_2514 | 0.00 | 0.00 | 1.00 | 1.00 | #VALEUR! | NaN | NaN |
| VC_2513 | 0.96 | 0.20 | 3.15 | 1.00 | 1.30 | 0.24 | 1.000 |
| VC_2512 | 8.00 | 0.00 | 84.12 | 98.00 | -0.24 | 0.14 | 0.166 |
| VC_2511 | 7.82 | 0.39 | 109.11 | 82.00 | 0.40 | 0.07 | 0.525 |
| VC_2510 | 13.79 | 0.41 | 235.54 | 191.00 | 0.30 | 0.05 | 0.523 |
| VC_2509 | 2.00 | 0.00 | 8.56 | 1.00 | 2.84 | 0.07 | 1.000 |
| VC_2508 | 24.64 | 0.48 | 345.85 | 281.00 | 0.29 | 0.05 | 0.305 |
| VC_2507 | 26.24 | 0.73 | 601.20 | 411.00 | 0.55 | 0.03 | 0.153 |
| VC_2506 | 49.27 | 0.66 | 1194.91 | 1077.00 | 0.15 | 0.03 | 0.693 |
| VC_2505 | 11.82 | 0.39 | 204.59 | 243.00 | -0.26 | 0.09 | 0.258 |
| VC_2504 | 27.70 | 0.46 | 571.06 | 448.00 | 0.35 | 0.03 | 0.672 |
| VC_2503 | 1.89 | 0.31 | 12.87 | 1.00 | 3.54 | 0.04 | 0.407 |
| VC_2502 | 10.84 | 0.37 | 231.61 | 207.00 | 0.16 | 0.06 | 0.729 |
| VC_2501 | 45.38 | 0.62 | 894.19 | 448.00 | 0.99 | 0.02 | 0.203 |
| VC_2500 | 1.99 | 0.10 | 5.27 | 14.00 | -1.86 | 2.60 | 1.000 |
| VC_2499 | 1.94 | 0.24 | 11.54 | 9.00 | 0.22 | 0.29 | 0.857 |
| VC_2498 | 13.58 | 0.50 | 431.48 | 192.00 | 1.16 | 0.02 | 0.935 |
| VC_2497 | 44.56 | 0.57 | 491.27 | 535.00 | -0.13 | 0.05 | 0.759 |

| | | | | | | | |
|---|---|---|---|---|---|---|---|
| VC_2496 | 4.00 | 0.00 | 16.92 | 32.00 | -1.01 | 0.58 | 0.351 |
| VC_2495 | 5.59 | 0.49 | 29.06 | 28.00 | 0.00 | 0.20 | 0.821 |
| VC_2494 | 34.85 | 0.36 | 575.77 | 585.00 | -0.03 | 0.04 | 0.518 |
| VC_2493 | 11.86 | 0.38 | 140.06 | 129.00 | 0.11 | 0.08 | 0.493 |
| VC_2492 | 25.95 | 0.22 | 472.76 | 286.00 | 0.72 | 0.03 | 0.531 |
| VC_2491 | 24.62 | 0.58 | 474.28 | 560.00 | -0.24 | 0.05 | 0.574 |
| VC_2490 | 41.37 | 0.61 | 1095.25 | 1049.00 | 0.06 | 0.03 | 0.472 |
| VC_2489 | 21.62 | 0.49 | 262.80 | 418.00 | -0.67 | 0.10 | 0.317 |
| VC_2488 | 27.62 | 0.49 | 753.31 | 552.00 | 0.45 | 0.03 | 0.439 |
| VC_2487 | 27.53 | 0.54 | 337.78 | 344.00 | -0.03 | 0.06 | 0.247 |
| VC_2486 | 5.00 | 0.00 | 17.01 | 62.00 | -1.98 | 1.33 | 0.059 |
| VC_2485 | 18.64 | 1.01 | 362.72 | 169.00 | 1.10 | 0.03 | 0.252 |
| VC_2484 | 51.24 | 0.95 | 624.82 | 790.00 | -0.34 | 0.05 | 0.286 |
| VC_2483 | 50.35 | 0.73 | 608.43 | 1009.00 | -0.73 | 0.07 | 0.894 |
| VC_2482 | 10.98 | 0.14 | 291.44 | 79.00 | 1.88 | 0.02 | 0.223 |
| VC_2481 | 31.55 | 0.54 | 675.13 | 760.00 | -0.17 | 0.04 | 0.868 |
| VC_2480 | 4.43 | 0.57 | 7.36 | 20.00 | -1.68 | 1.52 | 0.862 |
| VC_2479 | 12.67 | 0.47 | 185.75 | 407.00 | -1.14 | 0.16 | 0.929 |
| VC_2478 | 8.00 | 0.00 | 138.19 | 29.00 | 2.24 | 0.02 | 0.512 |
| VC_2477 | 4.00 | 0.00 | 21.08 | 45.00 | -1.17 | 0.56 | 0.412 |
| VC_2476 | 14.85 | 0.36 | 104.64 | 305.00 | -1.56 | 0.27 | 0.278 |
| VC_2475 | 9.46 | 0.59 | 65.49 | 30.00 | 1.10 | 0.07 | 0.004 |
| VC_2474 | 2.88 | 0.33 | 3.49 | 11.00 | -2.20 | 3.03 | 0.488 |
| VC_2473 | 12.00 | 0.00 | 177.96 | 86.00 | 1.04 | 0.03 | 0.282 |
| VC_2472 | 6.99 | 0.77 | 9.53 | 20.00 | -1.20 | 0.75 | 0.825 |
| VC_2471 | 2.62 | 0.49 | 46.22 | 41.00 | 0.14 | 0.14 | 0.740 |
| VC_2470 | 11.00 | 0.00 | 198.63 | 154.00 | 0.36 | 0.06 | 0.744 |
| VC_2469 | 54.17 | 0.70 | 1642.26 | 2051.00 | -0.32 | 0.03 | 0.276 |
| VC_2468 | 2.00 | 0.00 | 14.16 | 81.00 | -2.66 | 2.29 | 0.523 |
| VC_2467 | 0.00 | 0.00 | 1.00 | 1.00 | #VALEUR! | NaN | NaN |
| VC_2466 | 5.74 | 0.44 | 31.96 | 16.00 | 0.95 | 0.10 | 0.771 |
| VC_2465 | 17.87 | 0.37 | 191.54 | 74.00 | 1.36 | 0.03 | 0.040 |
| VC_2464 | 2.00 | 0.00 | 4.16 | 24.00 | -2.95 | 5.30 | 0.870 |
| VC_2463 | 38.31 | 0.75 | 504.39 | 448.00 | 0.17 | 0.04 | 0.655 |
| VC_2462 | 2.00 | 0.00 | 5.25 | 8.00 | -1.04 | 1.57 | 0.827 |
| VC_2461 | 1.00 | 0.00 | 1.31 | 23.00 | -4.32 | 5.25 | 1.000 |
| VC_2460 | 1.00 | 0.00 | 24.97 | 1.00 | 4.57 | 0.01 | 1.000 |
| VC_2459 | 24.57 | 0.56 | 210.15 | 247.00 | -0.24 | 0.08 | 0.838 |
| VC_2458 | 0.00 | 0.00 | 1.00 | 1.00 | #VALEUR! | NaN | NaN |
| VC_2457 | 1.00 | 0.00 | 1.00 | 19.00 | -4.25 | 0.00 | 1.000 |

| | | | | | | | |
|---|---|---|---|---|---|---|---|
| VC_2456 | 39.13 | 0.69 | 573.96 | 758.00 | -0.40 | 0.05 | 0.877 |
| VC_2455 | 14.18 | 0.67 | 279.76 | 188.00 | 0.57 | 0.04 | 0.657 |
| VC_2454 | 34.63 | 0.49 | 394.54 | 366.00 | 0.10 | 0.05 | 0.773 |
| VC_2453 | 62.90 | 0.30 | 760.37 | 816.00 | -0.10 | 0.04 | 0.411 |
| VC_2452 | 26.00 | 0.00 | 689.66 | 408.00 | 0.76 | 0.02 | 0.237 |
| VC_2451 | 48.98 | 0.89 | 397.54 | 649.00 | -0.71 | 0.08 | 0.193 |
| VC_2450 | 11.93 | 0.26 | 77.81 | 122.00 | -0.67 | 0.19 | 0.220 |
| VC_2449 | 2.56 | 0.50 | 5.19 | 35.00 | -3.11 | 6.16 | 0.862 |
| VC_2448 | 1.00 | 0.00 | 1.00 | 13.00 | -3.70 | 0.00 | 1.000 |
| VC_2447 | 0.00 | 0.00 | 1.00 | 1.00 | #VALEUR! | NaN | NaN |
| VC_2446 | 23.97 | 0.17 | 470.81 | 318.00 | 0.56 | 0.03 | 0.224 |
| VC_2445 | 43.22 | 0.79 | 484.84 | 853.00 | -0.82 | 0.09 | 0.303 |
| VC_2444 | 20.97 | 0.17 | 395.06 | 293.00 | 0.43 | 0.04 | 0.830 |
| VC_2443 | 21.89 | 0.31 | 395.27 | 537.00 | -0.45 | 0.07 | 0.436 |
| VC_2442 | 29.95 | 0.22 | 584.89 | 308.00 | 0.92 | 0.02 | 0.310 |
| VC_2441 | 17.85 | 0.36 | 384.29 | 430.00 | -0.17 | 0.06 | 0.160 |
| VC_2440 | 37.23 | 0.81 | 714.00 | 527.00 | 0.44 | 0.03 | 0.751 |
| VC_2439 | 16.37 | 0.61 | 168.35 | 302.00 | -0.85 | 0.15 | 0.073 |
| VC_2438 | 62.96 | 0.90 | 1261.96 | 1165.00 | 0.11 | 0.03 | 0.009 |
| VC_2437 | 10.00 | 0.00 | 1.41 | 64.00 | -5.72 | 16.20 | 0.000 |
| VC_2436 | 29.69 | 0.46 | 28.31 | 661.00 | -4.63 | 6.37 | 0.000 |
| VC_2435 | 14.27 | 0.71 | 122.88 | 85.00 | 0.52 | 0.06 | 0.844 |
| VC_2434 | 8.00 | 0.00 | 155.99 | 171.00 | -0.14 | 0.10 | 0.119 |
| VC_2433 | 21.35 | 0.64 | 202.10 | 383.00 | -0.93 | 0.11 | 0.797 |
| VC_2432 | 14.74 | 0.44 | 259.84 | 223.00 | 0.22 | 0.05 | 0.833 |
| VC_2431 | 0.00 | 0.00 | 1.00 | 1.00 | #VALEUR! | NaN | NaN |
| VC_2430 | 1.00 | 0.00 | 6.24 | 2.00 | 1.40 | 0.17 | 1.000 |
| VC_2429 | 4.00 | 0.00 | 40.36 | 23.00 | 0.77 | 0.11 | 0.769 |
| VC_2428 | 15.00 | 0.00 | 333.23 | 143.00 | 1.22 | 0.02 | 0.345 |
| VC_2427 | 4.49 | 0.61 | 13.36 | 84.00 | -2.78 | 2.19 | 0.915 |
| VC_2426 | 4.60 | 0.51 | 13.90 | 4.00 | 1.67 | 0.10 | 0.642 |
| VC_2425 | 37.89 | 0.31 | 720.36 | 885.00 | -0.30 | 0.05 | 0.277 |
| VC_2424 | 35.20 | 0.68 | 378.22 | 805.00 | -1.09 | 0.10 | 0.017 |
| VC_2423 | 24.90 | 0.81 | 478.04 | 436.00 | 0.13 | 0.04 | 0.904 |
| VC_2422 | 17.59 | 0.53 | 188.87 | 242.00 | -0.36 | 0.09 | 0.626 |
| VC_2421 | 10.00 | 0.00 | 321.90 | 291.00 | 0.14 | 0.05 | 0.432 |
| VC_2420 | 15.82 | 0.41 | 233.74 | 141.00 | 0.72 | 0.04 | 0.237 |
| VC_2419 | 18.50 | 0.52 | 141.83 | 192.00 | -0.45 | 0.12 | 0.347 |
| VC_2418 | 17.00 | 0.00 | 288.89 | 392.00 | -0.45 | 0.08 | 0.233 |
| VC_2417 | 33.89 | 0.31 | 647.07 | 625.00 | 0.05 | 0.04 | 0.680 |

| | | | | | | | |
|---|---|---|---|---|---|---|---|
| VC_2416 | 61.51 | 0.58 | 1331.05 | 1456.00 | -0.13 | 0.03 | 0.737 |
| VC_2415 | 15.66 | 0.48 | 127.47 | 104.00 | 0.28 | 0.08 | 0.773 |
| VC_2414 | 52.92 | 0.85 | 469.96 | 443.00 | 0.08 | 0.04 | 0.356 |
| VC_2413 | 20.08 | 0.75 | 75.66 | 90.00 | -0.27 | 0.13 | 0.830 |
| VC_2412 | 21.30 | 1.04 | 121.06 | 34.00 | 1.82 | 0.03 | 0.002 |
| VC_2411 | 1.00 | 0.00 | 8.36 | 1.00 | 2.80 | 0.08 | 1.000 |
| VC_2410 | 3.00 | 0.00 | 11.19 | 6.00 | 0.73 | 0.23 | 0.724 |
| VC_2409 | 4.00 | 0.00 | 1.00 | 21.00 | -4.39 | 0.00 | 0.029 |
| VC_2408 | 2.74 | 0.44 | 1.57 | 8.00 | -2.68 | 2.24 | 0.419 |
| VC_2407 | 1.00 | 0.00 | 20.48 | 68.00 | -1.80 | 0.78 | 1.000 |
| VC_2406 | 2.00 | 0.00 | 66.25 | 12.00 | 2.44 | 0.02 | 0.533 |
| VC_2405 | 1.00 | 0.00 | 1.00 | 8.00 | -3.00 | 0.00 | 1.000 |
| VC_2404 | 1.00 | 0.00 | 23.54 | 1.00 | 4.50 | 0.01 | 1.000 |
| VC_2403 | 2.00 | 0.00 | 3.31 | 2.00 | 0.23 | 0.57 | 0.980 |
| VC_2402 | 0.00 | 0.00 | 1.00 | 1.00 | #VALEUR! | NaN | NaN |
| VC_2401 | 0.00 | 0.00 | 1.00 | 1.00 | #VALEUR! | NaN | NaN |
| VC_2400 | 3.61 | 0.49 | 41.67 | 61.00 | -0.59 | 0.25 | 0.889 |
| VC_2399 | 0.00 | 0.00 | 1.00 | 1.00 | #VALEUR! | NaN | NaN |
| VC_2398 | 0.00 | 0.00 | 1.00 | 1.00 | #VALEUR! | NaN | NaN |
| VC_2397 | 0.00 | 0.00 | 1.00 | 1.00 | #VALEUR! | NaN | NaN |
| VC_2396 | 0.00 | 0.00 | 1.00 | 1.00 | #VALEUR! | NaN | NaN |
| VC_2395 | 2.00 | 0.00 | 19.18 | 37.00 | -1.03 | 0.52 | 1.000 |
| VC_2394 | 2.00 | 0.00 | 23.50 | 12.00 | 0.90 | 0.12 | 1.000 |
| VC_2393 | 9.00 | 0.00 | 28.95 | 42.00 | -0.59 | 0.32 | 0.214 |
| VC_2392 | 2.93 | 0.26 | 16.99 | 4.00 | 2.00 | 0.07 | 0.592 |
| VC_2391 | 1.00 | 0.00 | 1.00 | 1.00 | 0.00 | 0.00 | 1.000 |
| VC_2390 | 24.61 | 0.49 | 388.89 | 572.00 | -0.56 | 0.07 | 0.796 |
| VC_2389 | 59.79 | 0.91 | 1092.56 | 934.00 | 0.22 | 0.03 | 0.704 |
| VC_2388 | 9.00 | 0.00 | 104.14 | 126.00 | -0.29 | 0.12 | 0.555 |
| VC_2387 | 9.22 | 0.72 | 67.20 | 34.00 | 0.96 | 0.06 | 0.454 |
| VC_2386 | 21.92 | 0.79 | 59.31 | 53.00 | 0.14 | 0.12 | 0.819 |
| VC_2385 | 25.36 | 0.67 | 227.16 | 173.00 | 0.39 | 0.05 | 0.767 |
| VC_2384 | 13.35 | 0.72 | 147.25 | 158.00 | -0.11 | 0.10 | 0.926 |
| VC_2383 | 16.63 | 0.56 | 231.80 | 119.00 | 0.95 | 0.04 | 0.437 |
| VC_2382 | 14.55 | 0.50 | 144.38 | 184.00 | -0.36 | 0.11 | 0.584 |
| VC_2381 | 16.47 | 0.59 | 197.87 | 189.00 | 0.06 | 0.07 | 0.175 |
| VC_2380 | 25.93 | 0.26 | 286.04 | 476.00 | -0.74 | 0.11 | 0.414 |
| VC_2379 | 2.64 | 0.48 | 2.26 | 13.00 | -2.93 | 3.89 | 0.434 |
| VC_2378 | 7.00 | 0.00 | 42.56 | 141.00 | -1.77 | 0.64 | 0.053 |
| VC_2377 | 25.76 | 0.45 | 864.06 | 464.00 | 0.90 | 0.02 | 0.201 |

| | | | | | | | |
|---|---|---|---|---|---|---|---|
| VC_2376 | 86.98 | 0.78 | 1377.52 | 1685.00 | -0.29 | 0.03 | 0.363 |
| VC_2375 | 2.00 | 0.00 | 45.64 | 1.00 | 5.48 | 0.00 | 0.333 |
| VC_2374 | 18.94 | 0.24 | 349.46 | 204.00 | 0.77 | 0.03 | 0.059 |
| VC_2373 | 93.77 | 1.00 | 1731.21 | 1949.00 | -0.17 | 0.03 | 0.718 |
| VC_2372 | 3.71 | 0.46 | 12.02 | 26.00 | -1.26 | 0.81 | 0.913 |
| VC_2371 | 31.81 | 0.39 | 617.54 | 603.00 | 0.03 | 0.04 | 0.387 |
| VC_2370 | 45.89 | 0.94 | 907.92 | 402.00 | 1.17 | 0.01 | 0.000 |
| VC_2369 | 40.43 | 1.42 | 140.11 | 141.00 | -0.02 | 0.09 | 0.695 |
| VC_2368 | 2.70 | 0.46 | 1.49 | 2.00 | -0.68 | 0.54 | 0.800 |
| VC_2367 | 10.64 | 0.48 | 57.10 | 76.00 | -0.45 | 0.22 | 0.351 |
| VC_2366 | 20.98 | 0.14 | 371.64 | 324.00 | 0.19 | 0.04 | 0.511 |
| VC_2365 | 10.88 | 0.33 | 113.75 | 76.00 | 0.57 | 0.07 | 0.688 |
| VC_2364.1 | 5.00 | 0.00 | 94.08 | 74.00 | 0.33 | 0.09 | 0.993 |
| VC_2364 | 62.51 | 0.58 | 858.04 | 927.00 | -0.11 | 0.03 | 0.620 |
| VC_2363 | 19.58 | 0.52 | 265.27 | 198.00 | 0.42 | 0.04 | 0.365 |
| VC_2362 | 26.88 | 0.33 | 389.36 | 490.00 | -0.34 | 0.07 | 0.303 |
| VC_2361 | 9.40 | 0.65 | 109.33 | 121.00 | -0.16 | 0.12 | 0.583 |
| VC_2360 | 17.00 | 0.00 | 362.38 | 441.00 | -0.29 | 0.07 | 0.819 |
| VC_2359 | 20.85 | 0.36 | 187.11 | 214.00 | -0.20 | 0.10 | 0.119 |
| VC_2358 | 23.65 | 0.48 | 338.60 | 522.00 | -0.63 | 0.09 | 0.279 |
| VC_2357 | 7.71 | 0.46 | 91.67 | 57.00 | 0.67 | 0.07 | 0.582 |
| VC_2356 | 49.04 | 0.83 | 1241.37 | 859.00 | 0.53 | 0.02 | 0.230 |
| VC_2355 | 18.69 | 0.46 | 356.85 | 398.00 | -0.16 | 0.06 | 0.146 |
| VC_2354 | 3.00 | 0.00 | 52.90 | 82.00 | -0.66 | 0.23 | 0.877 |
| VC_2353 | 26.48 | 0.56 | 540.47 | 412.00 | 0.39 | 0.04 | 0.815 |
| VC_2352 | 39.75 | 0.46 | 821.50 | 530.00 | 0.63 | 0.02 | 0.035 |
| VC_2351 | 8.00 | 0.00 | 176.76 | 107.00 | 0.71 | 0.05 | 0.793 |
| VC_2350 | 22.60 | 0.49 | 335.48 | 435.00 | -0.38 | 0.07 | 0.910 |
| VC_2349 | 28.74 | 0.84 | 284.36 | 380.00 | -0.42 | 0.08 | 0.438 |
| VC_2348 | 28.64 | 0.50 | 345.12 | 454.00 | -0.40 | 0.08 | 0.369 |
| VC_2347 | 23.57 | 0.56 | 519.80 | 524.00 | -0.01 | 0.04 | 0.350 |
| VC_2346 | 25.93 | 0.78 | 284.86 | 491.00 | -0.79 | 0.12 | 0.901 |
| VC_2345 | 24.54 | 0.54 | 401.94 | 375.00 | 0.10 | 0.05 | 0.505 |
| VC_2344 | 63.52 | 1.11 | 1118.98 | 798.00 | 0.49 | 0.02 | 0.899 |
| VC_2343 | 39.74 | 0.44 | 809.36 | 831.00 | -0.04 | 0.04 | 0.380 |
| VC_2342 | 1.00 | 0.00 | 1.00 | 4.00 | -2.00 | 0.00 | 1.000 |
| VC_2341 | 64.12 | 0.84 | 914.29 | 707.00 | 0.37 | 0.02 | 0.165 |
| VC_2340 | 33.15 | 0.85 | 674.03 | 528.00 | 0.35 | 0.04 | 0.909 |
| VC_2339 | 29.63 | 0.54 | 604.68 | 395.00 | 0.61 | 0.03 | 0.206 |
| VC_2338 | 77.66 | 0.95 | 1549.30 | 2275.00 | -0.56 | 0.03 | 0.065 |

| | | | | | | | |
|---|---|---|---|---|---|---|---|
| VC_2337 | 21.95 | 0.22 | 353.87 | 333.00 | 0.08 | 0.05 | 0.423 |
| VC_2336 | 10.82 | 0.39 | 335.12 | 241.00 | 0.47 | 0.04 | 0.965 |
| VC_2335 | 12.66 | 0.50 | 167.54 | 130.00 | 0.36 | 0.06 | 0.876 |
| VC_2334 | 24.29 | 0.69 | 529.03 | 344.00 | 0.62 | 0.03 | 0.602 |
| VC_2333 | 29.96 | 0.86 | 426.83 | 702.00 | -0.72 | 0.08 | 0.491 |
| VC_2332 | 30.61 | 0.49 | 564.96 | 475.00 | 0.25 | 0.03 | 0.323 |
| VC_2331 | 3.00 | 0.00 | 7.52 | 3.00 | 1.13 | 0.18 | 1.000 |
| VC_2330 | 32.33 | 0.70 | 798.92 | 501.00 | 0.67 | 0.02 | 0.099 |
| VC_2329 | 0.00 | 0.00 | 1.00 | 1.00 | #VALEUR! | NaN | NaN |
| VC_2328 | 2.00 | 0.00 | 69.93 | 116.00 | -0.75 | 0.21 | 0.667 |
| VC_2327 | 1.90 | 0.30 | 2.25 | 1.00 | 0.72 | 0.31 | 1.000 |
| VC_2326 | 5.00 | 0.00 | 121.42 | 242.00 | -1.01 | 0.20 | 0.812 |
| VC_2325 | 2.00 | 0.00 | 1.00 | 4.00 | -2.00 | 0.00 | 0.333 |
| VC_2324 | 22.88 | 0.33 | 141.26 | 200.00 | -0.51 | 0.13 | 0.276 |
| VC_2323 | 17.54 | 0.50 | 401.14 | 390.00 | 0.04 | 0.05 | 0.800 |
| VC_2322 | 63.56 | 1.05 | 305.99 | 317.00 | -0.06 | 0.06 | 0.516 |
| VC_2321 | 2.00 | 0.00 | 1.00 | 18.00 | -4.17 | 0.00 | 0.333 |
| VC_2320 | 47.93 | 1.43 | 387.78 | 239.00 | 0.70 | 0.03 | 0.374 |
| VC_2319 | 37.25 | 0.70 | 791.15 | 749.00 | 0.08 | 0.03 | 0.454 |
| VC_2318 | 4.89 | 0.31 | 12.33 | 11.00 | 0.02 | 0.34 | 0.826 |
| VC_2317 | 4.00 | 0.00 | 1.00 | 35.00 | -5.13 | 0.00 | 0.029 |
| VC_2316 | 26.71 | 0.46 | 730.03 | 847.00 | -0.22 | 0.05 | 0.523 |
| VC_2315 | 6.00 | 0.00 | 45.84 | 58.00 | -0.37 | 0.21 | 0.934 |
| VC_2314 | 16.44 | 0.52 | 351.40 | 169.00 | 1.05 | 0.03 | 0.233 |
| VC_2313 | 1.00 | 0.00 | 41.99 | 13.00 | 1.65 | 0.06 | 1.000 |
| VC_2312 | 22.91 | 0.90 | 215.03 | 225.00 | -0.07 | 0.08 | 0.264 |
| VC_2311 | 13.22 | 0.75 | 138.91 | 272.00 | -0.98 | 0.15 | 0.317 |
| VC_2310 | 5.00 | 0.00 | 58.71 | 108.00 | -0.91 | 0.26 | 0.440 |
| VC_2309 | 27.72 | 0.47 | 313.77 | 903.00 | -1.53 | 0.17 | 0.006 |
| VC_2308 | 9.60 | 0.49 | 80.91 | 84.00 | -0.07 | 0.12 | 0.751 |
| VC_2307 | 16.84 | 0.37 | 133.40 | 247.00 | -0.90 | 0.17 | 0.919 |
| VC_2306 | 3.00 | 0.00 | 3.42 | 30.00 | -3.63 | 8.52 | 0.314 |
| VC_2305 | 39.61 | 0.90 | 484.60 | 603.00 | -0.32 | 0.06 | 0.664 |
| VC_2304 | 3.22 | 0.75 | 96.48 | 6.00 | 3.99 | 0.01 | 0.223 |
| VC_2303 | 10.71 | 0.46 | 159.98 | 87.00 | 0.87 | 0.05 | 0.253 |
| VC_2302 | 23.54 | 0.52 | 359.58 | 501.00 | -0.48 | 0.08 | 0.763 |
| VC_2301 | 15.68 | 0.55 | 201.22 | 149.00 | 0.42 | 0.06 | 0.226 |
| VC_2300 | 43.08 | 0.79 | 504.84 | 498.00 | 0.02 | 0.05 | 0.859 |
| VC_2299 | 17.54 | 0.50 | 125.61 | 234.00 | -0.91 | 0.16 | 0.029 |
| VC_2298 | 12.67 | 0.47 | 193.89 | 323.00 | -0.74 | 0.12 | 0.255 |

| | | | | | | | |
|---|---|---|---|---|---|---|---|
| VC_2297 | 36.67 | 0.49 | 579.30 | 566.00 | 0.03 | 0.04 | 0.751 |
| VC_2296 | 6.63 | 0.49 | 106.87 | 84.00 | 0.33 | 0.08 | 0.884 |
| VC_2295 | 40.23 | 0.74 | 1424.45 | 610.00 | 1.22 | 0.01 | 0.046 |
| VC_2294 | 38.64 | 0.54 | 924.07 | 707.00 | 0.38 | 0.03 | 0.267 |
| VC_2293 | 16.58 | 0.52 | 295.44 | 272.00 | 0.11 | 0.05 | 0.901 |
| VC_2292 | 21.67 | 0.49 | 296.17 | 190.00 | 0.64 | 0.04 | 0.021 |
| VC_2291 | 25.79 | 0.46 | 485.41 | 331.00 | 0.55 | 0.03 | 0.026 |
| VC_2290 | 42.85 | 0.86 | 698.13 | 312.00 | 1.16 | 0.02 | 0.025 |
| VC_2289 | 9.55 | 0.54 | 68.92 | 70.00 | -0.05 | 0.16 | 0.709 |
| VC_2288 | 4.89 | 0.31 | 98.17 | 18.00 | 2.43 | 0.02 | 0.179 |
| VC_2287 | 28.60 | 0.55 | 347.31 | 442.00 | -0.35 | 0.08 | 0.830 |
| VC_2286 | 24.02 | 0.83 | 332.66 | 151.00 | 1.14 | 0.02 | 0.582 |
| VC_2285 | 45.59 | 1.02 | 939.10 | 559.00 | 0.75 | 0.02 | 0.122 |
| VC_2284 | 2.99 | 0.10 | 18.65 | 1.00 | 4.14 | 0.01 | 0.156 |
| VC_2283 | 32.17 | 0.82 | 369.09 | 577.00 | -0.65 | 0.09 | 0.258 |
| VC_2282 | 28.61 | 0.62 | 315.48 | 320.00 | -0.02 | 0.06 | 0.724 |
| VC_2281 | 18.18 | 0.82 | 198.71 | 71.00 | 1.48 | 0.03 | 0.042 |
| VC_2280 | 13.00 | 0.00 | 490.11 | 182.00 | 1.43 | 0.02 | 0.321 |
| VC_2279 | 44.34 | 0.65 | 610.78 | 612.00 | -0.01 | 0.04 | 0.914 |
| VC_2278 | 33.26 | 0.72 | 361.57 | 740.00 | -1.04 | 0.11 | 0.053 |
| VC_2277 | 13.29 | 0.67 | 368.51 | 553.00 | -0.59 | 0.07 | 0.879 |
| VC_2276 | 29.45 | 0.61 | 321.46 | 251.00 | 0.35 | 0.05 | 0.510 |
| VC_2275 | 10.62 | 0.49 | 85.95 | 113.00 | -0.41 | 0.14 | 0.354 |
| VC_2274 | 19.00 | 0.00 | 197.34 | 189.00 | 0.05 | 0.07 | 0.580 |
| VC_2273 | 26.60 | 0.55 | 473.85 | 536.00 | -0.18 | 0.06 | 0.895 |
| VC_2272 | 14.00 | 0.00 | 196.81 | 394.00 | -1.01 | 0.14 | 0.066 |
| VC_2271 | 27.97 | 0.17 | 421.53 | 363.00 | 0.21 | 0.05 | 0.682 |
| VC_2270 | 19.55 | 0.52 | 326.36 | 364.00 | -0.16 | 0.07 | 0.776 |
| VC_2269 | 22.36 | 0.66 | 230.56 | 446.00 | -0.96 | 0.13 | 0.375 |
| VC_2268 | 12.00 | 0.00 | 18.06 | 124.00 | -2.85 | 1.69 | 0.000 |
| VC_2267 | 10.82 | 0.39 | 62.62 | 38.00 | 0.70 | 0.08 | 0.301 |
| VC_2266 | 3.24 | 0.65 | 15.65 | 1.00 | 3.85 | 0.02 | 0.102 |
| VC_2265 | 6.88 | 0.33 | 10.88 | 6.00 | 0.70 | 0.25 | 0.827 |
| VC_2264 | 15.86 | 0.35 | 130.52 | 93.00 | 0.47 | 0.07 | 0.852 |
| VC_2263 | 6.00 | 0.00 | 87.99 | 62.00 | 0.49 | 0.08 | 0.965 |
| VC_2262 | 51.97 | 0.96 | 842.17 | 724.00 | 0.22 | 0.03 | 0.261 |
| VC_2261 | 1.00 | 0.00 | 1.00 | 24.00 | -4.58 | 0.00 | 1.000 |
| VC_2260 | 1.00 | 0.00 | 1.00 | 7.00 | -2.81 | 0.00 | 1.000 |
| VC_2259 | 0.00 | 0.00 | 1.00 | 1.00 | #VALEUR! | NaN | NaN |
| VC_2258 | 0.00 | 0.00 | 1.00 | 1.00 | #VALEUR! | NaN | NaN |

| | | | | | | | |
|---|---|---|---|---|---|---|---|
| VC_2257 | 1.00 | 0.00 | 40.02 | 1.00 | 5.29 | 0.00 | 1.000 |
| VC_2256 | 0.00 | 0.00 | 1.00 | 1.00 | #VALEUR! | NaN | NaN |
| VC_2255 | 1.00 | 0.00 | 1.00 | 6.00 | -2.58 | 0.00 | 1.000 |
| VC_2254 | 2.00 | 0.00 | 1.00 | 86.00 | -6.43 | 0.00 | 0.333 |
| VC_2253 | 0.00 | 0.00 | 1.00 | 1.00 | #VALEUR! | NaN | NaN |
| VC_2252 | 0.57 | 0.50 | 1.40 | 1.00 | 0.50 | 0.28 | 1.000 |
| VC_2251 | 8.78 | 0.44 | 61.43 | 16.00 | 1.92 | 0.03 | 0.026 |
| VC_2250 | 1.00 | 0.00 | 5.28 | 1.00 | 2.02 | 0.16 | 1.000 |
| VC_2249 | 0.00 | 0.00 | 1.00 | 1.00 | #VALEUR! | NaN | NaN |
| VC_2248 | 1.00 | 0.00 | 1.00 | 5.00 | -2.32 | 0.00 | 1.000 |
| VC_2247 | 1.00 | 0.00 | 1.00 | 17.00 | -4.09 | 0.00 | 1.000 |
| VC_2246 | 12.53 | 0.61 | 151.19 | 82.00 | 0.87 | 0.04 | 0.787 |
| VC_2245 | 0.00 | 0.00 | 1.00 | 1.00 | #VALEUR! | NaN | NaN |
| VC_2244 | 1.00 | 0.00 | 1.00 | 6.00 | -2.58 | 0.00 | 1.000 |
| VC_2243 | 0.58 | 0.50 | 1.40 | 1.00 | 0.45 | 0.28 | 1.000 |
| VC_2242 | 1.46 | 0.59 | 2.91 | 1.00 | 1.16 | 0.27 | 0.642 |
| VC_2241 | 7.61 | 0.49 | 113.56 | 61.00 | 0.89 | 0.05 | 0.362 |
| VC_2240 | 18.80 | 0.40 | 252.43 | 321.00 | -0.35 | 0.08 | 0.727 |
| VC_2239 | 6.98 | 0.14 | 201.29 | 245.00 | -0.29 | 0.09 | 0.958 |
| VC_2238 | 31.92 | 0.80 | 542.72 | 421.00 | 0.36 | 0.03 | 0.094 |
| VC_2237 | 39.79 | 0.81 | 1092.80 | 561.00 | 0.96 | 0.02 | 0.214 |
| VC_2236 | 19.38 | 0.68 | 170.79 | 263.00 | -0.63 | 0.12 | 0.091 |
| VC_2235 | 22.59 | 0.51 | 195.46 | 290.00 | -0.58 | 0.11 | 0.540 |
| VC_2234 | 3.00 | 0.00 | 23.70 | 12.00 | 0.92 | 0.12 | 0.736 |
| VC_2233 | 13.75 | 1.02 | 39.17 | 31.00 | 0.29 | 0.16 | 0.230 |
| VC_2232 | 38.62 | 0.51 | 725.23 | 1110.00 | -0.62 | 0.05 | 0.171 |
| VC_2231 | 64.50 | 0.99 | 1311.32 | 1205.00 | 0.12 | 0.03 | 0.855 |
| VC_2230 | 1.00 | 0.00 | 1.00 | 4.00 | -2.00 | 0.00 | 1.000 |
| VC_2229 | 12.59 | 0.49 | 230.98 | 179.00 | 0.36 | 0.05 | 0.590 |
| VC_2228 | 4.00 | 0.00 | 46.04 | 32.00 | 0.49 | 0.12 | 0.798 |
| VC_2227 | 14.59 | 0.49 | 137.37 | 49.00 | 1.48 | 0.03 | 0.202 |
| VC_2226 | 22.82 | 0.41 | 238.73 | 369.00 | -0.64 | 0.11 | 0.177 |
| VC_2225 | 21.20 | 0.65 | 385.65 | 139.00 | 1.47 | 0.02 | 0.825 |
| VC_2224 | 53.39 | 0.63 | 1416.52 | 942.00 | 0.59 | 0.02 | 0.446 |
| VC_2223 | 30.24 | 0.61 | 354.19 | 290.00 | 0.28 | 0.05 | 0.253 |
| VC_2222 | 17.77 | 0.42 | 389.78 | 334.00 | 0.22 | 0.04 | 0.743 |
| VC_2221 | 5.00 | 0.00 | 56.19 | 69.00 | -0.32 | 0.17 | 0.730 |
| VC_2220 | 23.59 | 0.53 | 247.09 | 179.00 | 0.46 | 0.05 | 0.465 |
| VC_2219 | 1.00 | 0.00 | 6.46 | 27.00 | -2.46 | 4.34 | 1.000 |
| VC_2218 | 1.96 | 0.20 | 3.11 | 24.00 | -3.45 | 6.93 | 1.000 |

| | | | | | | | |
|---|---|---|---|---|---|---|---|
| VC_2217 | 71.14 | 0.83 | 1133.82 | 1631.00 | -0.53 | 0.05 | 0.074 |
| VC_2216 | 15.91 | 0.29 | 129.31 | 217.00 | -0.76 | 0.15 | 0.745 |
| VC_2215 | 64.37 | 1.00 | 732.13 | 1211.00 | -0.73 | 0.05 | 0.325 |
| VC_2214 | 3.99 | 0.10 | 17.96 | 26.00 | -0.63 | 0.46 | 0.795 |
| VC_2213 | 33.87 | 0.37 | 877.16 | 850.00 | 0.04 | 0.03 | 0.653 |
| VC_2212 | 29.08 | 0.66 | 144.28 | 223.00 | -0.64 | 0.12 | 0.311 |
| VC_2211 | 61.42 | 0.67 | 808.95 | 1135.00 | -0.49 | 0.05 | 0.067 |
| VC_2210 | 29.81 | 0.42 | 287.09 | 315.00 | -0.14 | 0.06 | 0.874 |
| VC_2209 | 176.18 | 1.81 | 3103.07 | 3074.00 | 0.01 | 0.02 | 0.388 |
| VC_2208 | 30.59 | 0.53 | 935.87 | 340.00 | 1.46 | 0.01 | 0.082 |
| VC_2207 | 18.00 | 0.00 | 207.07 | 273.00 | -0.41 | 0.10 | 0.823 |
| VC_2206 | 14.58 | 0.50 | 249.90 | 416.00 | -0.74 | 0.10 | 0.297 |
| VC_2205 | 13.99 | 0.10 | 129.85 | 92.00 | 0.48 | 0.07 | 0.878 |
| VC_2204 | 5.00 | 0.00 | 291.04 | 56.00 | 2.37 | 0.01 | 0.407 |
| VC_2203 | 19.66 | 0.54 | 241.79 | 335.00 | -0.48 | 0.10 | 0.780 |
| VC_2202 | 26.73 | 0.49 | 312.18 | 344.00 | -0.15 | 0.07 | 0.438 |
| VC_2201 | 31.98 | 0.84 | 328.67 | 195.00 | 0.75 | 0.04 | 0.128 |
| VC_2200 | 13.09 | 0.75 | 132.91 | 139.00 | -0.08 | 0.09 | 0.688 |
| VC_2199 | 18.88 | 0.33 | 264.71 | 279.00 | -0.08 | 0.07 | 0.802 |
| VC_2198 | 12.12 | 0.70 | 185.59 | 66.00 | 1.48 | 0.03 | 0.494 |
| VC_2197 | 47.02 | 0.86 | 986.13 | 1093.00 | -0.15 | 0.04 | 0.395 |
| VC_2196 | 8.91 | 0.29 | 159.25 | 52.00 | 1.61 | 0.02 | 0.047 |
| VC_2195 | 14.00 | 0.00 | 547.70 | 307.00 | 0.83 | 0.03 | 0.199 |
| VC_2194 | 22.17 | 0.75 | 239.53 | 320.00 | -0.43 | 0.10 | 0.860 |
| VC_2193 | 19.32 | 0.71 | 460.77 | 189.00 | 1.28 | 0.02 | 0.088 |
| VC_2192 | 21.98 | 0.14 | 200.92 | 133.00 | 0.59 | 0.06 | 0.851 |
| VC_2191 | 38.16 | 0.81 | 493.84 | 739.00 | -0.58 | 0.06 | 0.659 |
| VC_2190 | 31.59 | 0.64 | 421.78 | 337.00 | 0.32 | 0.03 | 0.652 |
| VC_2189 | 2.86 | 0.35 | 21.05 | 5.00 | 1.98 | 0.07 | 0.228 |
| VC_2188 | 27.02 | 0.88 | 624.87 | 325.00 | 0.94 | 0.02 | 0.006 |
| VC_2187 | 28.90 | 0.30 | 424.01 | 585.00 | -0.47 | 0.07 | 0.897 |
| VC_2186 | 4.44 | 0.56 | 8.38 | 32.00 | -2.08 | 1.40 | 0.774 |
| VC_2185 | 23.25 | 0.80 | 231.14 | 166.00 | 0.47 | 0.04 | 0.254 |
| VC_2184 | 1.00 | 0.00 | 3.88 | 2.00 | 0.43 | 0.53 | 1.000 |
| VC_2183 | 3.83 | 0.38 | 18.34 | 7.00 | 1.29 | 0.14 | 0.889 |
| VC_2182 | 2.00 | 0.00 | 14.61 | 1.00 | 3.76 | 0.02 | 1.000 |
| VC_2181 | 1.00 | 0.00 | 1.00 | 1.00 | 0.00 | 0.00 | 1.000 |
| VC_2180 | 3.33 | 0.67 | 9.16 | 2.00 | 2.04 | 0.09 | 0.500 |
| VC_2179 | 0.00 | 0.00 | 1.00 | 1.00 | #VALEUR! | NaN | NaN |
| VC_2178 | 3.85 | 0.36 | 42.11 | 42.00 | -0.03 | 0.16 | 0.713 |

| | | | | | | | |
|---|---|---|---|---|---|---|---|
| VC_2177 | 11.00 | 0.00 | 113.37 | 92.00 | 0.29 | 0.08 | 0.339 |
| VC_2176 | 27.63 | 0.51 | 498.26 | 408.00 | 0.29 | 0.03 | 0.810 |
| VC_2175 | 1.00 | 0.00 | 7.23 | 1.00 | 2.60 | 0.08 | 1.000 |
| VC_2174 | 45.46 | 0.59 | 893.50 | 702.00 | 0.35 | 0.02 | 0.577 |
| VC_2173 | 2.00 | 0.00 | 37.62 | 8.00 | 2.20 | 0.04 | 1.000 |
| VC_2172 | 6.60 | 0.49 | 29.34 | 79.00 | -1.48 | 0.53 | 0.344 |
| VC_2171 | 30.39 | 0.63 | 411.58 | 510.00 | -0.31 | 0.07 | 0.641 |
| VC_2170 | 2.00 | 0.00 | 1.00 | 30.00 | -4.91 | 0.00 | 0.333 |
| VC_2169 | 1.00 | 0.00 | 1.00 | 12.00 | -3.58 | 0.00 | 1.000 |
| VC_2168 | 25.64 | 0.96 | 164.52 | 304.00 | -0.90 | 0.16 | 0.866 |
| VC_2167 | 14.68 | 0.47 | 204.54 | 254.00 | -0.32 | 0.08 | 0.432 |
| VC_2166 | 16.00 | 0.00 | 345.14 | 250.00 | 0.46 | 0.04 | 0.740 |
| VC_2165 | 5.64 | 0.48 | 26.85 | 67.00 | -1.37 | 0.52 | 0.298 |
| VC_2164 | 35.80 | 0.93 | 752.24 | 634.00 | 0.24 | 0.03 | 0.670 |
| VC_2163 | 8.00 | 0.00 | 79.59 | 24.00 | 1.71 | 0.03 | 0.451 |
| VC_2162 | 31.13 | 0.77 | 353.82 | 358.00 | -0.02 | 0.06 | 0.669 |
| VC_2161 | 49.51 | 0.61 | 939.48 | 760.00 | 0.30 | 0.03 | 0.881 |
| VC_2160 | 9.08 | 0.68 | 84.96 | 71.00 | 0.24 | 0.10 | 0.422 |
| VC_2159 | 15.38 | 0.62 | 430.06 | 300.00 | 0.52 | 0.03 | 0.057 |
| VC_2158 | 3.00 | 0.00 | 74.08 | 11.00 | 2.73 | 0.02 | 0.101 |
| VC_2157 | 1.64 | 0.48 | 5.25 | 32.00 | -3.09 | 6.63 | 1.000 |
| VC_2156 | 27.74 | 0.44 | 462.36 | 446.00 | 0.05 | 0.04 | 0.259 |
| VC_2155 | 2.00 | 0.00 | 27.52 | 25.00 | 0.08 | 0.19 | 0.833 |
| VC_2154 | 3.00 | 0.00 | 11.04 | 107.00 | -3.42 | 3.72 | 0.200 |
| VC_2153 | 14.31 | 0.63 | 97.81 | 281.00 | -1.54 | 0.32 | 0.089 |
| VC_2152 | 0.96 | 0.20 | 3.13 | 1.00 | 1.23 | 0.27 | 1.000 |
| VC_2151 | 3.00 | 0.00 | 8.37 | 15.00 | -1.03 | 0.87 | 0.770 |
| VC_2150 | 10.54 | 0.54 | 100.58 | 108.00 | -0.12 | 0.11 | 0.906 |
| VC_2149 | 7.00 | 0.00 | 85.94 | 65.00 | 0.39 | 0.08 | 0.697 |
| VC_2148 | 1.00 | 0.00 | 27.37 | 1.00 | 4.72 | 0.01 | 1.000 |
| VC_2147 | 8.00 | 0.00 | 137.01 | 175.00 | -0.36 | 0.11 | 0.621 |
| VC_2146 | 13.00 | 0.00 | 201.91 | 403.00 | -1.00 | 0.14 | 0.628 |
| VC_2145 | 25.08 | 0.82 | 288.73 | 314.00 | -0.13 | 0.07 | 0.181 |
| VC_2144 | 28.85 | 0.93 | 504.85 | 433.00 | 0.22 | 0.04 | 0.114 |
| VC_2143 | 24.89 | 0.31 | 781.43 | 540.00 | 0.53 | 0.03 | 0.807 |
| VC_2142 | 28.96 | 0.70 | 751.27 | 909.00 | -0.28 | 0.04 | 0.867 |
| VC_2141 | 14.00 | 0.00 | 277.84 | 163.00 | 0.76 | 0.04 | 0.618 |
| VC_2140 | 33.16 | 0.77 | 586.70 | 479.00 | 0.29 | 0.03 | 0.317 |
| VC_2139 | 11.77 | 0.42 | 166.27 | 137.00 | 0.27 | 0.07 | 0.436 |
| VC_2138 | 9.61 | 0.49 | 180.76 | 177.00 | 0.02 | 0.09 | 0.837 |

| | | | | | | | | |
|---|---|---|---|---|---|---|---|---|
| VC_2137 | 26.83 | 0.91 | 277.53 | 316.00 | -0.19 | 0.07 | 0.261 |
| VC_2136 | 19.99 | 0.10 | 420.07 | 208.00 | 1.01 | 0.03 | 0.602 |
| VC_2135 | 37.54 | 0.61 | 611.57 | 481.00 | 0.34 | 0.03 | 0.144 |
| VC_2134 | 9.61 | 0.49 | 125.73 | 112.00 | 0.16 | 0.08 | 0.459 |
| VC_2133 | 38.00 | 0.00 | 762.87 | 803.00 | -0.08 | 0.04 | 0.600 |
| VC_2132 | 19.97 | 0.17 | 205.39 | 317.00 | -0.63 | 0.11 | 0.493 |
| VC_2131 | 12.12 | 0.78 | 96.43 | 148.00 | -0.63 | 0.17 | 0.505 |
| VC_2130 | 26.00 | 0.00 | 530.45 | 542.00 | -0.03 | 0.05 | 0.822 |
| VC_2129 | 10.84 | 0.37 | 179.50 | 219.00 | -0.30 | 0.10 | 0.903 |
| VC_2128 | 30.41 | 0.67 | 323.09 | 352.00 | -0.13 | 0.05 | 0.338 |
| VC_2127 | 9.00 | 0.00 | 89.01 | 179.00 | -1.03 | 0.22 | 0.663 |
| VC_2126 | 21.00 | 0.00 | 250.76 | 304.00 | -0.28 | 0.08 | 0.772 |
| VC_2125 | 7.00 | 0.00 | 77.00 | 40.00 | 0.93 | 0.06 | 0.979 |
| VC_2124 | 8.88 | 0.33 | 135.36 | 128.00 | 0.07 | 0.07 | 0.815 |
| VC_2123 | 18.61 | 0.49 | 126.60 | 173.00 | -0.46 | 0.13 | 0.064 |
| VC_2122 | 7.66 | 0.48 | 151.06 | 172.00 | -0.20 | 0.10 | 0.511 |
| VC_2121 | 16.22 | 0.70 | 148.99 | 168.00 | -0.18 | 0.10 | 0.860 |
| VC_2120 | 24.28 | 0.71 | 514.23 | 538.00 | -0.07 | 0.05 | 0.891 |
| VC_2119 | 11.00 | 0.00 | 367.11 | 140.00 | 1.39 | 0.02 | 0.297 |
| VC_2118 | 20.35 | 0.67 | 520.53 | 309.00 | 0.75 | 0.03 | 0.236 |
| VC_2117 | 3.00 | 0.00 | 77.45 | 14.00 | 2.45 | 0.02 | 0.108 |
| VC_2116 | 23.83 | 0.40 | 297.13 | 480.00 | -0.70 | 0.08 | 0.770 |
| VC_2115 | 15.64 | 0.48 | 222.13 | 314.00 | -0.51 | 0.10 | 0.494 |
| VC_2114 | 7.90 | 0.30 | 53.56 | 88.00 | -0.74 | 0.24 | 0.886 |
| VC_2113 | 15.58 | 0.50 | 97.02 | 123.00 | -0.36 | 0.15 | 0.759 |
| VC_2112 | 2.00 | 0.00 | 42.58 | 8.00 | 2.38 | 0.03 | 1.000 |
| VC_2111 | 14.68 | 0.47 | 345.14 | 167.00 | 1.04 | 0.03 | 0.294 |
| VC_2110 | 27.77 | 0.95 | 461.11 | 854.00 | -0.89 | 0.09 | 0.579 |
| VC_2109 | 0.00 | 0.00 | 1.00 | 1.00 | #VALEUR! | NaN | NaN |
| VC_2108 | 4.00 | 0.00 | 144.90 | 86.00 | 0.74 | 0.05 | 0.755 |
| VC_2107 | 23.22 | 0.69 | 315.86 | 371.00 | -0.24 | 0.07 | 0.912 |
| VC_2106 | 11.86 | 0.35 | 231.17 | 50.00 | 2.20 | 0.02 | 0.020 |
| VC_2105 | 8.93 | 0.95 | 119.06 | 96.00 | 0.30 | 0.08 | 0.811 |
| VC_2104 | 5.92 | 0.27 | 108.82 | 69.00 | 0.65 | 0.06 | 0.237 |
| VC_2103 | 21.88 | 0.33 | 344.55 | 314.00 | 0.13 | 0.05 | 0.794 |
| VC_2102 | 5.85 | 0.36 | 47.13 | 61.00 | -0.40 | 0.20 | 0.549 |
| VC_2101 | 10.17 | 0.71 | 79.09 | 82.00 | -0.07 | 0.11 | 0.604 |
| VC_2100 | 18.51 | 0.54 | 668.42 | 357.00 | 0.90 | 0.02 | 0.103 |
| VC_2099 | 1.00 | 0.00 | 1.51 | 5.00 | -1.99 | 1.35 | 1.000 |
| VC_2098 | 9.00 | 0.00 | 116.10 | 155.00 | -0.43 | 0.13 | 0.897 |

| | | | | | | | |
|---|---|---|---|---|---|---|---|
| VC_2097 | 19.54 | 0.56 | 274.84 | 140.00 | 0.97 | 0.03 | 0.170 |
| VC_2096 | 2.89 | 0.31 | 18.30 | 1.00 | 4.11 | 0.01 | 0.126 |
| VC_2095 | 38.25 | 0.70 | 349.60 | 655.00 | -0.91 | 0.11 | 0.298 |
| VC_2094 | 20.50 | 0.67 | 211.87 | 202.00 | 0.06 | 0.07 | 0.671 |
| VC_2093 | 28.36 | 0.72 | 181.19 | 264.00 | -0.55 | 0.11 | 0.083 |
| VC_2092 | 43.44 | 0.64 | 784.23 | 512.00 | 0.61 | 0.02 | 0.145 |
| VC_2091 | 6.94 | 0.24 | 41.11 | 41.00 | -0.03 | 0.17 | 0.410 |
| VC_2090 | 14.87 | 0.34 | 109.26 | 128.00 | -0.24 | 0.10 | 0.453 |
| VC_2089 | 39.62 | 0.53 | 599.28 | 625.00 | -0.06 | 0.04 | 0.452 |
| VC_2088 | 22.00 | 0.00 | 312.17 | 185.00 | 0.75 | 0.04 | 0.917 |
| VC_2087 | 62.25 | 0.81 | 730.19 | 672.00 | 0.12 | 0.04 | 0.267 |
| VC_2086 | 26.94 | 0.79 | 193.60 | 228.00 | -0.24 | 0.08 | 0.464 |
| VC_2085 | 36.98 | 0.14 | 1036.32 | 939.00 | 0.14 | 0.03 | 0.853 |
| VC_2084 | 23.69 | 0.49 | 279.84 | 349.00 | -0.32 | 0.08 | 0.686 |
| VC_2083 | 19.94 | 0.24 | 228.35 | 276.00 | -0.28 | 0.08 | 0.866 |
| VC_2082 | 22.66 | 0.57 | 459.92 | 323.00 | 0.51 | 0.03 | 0.814 |
| VC_2081 | 33.78 | 0.42 | 464.71 | 346.00 | 0.42 | 0.03 | 0.398 |
| VC_2080 | 114.47 | 1.18 | 1314.20 | 1477.00 | -0.17 | 0.03 | 0.866 |
| VC_2079 | 6.00 | 0.00 | 77.01 | 77.00 | -0.02 | 0.13 | 0.625 |
| VC_2078 | 4.59 | 0.49 | 111.03 | 93.00 | 0.24 | 0.08 | 0.889 |
| VC_2077 | 79.03 | 1.00 | 1467.05 | 1621.00 | -0.14 | 0.03 | 0.631 |
| VC_2076 | 4.94 | 0.24 | 164.38 | 221.00 | -0.43 | 0.10 | 0.882 |
| VC_2075 | 11.55 | 0.63 | 93.22 | 54.00 | 0.78 | 0.05 | 0.084 |
| VC_2074 | 1.00 | 0.00 | 1.00 | 4.00 | -2.00 | 0.00 | 1.000 |
| VC_2073 | 10.00 | 0.00 | 116.13 | 104.00 | 0.15 | 0.08 | 0.676 |
| VC_2072 | 78.18 | 1.10 | 1105.94 | 1088.00 | 0.02 | 0.03 | 0.900 |
| VC_2071 | 6.30 | 0.63 | 129.96 | 134.00 | -0.06 | 0.10 | 0.885 |
| VC_2070 | 9.00 | 0.00 | 221.29 | 96.00 | 1.20 | 0.03 | 0.548 |
| VC_2069 | 43.58 | 0.55 | 717.31 | 1169.00 | -0.71 | 0.06 | 0.184 |
| VC_2068 | 34.28 | 0.70 | 459.67 | 704.00 | -0.62 | 0.07 | 0.439 |
| VC_2067 | 25.62 | 0.49 | 397.69 | 225.00 | 0.82 | 0.03 | 0.354 |
| VC_2066 | 22.65 | 0.48 | 277.19 | 321.00 | -0.22 | 0.07 | 0.575 |
| VC_2065 | 10.00 | 0.00 | 85.43 | 170.00 | -1.01 | 0.21 | 0.039 |
| VC_2064 | 15.98 | 0.14 | 179.01 | 123.00 | 0.53 | 0.06 | 0.634 |
| VC_2063 | 77.28 | 1.05 | 1214.83 | 1322.00 | -0.12 | 0.03 | 0.688 |
| VC_2062 | 35.32 | 0.69 | 633.21 | 595.00 | 0.09 | 0.04 | 0.858 |
| VC_2061 | 28.14 | 0.78 | 636.71 | 330.00 | 0.95 | 0.02 | 0.006 |
| VC_2060 | 26.47 | 0.64 | 431.91 | 292.00 | 0.56 | 0.04 | 0.643 |
| VC_2059 | 13.62 | 0.49 | 348.89 | 229.00 | 0.60 | 0.04 | 0.233 |
| VC_2058 | 8.00 | 0.00 | 144.70 | 130.00 | 0.15 | 0.07 | 0.525 |

| | | | | | | | |
|---|---|---|---|---|---|---|---|
| VC_2057 | 12.97 | 0.17 | 53.73 | 211.00 | -2.01 | 0.62 | 0.102 |
| VC_2056 | 15.46 | 0.58 | 80.10 | 103.00 | -0.38 | 0.14 | 0.795 |
| VC_2055 | 15.97 | 0.17 | 207.17 | 253.00 | -0.30 | 0.09 | 0.600 |
| VC_2054 | 6.00 | 0.00 | 43.79 | 75.00 | -0.82 | 0.30 | 0.096 |
| VC_2053 | 13.44 | 0.61 | 155.53 | 105.00 | 0.56 | 0.05 | 0.720 |
| VC_2052 | 49.52 | 0.64 | 942.11 | 698.00 | 0.43 | 0.03 | 0.182 |
| VC_2051 | 14.20 | 1.11 | 221.90 | 102.00 | 1.12 | 0.03 | 0.875 |
| VC_2049 | 31.05 | 0.77 | 413.52 | 632.00 | -0.62 | 0.08 | 0.271 |
| VC_2048 | 34.07 | 0.78 | 277.10 | 453.00 | -0.71 | 0.10 | 0.366 |
| VC_2047 | 20.42 | 0.65 | 339.81 | 103.00 | 1.72 | 0.02 | 0.024 |
| VC_2046 | 17.00 | 0.00 | 154.18 | 362.00 | -1.24 | 0.21 | 0.171 |
| VC_2045 | 2.59 | 0.53 | 3.22 | 2.00 | 0.23 | 0.53 | 0.805 |
| VC_2044 | 5.71 | 0.46 | 98.76 | 44.00 | 1.15 | 0.05 | 0.312 |
| VC_2043 | 43.37 | 1.18 | 501.08 | 373.00 | 0.42 | 0.04 | 0.576 |
| VC_2042 | 21.21 | 0.71 | 409.89 | 279.00 | 0.55 | 0.03 | 0.109 |
| VC_2041 | 33.44 | 0.67 | 585.00 | 272.00 | 1.10 | 0.02 | 0.569 |
| VC_2040 | 7.57 | 0.50 | 81.92 | 159.00 | -0.98 | 0.24 | 0.492 |
| VC_2039 | 27.92 | 0.80 | 172.47 | 225.00 | -0.39 | 0.10 | 0.407 |
| VC_2038 | 25.34 | 0.67 | 281.17 | 177.00 | 0.66 | 0.04 | 0.697 |
| VC_2037 | 43.90 | 0.30 | 762.33 | 1154.00 | -0.60 | 0.05 | 0.063 |
| VC_2036 | 19.65 | 0.48 | 306.14 | 187.00 | 0.71 | 0.04 | 0.451 |
| VC_2035 | 13.64 | 0.48 | 308.11 | 272.00 | 0.17 | 0.06 | 0.914 |
| VC_2034 | 0.00 | 0.00 | 1.00 | 1.00 | #VALEUR! | NaN | NaN |
| VC_2033 | 84.62 | 1.03 | 1203.24 | 1713.00 | -0.51 | 0.04 | 0.805 |
| VC_2032 | 26.52 | 0.59 | 136.73 | 400.00 | -1.56 | 0.27 | 0.006 |
| VC_2031 | 37.18 | 0.70 | 285.92 | 304.00 | -0.09 | 0.06 | 0.553 |
| VC_2030 | 30.56 | 0.54 | 579.08 | 723.00 | -0.32 | 0.05 | 0.113 |
| VC_2029 | 4.58 | 0.54 | 41.33 | 50.00 | -0.31 | 0.22 | 0.902 |
| VC_2028 | 23.57 | 0.52 | 304.43 | 446.00 | -0.56 | 0.10 | 0.477 |
| VC_2027 | 20.26 | 0.65 | 87.02 | 220.00 | -1.36 | 0.30 | 0.122 |
| VC_2026 | 6.27 | 0.68 | 16.27 | 35.00 | -1.19 | 0.62 | 0.163 |
| VC_2025 | 4.83 | 0.38 | 12.33 | 6.00 | 0.88 | 0.21 | 0.592 |
| VC_2024 | 5.43 | 0.61 | 9.89 | 62.00 | -2.84 | 3.12 | 0.250 |
| VC_2023 | 3.66 | 0.48 | 9.05 | 6.00 | 0.38 | 0.36 | 0.949 |
| VC_2022 | 0.66 | 0.48 | 1.42 | 1.00 | 0.40 | 0.28 | 1.000 |
| VC_2021 | 1.00 | 0.00 | 73.15 | 4.00 | 4.18 | 0.01 | 1.000 |
| VC_2020 | 0.00 | 0.00 | 1.00 | 1.00 | #VALEUR! | NaN | NaN |
| VC_2019 | 32.30 | 0.64 | 551.18 | 645.00 | -0.23 | 0.05 | 0.861 |
| VC_2018 | 30.60 | 0.53 | 387.56 | 519.00 | -0.43 | 0.07 | 0.304 |
| VC_2017 | 17.45 | 0.56 | 166.41 | 119.00 | 0.48 | 0.05 | 0.887 |

| | | | | | | | |
|---|---|---|---|---|---|---|---|
| VC_2016 | 0.00 | 0.00 | 1.00 | 1.00 | #VALEUR! | NaN | NaN |
| VC_2015 | 1.69 | 0.46 | 14.92 | 1.00 | 3.78 | 0.02 | 0.540 |
| VC_2014 | 18.62 | 0.49 | 263.42 | 362.00 | -0.46 | 0.08 | 0.233 |
| VC_2013 | 55.04 | 0.83 | 1182.01 | 1245.00 | -0.08 | 0.03 | 0.733 |
| VC_2012 | 53.87 | 0.34 | 835.24 | 669.00 | 0.32 | 0.03 | 0.873 |
| VC_2011 | 23.98 | 0.14 | 322.27 | 227.00 | 0.50 | 0.04 | 0.744 |
| VC_2010 | 4.00 | 0.00 | 39.84 | 30.00 | 0.38 | 0.12 | 0.791 |
| VC_2009 | 42.51 | 0.95 | 363.83 | 524.00 | -0.53 | 0.09 | 0.487 |
| VC_2008 | 31.84 | 0.37 | 386.71 | 572.00 | -0.57 | 0.07 | 0.117 |
| VC_2007 | 35.87 | 0.34 | 401.16 | 292.00 | 0.45 | 0.04 | 0.730 |
| VC_2006 | 37.70 | 0.52 | 476.15 | 265.00 | 0.84 | 0.03 | 0.166 |
| VC_2005 | 11.79 | 0.41 | 118.15 | 211.00 | -0.85 | 0.15 | 0.574 |
| VC_2004 | 27.56 | 0.52 | 280.58 | 298.00 | -0.09 | 0.07 | 0.585 |
| VC_2003 | 19.24 | 0.70 | 472.74 | 322.00 | 0.55 | 0.03 | 0.302 |
| VC_2002 | 28.86 | 0.35 | 273.96 | 340.00 | -0.32 | 0.07 | 0.430 |
| VC_2001 | 22.59 | 0.55 | 512.26 | 304.00 | 0.75 | 0.03 | 0.073 |
| VC_2000 | 2.00 | 0.00 | 7.89 | 52.00 | -3.07 | 6.99 | 0.377 |
| VC_1999 | 4.99 | 0.10 | 88.82 | 124.00 | -0.50 | 0.15 | 0.539 |
| VC_1998 | 12.00 | 0.00 | 124.07 | 115.00 | 0.10 | 0.08 | 0.733 |
| VC_1997 | 24.94 | 0.24 | 325.39 | 554.00 | -0.77 | 0.10 | 0.133 |
| VC_1996 | 14.69 | 0.56 | 148.47 | 301.00 | -1.03 | 0.17 | 0.164 |
| VC_1995 | 29.24 | 0.71 | 263.48 | 289.00 | -0.14 | 0.06 | 0.577 |
| VC_1994 | 46.53 | 0.56 | 813.94 | 566.00 | 0.52 | 0.03 | 0.761 |
| VC_1993 | 32.00 | 0.77 | 324.34 | 379.00 | -0.23 | 0.07 | 0.863 |
| VC_1992 | 22.54 | 0.61 | 397.97 | 301.00 | 0.40 | 0.04 | 0.036 |
| VC_1991 | 13.00 | 0.00 | 118.41 | 167.00 | -0.51 | 0.13 | 0.888 |
| VC_1990 | 38.07 | 0.88 | 659.74 | 542.00 | 0.28 | 0.03 | 0.890 |
| VC_1989 | 1.00 | 0.00 | 1.00 | 3.00 | -1.58 | 0.00 | 1.000 |
| VC_1988 | 11.81 | 0.39 | 65.89 | 92.00 | -0.51 | 0.21 | 0.012 |
| VC_1987 | 15.62 | 0.49 | 293.14 | 549.00 | -0.91 | 0.11 | 0.535 |
| VC_1986 | 17.80 | 0.92 | 559.05 | 420.00 | 0.41 | 0.03 | 0.345 |
| VC_1985 | 30.88 | 0.33 | 629.37 | 817.00 | -0.38 | 0.06 | 0.091 |
| VC_1984 | 22.95 | 0.22 | 219.79 | 288.00 | -0.40 | 0.09 | 0.078 |
| VC_1983 | 47.79 | 0.88 | 568.14 | 694.00 | -0.29 | 0.05 | 0.767 |
| VC_1982 | 5.95 | 0.22 | 65.61 | 86.00 | -0.42 | 0.18 | 0.133 |
| VC_1981 | 7.29 | 0.67 | 93.50 | 27.00 | 1.78 | 0.03 | 0.119 |
| VC_1980 | 10.66 | 0.48 | 109.87 | 312.00 | -1.52 | 0.25 | 0.037 |
| VC_1979 | 26.46 | 0.64 | 653.82 | 365.00 | 0.84 | 0.02 | 0.173 |
| VC_1978 | 13.50 | 0.64 | 215.84 | 112.00 | 0.94 | 0.04 | 0.324 |
| VC_1977 | 30.19 | 0.77 | 707.80 | 612.00 | 0.21 | 0.03 | 0.549 |

| | | | | | | | |
|---|---|---|---|---|---|---|---|
| VC_1976 | 24.59 | 0.55 | 378.39 | 296.00 | 0.35 | 0.04 | 0.154 |
| VC_1975 | 31.53 | 0.61 | 375.42 | 455.00 | -0.28 | 0.07 | 0.255 |
| VC_1974 | 16.89 | 0.31 | 502.67 | 229.00 | 1.13 | 0.02 | 0.474 |
| VC_1973 | 10.55 | 0.54 | 258.38 | 483.00 | -0.91 | 0.11 | 0.492 |
| VC_1972 | 19.94 | 0.24 | 246.71 | 168.00 | 0.55 | 0.04 | 0.870 |
| VC_1971 | 30.54 | 0.58 | 359.55 | 469.00 | -0.39 | 0.07 | 0.675 |
| VC_1970 | 25.25 | 0.73 | 310.12 | 330.00 | -0.09 | 0.06 | 0.728 |
| VC_1969 | 4.57 | 0.54 | 19.89 | 7.00 | 1.42 | 0.10 | 0.241 |
| VC_1968 | 12.97 | 0.17 | 155.49 | 75.00 | 1.04 | 0.04 | 0.617 |
| VC_1967 | 80.24 | 1.15 | 949.98 | 726.00 | 0.39 | 0.02 | 0.766 |
| VC_1966 | 47.75 | 1.10 | 618.21 | 468.00 | 0.40 | 0.03 | 0.523 |
| VC_1965 | 26.00 | 0.00 | 149.04 | 534.00 | -1.85 | 0.30 | 0.002 |
| VC_1964 | 18.99 | 0.10 | 488.70 | 475.00 | 0.04 | 0.04 | 0.720 |
| VC_1963 | 10.99 | 0.10 | 213.05 | 83.00 | 1.35 | 0.03 | 0.413 |
| VC_1962 | 8.98 | 0.14 | 221.87 | 139.00 | 0.67 | 0.05 | 0.916 |
| VC_1961 | 1.66 | 0.48 | 27.54 | 1.00 | 4.73 | 0.01 | 0.560 |
| VC_1960 | 10.83 | 0.38 | 80.56 | 234.00 | -1.55 | 0.31 | 0.186 |
| VC_1959 | 16.26 | 0.72 | 112.69 | 263.00 | -1.24 | 0.26 | 0.048 |
| VC_1958 | 4.00 | 0.00 | 76.88 | 23.00 | 1.72 | 0.03 | 0.324 |
| VC_1957 | 8.00 | 0.00 | 54.37 | 83.00 | -0.64 | 0.25 | 0.166 |
| VC_1956 | 31.72 | 0.53 | 685.16 | 436.00 | 0.65 | 0.02 | 0.028 |
| VC_1955 | 19.58 | 0.57 | 249.95 | 319.00 | -0.36 | 0.10 | 0.257 |
| VC_1954 | 1.90 | 0.30 | 7.42 | 1.00 | 2.65 | 0.08 | 0.400 |
| VC_1953 | 27.65 | 0.97 | 461.25 | 705.00 | -0.61 | 0.07 | 0.268 |
| VC_1952 | 67.24 | 1.17 | 898.13 | 1139.00 | -0.34 | 0.04 | 0.316 |
| VC_1951 | 37.78 | 0.44 | 664.21 | 702.00 | -0.08 | 0.05 | 0.736 |
| VC_1950 | 56.76 | 1.11 | 855.09 | 678.00 | 0.33 | 0.03 | 0.020 |
| VC_1949 | 52.72 | 0.83 | 848.76 | 809.00 | 0.07 | 0.03 | 0.707 |
| VC_1948 | 2.56 | 0.50 | 10.57 | 1.00 | 3.24 | 0.04 | 0.664 |
| VC_1947 | 33.40 | 1.02 | 274.70 | 305.00 | -0.16 | 0.08 | 0.782 |
| VC_1946 | 1.88 | 0.33 | 17.65 | 34.00 | -1.06 | 0.72 | 1.000 |
| VC_1945 | 36.32 | 1.38 | 720.81 | 810.00 | -0.17 | 0.05 | 0.453 |
| VC_1944 | 29.16 | 0.79 | 347.81 | 375.00 | -0.11 | 0.06 | 0.769 |
| VC_1943 | 1.00 | 0.00 | 22.88 | 1.00 | 4.42 | 0.01 | 1.000 |
| VC_1942 | 20.96 | 0.20 | 194.64 | 458.00 | -1.24 | 0.20 | 0.148 |
| VC_1941 | 46.56 | 0.50 | 744.39 | 680.00 | 0.13 | 0.03 | 0.636 |
| VC_1940 | 18.50 | 0.58 | 310.95 | 236.00 | 0.39 | 0.04 | 0.063 |
| VC_1939 | 23.20 | 0.67 | 252.83 | 448.00 | -0.83 | 0.11 | 0.890 |
| VC_1938 | 16.00 | 0.00 | 186.45 | 153.00 | 0.28 | 0.07 | 0.616 |
| VC_1937 | 18.39 | 1.04 | 287.51 | 123.00 | 1.22 | 0.03 | 0.517 |

| | | | | | | | |
|---|---|---|---|---|---|---|---|
| VC_1936 | 32.50 | 0.56 | 663.51 | 404.00 | 0.71 | 0.03 | 0.871 |
| VC_1935 | 24.50 | 0.59 | 423.81 | 364.00 | 0.22 | 0.05 | 0.884 |
| VC_1934 | 69.86 | 0.86 | 960.91 | 859.00 | 0.16 | 0.03 | 0.280 |
| VC_1933 | 17.78 | 0.42 | 131.00 | 157.00 | -0.27 | 0.10 | 0.169 |
| VC_1932 | 10.00 | 0.00 | 118.89 | 73.00 | 0.69 | 0.06 | 0.741 |
| VC_1931 | 32.61 | 0.49 | 377.46 | 811.00 | -1.11 | 0.13 | 0.084 |
| VC_1930 | 0.00 | 0.00 | 1.00 | 1.00 | #VALEUR! | NaN | NaN |
| VC_1929 | 19.14 | 0.78 | 165.88 | 367.00 | -1.16 | 0.18 | 0.379 |
| VC_1928 | 18.82 | 0.39 | 265.52 | 351.00 | -0.41 | 0.08 | 0.200 |
| VC_1927 | 31.63 | 0.49 | 410.21 | 944.00 | -1.21 | 0.11 | 0.148 |
| VC_1926 | 32.97 | 0.17 | 257.58 | 696.00 | -1.44 | 0.16 | 0.183 |
| VC_1925 | 40.65 | 0.95 | 431.51 | 780.00 | -0.86 | 0.09 | 0.610 |
| VC_1924 | 10.97 | 0.17 | 42.26 | 69.00 | -0.74 | 0.27 | 0.409 |
| VC_1923 | 20.33 | 0.65 | 59.60 | 201.00 | -1.77 | 0.41 | 0.004 |
| VC_1922 | 12.65 | 0.48 | 93.36 | 134.00 | -0.54 | 0.17 | 0.340 |
| VC_1921 | 16.54 | 0.58 | 179.83 | 64.00 | 1.48 | 0.03 | 0.769 |
| VC_1920 | 43.16 | 0.79 | 684.21 | 568.00 | 0.27 | 0.03 | 0.481 |
| VC_1919 | 8.00 | 0.00 | 51.31 | 108.00 | -1.10 | 0.30 | 0.421 |
| VC_1918 | 49.36 | 1.11 | 602.29 | 635.00 | -0.08 | 0.04 | 0.205 |
| VC_1917 | 10.83 | 0.38 | 126.45 | 100.00 | 0.33 | 0.08 | 0.736 |
| VC_1916 | 2.00 | 0.00 | 44.55 | 1.00 | 5.44 | 0.00 | 0.333 |
| VC_1915 | 1.00 | 0.00 | 1.00 | 1.00 | 0.00 | 0.00 | 1.000 |
| VC_1914 | 10.51 | 0.52 | 242.23 | 109.00 | 1.15 | 0.03 | 0.351 |
| VC_1913 | 0.00 | 0.00 | 1.00 | 1.00 | #VALEUR! | NaN | NaN |
| VC_1912 | 1.00 | 0.00 | 1.00 | 9.00 | -3.17 | 0.00 | 1.000 |
| VC_1911 | 15.99 | 0.10 | 93.83 | 79.00 | 0.23 | 0.10 | 0.197 |
| VC_1910 | 27.17 | 0.75 | 292.42 | 230.00 | 0.34 | 0.04 | 0.492 |
| VC_1909 | 24.31 | 0.68 | 283.05 | 542.00 | -0.94 | 0.12 | 0.770 |
| VC_1908 | 4.50 | 0.58 | 4.18 | 10.00 | -1.83 | 2.53 | 0.616 |
| VC_1907 | 4.60 | 0.53 | 9.87 | 7.00 | 0.28 | 0.42 | 0.706 |
| VC_1906 | 27.78 | 0.44 | 250.61 | 306.00 | -0.29 | 0.07 | 0.794 |
| VC_1905 | 49.01 | 0.82 | 813.47 | 665.00 | 0.29 | 0.03 | 0.868 |
| VC_1904 | 8.24 | 0.77 | 61.72 | 11.00 | 2.46 | 0.03 | 0.003 |
| VC_1903 | 21.34 | 0.61 | 90.94 | 257.00 | -1.52 | 0.33 | 0.115 |
| VC_1902 | 18.86 | 0.35 | 428.20 | 399.00 | 0.10 | 0.04 | 0.389 |
| VC_1901 | 40.18 | 1.03 | 428.68 | 313.00 | 0.45 | 0.04 | 0.553 |
| VC_1900 | 11.35 | 0.73 | 17.76 | 41.00 | -1.32 | 0.82 | 0.066 |
| VC_1899 | 46.99 | 0.10 | 628.99 | 617.00 | 0.03 | 0.04 | 0.341 |
| VC_1898 | 34.99 | 0.10 | 252.74 | 863.00 | -1.78 | 0.23 | 0.014 |
| VC_1897 | 7.98 | 0.14 | 73.80 | 51.00 | 0.51 | 0.09 | 0.363 |

| | | | | | | | |
|---|---|---|---|---|---|---|---|
| VC_1896 | 45.34 | 0.70 | 586.88 | 574.00 | 0.03 | 0.04 | 0.685 |
| VC_1895 | 13.61 | 0.49 | 184.08 | 223.00 | -0.28 | 0.09 | 0.764 |
| VC_1894 | 16.98 | 0.14 | 429.98 | 547.00 | -0.35 | 0.06 | 0.013 |
| VC_1893 | 19.90 | 0.88 | 138.95 | 328.00 | -1.25 | 0.19 | 0.125 |
| VC_1892 | 15.98 | 0.14 | 398.71 | 127.00 | 1.65 | 0.01 | 0.461 |
| VC_1891 | 3.00 | 0.00 | 8.38 | 9.00 | -0.40 | 1.14 | 0.886 |
| VC_1890 | 27.96 | 0.20 | 249.27 | 462.00 | -0.89 | 0.11 | 0.089 |
| VC_1889 | 7.58 | 0.52 | 29.37 | 48.00 | -0.76 | 0.33 | 0.623 |
| VC_1888 | 63.31 | 0.71 | 1836.48 | 938.00 | 0.97 | 0.01 | 0.030 |
| VC_1887 | 12.66 | 0.50 | 67.66 | 172.00 | -1.37 | 0.35 | 0.064 |
| VC_1886 | 71.07 | 1.06 | 1225.09 | 1121.00 | 0.13 | 0.02 | 0.579 |
| VC_1885 | 10.65 | 0.48 | 238.10 | 220.00 | 0.11 | 0.07 | 0.610 |
| VC_1884 | 0.00 | 0.00 | 1.00 | 1.00 | #VALEUR! | NaN | NaN |
| VC_1883 | 0.00 | 0.00 | 1.00 | 1.00 | #VALEUR! | NaN | NaN |
| VC_1882 | 0.00 | 0.00 | 1.00 | 1.00 | #VALEUR! | NaN | NaN |
| VC_1881 | 2.98 | 0.14 | 37.90 | 16.00 | 1.20 | 0.08 | 0.339 |
| VC_1880 | 14.00 | 0.00 | 363.52 | 225.00 | 0.69 | 0.03 | 0.474 |
| VC_1879 | 63.66 | 0.54 | 1012.24 | 819.00 | 0.30 | 0.03 | 0.156 |
| VC_1878 | 0.00 | 0.00 | 1.00 | 1.00 | #VALEUR! | NaN | NaN |
| VC_1877 | 1.56 | 0.50 | 29.41 | 1.00 | 4.83 | 0.01 | 0.627 |
| VC_1876 | 2.99 | 0.10 | 29.00 | 1.00 | 4.81 | 0.01 | 0.102 |
| VC_1875 | 1.00 | 0.00 | 1.00 | 62.00 | -5.95 | 0.00 | 1.000 |
| VC_1874 | 39.20 | 0.75 | 361.75 | 840.00 | -1.22 | 0.12 | 0.000 |
| VC_1873 | 26.99 | 0.10 | 377.29 | 379.00 | -0.01 | 0.05 | 0.490 |
| VC_1872 | 43.36 | 1.05 | 642.07 | 495.00 | 0.37 | 0.03 | 0.512 |
| VC_1871 | 10.35 | 0.67 | 269.37 | 286.00 | -0.09 | 0.07 | 0.866 |
| VC_1870 | 16.89 | 0.84 | 303.47 | 298.00 | 0.02 | 0.06 | 0.870 |
| VC_1869 | 11.88 | 0.33 | 210.62 | 25.00 | 3.07 | 0.01 | 0.003 |
| VC_1868 | 40.24 | 1.40 | 491.43 | 509.00 | -0.05 | 0.06 | 0.784 |
| VC_1867 | 34.58 | 0.55 | 596.03 | 595.00 | 0.00 | 0.04 | 0.603 |
| VC_1866 | 75.73 | 1.27 | 1421.59 | 1644.00 | -0.21 | 0.03 | 0.802 |
| VC_1865 | 32.58 | 0.52 | 657.35 | 494.00 | 0.41 | 0.03 | 0.563 |
| VC_1864 | 18.17 | 0.70 | 141.44 | 276.00 | -0.98 | 0.17 | 0.100 |
| VC_1863 | 33.48 | 0.61 | 471.58 | 466.00 | 0.01 | 0.05 | 0.343 |
| VC_1862 | 24.66 | 1.02 | 708.02 | 389.00 | 0.86 | 0.02 | 0.297 |
| VC_1861 | 26.11 | 1.27 | 306.19 | 153.00 | 1.00 | 0.03 | 0.271 |
| VC_1860 | 13.38 | 0.62 | 60.79 | 275.00 | -2.20 | 0.57 | 0.108 |
| VC_1859 | 41.81 | 0.39 | 557.23 | 723.00 | -0.38 | 0.05 | 0.470 |
| VC_1858 | 2.00 | 0.00 | 1.00 | 58.00 | -5.86 | 0.00 | 0.333 |
| VC_1857 | 10.70 | 0.50 | 216.75 | 44.00 | 2.29 | 0.01 | 0.264 |

| | | | | | | | |
|---|---|---|---|---|---|---|---|
| VC_1856 | 21.88 | 0.33 | 226.22 | 530.00 | -1.24 | 0.18 | 0.058 |
| VC_1855 | 44.48 | 0.67 | 670.30 | 882.00 | -0.40 | 0.06 | 0.926 |
| VC_1854 | 44.90 | 0.33 | 960.51 | 1018.00 | -0.09 | 0.04 | 0.724 |
| VC_1853 | 29.74 | 0.46 | 240.64 | 230.00 | 0.06 | 0.05 | 0.529 |
| VC_1852 | 7.90 | 0.30 | 120.87 | 149.00 | -0.32 | 0.12 | 0.652 |
| VC_1851 | 31.94 | 0.24 | 385.60 | 313.00 | 0.30 | 0.04 | 0.070 |
| VC_1850 | 2.00 | 0.00 | 1.00 | 15.00 | -3.91 | 0.00 | 0.333 |
| VC_1849 | 9.75 | 0.48 | 127.02 | 257.00 | -1.03 | 0.18 | 0.887 |
| VC_1848 | 2.00 | 0.00 | 88.23 | 89.00 | -0.03 | 0.11 | 0.920 |
| VC_1847 | 12.73 | 0.47 | 152.54 | 121.00 | 0.32 | 0.07 | 0.560 |
| VC_1846 | 11.84 | 0.39 | 119.96 | 47.00 | 1.34 | 0.04 | 0.485 |
| VC_1845 | 13.82 | 0.39 | 47.19 | 221.00 | -2.26 | 0.75 | 0.020 |
| VC_1844 | 3.00 | 0.00 | 45.45 | 15.00 | 1.57 | 0.05 | 1.000 |
| VC_1843 | 4.74 | 0.46 | 15.50 | 4.00 | 1.86 | 0.07 | 0.525 |
| VC_1842 | 2.96 | 0.20 | 3.06 | 48.00 | -4.48 | 13.99 | 0.397 |
| VC_1841 | 14.00 | 0.00 | 286.57 | 305.00 | -0.09 | 0.06 | 0.714 |
| VC_1840 | 9.90 | 0.30 | 139.20 | 232.00 | -0.75 | 0.17 | 0.457 |
| VC_1839 | 9.00 | 0.00 | 7.15 | 72.00 | -3.52 | 5.14 | 0.001 |
| VC_1838 | 9.00 | 0.00 | 24.33 | 109.00 | -2.23 | 1.02 | 0.039 |
| VC_1837 | 7.00 | 0.00 | 10.11 | 93.00 | -3.35 | 4.48 | 0.011 |
| VC_1836 | 17.94 | 0.24 | 69.33 | 158.00 | -1.21 | 0.25 | 0.027 |
| VC_1835 | 6.99 | 0.10 | 61.85 | 31.00 | 0.97 | 0.07 | 0.774 |
| VC_1834 | 18.95 | 0.22 | 236.40 | 199.00 | 0.24 | 0.06 | 0.560 |
| VC_1833 | 30.90 | 0.30 | 630.27 | 516.00 | 0.29 | 0.03 | 0.360 |
| VC_1832 | 15.85 | 0.36 | 436.38 | 248.00 | 0.81 | 0.03 | 0.784 |
| VC_1831 | 55.84 | 0.37 | 889.68 | 780.00 | 0.19 | 0.03 | 0.199 |
| VC_1830 | 1.00 | 0.00 | 28.40 | 1.00 | 4.78 | 0.01 | 1.000 |
| VC_1829 | 1.00 | 0.00 | 1.00 | 2.00 | -1.00 | 0.00 | 1.000 |
| VC_1828 | 12.00 | 0.00 | 109.69 | 214.00 | -0.98 | 0.20 | 0.572 |
| VC_1827 | 35.66 | 0.50 | 494.70 | 460.00 | 0.10 | 0.04 | 0.177 |
| VC_1826 | 27.79 | 0.41 | 266.57 | 270.00 | -0.03 | 0.07 | 0.927 |
| VC_1825 | 30.79 | 0.43 | 373.88 | 448.00 | -0.26 | 0.06 | 0.479 |
| VC_1824 | 18.97 | 0.17 | 351.63 | 201.00 | 0.80 | 0.04 | 0.420 |
| VC_1823 | 6.00 | 0.00 | 113.94 | 286.00 | -1.34 | 0.25 | 0.248 |
| VC_1822 | 35.76 | 0.47 | 765.66 | 538.00 | 0.51 | 0.03 | 0.130 |
| VC_1821 | 32.05 | 0.95 | 556.15 | 497.00 | 0.16 | 0.04 | 0.064 |
| VC_1820 | 10.00 | 0.00 | 303.55 | 46.00 | 2.72 | 0.01 | 0.863 |
| VC_1819 | 34.85 | 0.39 | 612.29 | 527.00 | 0.21 | 0.04 | 0.466 |
| VC_1818 | 1.85 | 0.36 | 2.21 | 116.00 | -6.20 | 37.29 | 1.000 |
| VC_1817 | 36.54 | 0.56 | 415.60 | 527.00 | -0.35 | 0.06 | 0.627 |

| | | | | | | | |
|---|---|---|---|---|---|---|---|
| VC_1816 | 8.89 | 0.31 | 126.39 | 174.00 | -0.47 | 0.13 | 0.608 |
| VC_1815 | 17.73 | 0.49 | 690.03 | 286.00 | 1.27 | 0.02 | 0.025 |
| VC_1814 | 34.76 | 0.45 | 331.48 | 433.00 | -0.39 | 0.07 | 0.686 |
| VC_1813 | 2.00 | 0.00 | 38.92 | 16.00 | 1.25 | 0.06 | 1.000 |
| VC_1812 | 10.89 | 0.31 | 87.28 | 267.00 | -1.63 | 0.36 | 0.225 |
| VC_1811 | 9.99 | 0.10 | 179.40 | 177.00 | 0.01 | 0.07 | 0.906 |
| VC_1810 | 3.93 | 0.26 | 92.28 | 19.00 | 2.26 | 0.02 | 0.807 |
| VC_1809 | 5.94 | 0.24 | 200.44 | 115.00 | 0.80 | 0.04 | 0.331 |
| VC_1808 | 11.95 | 0.22 | 32.20 | 88.00 | -1.50 | 0.54 | 0.034 |
| VC_1807 | 9.77 | 0.45 | 29.65 | 26.00 | 0.14 | 0.17 | 0.603 |
| VC_1806 | 32.70 | 0.54 | 369.43 | 316.00 | 0.22 | 0.04 | 0.570 |
| VC_1805 | 11.99 | 0.10 | 198.91 | 265.00 | -0.42 | 0.09 | 0.764 |
| VC_1804 | 7.80 | 0.45 | 51.67 | 103.00 | -1.02 | 0.26 | 0.287 |
| VC_1803 | 0.00 | 0.00 | 1.00 | 1.00 | #VALEUR! | NaN | NaN |
| VC_1802 | 0.00 | 0.00 | 1.00 | 1.00 | #VALEUR! | NaN | NaN |
| VC_1801 | 0.78 | 0.42 | 2.13 | 1.00 | 0.80 | 0.31 | 1.000 |
| VC_1800 | 11.92 | 0.27 | 46.06 | 249.00 | -2.46 | 0.74 | 0.088 |
| VC_1799 | 32.31 | 0.76 | 519.00 | 542.00 | -0.07 | 0.05 | 0.149 |
| VC_1798 | 35.99 | 0.10 | 635.87 | 930.00 | -0.55 | 0.06 | 0.187 |
| VC_1797 | 13.89 | 0.31 | 183.34 | 240.00 | -0.40 | 0.10 | 0.716 |
| VC_1796 | 16.00 | 0.00 | 275.84 | 318.00 | -0.21 | 0.07 | 0.903 |
| VC_1795 | 9.78 | 0.42 | 70.15 | 78.00 | -0.17 | 0.14 | 0.713 |
| VC_1794 | 21.76 | 0.45 | 280.33 | 178.00 | 0.65 | 0.04 | 0.222 |
| VC_1793 | 19.72 | 0.47 | 306.45 | 343.00 | -0.17 | 0.07 | 0.749 |
| VC_1792 | 15.89 | 0.31 | 276.42 | 167.00 | 0.72 | 0.04 | 0.593 |
| VC_1791 | 38.61 | 0.60 | 667.06 | 474.00 | 0.49 | 0.02 | 0.554 |
| VC_1790 | 16.98 | 0.14 | 247.04 | 99.00 | 1.31 | 0.03 | 0.058 |
| VC_1789 | 42.67 | 0.53 | 512.49 | 490.00 | 0.06 | 0.04 | 0.878 |
| VC_1788 | 35.68 | 0.55 | 301.51 | 231.00 | 0.38 | 0.04 | 0.819 |
| VC_1787 | 3.84 | 0.37 | 49.39 | 87.00 | -0.84 | 0.25 | 0.617 |
| VC_1786 | 19.83 | 0.38 | 334.41 | 555.00 | -0.73 | 0.08 | 0.361 |
| VC_1785 | 2.94 | 0.24 | 3.04 | 22.00 | -3.32 | 6.23 | 0.570 |
| VC_1784 | 79.65 | 0.54 | 1386.42 | 1423.00 | -0.04 | 0.03 | 0.304 |
| VC_1783 | 30.75 | 0.48 | 254.09 | 470.00 | -0.89 | 0.12 | 0.229 |
| VC_1782 | 32.72 | 0.51 | 325.01 | 605.00 | -0.90 | 0.09 | 0.214 |
| VC_1781 | 15.71 | 0.50 | 323.36 | 162.00 | 0.99 | 0.03 | 0.189 |
| VC_1780 | 1.00 | 0.00 | 1.00 | 8.00 | -3.00 | 0.00 | 1.000 |
| VC_1779 | 26.52 | 0.70 | 289.53 | 197.00 | 0.55 | 0.04 | 0.556 |
| VC_1778 | 20.99 | 0.10 | 247.41 | 262.00 | -0.09 | 0.07 | 0.587 |
| VC_1777 | 42.94 | 0.24 | 516.19 | 736.00 | -0.51 | 0.06 | 0.594 |

| | | | | | | | |
|---|---|---|---|---|---|---|---|
| VC_1776 | 27.49 | 0.63 | 612.50 | 586.00 | 0.06 | 0.04 | 0.496 |
| VC_1775 | 28.71 | 0.48 | 122.23 | 263.00 | -1.12 | 0.19 | 0.071 |
| VC_1774 | 37.22 | 0.77 | 424.77 | 337.00 | 0.33 | 0.04 | 0.066 |
| VC_1773 | 28.58 | 0.59 | 224.43 | 174.00 | 0.36 | 0.06 | 0.211 |
| VC_1772 | 29.35 | 0.67 | 403.21 | 483.00 | -0.26 | 0.06 | 0.655 |
| VC_1771 | 152.42 | 1.17 | 2200.01 | 2251.00 | -0.03 | 0.02 | 0.730 |
| VC_1770 | 70.99 | 0.76 | 924.16 | 1143.00 | -0.31 | 0.04 | 0.822 |
| VC_1769 | 74.30 | 0.77 | 1298.96 | 1084.00 | 0.26 | 0.02 | 0.833 |
| VC_1768 | 56.20 | 0.84 | 599.12 | 742.00 | -0.31 | 0.05 | 0.888 |
| VC_1767 | 42.66 | 0.64 | 534.05 | 797.00 | -0.58 | 0.07 | 0.431 |
| VC_1766 | 59.29 | 0.69 | 1039.33 | 941.00 | 0.14 | 0.03 | 0.732 |
| VC_1765 | 106.10 | 0.87 | 1809.12 | 1172.00 | 0.63 | 0.01 | 0.245 |
| VC_1764 | 64.49 | 0.72 | 1080.42 | 862.00 | 0.32 | 0.02 | 0.267 |
| VC_1763 | 25.92 | 0.27 | 579.73 | 224.00 | 1.37 | 0.02 | 0.016 |
| VC_1762 | 63.11 | 0.90 | 856.82 | 680.00 | 0.33 | 0.03 | 0.285 |
| VC_1761 | 22.69 | 0.49 | 309.32 | 208.00 | 0.57 | 0.04 | 0.463 |
| VC_1760 | 109.48 | 1.46 | 1429.81 | 967.00 | 0.56 | 0.02 | 0.002 |
| VC_1759 | 12.60 | 0.49 | 125.43 | 130.00 | -0.06 | 0.09 | 0.398 |
| VC_1758 | 45.02 | 1.29 | 456.80 | 409.00 | 0.16 | 0.04 | 0.512 |
| VC_1757 | 63.38 | 0.75 | 1036.82 | 977.00 | 0.08 | 0.03 | 0.201 |
| VC_1756 | 13.55 | 0.50 | 194.53 | 167.00 | 0.21 | 0.06 | 0.609 |
| VC_1755 | 11.59 | 0.49 | 72.98 | 114.00 | -0.66 | 0.16 | 0.583 |
| VC_1754 | 50.25 | 0.74 | 776.36 | 669.00 | 0.21 | 0.03 | 0.332 |
| VC_1753 | 32.22 | 0.69 | 467.12 | 377.00 | 0.31 | 0.04 | 0.464 |
| VC_1752 | 4.00 | 0.00 | 29.56 | 10.00 | 1.50 | 0.08 | 0.329 |
| VC_1751 | 23.92 | 0.27 | 392.12 | 294.00 | 0.41 | 0.04 | 0.223 |
| VC_1750 | 35.46 | 0.58 | 567.94 | 672.00 | -0.25 | 0.05 | 0.622 |
| VC_1749 | 35.14 | 0.77 | 626.96 | 463.00 | 0.44 | 0.03 | 0.402 |
| VC_1748 | 20.95 | 0.22 | 222.38 | 427.00 | -0.95 | 0.14 | 0.811 |
| VC_1747 | 5.65 | 0.48 | 50.62 | 110.00 | -1.15 | 0.36 | 0.624 |
| VC_1746 | 12.97 | 0.17 | 102.28 | 233.00 | -1.20 | 0.22 | 0.155 |
| VC_1745 | 27.61 | 0.49 | 513.43 | 556.00 | -0.12 | 0.05 | 0.542 |
| VC_1744 | 8.95 | 0.22 | 44.07 | 93.00 | -1.12 | 0.37 | 0.304 |
| VC_1743 | 36.58 | 0.54 | 254.25 | 285.00 | -0.17 | 0.08 | 0.887 |
| VC_1742 | 1.00 | 0.00 | 1.00 | 1.00 | 0.00 | 0.00 | 1.000 |
| VC_1741 | 20.00 | 0.00 | 323.08 | 321.00 | 0.00 | 0.06 | 0.444 |
| VC_1740 | 55.98 | 0.14 | 1585.64 | 1841.00 | -0.22 | 0.03 | 0.165 |
| VC_1739 | 0.00 | 0.00 | 1.00 | 1.00 | #VALEUR! | NaN | NaN |
| VC_1738 | 0.00 | 0.00 | 1.00 | 1.00 | #VALEUR! | NaN | NaN |
| VC_1737 | 1.00 | 0.00 | 11.51 | 1.00 | 3.38 | 0.03 | 1.000 |

| | | | | | | | |
|---|---|---|---|---|---|---|---|
| VC_1736 | 21.15 | 0.76 | 409.17 | 242.00 | 0.75 | 0.03 | 0.072 |
| VC_1735 | 5.00 | 0.00 | 13.77 | 276.00 | -4.44 | 6.92 | 0.105 |
| VC_1734 | 2.00 | 0.00 | 1.00 | 12.00 | -3.58 | 0.00 | 0.333 |
| VC_1733 | 3.00 | 0.00 | 47.94 | 29.00 | 0.69 | 0.10 | 0.902 |
| VC_1732 | 28.21 | 0.67 | 712.51 | 428.00 | 0.73 | 0.02 | 0.160 |
| VC_1731 | 8.00 | 0.00 | 143.61 | 79.00 | 0.85 | 0.05 | 0.615 |
| VC_1730 | 6.00 | 0.00 | 42.40 | 94.00 | -1.18 | 0.34 | 0.223 |
| VC_1729 | 1.00 | 0.00 | 11.97 | 1.00 | 3.43 | 0.03 | 1.000 |
| VC_1728 | 2.00 | 0.00 | 51.27 | 20.00 | 1.33 | 0.06 | 0.400 |
| VC_1727 | 32.64 | 0.52 | 701.82 | 298.00 | 1.23 | 0.02 | 0.164 |
| VC_1726 | 28.49 | 0.59 | 326.90 | 205.00 | 0.67 | 0.04 | 0.111 |
| VC_1725 | 19.99 | 0.10 | 423.37 | 587.00 | -0.47 | 0.06 | 0.750 |
| VC_1724 | 0.00 | 0.00 | 1.00 | 1.00 | #VALEUR! | NaN | NaN |
| VC_1723 | 14.52 | 0.54 | 167.79 | 133.00 | 0.33 | 0.07 | 0.659 |
| VC_1722 | 18.81 | 0.42 | 263.41 | 152.00 | 0.79 | 0.03 | 0.463 |
| VC_1721 | 33.48 | 0.59 | 380.85 | 196.00 | 0.96 | 0.02 | 0.010 |
| VC_1720 | 7.93 | 0.26 | 62.39 | 81.00 | -0.40 | 0.18 | 0.556 |
| VC_1719 | 19.99 | 0.10 | 545.13 | 477.00 | 0.19 | 0.04 | 0.940 |
| VC_1718 | 26.52 | 0.59 | 303.12 | 403.00 | -0.42 | 0.08 | 0.202 |
| VC_1717 | 10.91 | 0.29 | 89.49 | 125.00 | -0.50 | 0.15 | 0.801 |
| VC_1716 | 23.83 | 0.40 | 115.21 | 112.00 | 0.03 | 0.10 | 0.208 |
| VC_1715 | 10.20 | 0.70 | 178.25 | 57.00 | 1.64 | 0.02 | 0.052 |
| VC_1714 | 40.53 | 0.87 | 308.72 | 321.00 | -0.06 | 0.07 | 0.489 |
| VC_1713 | 11.99 | 0.10 | 87.20 | 156.00 | -0.85 | 0.19 | 0.467 |
| VC_1712 | 9.99 | 0.82 | 55.37 | 58.00 | -0.09 | 0.14 | 0.872 |
| VC_1711 | 43.53 | 1.00 | 623.35 | 865.00 | -0.48 | 0.06 | 0.163 |
| VC_1710 | 64.28 | 0.82 | 947.55 | 737.00 | 0.36 | 0.03 | 0.461 |
| VC_1709 | 75.18 | 1.28 | 1101.42 | 1110.00 | -0.01 | 0.03 | 0.778 |
| VC_1708 | 16.95 | 0.22 | 227.24 | 275.00 | -0.28 | 0.09 | 0.791 |
| VC_1707 | 8.00 | 0.00 | 194.84 | 68.00 | 1.51 | 0.03 | 0.198 |
| VC_1706 | 11.91 | 0.29 | 88.59 | 118.00 | -0.43 | 0.15 | 0.576 |
| VC_1705 | 1.00 | 0.00 | 15.76 | 1.00 | 3.85 | 0.03 | 1.000 |
| VC_1704 | 52.11 | 0.97 | 1111.60 | 816.00 | 0.44 | 0.02 | 0.020 |
| VC_1703 | 11.00 | 0.00 | 150.37 | 69.00 | 1.11 | 0.04 | 0.025 |
| VC_1702 | 3.00 | 0.00 | 114.24 | 15.00 | 2.91 | 0.01 | 0.101 |
| VC_1701 | 5.49 | 0.58 | 65.47 | 20.00 | 1.69 | 0.04 | 0.063 |
| VC_1700 | 20.65 | 0.52 | 364.10 | 348.00 | 0.06 | 0.05 | 0.525 |
| VC_1699 | 8.96 | 0.20 | 116.17 | 45.00 | 1.35 | 0.04 | 0.444 |
| VC_1698 | 14.24 | 0.75 | 70.04 | 184.00 | -1.41 | 0.30 | 0.078 |
| VC_1697 | 14.92 | 0.27 | 265.31 | 390.00 | -0.56 | 0.09 | 0.353 |

| | | | | | | | |
|---|---|---|---|---|---|---|---|
| VC_1696 | 9.66 | 0.48 | 303.58 | 107.00 | 1.50 | 0.02 | 0.487 |
| VC_1695 | 27.72 | 0.93 | 143.72 | 282.00 | -0.98 | 0.16 | 0.091 |
| VC_1694 | 4.00 | 0.00 | 18.98 | 22.00 | -0.29 | 0.28 | 0.810 |
| VC_1693 | 19.20 | 0.68 | 158.32 | 225.00 | -0.52 | 0.12 | 0.685 |
| VC_1692 | 43.81 | 1.30 | 977.51 | 265.00 | 1.88 | 0.01 | 0.000 |
| VC_1691 | 2.00 | 0.00 | 33.17 | 1.00 | 5.01 | 0.01 | 1.000 |
| VC_1690 | 18.00 | 0.00 | 563.34 | 371.00 | 0.60 | 0.03 | 0.803 |
| VC_1689 | 14.50 | 0.59 | 218.82 | 34.00 | 2.68 | 0.01 | 0.019 |
| VC_1688 | 27.35 | 1.08 | 186.59 | 310.00 | -0.74 | 0.13 | 0.686 |
| VC_1687 | 19.89 | 0.91 | 204.69 | 257.00 | -0.34 | 0.10 | 0.587 |
| VC_1686 | 10.86 | 0.40 | 95.16 | 58.00 | 0.70 | 0.06 | 0.144 |
| VC_1685 | 15.61 | 0.51 | 152.22 | 305.00 | -1.01 | 0.16 | 0.819 |
| VC_1684 | 8.00 | 0.00 | 136.62 | 231.00 | -0.77 | 0.14 | 0.180 |
| VC_1683 | 9.33 | 0.70 | 49.16 | 50.00 | -0.05 | 0.15 | 0.789 |
| VC_1682 | 18.49 | 0.58 | 129.80 | 108.00 | 0.26 | 0.07 | 0.651 |
| VC_1681 | 10.82 | 0.41 | 128.82 | 9.00 | 3.83 | 0.01 | 0.025 |
| VC_1680 | 27.90 | 0.30 | 221.84 | 387.00 | -0.81 | 0.12 | 0.500 |
| VC_1679 | 19.00 | 0.00 | 463.96 | 379.00 | 0.29 | 0.03 | 0.080 |
| VC_1678 | 6.97 | 0.17 | 213.45 | 37.00 | 2.52 | 0.01 | 0.073 |
| VC_1677 | 4.00 | 0.00 | 19.47 | 21.00 | -0.19 | 0.31 | 0.788 |
| VC_1676 | 14.61 | 0.55 | 252.10 | 89.00 | 1.50 | 0.02 | 0.181 |
| VC_1675 | 24.06 | 0.76 | 524.88 | 358.00 | 0.55 | 0.03 | 0.860 |
| VC_1674 | 16.65 | 0.48 | 301.57 | 395.00 | -0.40 | 0.09 | 0.046 |
| VC_1673 | 67.45 | 1.07 | 808.10 | 1019.00 | -0.34 | 0.04 | 0.846 |
| VC_1672 | 16.94 | 0.24 | 375.62 | 290.00 | 0.37 | 0.04 | 0.725 |
| VC_1671 | 20.45 | 0.59 | 434.79 | 312.00 | 0.48 | 0.03 | 0.291 |
| VC_1670 | 27.55 | 0.58 | 438.34 | 306.00 | 0.52 | 0.03 | 0.738 |
| VC_1669 | 32.64 | 0.50 | 446.40 | 719.00 | -0.69 | 0.08 | 0.447 |
| VC_1668 | 15.86 | 0.35 | 168.96 | 170.00 | -0.02 | 0.08 | 0.794 |
| VC_1667 | 13.77 | 0.49 | 118.26 | 149.00 | -0.35 | 0.13 | 0.764 |
| VC_1666 | 10.56 | 0.64 | 206.30 | 157.00 | 0.39 | 0.05 | 0.115 |
| VC_1665 | 44.29 | 0.74 | 979.74 | 826.00 | 0.25 | 0.02 | 0.736 |
| VC_1664 | 20.94 | 0.24 | 197.45 | 348.00 | -0.82 | 0.11 | 0.331 |
| VC_1663 | 3.90 | 0.30 | 21.95 | 39.00 | -0.90 | 0.44 | 0.573 |
| VC_1662 | 4.88 | 0.33 | 220.51 | 17.00 | 3.69 | 0.00 | 0.042 |
| VC_1661 | 5.90 | 0.30 | 37.10 | 32.00 | 0.16 | 0.19 | 0.728 |
| VC_1660 | 40.53 | 0.63 | 852.17 | 601.00 | 0.50 | 0.02 | 0.327 |
| VC_1659 | 13.70 | 0.58 | 89.25 | 229.00 | -1.38 | 0.29 | 0.446 |
| VC_1658 | 24.00 | 0.00 | 137.88 | 347.00 | -1.34 | 0.20 | 0.002 |
| VC_1657 | 5.00 | 0.00 | 107.13 | 20.00 | 2.40 | 0.02 | 0.183 |

| | | | | | | | |
|---|---|---|---|---|---|---|---|
| VC_1656 | 20.90 | 0.30 | 301.30 | 425.00 | -0.50 | 0.08 | 0.226 |
| VC_1655 | 34.66 | 0.52 | 413.75 | 752.00 | -0.87 | 0.09 | 0.011 |
| VC_1654 | 2.00 | 0.00 | 32.18 | 20.00 | 0.63 | 0.13 | 1.000 |
| VC_1653 | 80.58 | 0.62 | 929.45 | 1119.00 | -0.27 | 0.04 | 0.295 |
| VC_1652 | 49.60 | 0.62 | 838.85 | 808.00 | 0.05 | 0.03 | 0.286 |
| VC_1651 | 43.37 | 0.75 | 423.95 | 559.00 | -0.40 | 0.06 | 0.911 |
| VC_1650 | 86.37 | 0.69 | 1333.23 | 1312.00 | 0.02 | 0.03 | 0.643 |
| VC_1649 | 53.68 | 0.53 | 671.32 | 646.00 | 0.05 | 0.04 | 0.868 |
| VC_1648 | 1.00 | 0.00 | 2.22 | 23.00 | -3.87 | 7.42 | 1.000 |
| VC_1647 | 50.64 | 0.54 | 653.13 | 583.00 | 0.16 | 0.04 | 0.914 |
| VC_1646 | 7.00 | 0.00 | 155.42 | 256.00 | -0.73 | 0.14 | 0.439 |
| VC_1645 | 15.47 | 0.66 | 142.37 | 99.00 | 0.51 | 0.06 | 0.093 |
| VC_1644 | 34.62 | 0.58 | 532.19 | 563.00 | -0.08 | 0.05 | 0.306 |
| VC_1643 | 26.83 | 0.38 | 573.99 | 321.00 | 0.84 | 0.03 | 0.153 |
| VC_1642 | 8.96 | 0.20 | 186.68 | 101.00 | 0.88 | 0.04 | 0.219 |
| VC_1641 | 16.58 | 0.61 | 104.95 | 46.00 | 1.18 | 0.05 | 0.230 |
| VC_1640 | 0.00 | 0.00 | 1.00 | 1.00 | #VALEUR! | NaN | NaN |
| VC_1639 | 25.51 | 0.70 | 65.33 | 228.00 | -1.82 | 0.43 | 0.090 |
| VC_1638 | 12.95 | 0.22 | 224.26 | 44.00 | 2.34 | 0.01 | 0.130 |
| VC_1637 | 6.71 | 0.48 | 242.96 | 89.00 | 1.44 | 0.02 | 0.594 |
| VC_1636 | 35.85 | 0.39 | 549.70 | 601.00 | -0.13 | 0.05 | 0.873 |
| VC_1635 | 13.92 | 0.27 | 397.98 | 175.00 | 1.18 | 0.02 | 0.007 |
| VC_1634 | 32.94 | 0.24 | 709.43 | 391.00 | 0.86 | 0.02 | 0.566 |
| VC_1633 | 11.97 | 0.17 | 93.31 | 146.00 | -0.66 | 0.17 | 0.729 |
| VC_1632 | 29.80 | 0.40 | 424.59 | 436.00 | -0.04 | 0.05 | 0.368 |
| VC_1631 | 24.58 | 0.65 | 286.72 | 308.00 | -0.11 | 0.07 | 0.371 |
| VC_1630 | 17.71 | 0.54 | 182.14 | 242.00 | -0.42 | 0.10 | 0.753 |
| VC_1629 | 60.58 | 0.59 | 645.48 | 732.00 | -0.18 | 0.05 | 0.032 |
| VC_1628 | 25.52 | 0.66 | 337.17 | 262.00 | 0.36 | 0.04 | 0.547 |
| VC_1627 | 33.52 | 0.61 | 386.73 | 315.00 | 0.29 | 0.04 | 0.171 |
| VC_1625 | 51.13 | 0.84 | 776.64 | 721.00 | 0.10 | 0.04 | 0.198 |
| VC_1624 | 29.59 | 0.67 | 284.71 | 305.00 | -0.11 | 0.07 | 0.822 |
| VC_1623 | 27.78 | 0.46 | 233.87 | 309.00 | -0.41 | 0.09 | 0.861 |
| VC_1622 | 18.77 | 0.45 | 157.05 | 348.00 | -1.16 | 0.17 | 0.241 |
| VC_1621 | 33.68 | 0.51 | 401.90 | 665.00 | -0.73 | 0.08 | 0.774 |
| VC_1619.1.1 | 73.90 | 0.30 | 2244.34 | 2135.00 | 0.07 | 0.02 | 0.850 |
| VC_1619.1 | 151.22 | 1.28 | 2221.35 | 2041.00 | 0.12 | 0.02 | 0.100 |
| VC_1619 | 6.81 | 0.39 | 49.72 | 83.00 | -0.76 | 0.22 | 0.815 |
| VC_1618 | 27.70 | 0.52 | 407.06 | 386.00 | 0.07 | 0.05 | 0.920 |
| VC_1617 | 14.60 | 0.59 | 119.69 | 67.00 | 0.83 | 0.05 | 0.499 |

| | | | | | | | |
|---|---|---|---|---|---|---|---|
| VC_1616 | 9.88 | 0.33 | 133.42 | 162.00 | -0.29 | 0.11 | 0.815 |
| VC_1615 | 15.61 | 0.63 | 256.69 | 282.00 | -0.14 | 0.07 | 0.672 |
| VC_1614 | 24.99 | 0.10 | 250.87 | 336.00 | -0.43 | 0.09 | 0.279 |
| VC_1613 | 4.00 | 0.00 | 1.00 | 32.00 | -5.00 | 0.00 | 0.029 |
| VC_1612 | 13.00 | 0.00 | 163.86 | 128.00 | 0.35 | 0.06 | 0.883 |
| VC_1611 | 22.75 | 0.46 | 553.06 | 437.00 | 0.34 | 0.04 | 0.291 |
| VC_1610 | 12.00 | 0.00 | 149.15 | 264.00 | -0.84 | 0.17 | 0.126 |
| VC_1609 | 22.50 | 0.70 | 196.43 | 153.00 | 0.35 | 0.06 | 0.189 |
| VC_1608 | 35.35 | 0.78 | 391.20 | 485.00 | -0.31 | 0.07 | 0.563 |
| VC_1607 | 12.67 | 0.51 | 228.36 | 217.00 | 0.07 | 0.06 | 0.542 |
| VC_1606 | 33.62 | 0.55 | 291.15 | 307.00 | -0.08 | 0.06 | 0.880 |
| VC_1605 | 31.94 | 0.24 | 324.61 | 613.00 | -0.92 | 0.10 | 0.378 |
| VC_1604 | 13.00 | 0.00 | 196.19 | 152.00 | 0.36 | 0.05 | 0.884 |
| VC_1603 | 23.00 | 0.00 | 319.66 | 421.00 | -0.40 | 0.08 | 0.003 |
| VC_1602 | 25.89 | 0.31 | 801.82 | 640.00 | 0.32 | 0.03 | 0.848 |
| VC_1601 | 22.00 | 0.00 | 267.93 | 286.00 | -0.10 | 0.06 | 0.242 |
| VC_1600 | 16.77 | 0.45 | 143.50 | 225.00 | -0.66 | 0.13 | 0.896 |
| VC_1599 | 31.64 | 0.54 | 315.73 | 349.00 | -0.15 | 0.07 | 0.406 |
| VC_1598 | 25.95 | 0.22 | 356.26 | 455.00 | -0.36 | 0.07 | 0.871 |
| VC_1597 | 30.75 | 0.46 | 667.72 | 433.00 | 0.62 | 0.02 | 0.781 |
| VC_1596 | 18.65 | 0.56 | 173.51 | 238.00 | -0.46 | 0.10 | 0.408 |
| VC_1595 | 17.56 | 0.61 | 194.05 | 462.00 | -1.26 | 0.16 | 0.105 |
| VC_1594 | 16.87 | 0.34 | 274.39 | 207.00 | 0.40 | 0.04 | 0.810 |
| VC_1593 | 36.36 | 0.77 | 299.67 | 457.00 | -0.61 | 0.09 | 0.853 |
| VC_1592 | 19.94 | 0.24 | 249.19 | 294.00 | -0.24 | 0.07 | 0.878 |
| VC_1591 | 19.55 | 0.63 | 251.41 | 78.00 | 1.68 | 0.02 | 0.035 |
| VC_1590 | 32.36 | 0.75 | 373.39 | 300.00 | 0.31 | 0.05 | 0.657 |
| VC_1589 | 12.98 | 0.14 | 150.11 | 62.00 | 1.27 | 0.03 | 0.096 |
| VC_1588 | 21.85 | 0.36 | 336.05 | 307.00 | 0.13 | 0.06 | 0.149 |
| VC_1587 | 11.85 | 0.36 | 48.08 | 82.00 | -0.81 | 0.30 | 0.529 |
| VC_1586 | 3.00 | 0.00 | 5.94 | 4.00 | 0.29 | 0.41 | 1.000 |
| VC_1585 | 36.47 | 0.70 | 719.75 | 513.00 | 0.49 | 0.03 | 0.190 |
| VC_1584 | 14.90 | 0.33 | 213.78 | 260.00 | -0.29 | 0.09 | 0.801 |
| VC_1583 | 10.74 | 0.48 | 723.41 | 259.00 | 1.48 | 0.01 | 0.148 |
| VC_1582 | 32.83 | 0.40 | 453.18 | 421.00 | 0.10 | 0.04 | 0.723 |
| VC_1581 | 39.74 | 0.44 | 642.65 | 440.00 | 0.54 | 0.03 | 0.070 |
| VC_1580 | 19.85 | 0.39 | 276.06 | 205.00 | 0.42 | 0.04 | 0.793 |
| VC_1579 | 40.77 | 0.42 | 537.75 | 712.00 | -0.41 | 0.05 | 0.701 |
| VC_1578 | 3.90 | 0.30 | 4.48 | 26.00 | -2.89 | 4.43 | 0.187 |
| VC_1577 | 13.87 | 0.34 | 91.88 | 133.00 | -0.55 | 0.16 | 0.710 |

| | | | | | | | |
|---|---|---|---|---|---|---|---|
| VC_1576 | 7.84 | 0.37 | 72.46 | 163.00 | -1.19 | 0.31 | 0.266 |
| VC_1575 | 14.86 | 0.35 | 166.49 | 377.00 | -1.19 | 0.18 | 0.402 |
| VC_1574 | 7.00 | 0.00 | 49.16 | 39.00 | 0.30 | 0.12 | 0.416 |
| VC_1573 | 20.63 | 0.51 | 228.44 | 284.00 | -0.32 | 0.08 | 0.675 |
| VC_1572 | 10.84 | 0.37 | 99.41 | 233.00 | -1.24 | 0.24 | 0.099 |
| VC_1571 | 35.73 | 0.47 | 596.12 | 497.00 | 0.26 | 0.04 | 0.770 |
| VC_1570 | 25.59 | 0.60 | 510.92 | 364.00 | 0.49 | 0.03 | 0.466 |
| VC_1569 | 3.96 | 0.20 | 58.11 | 15.00 | 1.93 | 0.04 | 0.105 |
| VC_1568 | 15.81 | 0.42 | 216.74 | 206.00 | 0.07 | 0.07 | 0.148 |
| VC_1567 | 26.88 | 0.36 | 507.91 | 234.00 | 1.12 | 0.02 | 0.036 |
| VC_1566 | 21.82 | 0.41 | 396.60 | 283.00 | 0.48 | 0.04 | 0.698 |
| VC_1565 | 22.83 | 0.38 | 188.71 | 317.00 | -0.76 | 0.12 | 0.089 |
| VC_1564 | 2.00 | 0.00 | 1.00 | 9.00 | -3.17 | 0.00 | 0.333 |
| VC_1563 | 23.61 | 0.57 | 415.84 | 262.00 | 0.66 | 0.03 | 0.156 |
| VC_1562 | 34.54 | 0.64 | 505.80 | 551.00 | -0.13 | 0.05 | 0.556 |
| VC_1561 | 22.54 | 0.67 | 228.82 | 176.00 | 0.37 | 0.05 | 0.762 |
| VC_1560 | 39.49 | 0.70 | 777.09 | 579.00 | 0.42 | 0.02 | 0.247 |
| VC_1559 | 3.00 | 0.00 | 1.00 | 37.00 | -5.21 | 0.00 | 0.100 |
| VC_1558 | 37.54 | 0.67 | 456.14 | 665.00 | -0.55 | 0.07 | 0.144 |
| VC_1557 | 16.48 | 0.70 | 41.75 | 141.00 | -1.79 | 0.56 | 0.418 |
| VC_1556 | 7.89 | 0.31 | 49.89 | 142.00 | -1.53 | 0.38 | 0.558 |
| VC_1555 | 8.91 | 0.32 | 84.81 | 151.00 | -0.85 | 0.21 | 0.424 |
| VC_1554 | 13.92 | 0.27 | 168.55 | 116.00 | 0.53 | 0.06 | 0.345 |
| VC_1553 | 15.81 | 0.42 | 143.74 | 172.00 | -0.27 | 0.11 | 0.601 |
| VC_1552 | 20.50 | 0.66 | 225.00 | 222.00 | 0.01 | 0.07 | 0.580 |
| VC_1551 | 19.92 | 0.27 | 384.24 | 530.00 | -0.47 | 0.07 | 0.324 |
| VC_1550 | 12.00 | 0.00 | 104.46 | 262.00 | -1.34 | 0.27 | 0.250 |
| VC_1549 | 36.74 | 0.46 | 410.65 | 368.00 | 0.15 | 0.05 | 0.819 |
| VC_1548 | 11.94 | 0.24 | 177.04 | 101.00 | 0.80 | 0.05 | 0.118 |
| VC_1547 | 17.80 | 0.43 | 313.04 | 335.00 | -0.10 | 0.06 | 0.738 |
| VC_1546 | 7.00 | 0.00 | 66.18 | 100.00 | -0.61 | 0.17 | 0.171 |
| VC_1545 | 3.00 | 0.00 | 75.20 | 64.00 | 0.21 | 0.11 | 0.997 |
| VC_1544 | 14.87 | 0.34 | 152.49 | 329.00 | -1.12 | 0.17 | 0.915 |
| VC_1543 | 25.99 | 0.10 | 255.56 | 764.00 | -1.59 | 0.18 | 0.038 |
| VC_1542 | 24.83 | 0.38 | 216.11 | 292.00 | -0.44 | 0.09 | 0.667 |
| VC_1541 | 24.45 | 0.73 | 429.83 | 300.00 | 0.52 | 0.03 | 0.023 |
| VC_1540 | 24.81 | 0.39 | 431.02 | 602.00 | -0.49 | 0.07 | 0.360 |
| VC_1539 | 26.82 | 0.39 | 256.33 | 351.00 | -0.46 | 0.08 | 0.897 |
| VC_1538 | 12.88 | 0.33 | 171.90 | 168.00 | 0.02 | 0.08 | 0.537 |
| VC_1537 | 13.00 | 0.00 | 79.95 | 198.00 | -1.33 | 0.27 | 0.132 |

| | | | | | | | |
|---|---|---|---|---|---|---|---|
| VC_1536 | 9.00 | 0.00 | 53.55 | 192.00 | -1.87 | 0.49 | 0.058 |
| VC_1535 | 29.83 | 0.40 | 444.77 | 269.00 | 0.72 | 0.03 | 0.086 |
| VC_1534 | 13.50 | 0.66 | 62.40 | 173.00 | -1.50 | 0.40 | 0.534 |
| VC_1533 | 20.39 | 0.71 | 404.18 | 227.00 | 0.83 | 0.03 | 0.714 |
| VC_1532 | 24.84 | 0.37 | 331.12 | 429.00 | -0.38 | 0.08 | 0.403 |
| VC_1531 | 5.94 | 0.24 | 25.02 | 25.00 | -0.06 | 0.21 | 0.738 |
| VC_1530 | 5.00 | 0.00 | 56.38 | 73.00 | -0.40 | 0.17 | 0.579 |
| VC_1529 | 10.99 | 0.10 | 270.43 | 148.00 | 0.86 | 0.03 | 0.270 |
| VC_1528 | 8.99 | 0.10 | 72.17 | 63.00 | 0.18 | 0.10 | 0.679 |
| VC_1527 | 31.51 | 0.63 | 253.95 | 575.00 | -1.18 | 0.14 | 0.091 |
| VC_1526 | 9.98 | 0.14 | 72.99 | 110.00 | -0.61 | 0.19 | 0.767 |
| VC_1525 | 13.99 | 0.10 | 273.29 | 103.00 | 1.40 | 0.02 | 0.283 |
| VC_1524 | 12.91 | 0.29 | 111.62 | 265.00 | -1.26 | 0.27 | 0.096 |
| VC_1523 | 21.87 | 0.37 | 158.84 | 343.00 | -1.12 | 0.19 | 0.848 |
| VC_1522 | 21.56 | 0.61 | 118.83 | 242.00 | -1.04 | 0.19 | 0.109 |
| VC_1521 | 38.86 | 0.38 | 640.44 | 836.00 | -0.39 | 0.04 | 0.383 |
| VC_1520 | 33.79 | 0.43 | 688.73 | 868.00 | -0.34 | 0.05 | 0.720 |
| VC_1519 | 17.92 | 0.27 | 226.62 | 303.00 | -0.43 | 0.10 | 0.835 |
| VC_1518 | 9.87 | 0.34 | 379.19 | 101.00 | 1.91 | 0.01 | 0.163 |
| VC_1517 | 6.00 | 0.00 | 42.32 | 105.00 | -1.33 | 0.32 | 0.190 |
| VC_1516 | 34.69 | 0.49 | 335.46 | 387.00 | -0.21 | 0.06 | 0.636 |
| VC_1515 | 8.00 | 0.00 | 98.09 | 118.00 | -0.28 | 0.13 | 0.316 |
| VC_1514 | 4.87 | 0.34 | 42.75 | 34.00 | 0.30 | 0.11 | 0.622 |
| VC_1513 | 43.66 | 0.54 | 674.51 | 679.00 | -0.01 | 0.04 | 0.890 |
| VC_1512 | 8.73 | 0.47 | 102.12 | 231.00 | -1.19 | 0.22 | 0.414 |
| VC_1511 | 28.67 | 0.55 | 337.41 | 491.00 | -0.55 | 0.07 | 0.120 |
| VC_1510 | 13.00 | 0.00 | 121.53 | 185.00 | -0.62 | 0.15 | 0.627 |
| VC_1509 | 12.92 | 0.27 | 418.06 | 216.00 | 0.95 | 0.03 | 0.228 |
| VC_1508 | 2.87 | 0.34 | 10.84 | 40.00 | -2.03 | 1.35 | 1.000 |
| VC_1507 | 28.31 | 0.85 | 412.92 | 189.00 | 1.12 | 0.02 | 0.019 |
| VC_1506 | 9.88 | 0.33 | 267.16 | 86.00 | 1.63 | 0.02 | 0.144 |
| VC_1505 | 23.87 | 0.34 | 513.25 | 309.00 | 0.73 | 0.03 | 0.220 |
| VC_1504 | 13.59 | 0.62 | 226.78 | 247.00 | -0.13 | 0.08 | 0.678 |
| VC_1503 | 21.92 | 0.27 | 170.79 | 185.00 | -0.12 | 0.08 | 0.372 |
| VC_1502 | 17.27 | 0.75 | 146.44 | 133.00 | 0.13 | 0.09 | 0.125 |
| VC_1501 | 57.83 | 1.06 | 741.15 | 530.00 | 0.48 | 0.03 | 0.197 |
| VC_1500 | 37.67 | 0.55 | 601.68 | 428.00 | 0.49 | 0.03 | 0.917 |
| VC_1499 | 41.37 | 0.69 | 618.74 | 481.00 | 0.36 | 0.03 | 0.556 |
| VC_1498 | 18.85 | 0.36 | 138.96 | 189.00 | -0.45 | 0.11 | 0.638 |
| VC_1497 | 6.00 | 0.00 | 110.66 | 97.00 | 0.18 | 0.09 | 0.711 |

| | | | | | | | |
|---|---|---|---|---|---|---|---|
| VC_1496 | 54.18 | 0.96 | 556.96 | 591.00 | -0.09 | 0.04 | 0.902 |
| VC_1495 | 16.80 | 0.43 | 185.80 | 241.00 | -0.38 | 0.09 | 0.589 |
| VC_1494 | 61.42 | 0.78 | 1105.55 | 663.00 | 0.74 | 0.02 | 0.116 |
| VC_1493 | 3.99 | 0.10 | 6.18 | 21.00 | -2.16 | 3.27 | 0.366 |
| VC_1492 | 91.13 | 0.82 | 1766.90 | 1324.00 | 0.42 | 0.02 | 0.741 |
| VC_1491 | 14.83 | 0.38 | 149.41 | 136.00 | 0.13 | 0.08 | 0.507 |
| VC_1490 | 10.98 | 0.14 | 272.14 | 42.00 | 2.69 | 0.01 | 0.045 |
| VC_1489 | 1.99 | 0.10 | 37.69 | 1.00 | 5.20 | 0.00 | 0.340 |
| VC_1488 | 39.76 | 0.45 | 331.20 | 370.00 | -0.16 | 0.06 | 0.400 |
| VC_1487 | 9.00 | 0.00 | 178.52 | 212.00 | -0.26 | 0.10 | 0.528 |
| VC_1486 | 40.43 | 0.73 | 395.40 | 402.00 | -0.03 | 0.05 | 0.904 |
| VC_1485 | 0.88 | 0.33 | 2.39 | 1.00 | 0.96 | 0.28 | 1.000 |
| VC_1484 | 8.69 | 0.56 | 90.67 | 48.00 | 0.90 | 0.05 | 0.378 |
| VC_1483 | 1.00 | 0.00 | 26.59 | 1.00 | 4.66 | 0.01 | 1.000 |
| VC_1482 | 30.87 | 0.34 | 191.27 | 286.00 | -0.59 | 0.13 | 0.146 |
| VC_1481 | 7.87 | 0.34 | 95.32 | 72.00 | 0.39 | 0.09 | 0.751 |
| VC_1480 | 7.99 | 0.10 | 41.56 | 98.00 | -1.26 | 0.31 | 0.121 |
| VC_1479 | 8.89 | 0.31 | 90.82 | 34.00 | 1.40 | 0.04 | 0.139 |
| VC_1478 | 40.88 | 0.33 | 650.53 | 670.00 | -0.05 | 0.05 | 0.751 |
| VC_1477 | 18.78 | 0.42 | 227.53 | 116.00 | 0.97 | 0.03 | 0.206 |
| VC_1476 | 20.59 | 0.65 | 198.25 | 220.00 | -0.16 | 0.09 | 0.853 |
| VC_1475 | 49.69 | 0.53 | 722.88 | 796.00 | -0.14 | 0.04 | 0.905 |
| VC_1474 | 7.87 | 0.34 | 262.82 | 170.00 | 0.62 | 0.04 | 0.510 |
| VC_1473 | 20.95 | 0.22 | 317.85 | 420.00 | -0.41 | 0.06 | 0.251 |
| VC_1472 | 5.00 | 0.00 | 68.43 | 140.00 | -1.05 | 0.24 | 0.943 |
| VC_1471 | 6.87 | 0.34 | 141.34 | 128.00 | 0.13 | 0.07 | 0.497 |
| VC_1470 | 19.97 | 0.17 | 207.43 | 212.00 | -0.04 | 0.07 | 0.598 |
| VC_1469 | 46.48 | 0.66 | 708.86 | 788.00 | -0.15 | 0.04 | 0.530 |
| VC_1468 | 8.91 | 0.29 | 169.76 | 186.00 | -0.14 | 0.09 | 0.397 |
| VC_1467 | 20.00 | 0.00 | 372.70 | 520.00 | -0.48 | 0.07 | 0.284 |
| VC_1466 | 6.00 | 0.00 | 91.91 | 208.00 | -1.19 | 0.24 | 0.403 |
| VC_1465 | 14.74 | 0.50 | 328.89 | 402.00 | -0.29 | 0.07 | 0.742 |
| VC_1464 | 10.88 | 0.33 | 61.11 | 152.00 | -1.34 | 0.34 | 0.728 |
| VC_1463 | 41.52 | 0.64 | 641.65 | 530.00 | 0.27 | 0.03 | 0.073 |
| VC_1462 | 7.86 | 0.35 | 108.64 | 186.00 | -0.79 | 0.14 | 0.834 |
| VC_1461 | 5.00 | 0.00 | 59.31 | 6.00 | 3.28 | 0.01 | 0.068 |
| VC_1460 | 27.62 | 0.58 | 245.32 | 446.00 | -0.87 | 0.13 | 0.672 |
| VC_1459 | 9.00 | 0.00 | 174.31 | 32.00 | 2.44 | 0.01 | 0.053 |
| VC_1458 | 23.82 | 0.39 | 366.49 | 292.00 | 0.32 | 0.04 | 0.617 |
| VC_1457 | 22.86 | 0.35 | 182.18 | 196.00 | -0.11 | 0.07 | 0.780 |

| | | | | | | | |
|---|---|---|---|---|---|---|---|
| VC_1456 | 5.84 | 0.37 | 23.79 | 12.00 | 0.93 | 0.11 | 0.848 |
| VC_1455 | 12.81 | 0.39 | 57.60 | 156.00 | -1.47 | 0.41 | 0.449 |
| VC_1454 | 37.77 | 0.45 | 687.76 | 666.00 | 0.04 | 0.03 | 0.403 |
| VC_1453 | 9.00 | 0.00 | 69.58 | 152.00 | -1.15 | 0.26 | 0.727 |
| VC_1452 | 10.59 | 0.67 | 73.40 | 84.00 | -0.22 | 0.15 | 0.545 |
| VC_1451 | 287.77 | 1.69 | 4566.87 | 4728.00 | -0.05 | 0.02 | 0.910 |
| VC_1450 | 9.00 | 0.00 | 110.90 | 300.00 | -1.45 | 0.29 | 0.069 |
| VC_1449 | 8.83 | 0.38 | 98.78 | 90.00 | 0.12 | 0.09 | 0.906 |
| VC_1448 | 41.62 | 0.56 | 659.17 | 718.00 | -0.13 | 0.05 | 0.912 |
| VC_1447 | 21.72 | 0.49 | 271.90 | 338.00 | -0.32 | 0.07 | 0.585 |
| VC_1446 | 44.43 | 0.74 | 669.93 | 781.00 | -0.22 | 0.05 | 0.894 |
| VC_1445 | 32.64 | 0.50 | 234.85 | 146.00 | 0.68 | 0.04 | 0.861 |
| VC_1444 | 28.49 | 0.69 | 194.85 | 422.00 | -1.12 | 0.16 | 0.309 |
| VC_1443 | 8.00 | 0.00 | 85.46 | 186.00 | -1.14 | 0.25 | 0.803 |
| VC_1442 | 52.48 | 0.66 | 982.69 | 758.00 | 0.37 | 0.02 | 0.486 |
| VC_1441 | 18.69 | 0.54 | 190.08 | 396.00 | -1.07 | 0.16 | 0.277 |
| VC_1440 | 8.00 | 0.00 | 72.99 | 92.00 | -0.36 | 0.17 | 0.410 |
| VC_1439 | 26.82 | 0.39 | 554.97 | 276.00 | 1.01 | 0.02 | 0.096 |
| VC_1438 | 13.94 | 0.24 | 274.41 | 134.00 | 1.03 | 0.03 | 0.514 |
| VC_1437 | 64.69 | 0.54 | 1213.17 | 1098.00 | 0.14 | 0.03 | 0.446 |
| VC_1436 | 3.00 | 0.00 | 43.27 | 6.00 | 2.82 | 0.02 | 0.120 |
| VC_1435 | 11.86 | 0.35 | 109.79 | 180.00 | -0.73 | 0.17 | 0.543 |
| VC_1434 | 19.69 | 0.49 | 164.36 | 266.00 | -0.70 | 0.11 | 0.481 |
| VC_1433 | 24.58 | 0.64 | 407.73 | 172.00 | 1.24 | 0.02 | 0.010 |
| VC_1432 | 27.69 | 0.53 | 589.33 | 396.00 | 0.57 | 0.03 | 0.107 |
| VC_1431 | 16.69 | 0.49 | 120.73 | 286.00 | -1.26 | 0.22 | 0.023 |
| VC_1430 | 13.00 | 0.00 | 122.37 | 216.00 | -0.83 | 0.15 | 0.248 |
| VC_1429 | 22.59 | 0.60 | 228.71 | 192.00 | 0.25 | 0.06 | 0.005 |
| VC_1428 | 22.56 | 0.64 | 235.26 | 336.00 | -0.52 | 0.10 | 0.697 |
| VC_1427 | 21.00 | 0.00 | 242.49 | 316.00 | -0.39 | 0.09 | 0.157 |
| VC_1426 | 16.96 | 0.20 | 228.64 | 196.00 | 0.22 | 0.05 | 0.165 |
| VC_1425 | 30.64 | 0.50 | 352.18 | 438.00 | -0.32 | 0.06 | 0.438 |
| VC_1424 | 24.76 | 0.45 | 321.94 | 140.00 | 1.20 | 0.02 | 0.536 |
| VC_1423 | 20.88 | 0.33 | 367.68 | 298.00 | 0.30 | 0.04 | 0.686 |
| VC_1422 | 30.98 | 0.14 | 422.66 | 426.00 | -0.01 | 0.05 | 0.812 |
| VC_1421 | 12.99 | 0.10 | 105.28 | 126.00 | -0.28 | 0.14 | 0.618 |
| VC_1420 | 28.61 | 0.60 | 353.46 | 396.00 | -0.17 | 0.06 | 0.420 |
| VC_1419 | 3.94 | 0.24 | 15.57 | 16.00 | -0.14 | 0.31 | 0.973 |
| VC_1418 | 60.26 | 0.81 | 630.98 | 478.00 | 0.40 | 0.03 | 0.363 |
| VC_1417 | 32.70 | 0.50 | 451.77 | 414.00 | 0.12 | 0.04 | 0.882 |

| | | | | | | | |
|---|---|---|---|---|---|---|---|
| VC_1416 | 68.41 | 0.74 | 1269.45 | 1250.00 | 0.02 | 0.03 | 0.616 |
| VC_1415 | 13.00 | 0.00 | 222.48 | 178.00 | 0.32 | 0.05 | 0.553 |
| VC_1414 | 23.89 | 0.31 | 430.59 | 542.00 | -0.34 | 0.07 | 0.610 |
| VC_1413 | 48.58 | 0.65 | 1009.75 | 650.00 | 0.63 | 0.02 | 0.110 |
| VC_1412 | 5.00 | 0.00 | 33.94 | 68.00 | -1.05 | 0.41 | 0.666 |
| VC_1411 | 25.78 | 0.44 | 203.86 | 248.00 | -0.29 | 0.08 | 0.362 |
| VC_1410 | 40.92 | 0.27 | 820.26 | 586.00 | 0.48 | 0.03 | 0.119 |
| VC_1409 | 29.63 | 0.56 | 413.99 | 428.00 | -0.05 | 0.05 | 0.350 |
| VC_1408 | 8.99 | 0.10 | 23.03 | 100.00 | -2.18 | 0.93 | 0.310 |
| VC_1407 | 15.84 | 0.37 | 310.92 | 100.00 | 1.63 | 0.02 | 0.017 |
| VC_1406 | 29.72 | 0.45 | 545.35 | 333.00 | 0.71 | 0.03 | 0.591 |
| VC_1405 | 44.15 | 0.85 | 486.18 | 443.00 | 0.13 | 0.04 | 0.870 |
| VC_1404 | 2.89 | 0.31 | 5.14 | 8.00 | -1.06 | 1.49 | 0.852 |
| VC_1403 | 43.05 | 0.96 | 616.69 | 537.00 | 0.20 | 0.04 | 0.432 |
| VC_1402 | 46.43 | 0.87 | 588.61 | 774.00 | -0.40 | 0.06 | 0.772 |
| VC_1401 | 29.88 | 0.33 | 591.66 | 567.00 | 0.06 | 0.04 | 0.521 |
| VC_1400 | 14.63 | 0.58 | 194.93 | 199.00 | -0.04 | 0.08 | 0.864 |
| VC_1399 | 19.83 | 0.38 | 178.23 | 117.00 | 0.60 | 0.05 | 0.392 |
| VC_1398 | 7.76 | 0.45 | 210.57 | 73.00 | 1.52 | 0.02 | 0.252 |
| VC_1397 | 45.14 | 0.82 | 448.28 | 582.00 | -0.38 | 0.06 | 0.617 |
| VC_1396 | 9.00 | 0.00 | 88.68 | 153.00 | -0.80 | 0.18 | 0.087 |
| VC_1395 | 8.89 | 0.31 | 164.87 | 123.00 | 0.41 | 0.06 | 0.733 |
| VC_1394 | 34.27 | 0.87 | 306.31 | 488.00 | -0.68 | 0.09 | 0.260 |
| VC_1393 | 4.90 | 0.30 | 104.26 | 11.00 | 3.23 | 0.01 | 0.523 |
| VC_1392 | 28.74 | 0.50 | 438.22 | 528.00 | -0.27 | 0.05 | 0.552 |
| VC_1391 | 35.47 | 0.72 | 614.50 | 502.00 | 0.29 | 0.04 | 0.067 |
| VC_1390 | 19.95 | 0.22 | 298.48 | 243.00 | 0.29 | 0.05 | 0.464 |
| VC_1389 | 2.93 | 0.26 | 3.28 | 50.00 | -4.44 | 13.98 | 0.395 |
| VC_1388 | 12.86 | 0.35 | 95.18 | 55.00 | 0.77 | 0.06 | 0.578 |
| VC_1387 | 2.00 | 0.00 | 41.86 | 47.00 | -0.20 | 0.18 | 0.977 |
| VC_1386 | 18.00 | 0.00 | 266.72 | 178.00 | 0.58 | 0.04 | 0.253 |
| VC_1385 | 2.00 | 0.00 | 16.53 | 36.00 | -1.23 | 0.68 | 0.560 |
| VC_1384 | 10.70 | 0.50 | 90.77 | 166.00 | -0.89 | 0.21 | 0.865 |
| VC_1383 | 3.00 | 0.00 | 1.00 | 80.00 | -6.32 | 0.00 | 0.100 |
| VC_1382 | 57.34 | 0.74 | 766.60 | 770.00 | -0.01 | 0.04 | 0.528 |
| VC_1381 | 4.00 | 0.00 | 45.74 | 19.00 | 1.23 | 0.07 | 0.961 |
| VC_1380 | 4.74 | 0.52 | 37.28 | 37.00 | -0.04 | 0.19 | 0.453 |
| VC_1379 | 13.85 | 0.36 | 110.09 | 339.00 | -1.64 | 0.32 | 0.148 |
| VC_1378 | 7.00 | 0.00 | 86.95 | 207.00 | -1.27 | 0.25 | 0.187 |
| VC_1377 | 10.00 | 0.00 | 57.00 | 202.00 | -1.86 | 0.53 | 0.012 |

| | | | | | | | |
|---|---|---|---|---|---|---|---|
| VC_1376 | 43.73 | 0.47 | 613.51 | 614.00 | 0.00 | 0.05 | 0.482 |
| VC_1375 | 10.74 | 0.48 | 18.36 | 117.00 | -2.74 | 1.45 | 0.110 |
| VC_1374 | 33.79 | 0.46 | 617.30 | 428.00 | 0.53 | 0.03 | 0.524 |
| VC_1373 | 46.67 | 0.49 | 633.78 | 613.00 | 0.05 | 0.04 | 0.231 |
| VC_1372 | 28.86 | 0.35 | 381.61 | 418.00 | -0.14 | 0.06 | 0.204 |
| VC_1371 | 16.66 | 0.52 | 68.93 | 221.00 | -1.71 | 0.45 | 0.075 |
| VC_1370 | 37.85 | 0.36 | 705.20 | 615.00 | 0.20 | 0.03 | 0.219 |
| VC_1369 | 21.92 | 0.27 | 261.27 | 301.00 | -0.21 | 0.08 | 0.527 |
| VC_1368 | 4.00 | 0.00 | 132.65 | 109.00 | 0.27 | 0.07 | 0.800 |
| VC_1367 | 63.08 | 0.93 | 744.68 | 858.00 | -0.21 | 0.04 | 0.764 |
| VC_1366 | 11.93 | 0.29 | 113.50 | 118.00 | -0.07 | 0.10 | 0.810 |
| VC_1365 | 12.80 | 0.45 | 126.79 | 94.00 | 0.42 | 0.07 | 0.712 |
| VC_1364 | 22.93 | 0.26 | 254.38 | 257.00 | -0.02 | 0.07 | 0.678 |
| VC_1363 | 19.87 | 0.34 | 241.07 | 273.00 | -0.18 | 0.07 | 0.840 |
| VC_1362 | 22.90 | 0.30 | 384.54 | 582.00 | -0.60 | 0.08 | 0.715 |
| VC_1361 | 38.62 | 0.53 | 642.62 | 523.00 | 0.29 | 0.03 | 0.478 |
| VC_1360 | 36.68 | 0.55 | 488.15 | 611.00 | -0.33 | 0.05 | 0.684 |
| VC_1359 | 13.79 | 0.46 | 187.71 | 164.00 | 0.19 | 0.06 | 0.602 |
| VC_1358 | 10.73 | 0.47 | 94.93 | 15.00 | 2.65 | 0.01 | 0.004 |
| VC_1357 | 1.00 | 0.00 | 11.20 | 1.00 | 3.31 | 0.05 | 1.000 |
| VC_1356 | 1.00 | 0.00 | 1.00 | 17.00 | -4.09 | 0.00 | 1.000 |
| VC_1355 | 7.96 | 0.20 | 175.75 | 34.00 | 2.36 | 0.02 | 0.009 |
| VC_1354 | 31.48 | 0.70 | 244.93 | 426.00 | -0.81 | 0.13 | 0.815 |
| VC_1353 | 73.67 | 0.49 | 1034.78 | 1266.00 | -0.29 | 0.03 | 0.416 |
| VC_1352 | 0.00 | 0.00 | 1.00 | 1.00 | #VALEUR! | NaN | NaN |
| VC_1351 | 7.95 | 0.22 | 94.28 | 48.00 | 0.96 | 0.06 | 0.958 |
| VC_1350 | 9.81 | 0.39 | 96.60 | 74.00 | 0.37 | 0.08 | 0.911 |
| VC_1349 | 99.72 | 0.98 | 1773.10 | 1591.00 | 0.16 | 0.02 | 0.199 |
| VC_1348 | 36.50 | 0.61 | 337.01 | 432.00 | -0.36 | 0.07 | 0.664 |
| VC_1347 | 14.84 | 0.37 | 146.58 | 199.00 | -0.45 | 0.11 | 0.544 |
| VC_1346 | 14.98 | 0.14 | 370.76 | 304.00 | 0.28 | 0.04 | 0.291 |
| VC_1345 | 24.00 | 0.00 | 717.31 | 459.00 | 0.64 | 0.02 | 0.301 |
| VC_1344 | 24.77 | 0.45 | 615.21 | 452.00 | 0.44 | 0.03 | 0.592 |
| VC_1343 | 21.65 | 0.58 | 346.42 | 223.00 | 0.63 | 0.03 | 0.434 |
| VC_1342 | 29.48 | 0.66 | 84.72 | 551.00 | -2.72 | 0.86 | 0.000 |
| VC_1341 | 9.88 | 0.33 | 66.93 | 116.00 | -0.81 | 0.21 | 0.347 |
| VC_1340 | 28.70 | 0.46 | 620.65 | 789.00 | -0.35 | 0.05 | 0.231 |
| VC_1339 | 15.83 | 0.40 | 52.00 | 500.00 | -3.30 | 1.45 | 0.008 |
| VC_1338 | 38.86 | 0.38 | 896.81 | 854.00 | 0.07 | 0.04 | 0.840 |
| VC_1337 | 13.95 | 0.22 | 231.17 | 193.00 | 0.25 | 0.06 | 0.912 |

| | | | | | | | |
|---|---|---|---|---|---|---|---|
| VC_1336 | 12.69 | 0.56 | 160.22 | 302.00 | -0.92 | 0.15 | 0.326 |
| VC_1335 | 11.00 | 0.00 | 103.92 | 187.00 | -0.86 | 0.19 | 0.530 |
| VC_1334 | 13.84 | 0.37 | 130.22 | 92.00 | 0.49 | 0.07 | 0.549 |
| VC_1333 | 10.98 | 0.14 | 94.41 | 103.00 | -0.14 | 0.12 | 0.919 |
| VC_1332 | 34.71 | 0.54 | 870.19 | 482.00 | 0.85 | 0.02 | 0.501 |
| VC_1331 | 2.92 | 0.27 | 9.99 | 9.00 | 0.00 | 0.40 | 0.922 |
| VC_1330 | 14.64 | 0.59 | 111.02 | 79.00 | 0.48 | 0.07 | 0.878 |
| VC_1329 | 16.60 | 0.64 | 166.56 | 43.00 | 1.94 | 0.02 | 0.402 |
| VC_1328 | 25.86 | 0.40 | 401.09 | 424.00 | -0.08 | 0.05 | 0.746 |
| VC_1327 | 20.92 | 0.27 | 401.94 | 334.00 | 0.26 | 0.04 | 0.836 |
| VC_1326 | 3.95 | 0.22 | 63.51 | 44.00 | 0.51 | 0.08 | 0.916 |
| VC_1325 | 21.00 | 0.00 | 338.92 | 342.00 | -0.02 | 0.06 | 0.156 |
| VC_1324 | 4.00 | 0.00 | 18.57 | 114.00 | -2.69 | 1.61 | 0.453 |
| VC_1323 | 10.00 | 0.00 | 105.64 | 102.00 | 0.04 | 0.09 | 0.110 |
| VC_1322 | 5.00 | 0.00 | 39.61 | 43.00 | -0.16 | 0.19 | 0.819 |
| VC_1321 | 88.93 | 0.96 | 1355.41 | 1211.00 | 0.16 | 0.03 | 0.878 |
| VC_1320 | 18.93 | 0.26 | 355.53 | 347.00 | 0.03 | 0.05 | 0.832 |
| VC_1319 | 39.59 | 0.57 | 444.61 | 394.00 | 0.17 | 0.05 | 0.842 |
| VC_1318 | 23.86 | 0.35 | 295.91 | 227.00 | 0.38 | 0.05 | 0.791 |
| VC_1317 | 18.44 | 0.66 | 104.02 | 71.00 | 0.53 | 0.08 | 0.436 |
| VC_1316 | 9.44 | 0.61 | 132.56 | 132.00 | 0.00 | 0.08 | 0.898 |
| VC_1315 | 23.97 | 0.17 | 355.55 | 412.00 | -0.22 | 0.06 | 0.863 |
| VC_1314 | 38.90 | 0.97 | 462.81 | 525.00 | -0.19 | 0.07 | 0.324 |
| VC_1313 | 31.29 | 0.96 | 398.29 | 274.00 | 0.54 | 0.04 | 0.376 |
| VC_1312 | 23.00 | 0.00 | 228.10 | 320.00 | -0.50 | 0.11 | 0.395 |
| VC_1311 | 21.89 | 0.85 | 202.64 | 402.00 | -0.99 | 0.14 | 0.280 |
| VC_1310 | 4.67 | 0.47 | 82.04 | 18.00 | 2.17 | 0.03 | 0.304 |
| VC_1309 | 19.00 | 0.00 | 321.13 | 469.00 | -0.55 | 0.08 | 0.889 |
| VC_1308 | 24.77 | 0.90 | 360.95 | 474.00 | -0.40 | 0.07 | 0.380 |
| VC_1307 | 20.45 | 0.56 | 277.52 | 260.00 | 0.09 | 0.06 | 0.347 |
| VC_1306 | 27.62 | 0.49 | 284.24 | 513.00 | -0.86 | 0.10 | 0.214 |
| VC_1305 | 6.00 | 0.00 | 57.00 | 91.00 | -0.70 | 0.21 | 0.240 |
| VC_1304 | 28.53 | 0.56 | 492.22 | 325.00 | 0.60 | 0.03 | 0.183 |
| VC_1303 | 30.14 | 0.78 | 330.09 | 370.00 | -0.17 | 0.07 | 0.899 |
| VC_1302 | 12.00 | 0.00 | 69.87 | 70.00 | -0.02 | 0.11 | 0.449 |
| VC_1301 | 37.58 | 1.42 | 516.11 | 478.00 | 0.11 | 0.04 | 0.491 |
| VC_1300 | 31.85 | 0.91 | 342.88 | 354.00 | -0.05 | 0.05 | 0.853 |
| VC_1299 | 12.62 | 0.51 | 80.70 | 117.00 | -0.56 | 0.17 | 0.663 |
| VC_1298 | 43.86 | 0.38 | 757.49 | 659.00 | 0.20 | 0.03 | 0.422 |
| VC_1297 | 2.00 | 0.00 | 14.32 | 6.00 | 1.12 | 0.16 | 1.000 |

| | | | | | | | |
|---|---|---|---|---|---|---|---|
| VC_1296 | 26.44 | 0.61 | 319.48 | 328.00 | -0.04 | 0.06 | 0.679 |
| VC_1295 | 35.95 | 0.85 | 647.51 | 390.00 | 0.73 | 0.02 | 0.163 |
| VC_1294 | 7.00 | 0.00 | 109.85 | 77.00 | 0.50 | 0.07 | 0.611 |
| VC_1293 | 23.27 | 0.71 | 276.74 | 304.00 | -0.14 | 0.08 | 0.739 |
| VC_1292 | 2.00 | 0.00 | 59.68 | 22.00 | 1.41 | 0.05 | 0.817 |
| VC_1291 | 48.24 | 0.70 | 1027.48 | 406.00 | 1.34 | 0.01 | 0.006 |
| VC_1290 | 13.99 | 0.10 | 297.88 | 42.00 | 2.82 | 0.01 | 0.005 |
| VC_1289 | 40.00 | 0.00 | 684.15 | 683.00 | 0.00 | 0.04 | 0.871 |
| VC_1288 | 37.97 | 0.17 | 633.42 | 378.00 | 0.74 | 0.02 | 0.871 |
| VC_1287 | 56.88 | 0.84 | 526.45 | 692.00 | -0.40 | 0.06 | 0.645 |
| VC_1286 | 22.23 | 0.76 | 222.03 | 166.00 | 0.41 | 0.05 | 0.665 |
| VC_1285 | 16.27 | 0.63 | 180.50 | 319.00 | -0.83 | 0.14 | 0.232 |
| VC_1284 | 30.55 | 0.54 | 616.72 | 278.00 | 1.15 | 0.02 | 0.903 |
| VC_1283 | 7.54 | 0.50 | 18.17 | 58.00 | -1.75 | 0.82 | 0.243 |
| VC_1282 | 37.44 | 0.67 | 454.50 | 640.00 | -0.50 | 0.07 | 0.334 |
| VC_1281 | 7.24 | 0.65 | 39.73 | 94.00 | -1.27 | 0.37 | 0.155 |
| VC_1280 | 42.50 | 1.05 | 442.12 | 529.00 | -0.26 | 0.06 | 0.621 |
| VC_1279 | 32.68 | 0.51 | 248.87 | 345.00 | -0.48 | 0.09 | 0.748 |
| VC_1278 | 17.00 | 0.00 | 329.99 | 263.00 | 0.32 | 0.05 | 0.390 |
| VC_1277 | 18.31 | 0.75 | 464.99 | 225.00 | 1.04 | 0.02 | 0.053 |
| VC_1276 | 25.84 | 0.77 | 306.37 | 206.00 | 0.57 | 0.04 | 0.429 |
| VC_1275 | 26.18 | 0.76 | 197.49 | 487.00 | -1.31 | 0.17 | 0.335 |
| VC_1274 | 10.14 | 0.75 | 70.33 | 131.00 | -0.92 | 0.23 | 0.886 |
| VC_1273 | 34.65 | 0.48 | 614.42 | 601.00 | 0.03 | 0.04 | 0.803 |
| VC_1272 | 6.00 | 0.00 | 58.41 | 97.00 | -0.75 | 0.20 | 0.577 |
| VC_1271 | 20.78 | 0.42 | 280.03 | 461.00 | -0.72 | 0.10 | 0.579 |
| VC_1270 | 10.10 | 0.77 | 219.03 | 84.00 | 1.38 | 0.03 | 0.188 |
| VC_1269 | 12.00 | 0.00 | 234.54 | 167.00 | 0.48 | 0.05 | 0.686 |
| VC_1268 | 38.89 | 0.86 | 566.54 | 538.00 | 0.07 | 0.04 | 0.465 |
| VC_1267 | 24.72 | 0.45 | 370.23 | 500.00 | -0.44 | 0.07 | 0.918 |
| VC_1266 | 20.23 | 0.63 | 364.14 | 198.00 | 0.87 | 0.03 | 0.324 |
| VC_1265 | 27.49 | 0.59 | 410.64 | 420.00 | -0.04 | 0.05 | 0.576 |
| VC_1264 | 33.36 | 0.75 | 843.88 | 477.00 | 0.82 | 0.02 | 0.125 |
| VC_1263 | 8.96 | 0.20 | 65.04 | 193.00 | -1.59 | 0.41 | 0.250 |
| VC_1262 | 2.00 | 0.00 | 73.22 | 1.00 | 6.17 | 0.00 | 0.333 |
| VC_1261 | 34.38 | 0.62 | 308.20 | 386.00 | -0.33 | 0.09 | 0.885 |
| VC_1260 | 26.98 | 0.14 | 355.67 | 426.00 | -0.26 | 0.06 | 0.858 |
| VC_1259 | 20.98 | 0.14 | 386.17 | 463.00 | -0.27 | 0.06 | 0.892 |
| VC_1258 | 2.95 | 0.22 | 7.28 | 35.00 | -2.60 | 4.06 | 1.000 |
| VC_1257 | 4.30 | 0.69 | 54.25 | 1.00 | 5.73 | 0.00 | 0.029 |

| | | | | | | | |
|---|---|---|---|---|---|---|---|
| VC_1256 | 0.00 | 0.00 | 1.00 | 1.00 | #VALEUR! | NaN | NaN |
| VC_1255 | 1.00 | 0.00 | 8.70 | 1.00 | 2.96 | 0.05 | 1.000 |
| VC_1254 | 10.00 | 0.00 | 179.60 | 167.00 | 0.10 | 0.07 | 0.919 |
| VC_1253 | 8.00 | 0.00 | 106.33 | 104.00 | 0.02 | 0.11 | 0.734 |
| VC_1252 | 29.00 | 0.00 | 518.01 | 420.00 | 0.30 | 0.04 | 0.586 |
| VC_1251 | 5.98 | 0.14 | 69.84 | 20.00 | 1.79 | 0.03 | 0.411 |
| VC_1250 | 19.99 | 0.10 | 326.44 | 355.00 | -0.13 | 0.06 | 0.730 |
| VC_1249 | 11.63 | 0.49 | 163.28 | 117.00 | 0.47 | 0.06 | 0.752 |
| VC_1248 | 26.32 | 0.65 | 478.96 | 407.00 | 0.23 | 0.04 | 0.831 |
| VC_1247 | 5.00 | 0.00 | 70.12 | 69.00 | 0.00 | 0.14 | 0.867 |
| VC_1246 | 1.00 | 0.00 | 1.00 | 6.00 | -2.58 | 0.00 | 1.000 |
| VC_1245 | 11.61 | 0.49 | 143.82 | 164.00 | -0.20 | 0.10 | 0.907 |
| VC_1244 | 14.60 | 0.49 | 178.65 | 405.00 | -1.19 | 0.18 | 0.202 |
| VC_1243 | 4.00 | 0.00 | 38.63 | 71.00 | -0.92 | 0.33 | 0.200 |
| VC_1242 | 26.62 | 0.49 | 528.91 | 477.00 | 0.15 | 0.04 | 0.226 |
| VC_1241 | 4.00 | 0.00 | 45.43 | 81.00 | -0.87 | 0.27 | 0.805 |
| VC_1240 | 11.55 | 0.50 | 210.91 | 135.00 | 0.64 | 0.05 | 0.235 |
| VC_1239 | 12.41 | 0.64 | 167.51 | 150.00 | 0.15 | 0.07 | 0.265 |
| VC_1238 | 20.00 | 0.00 | 364.04 | 320.00 | 0.18 | 0.05 | 0.917 |
| VC_1237 | 22.71 | 0.46 | 589.26 | 235.00 | 1.32 | 0.02 | 0.376 |
| VC_1236 | 10.63 | 0.49 | 129.51 | 298.00 | -1.21 | 0.20 | 0.472 |
| VC_1235 | 30.51 | 0.56 | 381.10 | 361.00 | 0.07 | 0.05 | 0.783 |
| VC_1234 | 30.99 | 0.10 | 463.31 | 271.00 | 0.77 | 0.02 | 0.312 |
| VC_1233 | 8.59 | 0.49 | 135.54 | 42.00 | 1.68 | 0.03 | 0.411 |
| VC_1232 | 18.66 | 0.48 | 410.02 | 311.00 | 0.40 | 0.04 | 0.589 |
| VC_1231 | 21.95 | 0.87 | 315.96 | 247.00 | 0.35 | 0.04 | 0.236 |
| VC_1230 | 3.19 | 0.69 | 3.17 | 27.00 | -3.54 | 7.15 | 0.567 |
| VC_1229 | 22.94 | 0.86 | 474.81 | 471.00 | 0.01 | 0.04 | 0.473 |
| VC_1228 | 15.00 | 0.00 | 154.16 | 228.00 | -0.57 | 0.12 | 0.716 |
| VC_1227 | 1.99 | 0.10 | 4.31 | 1.00 | 1.66 | 0.21 | 1.000 |
| VC_1226 | 8.00 | 0.00 | 93.11 | 37.00 | 1.32 | 0.04 | 0.189 |
| VC_1225 | 5.86 | 0.77 | 246.39 | 58.00 | 2.08 | 0.02 | 0.372 |
| VC_1224 | 21.62 | 0.51 | 337.39 | 456.00 | -0.44 | 0.07 | 0.916 |
| VC_1223 | 6.00 | 0.00 | 75.86 | 117.00 | -0.65 | 0.20 | 0.664 |
| VC_1222 | 3.00 | 0.00 | 1.00 | 69.00 | -6.11 | 0.00 | 0.100 |
| VC_1221 | 1.00 | 0.00 | 16.40 | 1.00 | 3.94 | 0.02 | 1.000 |
| VC_1220 | 1.97 | 0.17 | 4.01 | 1.00 | 1.53 | 0.23 | 1.000 |
| VC_1219 | 3.00 | 0.00 | 1.00 | 24.00 | -4.58 | 0.00 | 0.100 |
| VC_1218 | 2.00 | 0.00 | 9.59 | 5.00 | 0.66 | 0.49 | 0.673 |
| VC_1217 | 10.61 | 0.49 | 221.99 | 84.00 | 1.39 | 0.03 | 0.048 |

| | | | | | | | |
|---|---|---|---|---|---|---|---|
| VC_1216 | 23.00 | 0.00 | 290.35 | 407.00 | -0.49 | 0.09 | 0.117 |
| VC_1215 | 0.00 | 0.00 | 1.00 | 1.00 | #VALEUR! | NaN | NaN |
| VC_1214 | 42.37 | 0.75 | 481.16 | 563.00 | -0.23 | 0.06 | 0.636 |
| VC_1213 | 12.66 | 0.54 | 119.91 | 134.00 | -0.17 | 0.11 | 0.721 |
| VC_1212 | 53.19 | 1.00 | 897.80 | 901.00 | -0.01 | 0.03 | 0.382 |
| VC_1211 | 62.20 | 1.25 | 832.68 | 918.00 | -0.14 | 0.04 | 0.327 |
| VC_1210 | 18.33 | 0.64 | 154.04 | 291.00 | -0.93 | 0.16 | 0.204 |
| VC_1209 | 17.70 | 0.46 | 165.02 | 137.00 | 0.26 | 0.06 | 0.832 |
| VC_1208 | 6.99 | 0.10 | 80.36 | 80.00 | -0.01 | 0.11 | 0.617 |
| VC_1207 | 27.87 | 0.34 | 446.72 | 292.00 | 0.61 | 0.03 | 0.509 |
| VC_1206 | 13.98 | 0.14 | 132.18 | 174.00 | -0.41 | 0.12 | 0.859 |
| VC_1205 | 22.19 | 0.71 | 459.24 | 421.00 | 0.12 | 0.05 | 0.659 |
| VC_1204 | 18.68 | 0.49 | 130.80 | 260.00 | -1.00 | 0.16 | 0.326 |
| VC_1203 | 21.20 | 0.72 | 471.58 | 423.00 | 0.15 | 0.05 | 0.409 |
| VC_1202 | 18.83 | 0.38 | 266.61 | 210.00 | 0.34 | 0.05 | 0.884 |
| VC_1201 | 12.86 | 0.35 | 135.78 | 146.00 | -0.11 | 0.09 | 0.547 |
| VC_1200 | 42.28 | 0.70 | 612.11 | 522.00 | 0.23 | 0.03 | 0.865 |
| VC_1199 | 13.91 | 0.29 | 173.16 | 107.00 | 0.68 | 0.05 | 0.422 |
| VC_1198 | 80.31 | 0.72 | 1320.78 | 976.00 | 0.44 | 0.02 | 0.289 |
| VC_1197 | 17.63 | 0.51 | 191.48 | 299.00 | -0.65 | 0.11 | 0.233 |
| VC_1196 | 13.96 | 0.20 | 129.17 | 174.00 | -0.44 | 0.12 | 0.638 |
| VC_1195 | 21.40 | 0.62 | 307.26 | 471.00 | -0.62 | 0.09 | 0.901 |
| VC_1194 | 17.64 | 0.48 | 188.36 | 217.00 | -0.21 | 0.08 | 0.869 |
| VC_1193 | 5.00 | 0.00 | 155.23 | 50.00 | 1.63 | 0.03 | 0.758 |
| VC_1192 | 0.00 | 0.00 | 1.00 | 1.00 | #VALEUR! | NaN | NaN |
| VC_1191 | 7.99 | 0.10 | 513.06 | 308.00 | 0.73 | 0.03 | 0.486 |
| VC_1190 | 22.83 | 0.38 | 417.73 | 224.00 | 0.90 | 0.03 | 0.002 |
| VC_1189 | 5.56 | 0.50 | 36.56 | 76.00 | -1.09 | 0.36 | 0.377 |
| VC_1188 | 37.48 | 0.63 | 607.86 | 268.00 | 1.18 | 0.02 | 0.034 |
| VC_1187 | 8.62 | 0.49 | 37.42 | 105.00 | -1.52 | 0.48 | 0.156 |
| VC_1186 | 16.19 | 0.75 | 194.35 | 225.00 | -0.22 | 0.08 | 0.156 |
| VC_1185 | 25.42 | 1.05 | 387.07 | 358.00 | 0.11 | 0.05 | 0.478 |
| VC_1184 | 30.67 | 0.49 | 668.11 | 921.00 | -0.47 | 0.06 | 0.781 |
| VC_1183 | 16.61 | 0.51 | 277.13 | 382.00 | -0.47 | 0.10 | 0.644 |
| VC_1182 | 21.97 | 0.17 | 329.29 | 141.00 | 1.22 | 0.02 | 0.880 |
| VC_1181 | 6.00 | 0.00 | 33.21 | 38.00 | -0.24 | 0.21 | 0.883 |
| VC_1180 | 5.00 | 0.00 | 82.00 | 46.00 | 0.81 | 0.07 | 0.861 |
| VC_1179 | 16.83 | 0.38 | 147.39 | 457.00 | -1.64 | 0.29 | 0.226 |
| VC_1178 | 12.00 | 0.00 | 191.65 | 115.00 | 0.73 | 0.04 | 0.081 |
| VC_1177 | 21.49 | 0.54 | 251.43 | 476.00 | -0.93 | 0.13 | 0.499 |

| | | | | | | | |
|---|---|---|---|---|---|---|---|
| VC_1176 | 2.65 | 0.48 | 7.38 | 22.00 | -1.78 | 1.60 | 1.000 |
| VC_1175 | 0.87 | 0.34 | 2.34 | 1.00 | 0.88 | 0.30 | 1.000 |
| VC_1174 | 42.00 | 0.00 | 1018.39 | 924.00 | 0.14 | 0.03 | 0.631 |
| VC_1173 | 9.00 | 0.00 | 180.87 | 122.00 | 0.56 | 0.05 | 0.369 |
| VC_1172 | 12.66 | 0.48 | 105.71 | 123.00 | -0.23 | 0.11 | 0.868 |
| VC_1171 | 26.62 | 0.49 | 381.20 | 576.00 | -0.60 | 0.07 | 0.397 |
| VC_1170 | 28.88 | 0.33 | 284.45 | 495.00 | -0.80 | 0.10 | 0.201 |
| VC_1169 | 10.58 | 0.50 | 184.37 | 272.00 | -0.57 | 0.09 | 0.813 |
| VC_1168 | 32.54 | 0.58 | 605.36 | 542.00 | 0.16 | 0.04 | 0.795 |
| VC_1167 | 10.00 | 0.00 | 218.51 | 147.00 | 0.57 | 0.05 | 0.653 |
| VC_1166 | 2.00 | 0.00 | 39.67 | 5.00 | 2.96 | 0.02 | 1.000 |
| VC_1165 | 9.00 | 0.00 | 96.55 | 111.00 | -0.22 | 0.12 | 0.373 |
| VC_1164 | 12.67 | 0.47 | 155.39 | 143.00 | 0.11 | 0.08 | 0.831 |
| VC_1163 | 17.86 | 0.35 | 303.24 | 497.00 | -0.72 | 0.09 | 0.393 |
| VC_1162 | 11.90 | 0.30 | 138.56 | 128.00 | 0.10 | 0.09 | 0.826 |
| VC_1161 | 28.64 | 0.48 | 610.23 | 418.00 | 0.54 | 0.03 | 0.925 |
| VC_1160 | 27.26 | 0.65 | 520.37 | 142.00 | 1.87 | 0.01 | 0.171 |
| VC_1159 | 3.00 | 0.00 | 35.55 | 1.00 | 5.10 | 0.01 | 0.400 |
| VC_1158 | 20.71 | 0.50 | 122.20 | 130.00 | -0.10 | 0.11 | 0.826 |
| VC_1157 | 23.35 | 0.67 | 254.67 | 425.00 | -0.74 | 0.11 | 0.167 |
| VC_1156 | 34.41 | 0.60 | 482.58 | 935.00 | -0.96 | 0.08 | 0.386 |
| VC_1155 | 7.00 | 0.00 | 45.42 | 207.00 | -2.22 | 0.73 | 0.538 |
| VC_1154 | 18.60 | 0.49 | 245.31 | 220.00 | 0.15 | 0.06 | 0.670 |
| VC_1153 | 21.52 | 0.54 | 410.44 | 334.00 | 0.29 | 0.04 | 0.272 |
| VC_1152 | 24.61 | 0.49 | 259.49 | 368.00 | -0.51 | 0.09 | 0.046 |
| VC_1151 | 29.85 | 0.36 | 409.47 | 397.00 | 0.04 | 0.05 | 0.907 |
| VC_1150 | 14.87 | 0.34 | 257.29 | 253.00 | 0.02 | 0.06 | 0.659 |
| VC_1149 | 9.09 | 0.77 | 121.83 | 35.00 | 1.79 | 0.03 | 0.386 |
| VC_1148 | 14.13 | 0.77 | 376.78 | 345.00 | 0.12 | 0.05 | 0.213 |
| VC_1147 | 21.47 | 0.56 | 198.09 | 260.00 | -0.40 | 0.09 | 0.485 |
| VC_1146 | 7.85 | 0.36 | 30.28 | 64.00 | -1.13 | 0.40 | 0.238 |
| VC_1145 | 41.88 | 1.17 | 269.50 | 480.00 | -0.84 | 0.12 | 0.013 |
| VC_1144 | 39.75 | 0.52 | 415.12 | 438.00 | -0.08 | 0.05 | 0.711 |
| VC_1143 | 8.98 | 0.14 | 302.38 | 177.00 | 0.77 | 0.04 | 0.369 |
| VC_1142 | 9.98 | 0.14 | 135.29 | 210.00 | -0.65 | 0.15 | 0.456 |
| VC_1141 | 44.80 | 0.98 | 508.13 | 415.00 | 0.29 | 0.03 | 0.241 |
| VC_1140 | 20.97 | 0.17 | 153.75 | 255.00 | -0.74 | 0.14 | 0.357 |
| VC_1139 | 11.98 | 0.14 | 132.54 | 139.00 | -0.08 | 0.09 | 0.698 |
| VC_1138 | 11.61 | 0.55 | 177.47 | 92.00 | 0.94 | 0.05 | 0.405 |
| VC_1137 | 17.00 | 0.00 | 136.51 | 278.00 | -1.04 | 0.17 | 0.028 |

| | | | | | | | |
|---|---|---|---|---|---|---|---|
| VC_1136 | 13.00 | 0.00 | 243.06 | 173.00 | 0.48 | 0.05 | 0.713 |
| VC_1135 | 15.62 | 0.49 | 99.56 | 216.00 | -1.13 | 0.23 | 0.032 |
| VC_1134 | 24.00 | 0.85 | 323.22 | 169.00 | 0.93 | 0.03 | 0.282 |
| VC_1133 | 24.75 | 0.54 | 623.71 | 442.00 | 0.49 | 0.03 | 0.835 |
| VC_1132 | 17.00 | 0.00 | 223.92 | 256.00 | -0.20 | 0.07 | 0.355 |
| VC_1131.1 | 1.00 | 0.00 | 4.36 | 3.00 | 0.16 | 0.56 | 1.000 |
| VC_1131 | 44.32 | 0.66 | 998.15 | 541.00 | 0.88 | 0.02 | 0.158 |
| VC_1130 | 1.47 | 0.59 | 3.27 | 1.00 | 1.32 | 0.25 | 0.635 |
| VC_1129 | 51.68 | 1.02 | 425.26 | 501.00 | -0.24 | 0.06 | 0.796 |
| VC_1128 | 0.00 | 0.00 | 1.00 | 1.00 | #VALEUR! | NaN | NaN |
| VC_1127 | 7.74 | 0.44 | 50.25 | 47.00 | 0.06 | 0.16 | 0.206 |
| VC_1126 | 1.00 | 0.00 | 11.80 | 28.00 | -1.41 | 1.08 | 1.000 |
| VC_1125 | 13.00 | 0.00 | 110.44 | 135.00 | -0.31 | 0.14 | 0.503 |
| VC_1124 | 14.58 | 0.50 | 180.48 | 154.00 | 0.22 | 0.07 | 0.666 |
| VC_1123 | 11.40 | 0.62 | 298.88 | 404.00 | -0.44 | 0.09 | 0.774 |
| VC_1122 | 38.73 | 0.94 | 597.43 | 600.00 | -0.01 | 0.05 | 0.445 |
| VC_1121 | 25.00 | 0.00 | 289.69 | 127.00 | 1.18 | 0.03 | 0.150 |
| VC_1120 | 30.72 | 0.49 | 352.84 | 591.00 | -0.75 | 0.08 | 0.421 |
| VC_1119 | 18.60 | 0.49 | 530.91 | 497.00 | 0.09 | 0.04 | 0.637 |
| VC_1118 | 15.64 | 0.50 | 149.37 | 243.00 | -0.71 | 0.15 | 0.452 |
| VC_1117 | 23.44 | 0.62 | 612.46 | 388.00 | 0.66 | 0.03 | 0.231 |
| VC_1116 | 6.00 | 0.00 | 118.76 | 48.00 | 1.30 | 0.03 | 0.454 |
| VC_1115 | 27.57 | 0.52 | 375.03 | 604.00 | -0.69 | 0.09 | 0.911 |
| VC_1114 | 18.00 | 0.00 | 139.17 | 122.00 | 0.18 | 0.07 | 0.766 |
| VC_1113 | 13.69 | 0.53 | 223.44 | 374.00 | -0.75 | 0.12 | 0.606 |
| VC_1112 | 14.59 | 0.53 | 294.22 | 155.00 | 0.92 | 0.03 | 0.884 |
| VC_1111 | 29.22 | 0.76 | 688.38 | 408.00 | 0.75 | 0.02 | 0.248 |
| VC_1110 | 0.66 | 0.48 | 1.42 | 1.00 | 0.44 | 0.28 | 1.000 |
| VC_1109 | 1.86 | 0.35 | 2.19 | 25.00 | -3.93 | 7.63 | 1.000 |
| VC_1108 | 36.99 | 0.83 | 281.39 | 358.00 | -0.35 | 0.08 | 0.666 |
| VC_1107 | 2.99 | 0.10 | 6.82 | 3.00 | 0.90 | 0.27 | 0.652 |
| VC_1106 | 11.86 | 0.35 | 71.09 | 120.00 | -0.78 | 0.21 | 0.381 |
| VC_1105 | 23.00 | 0.00 | 382.11 | 660.00 | -0.79 | 0.08 | 0.296 |
| VC_1104 | 51.44 | 1.01 | 852.92 | 676.00 | 0.33 | 0.02 | 0.336 |
| VC_1103 | 10.69 | 0.49 | 97.20 | 140.00 | -0.55 | 0.18 | 0.931 |
| VC_1102 | 23.55 | 0.69 | 434.54 | 413.00 | 0.07 | 0.05 | 0.652 |
| VC_1101 | 25.57 | 0.52 | 323.83 | 494.00 | -0.61 | 0.09 | 0.693 |
| VC_1100 | 10.62 | 0.51 | 68.16 | 138.00 | -1.04 | 0.25 | 0.899 |
| VC_1099 | 13.00 | 0.00 | 77.81 | 131.00 | -0.77 | 0.22 | 0.372 |
| VC_1098 | 21.98 | 0.79 | 296.69 | 343.00 | -0.21 | 0.07 | 0.291 |

| | | | | | | | |
|---|---|---|---|---|---|---|---|
| VC_1097 | 47.40 | 0.70 | 396.81 | 547.00 | -0.47 | 0.08 | 0.086 |
| VC_1096 | 27.63 | 0.49 | 399.30 | 182.00 | 1.13 | 0.02 | 0.048 |
| VC_1095 | 19.97 | 0.17 | 285.79 | 416.00 | -0.55 | 0.09 | 0.849 |
| VC_1094 | 23.29 | 0.74 | 228.46 | 275.00 | -0.27 | 0.07 | 0.681 |
| VC_1093 | 22.63 | 0.49 | 370.72 | 427.00 | -0.21 | 0.06 | 0.064 |
| VC_1092 | 39.23 | 1.06 | 888.06 | 721.00 | 0.30 | 0.03 | 0.496 |
| VC_1091 | 40.32 | 0.71 | 491.50 | 715.00 | -0.54 | 0.07 | 0.183 |
| VC_1090 | 6.89 | 0.31 | 14.40 | 44.00 | -1.70 | 0.86 | 0.176 |
| VC_1089 | 19.86 | 0.35 | 115.70 | 178.00 | -0.63 | 0.15 | 0.678 |
| VC_1088 | 42.53 | 0.59 | 507.62 | 271.00 | 0.90 | 0.02 | 0.029 |
| VC_1087 | 39.47 | 1.24 | 543.99 | 631.00 | -0.22 | 0.05 | 0.275 |
| VC_1086 | 32.18 | 0.70 | 322.90 | 327.00 | -0.02 | 0.06 | 0.754 |
| VC_1085 | 41.39 | 0.65 | 661.28 | 680.00 | -0.04 | 0.04 | 0.403 |
| VC_1084 | 34.46 | 0.61 | 568.28 | 412.00 | 0.46 | 0.03 | 0.232 |
| VC_1083 | 16.00 | 0.00 | 276.70 | 301.00 | -0.13 | 0.07 | 0.730 |
| VC_1082 | 8.63 | 0.49 | 440.31 | 121.00 | 1.86 | 0.02 | 0.035 |
| VC_1081 | 38.60 | 0.55 | 492.27 | 346.00 | 0.51 | 0.03 | 0.297 |
| VC_1080 | 8.00 | 0.00 | 265.31 | 268.00 | -0.02 | 0.07 | 0.789 |
| VC_1079 | 12.72 | 0.45 | 269.49 | 199.00 | 0.43 | 0.05 | 0.816 |
| VC_1078 | 1.00 | 0.00 | 42.25 | 146.00 | -1.82 | 0.55 | 1.000 |
| VC_1077 | 7.00 | 0.00 | 72.46 | 32.00 | 1.16 | 0.06 | 0.506 |
| VC_1076 | 23.87 | 0.86 | 323.13 | 128.00 | 1.33 | 0.02 | 0.024 |
| VC_1075 | 9.00 | 0.00 | 495.26 | 193.00 | 1.36 | 0.02 | 0.910 |
| VC_1074 | 19.23 | 0.69 | 147.34 | 194.00 | -0.41 | 0.11 | 0.360 |
| VC_1073 | 62.30 | 1.02 | 1293.11 | 1591.00 | -0.30 | 0.04 | 0.102 |
| VC_1072 | 1.65 | 0.48 | 7.52 | 1.00 | 2.70 | 0.07 | 0.567 |
| VC_1071 | 28.41 | 0.64 | 415.17 | 415.00 | 0.00 | 0.05 | 0.295 |
| VC_1070 | 8.00 | 0.00 | 143.63 | 127.00 | 0.16 | 0.08 | 0.688 |
| VC_1069 | 17.00 | 0.00 | 487.42 | 258.00 | 0.91 | 0.03 | 0.212 |
| VC_1068 | 4.00 | 0.00 | 31.49 | 20.00 | 0.62 | 0.10 | 0.803 |
| VC_1067 | 76.38 | 0.68 | 1319.00 | 1280.00 | 0.04 | 0.03 | 0.825 |
| VC_1066 | 23.71 | 0.50 | 268.59 | 384.00 | -0.52 | 0.08 | 0.103 |
| VC_1065 | 7.00 | 0.00 | 36.42 | 107.00 | -1.59 | 0.48 | 0.020 |
| VC_1064 | 19.00 | 0.00 | 493.71 | 535.00 | -0.12 | 0.05 | 0.501 |
| VC_1063 | 15.89 | 0.31 | 289.43 | 245.00 | 0.24 | 0.05 | 0.882 |
| VC_1062 | 7.66 | 0.48 | 65.86 | 87.00 | -0.42 | 0.15 | 0.166 |
| VC_1061 | 30.84 | 0.95 | 397.94 | 506.00 | -0.35 | 0.06 | 0.462 |
| VC_1060 | 18.33 | 0.64 | 387.13 | 272.00 | 0.51 | 0.04 | 0.815 |
| VC_1059 | 23.61 | 0.51 | 561.41 | 229.00 | 1.29 | 0.02 | 0.137 |
| VC_1058 | 12.85 | 0.36 | 197.50 | 155.00 | 0.34 | 0.06 | 0.380 |

| | | | | | | | |
|---|---|---|---|---|---|---|---|
| VC_1057 | 6.00 | 0.00 | 34.44 | 31.00 | 0.10 | 0.19 | 0.921 |
| VC_1056 | 10.00 | 0.00 | 102.27 | 151.00 | -0.57 | 0.14 | 0.183 |
| VC_1055 | 2.00 | 0.00 | 11.52 | 2.00 | 2.41 | 0.06 | 0.353 |
| VC_1054 | 2.00 | 0.00 | 60.15 | 20.00 | 1.56 | 0.05 | 0.660 |
| VC_1053 | 10.69 | 0.46 | 160.89 | 184.00 | -0.20 | 0.09 | 0.632 |
| VC_1052 | 11.00 | 0.00 | 75.86 | 141.00 | -0.91 | 0.23 | 0.423 |
| VC_1051 | 5.00 | 0.00 | 25.74 | 30.00 | -0.28 | 0.26 | 0.497 |
| VC_1050 | 28.46 | 0.63 | 464.78 | 600.00 | -0.37 | 0.06 | 0.530 |
| VC_1049 | 21.69 | 0.46 | 548.28 | 508.00 | 0.11 | 0.04 | 0.178 |
| VC_1048 | 10.40 | 0.70 | 91.29 | 80.00 | 0.17 | 0.10 | 0.898 |
| VC_1047 | 46.55 | 0.63 | 619.28 | 572.00 | 0.11 | 0.04 | 0.142 |
| VC_1046 | 40.00 | 0.90 | 676.88 | 591.00 | 0.19 | 0.03 | 0.710 |
| VC_1045 | 19.99 | 0.10 | 310.80 | 215.00 | 0.53 | 0.03 | 0.500 |
| VC_1044 | 12.62 | 0.49 | 158.28 | 236.00 | -0.59 | 0.14 | 0.820 |
| VC_1043 | 47.96 | 0.20 | 828.14 | 824.00 | 0.01 | 0.03 | 0.726 |
| VC_1042 | 46.21 | 0.70 | 637.49 | 576.00 | 0.14 | 0.03 | 0.849 |
| VC_1041 | 7.66 | 0.48 | 120.32 | 48.00 | 1.31 | 0.04 | 0.881 |
| VC_1040 | 10.51 | 0.50 | 222.53 | 132.00 | 0.75 | 0.04 | 0.914 |
| VC_1039 | 36.64 | 0.48 | 283.19 | 574.00 | -1.02 | 0.12 | 0.095 |
| VC_1038 | 17.00 | 0.00 | 102.46 | 602.00 | -2.57 | 0.54 | 0.003 |
| VC_1037 | 28.06 | 0.78 | 140.94 | 514.00 | -1.88 | 0.33 | 0.032 |
| VC_1036 | 8.82 | 0.85 | 39.06 | 50.00 | -0.39 | 0.19 | 0.541 |
| VC_1035 | 17.91 | 0.29 | 290.65 | 246.00 | 0.24 | 0.05 | 0.627 |
| VC_1034 | 14.29 | 0.67 | 483.73 | 210.00 | 1.20 | 0.02 | 0.072 |
| VC_1033 | 43.48 | 0.58 | 582.01 | 448.00 | 0.37 | 0.03 | 0.459 |
| VC_1032 | 1.00 | 0.00 | 5.38 | 21.00 | -2.29 | 2.99 | 1.000 |
| VC_1031 | 10.59 | 0.49 | 142.68 | 192.00 | -0.44 | 0.11 | 0.097 |
| VC_1030 | 0.00 | 0.00 | 1.00 | 1.00 | #VALEUR! | NaN | NaN |
| VC_1029 | 25.70 | 0.46 | 227.22 | 654.00 | -1.53 | 0.17 | 0.049 |
| VC_1028 | 5.00 | 0.00 | 119.06 | 70.00 | 0.75 | 0.06 | 0.905 |
| VC_1027 | 5.63 | 0.49 | 67.31 | 33.00 | 1.01 | 0.06 | 0.781 |
| VC_1026 | 10.62 | 0.49 | 261.48 | 313.00 | -0.26 | 0.07 | 0.202 |
| VC_1025 | 9.47 | 0.59 | 260.16 | 162.00 | 0.68 | 0.04 | 0.911 |
| VC_1024 | 21.19 | 0.72 | 325.50 | 385.00 | -0.25 | 0.06 | 0.449 |
| VC_1023 | 14.00 | 0.00 | 256.20 | 107.00 | 1.25 | 0.03 | 0.466 |
| VC_1022 | 9.00 | 0.00 | 96.44 | 119.00 | -0.32 | 0.13 | 0.548 |
| VC_1021 | 33.34 | 0.67 | 732.37 | 464.00 | 0.66 | 0.03 | 0.904 |
| VC_1020 | 8.00 | 0.00 | 60.24 | 49.00 | 0.27 | 0.12 | 0.445 |
| VC_1018 | 59.85 | 0.39 | 753.97 | 1026.00 | -0.45 | 0.05 | 0.117 |
| VC_1017 | 4.97 | 0.17 | 23.56 | 64.00 | -1.51 | 0.65 | 0.487 |

| | | | | | | | |
|---|---|---|---|---|---|---|---|
| VC_1016 | 2.00 | 0.00 | 19.24 | 17.00 | 0.11 | 0.21 | 0.953 |
| VC_1015 | 8.00 | 0.00 | 153.01 | 165.00 | -0.12 | 0.10 | 0.529 |
| VC_1014 | 3.00 | 0.00 | 11.69 | 49.00 | -2.21 | 1.65 | 0.499 |
| VC_1013 | 2.00 | 0.00 | 15.90 | 1.00 | 3.89 | 0.02 | 0.333 |
| VC_1012 | 1.00 | 0.00 | 102.99 | 1.00 | 6.67 | 0.00 | 1.000 |
| VC_1011 | 18.19 | 0.73 | 194.16 | 179.00 | 0.11 | 0.07 | 0.350 |
| VC_1010 | 15.00 | 0.00 | 295.82 | 300.00 | -0.03 | 0.06 | 0.697 |
| VC_1009 | 15.63 | 0.49 | 361.04 | 396.00 | -0.14 | 0.06 | 0.243 |
| VC_1008 | 24.48 | 0.59 | 442.33 | 206.00 | 1.10 | 0.02 | 0.056 |
| VC_1007 | 2.66 | 0.48 | 11.75 | 4.00 | 1.45 | 0.10 | 0.796 |
| VC_1006 | 10.70 | 0.83 | 83.11 | 229.00 | -1.48 | 0.32 | 0.681 |
| VC_1005 | 24.23 | 0.65 | 402.62 | 477.00 | -0.25 | 0.05 | 0.568 |
| VC_1004 | 37.52 | 1.07 | 523.14 | 588.00 | -0.17 | 0.05 | 0.388 |
| VC_1003 | 10.00 | 0.00 | 172.68 | 123.00 | 0.48 | 0.06 | 0.176 |
| VC_1002 | 6.98 | 0.14 | 105.12 | 27.00 | 1.95 | 0.03 | 0.035 |
| VC_1001 | 0.00 | 0.00 | 1.00 | 1.00 | #VALEUR! | NaN | NaN |
| VC_1000 | 1.00 | 0.00 | 9.53 | 4.00 | 1.10 | 0.19 | 1.000 |
| VC_0999 | 19.00 | 0.00 | 425.84 | 294.00 | 0.53 | 0.04 | 0.363 |
| VC_0998 | 91.45 | 1.34 | 1400.47 | 1177.00 | 0.25 | 0.02 | 0.031 |
| VC_0997 | 1.00 | 0.00 | 6.56 | 9.00 | -0.82 | 1.17 | 1.000 |
| VC_0996 | 1.64 | 0.48 | 5.23 | 1.00 | 1.99 | 0.17 | 0.573 |
| VC_0995 | 43.81 | 0.42 | 1241.99 | 981.00 | 0.34 | 0.02 | 0.663 |
| VC_0994 | 37.75 | 0.93 | 631.03 | 557.00 | 0.18 | 0.04 | 0.494 |
| VC_0993 | 23.32 | 0.87 | 482.33 | 252.00 | 0.93 | 0.02 | 0.747 |
| VC_0992 | 45.64 | 0.50 | 502.64 | 636.00 | -0.34 | 0.06 | 0.297 |
| VC_0991 | 48.91 | 0.83 | 859.93 | 736.00 | 0.22 | 0.03 | 0.505 |
| VC_0990 | 13.60 | 0.49 | 260.90 | 232.00 | 0.16 | 0.06 | 0.917 |
| VC_0989 | 5.00 | 0.00 | 27.49 | 68.00 | -1.36 | 0.50 | 0.073 |
| VC_0988 | 54.22 | 0.69 | 718.76 | 988.00 | -0.46 | 0.05 | 0.686 |
| VC_0987 | 2.61 | 0.49 | 67.95 | 20.00 | 1.75 | 0.03 | 0.817 |
| VC_0986 | 1.00 | 0.00 | 1.00 | 25.00 | -4.64 | 0.00 | 1.000 |
| VC_0985 | 59.32 | 1.04 | 1058.93 | 811.00 | 0.38 | 0.02 | 0.225 |
| VC_0984 | 18.74 | 0.48 | 306.13 | 131.00 | 1.22 | 0.03 | 0.925 |
| VC_0983 | 21.00 | 0.00 | 234.56 | 151.00 | 0.63 | 0.05 | 0.125 |
| VC_0982 | 8.00 | 0.00 | 105.47 | 156.00 | -0.58 | 0.15 | 0.500 |
| VC_0981 | 20.31 | 0.65 | 325.23 | 459.00 | -0.50 | 0.08 | 0.613 |
| VC_0980 | 22.96 | 0.74 | 312.98 | 541.00 | -0.79 | 0.10 | 0.148 |
| VC_0979 | 17.45 | 0.69 | 260.35 | 527.00 | -1.02 | 0.13 | 0.090 |
| VC_0978 | 6.00 | 0.00 | 86.65 | 55.00 | 0.64 | 0.08 | 0.392 |
| VC_0977 | 28.74 | 0.48 | 278.18 | 535.00 | -0.95 | 0.11 | 0.025 |

| | | | | | | | |
|---|---|---|---|---|---|---|---|
| VC_0976 | 29.22 | 0.72 | 380.19 | 286.00 | 0.41 | 0.04 | 0.842 |
| VC_0975 | 13.64 | 0.48 | 124.50 | 178.00 | -0.53 | 0.12 | 0.676 |
| VC_0974 | 11.54 | 0.50 | 110.45 | 131.00 | -0.26 | 0.11 | 0.580 |
| VC_0973 | 29.20 | 0.74 | 285.97 | 317.00 | -0.15 | 0.07 | 0.885 |
| VC_0972 | 47.77 | 0.90 | 917.51 | 1013.00 | -0.14 | 0.04 | 0.365 |
| VC_0971 | 1.00 | 0.00 | 4.89 | 2.00 | 0.88 | 0.37 | 1.000 |
| VC_0970 | 3.82 | 0.39 | 5.94 | 2.00 | 1.30 | 0.23 | 0.719 |
| VC_0969 | 25.57 | 0.52 | 636.10 | 757.00 | -0.25 | 0.05 | 0.362 |
| VC_0968 | 20.00 | 0.00 | 198.98 | 335.00 | -0.76 | 0.13 | 0.286 |
| VC_0967 | 3.00 | 0.00 | 39.11 | 17.00 | 1.17 | 0.07 | 0.941 |
| VC_0966 | 6.86 | 0.35 | 228.48 | 45.00 | 2.34 | 0.01 | 0.632 |
| VC_0965 | 37.00 | 0.00 | 730.45 | 486.00 | 0.59 | 0.03 | 0.667 |
| VC_0964 | 9.99 | 0.10 | 112.84 | 152.00 | -0.44 | 0.11 | 0.804 |
| VC_0963 | 6.17 | 0.75 | 29.99 | 14.00 | 1.04 | 0.11 | 0.288 |
| VC_0962 | 34.00 | 0.00 | 409.03 | 644.00 | -0.66 | 0.08 | 0.397 |
| VC_0961 | 8.82 | 0.39 | 39.06 | 76.00 | -0.99 | 0.31 | 0.889 |
| VC_0960 | 1.00 | 0.00 | 5.20 | 16.00 | -1.88 | 2.17 | 1.000 |
| VC_0959 | 11.99 | 0.10 | 131.69 | 113.00 | 0.21 | 0.08 | 0.840 |
| VC_0958 | 19.29 | 0.74 | 32.86 | 221.00 | -2.80 | 1.37 | 0.014 |
| VC_0957 | 13.36 | 0.69 | 132.44 | 99.00 | 0.41 | 0.07 | 0.585 |
| VC_0956 | 4.18 | 0.72 | 64.45 | 53.00 | 0.26 | 0.11 | 0.924 |
| VC_0955 | 4.88 | 0.33 | 22.30 | 58.00 | -1.45 | 0.65 | 0.578 |
| VC_0954 | 1.00 | 0.00 | 1.00 | 17.00 | -4.09 | 0.00 | 1.000 |
| VC_0953 | 1.00 | 0.00 | 1.00 | 69.00 | -6.11 | 0.00 | 1.000 |
| VC_0952 | 5.00 | 0.00 | 36.17 | 19.00 | 0.88 | 0.10 | 0.824 |
| VC_0951 | 13.00 | 0.00 | 148.71 | 163.00 | -0.14 | 0.09 | 0.872 |
| VC_0950 | 0.82 | 0.39 | 2.08 | 1.00 | 0.83 | 0.29 | 1.000 |
| VC_0949 | 1.00 | 0.00 | 13.73 | 11.00 | 0.20 | 0.27 | 1.000 |
| VC_0948 | 30.06 | 0.75 | 303.47 | 432.00 | -0.51 | 0.08 | 0.783 |
| VC_0947 | 10.15 | 1.13 | 54.96 | 1.00 | 5.76 | 0.00 | 0.000 |
| VC_0946 | 1.00 | 0.00 | 6.05 | 10.00 | -0.99 | 0.99 | 1.000 |
| VC_0945 | 0.00 | 0.00 | 1.00 | 1.00 | #VALEUR! | NaN | NaN |
| VC_0944 | 11.91 | 0.84 | 76.07 | 59.00 | 0.34 | 0.10 | 0.121 |
| VC_0943 | 11.17 | 0.75 | 61.28 | 55.00 | 0.13 | 0.12 | 0.815 |
| VC_0942 | 17.58 | 0.54 | 536.91 | 198.00 | 1.44 | 0.01 | 0.057 |
| VC_0941 | 18.35 | 0.69 | 169.33 | 109.00 | 0.63 | 0.05 | 0.019 |
| VC_0940 | 13.70 | 0.56 | 124.81 | 77.00 | 0.68 | 0.06 | 0.193 |
| VC_0939 | 11.91 | 0.29 | 113.37 | 176.00 | -0.65 | 0.16 | 0.752 |
| VC_0938 | 15.09 | 0.85 | 317.22 | 236.00 | 0.42 | 0.04 | 0.355 |
| VC_0937 | 62.82 | 0.83 | 1067.22 | 881.00 | 0.28 | 0.02 | 0.521 |

| | | | | | | | |
|---|---|---|---|---|---|---|---|
| VC_0936 | 15.56 | 0.50 | 323.54 | 292.00 | 0.14 | 0.05 | 0.253 |
| VC_0935 | 33.83 | 0.97 | 271.53 | 594.00 | -1.13 | 0.13 | 0.240 |
| VC_0934 | 38.62 | 0.53 | 551.36 | 477.00 | 0.21 | 0.03 | 0.315 |
| VC_0933 | 11.00 | 0.00 | 265.15 | 160.00 | 0.72 | 0.04 | 0.260 |
| VC_0932 | 5.00 | 0.00 | 113.08 | 92.00 | 0.28 | 0.09 | 0.968 |
| VC_0931 | 41.78 | 0.92 | 428.48 | 559.00 | -0.39 | 0.06 | 0.395 |
| VC_0930 | 98.18 | 1.11 | 1929.38 | 1640.00 | 0.23 | 0.02 | 0.585 |
| VC_0929 | 21.86 | 0.35 | 207.66 | 193.00 | 0.10 | 0.06 | 0.191 |
| VC_0928 | 24.14 | 0.78 | 277.83 | 317.00 | -0.20 | 0.08 | 0.859 |
| VC_0927 | 14.98 | 0.14 | 189.78 | 110.00 | 0.78 | 0.04 | 0.810 |
| VC_0926 | 33.25 | 0.67 | 468.25 | 642.00 | -0.46 | 0.07 | 0.183 |
| VC_0925 | 29.60 | 0.49 | 648.76 | 453.00 | 0.52 | 0.03 | 0.897 |
| VC_0924 | 40.25 | 1.39 | 570.03 | 604.00 | -0.09 | 0.05 | 0.435 |
| VC_0923 | 22.60 | 0.49 | 274.30 | 296.00 | -0.12 | 0.07 | 0.304 |
| VC_0922 | 51.02 | 1.35 | 510.23 | 411.00 | 0.31 | 0.04 | 0.481 |
| VC_0921 | 53.73 | 0.95 | 611.60 | 921.00 | -0.59 | 0.06 | 0.368 |
| VC_0920 | 34.40 | 0.60 | 357.05 | 580.00 | -0.70 | 0.09 | 0.147 |
| VC_0919 | 27.30 | 0.67 | 528.70 | 545.00 | -0.05 | 0.05 | 0.492 |
| VC_0918 | 30.99 | 0.10 | 525.64 | 599.00 | -0.19 | 0.05 | 0.201 |
| VC_0917 | 26.71 | 1.00 | 481.16 | 470.00 | 0.03 | 0.04 | 0.827 |
| VC_0916 | 7.99 | 0.10 | 180.80 | 27.00 | 2.73 | 0.01 | 0.758 |
| VC_0915 | 8.72 | 0.45 | 196.98 | 114.00 | 0.78 | 0.05 | 0.223 |
| VC_0914 | 76.36 | 0.99 | 1145.88 | 1333.00 | -0.22 | 0.04 | 0.376 |
| VC_0913 | 26.90 | 0.33 | 317.37 | 850.00 | -1.43 | 0.18 | 0.372 |
| VC_0912 | 0.00 | 0.00 | 1.00 | 1.00 | #VALEUR! | NaN | NaN |
| VC_0911 | 33.40 | 0.68 | 182.12 | 201.00 | -0.15 | 0.08 | 0.780 |
| VC_0910 | 34.27 | 0.65 | 973.62 | 774.00 | 0.33 | 0.03 | 0.134 |
| VC_0909 | 26.92 | 0.27 | 391.95 | 269.00 | 0.54 | 0.03 | 0.163 |
| VC_0908 | 7.86 | 0.35 | 25.54 | 142.00 | -2.56 | 1.54 | 0.054 |
| VC_0907 | 20.00 | 0.00 | 266.50 | 232.00 | 0.20 | 0.05 | 0.283 |
| VC_0906 | 12.47 | 0.59 | 158.06 | 297.00 | -0.92 | 0.14 | 0.887 |
| VC_0905 | 20.71 | 0.46 | 680.82 | 560.00 | 0.28 | 0.03 | 0.680 |
| VC_0904 | 17.15 | 0.76 | 596.95 | 329.00 | 0.86 | 0.03 | 0.132 |
| VC_0903 | 12.00 | 0.00 | 206.19 | 342.00 | -0.74 | 0.12 | 0.372 |
| VC_0902 | 18.84 | 0.37 | 280.55 | 266.00 | 0.07 | 0.06 | 0.300 |
| VC_0901 | 80.44 | 1.09 | 1050.58 | 885.00 | 0.25 | 0.02 | 0.509 |
| VC_0900 | 31.99 | 0.82 | 509.04 | 390.00 | 0.38 | 0.03 | 0.461 |
| VC_0899 | 41.86 | 0.98 | 944.34 | 678.00 | 0.48 | 0.02 | 0.500 |
| VC_0898 | 16.46 | 0.61 | 101.36 | 236.00 | -1.24 | 0.29 | 0.041 |
| VC_0897 | 11.85 | 0.36 | 69.86 | 223.00 | -1.69 | 0.38 | 0.329 |

| | | | | | | | |
|---|---|---|---|---|---|---|---|
| VC_0896 | 23.30 | 0.63 | 267.79 | 262.00 | 0.03 | 0.06 | 0.748 |
| VC_0895 | 6.00 | 0.00 | 12.53 | 45.00 | -1.95 | 1.08 | 0.063 |
| VC_0894 | 35.26 | 0.72 | 747.80 | 346.00 | 1.11 | 0.02 | 0.030 |
| VC_0893 | 12.00 | 0.00 | 100.08 | 205.00 | -1.05 | 0.19 | 0.130 |
| VC_0892 | 16.69 | 0.46 | 461.03 | 190.00 | 1.28 | 0.02 | 0.090 |
| VC_0891 | 5.00 | 0.00 | 47.60 | 80.00 | -0.78 | 0.27 | 0.446 |
| VC_0890 | 0.00 | 0.00 | 1.00 | 1.00 | #VALEUR! | NaN | NaN |
| VC_0889 | 2.00 | 0.00 | 2.26 | 37.00 | -4.48 | 11.45 | 0.540 |
| VC_0888 | 10.96 | 0.20 | 166.35 | 263.00 | -0.67 | 0.12 | 0.832 |
| VC_0887 | 4.00 | 0.00 | 135.01 | 22.00 | 2.61 | 0.02 | 0.091 |
| VC_0886 | 18.90 | 0.77 | 118.74 | 329.00 | -1.49 | 0.32 | 0.736 |
| VC_0885 | 4.00 | 0.00 | 18.60 | 43.00 | -1.29 | 0.63 | 0.938 |
| VC_0884 | 19.94 | 0.84 | 346.80 | 373.00 | -0.11 | 0.06 | 0.303 |
| VC_0883 | 2.00 | 0.00 | 30.68 | 14.00 | 1.08 | 0.09 | 0.347 |
| VC_0882 | 8.97 | 0.17 | 104.21 | 162.00 | -0.65 | 0.17 | 0.833 |
| VC_0881 | 9.98 | 0.14 | 114.60 | 89.00 | 0.35 | 0.08 | 0.851 |
| VC_0880 | 49.03 | 0.86 | 940.64 | 961.00 | -0.03 | 0.03 | 0.557 |
| VC_0879 | 1.00 | 0.00 | 12.71 | 22.00 | -0.89 | 0.51 | 1.000 |
| VC_0878 | 8.00 | 0.00 | 79.05 | 159.00 | -1.02 | 0.22 | 0.412 |
| VC_0877 | 3.59 | 0.49 | 36.75 | 21.00 | 0.76 | 0.11 | 0.852 |
| VC_0876 | 17.25 | 0.67 | 263.14 | 351.00 | -0.42 | 0.09 | 0.580 |
| VC_0875 | 1.00 | 0.00 | 1.00 | 11.00 | -3.46 | 0.00 | 1.000 |
| VC_0874 | 8.00 | 0.00 | 70.98 | 91.00 | -0.38 | 0.17 | 0.109 |
| VC_0873 | 14.37 | 0.68 | 274.98 | 174.00 | 0.65 | 0.04 | 0.651 |
| VC_0872 | 9.00 | 0.00 | 224.30 | 111.00 | 1.01 | 0.04 | 0.377 |
| VC_0871 | 3.00 | 0.00 | 51.60 | 35.00 | 0.54 | 0.09 | 0.675 |
| VC_0870 | 22.00 | 0.00 | 220.30 | 184.00 | 0.25 | 0.07 | 0.669 |
| VC_0869 | 68.47 | 0.64 | 1136.76 | 1518.00 | -0.42 | 0.05 | 0.344 |
| VC_0868 | 9.66 | 0.48 | 176.62 | 166.00 | 0.08 | 0.07 | 0.506 |
| VC_0867 | 3.00 | 0.00 | 46.56 | 30.00 | 0.60 | 0.11 | 0.889 |
| VC_0866 | 38.87 | 0.79 | 422.46 | 591.00 | -0.49 | 0.07 | 0.899 |
| VC_0865 | 6.99 | 0.10 | 174.52 | 115.00 | 0.59 | 0.06 | 0.721 |
| VC_0864 | 3.58 | 0.50 | 1.33 | 22.00 | -4.24 | 5.18 | 0.132 |
| VC_0863 | 36.15 | 0.67 | 603.45 | 745.00 | -0.31 | 0.06 | 0.237 |
| VC_0862 | 10.82 | 0.39 | 136.12 | 91.00 | 0.57 | 0.06 | 0.193 |
| VC_0861 | 10.32 | 0.75 | 97.70 | 135.00 | -0.48 | 0.14 | 0.444 |
| VC_0860 | 29.91 | 0.87 | 306.32 | 338.00 | -0.15 | 0.06 | 0.865 |
| VC_0859 | 20.97 | 0.17 | 172.86 | 260.00 | -0.60 | 0.12 | 0.726 |
| VC_0858 | 10.25 | 0.73 | 290.42 | 261.00 | 0.15 | 0.05 | 0.305 |
| VC_0857 | 13.98 | 0.14 | 327.77 | 359.00 | -0.14 | 0.06 | 0.481 |

| | | | | | | | |
|---|---|---|---|---|---|---|---|
| VC_0856 | 1.00 | 0.00 | 1.00 | 10.00 | -3.32 | 0.00 | 1.000 |
| VC_0855 | 3.00 | 0.00 | 47.36 | 10.00 | 2.22 | 0.03 | 0.952 |
| VC_0854 | 1.00 | 0.00 | 14.34 | 1.00 | 3.71 | 0.03 | 1.000 |
| VC_0853 | 0.00 | 0.00 | 1.00 | 1.00 | #VALEUR! | NaN | NaN |
| VC_0852 | 37.90 | 0.88 | 363.56 | 782.00 | -1.11 | 0.11 | 0.235 |
| VC_0851 | 14.00 | 0.00 | 457.53 | 621.00 | -0.44 | 0.07 | 0.730 |
| VC_0850 | 0.00 | 0.00 | 1.00 | 1.00 | #VALEUR! | NaN | NaN |
| VC_0849 | 2.00 | 0.00 | 20.29 | 1.00 | 4.27 | 0.01 | 0.333 |
| VC_0848 | 7.77 | 0.45 | 28.82 | 50.00 | -0.87 | 0.46 | 0.593 |
| VC_0847 | 32.84 | 0.86 | 545.88 | 537.00 | 0.02 | 0.04 | 0.676 |
| VC_0846 | 29.79 | 0.46 | 249.45 | 211.00 | 0.24 | 0.05 | 0.344 |
| VC_0845 | 112.77 | 1.54 | 825.39 | 841.00 | -0.03 | 0.04 | 0.236 |
| VC_0844 | 7.86 | 0.35 | 37.87 | 44.00 | -0.26 | 0.22 | 0.664 |
| VC_0843 | 23.66 | 1.20 | 271.54 | 240.00 | 0.17 | 0.06 | 0.602 |
| VC_0842 | 9.24 | 0.67 | 85.02 | 18.00 | 2.22 | 0.02 | 0.035 |
| VC_0841 | 14.99 | 0.10 | 171.14 | 113.00 | 0.59 | 0.05 | 0.625 |
| VC_0840 | 25.86 | 0.95 | 115.11 | 208.00 | -0.87 | 0.19 | 0.235 |
| VC_0839 | 8.63 | 0.51 | 12.91 | 60.00 | -2.35 | 1.74 | 0.147 |
| VC_0838 | 6.23 | 0.79 | 11.22 | 29.00 | -1.53 | 1.18 | 0.832 |
| VC_0837 | 30.36 | 0.73 | 116.00 | 219.00 | -0.93 | 0.16 | 0.083 |
| VC_0836 | 22.12 | 0.83 | 137.18 | 195.00 | -0.52 | 0.12 | 0.754 |
| VC_0835 | 25.63 | 0.98 | 199.06 | 181.00 | 0.13 | 0.06 | 0.458 |
| VC_0834 | 0.00 | 0.00 | 1.00 | 1.00 | #VALEUR! | NaN | NaN |
| VC_0833 | 3.63 | 0.49 | 14.57 | 34.00 | -1.41 | 1.31 | 0.898 |
| VC_0832 | 5.00 | 0.00 | 10.26 | 19.00 | -1.06 | 0.83 | 0.186 |
| VC_0831 | 47.12 | 1.14 | 214.00 | 455.00 | -1.09 | 0.14 | 0.010 |
| VC_0830 | 10.60 | 0.49 | 82.61 | 129.00 | -0.67 | 0.21 | 0.154 |
| VC_0829 | 30.48 | 1.05 | 264.43 | 361.00 | -0.46 | 0.09 | 0.597 |
| VC_0828 | 24.78 | 0.44 | 392.10 | 261.00 | 0.58 | 0.04 | 0.680 |
| VC_0827 | 11.84 | 0.37 | 105.84 | 70.00 | 0.58 | 0.07 | 0.343 |
| VC_0826 | 17.76 | 0.91 | 110.34 | 171.00 | -0.65 | 0.17 | 0.789 |
| VC_0825 | 68.31 | 1.07 | 816.79 | 925.00 | -0.18 | 0.04 | 0.093 |
| VC_0824 | 15.33 | 0.67 | 205.94 | 194.00 | 0.08 | 0.06 | 0.441 |
| VC_0823 | 21.39 | 1.01 | 93.21 | 107.00 | -0.21 | 0.12 | 0.381 |
| VC_0822 | 44.04 | 1.16 | 286.30 | 242.00 | 0.24 | 0.05 | 0.760 |
| VC_0821 | 3.58 | 0.50 | 21.44 | 4.00 | 2.33 | 0.06 | 0.333 |
| VC_0820 | 45.42 | 1.03 | 350.98 | 360.00 | -0.04 | 0.06 | 0.274 |
| VC_0819 | 21.84 | 0.87 | 140.06 | 288.00 | -1.05 | 0.18 | 0.348 |
| VC_0818 | 27.01 | 0.77 | 320.48 | 359.00 | -0.17 | 0.06 | 0.067 |
| VC_0817 | 34.66 | 0.48 | 528.94 | 767.00 | -0.54 | 0.06 | 0.381 |

| | | | | | | | |
|---|---|---|---|---|---|---|---|
| VC_0816 | 7.87 | 0.34 | 82.44 | 61.00 | 0.42 | 0.08 | 0.664 |
| VC_0815 | 59.06 | 1.16 | 595.61 | 679.00 | -0.19 | 0.04 | 0.691 |
| VC_0814 | 5.00 | 0.00 | 64.88 | 97.00 | -0.60 | 0.18 | 0.963 |
| VC_0813 | 21.29 | 0.62 | 552.56 | 289.00 | 0.93 | 0.02 | 0.074 |
| VC_0812 | 83.06 | 1.02 | 1039.57 | 913.00 | 0.19 | 0.03 | 0.868 |
| VC_0811 | 5.29 | 0.80 | 13.19 | 5.00 | 1.27 | 0.14 | 0.296 |
| VC_0810 | 8.30 | 0.66 | 29.42 | 82.00 | -1.53 | 0.59 | 0.237 |
| VC_0809 | 41.53 | 0.92 | 664.78 | 481.00 | 0.46 | 0.03 | 0.439 |
| VC_0808 | 3.88 | 0.33 | 16.46 | 3.00 | 2.35 | 0.06 | 0.748 |
| VC_0807 | 15.95 | 0.22 | 228.73 | 239.00 | -0.07 | 0.07 | 0.902 |
| VC_0806 | 31.00 | 0.00 | 328.87 | 562.00 | -0.78 | 0.09 | 0.098 |
| VC_0805 | 2.00 | 0.00 | 13.42 | 3.00 | 2.05 | 0.08 | 1.000 |
| VC_0804 | 7.70 | 0.46 | 180.09 | 53.00 | 1.76 | 0.02 | 0.098 |
| VC_0803 | 22.00 | 0.00 | 303.79 | 457.00 | -0.59 | 0.09 | 0.504 |
| VC_0802 | 6.83 | 0.38 | 64.20 | 63.00 | 0.01 | 0.11 | 0.877 |
| VC_0801 | 25.60 | 0.53 | 216.93 | 335.00 | -0.63 | 0.12 | 0.755 |
| VC_0800 | 4.00 | 0.00 | 163.25 | 182.00 | -0.16 | 0.08 | 0.741 |
| VC_0799 | 26.98 | 0.14 | 573.08 | 429.00 | 0.42 | 0.03 | 0.927 |
| VC_0798 | 11.62 | 0.49 | 209.57 | 423.00 | -1.02 | 0.13 | 0.028 |
| VC_0797 | 2.00 | 0.00 | 113.73 | 46.00 | 1.29 | 0.04 | 1.000 |
| VC_0796 | 13.62 | 0.56 | 277.73 | 220.00 | 0.33 | 0.06 | 0.578 |
| VC_0795 | 33.17 | 0.80 | 384.78 | 645.00 | -0.75 | 0.07 | 0.898 |
| VC_0794 | 4.00 | 0.00 | 176.89 | 77.00 | 1.19 | 0.03 | 0.592 |
| VC_0793 | 36.67 | 1.16 | 823.82 | 1056.00 | -0.36 | 0.04 | 0.596 |
| VC_0792 | 31.25 | 0.76 | 673.26 | 703.00 | -0.06 | 0.04 | 0.807 |
| VC_0791 | 25.81 | 0.42 | 216.72 | 274.00 | -0.35 | 0.09 | 0.899 |
| VC_0790 | 19.64 | 0.52 | 154.08 | 101.00 | 0.60 | 0.05 | 0.325 |
| VC_0789 | 8.00 | 0.00 | 203.92 | 111.00 | 0.87 | 0.04 | 0.448 |
| VC_0788 | 7.00 | 0.00 | 87.23 | 100.00 | -0.21 | 0.11 | 0.709 |
| VC_0787 | 27.64 | 0.48 | 435.96 | 588.00 | -0.43 | 0.06 | 0.299 |
| VC_0786 | 29.15 | 0.67 | 510.09 | 270.00 | 0.92 | 0.02 | 0.010 |
| VC_0785 | 1.00 | 0.00 | 1.00 | 9.00 | -3.17 | 0.00 | 1.000 |
| VC_0784 | 40.41 | 0.68 | 709.76 | 531.00 | 0.42 | 0.03 | 0.773 |
| VC_0783 | 55.39 | 0.98 | 954.06 | 1142.00 | -0.26 | 0.04 | 0.634 |
| VC_0782 | 1.64 | 0.48 | 1.48 | 30.00 | -4.61 | 8.13 | 1.000 |
| VC_0781 | 29.88 | 0.84 | 319.76 | 472.00 | -0.57 | 0.09 | 0.637 |
| VC_0780 | 24.16 | 0.79 | 606.58 | 486.00 | 0.32 | 0.03 | 0.283 |
| VC_0779 | 19.00 | 0.00 | 567.69 | 293.00 | 0.95 | 0.02 | 0.767 |
| VC_0778 | 20.90 | 0.30 | 633.38 | 220.00 | 1.52 | 0.01 | 0.037 |
| VC_0777 | 27.33 | 0.64 | 514.48 | 617.00 | -0.26 | 0.05 | 0.581 |

| | | | | | | | |
|---|---|---|---|---|---|---|---|
| VC_0776 | 11.00 | 0.00 | 261.03 | 158.00 | 0.72 | 0.04 | 0.605 |
| VC_0775 | 37.99 | 0.90 | 581.46 | 613.00 | -0.08 | 0.05 | 0.630 |
| VC_0774 | 17.00 | 0.00 | 331.51 | 400.00 | -0.27 | 0.06 | 0.209 |
| VC_0773 | 27.06 | 0.83 | 617.35 | 492.00 | 0.33 | 0.03 | 0.630 |
| VC_0772 | 39.82 | 0.41 | 645.88 | 867.00 | -0.43 | 0.06 | 0.818 |
| VC_0771 | 23.00 | 0.00 | 129.57 | 466.00 | -1.86 | 0.33 | 0.003 |
| VC_0770 | 16.49 | 0.64 | 244.92 | 257.00 | -0.08 | 0.08 | 0.712 |
| VC_0769 | 51.26 | 0.92 | 812.16 | 876.00 | -0.11 | 0.04 | 0.909 |
| VC_0768 | 32.95 | 0.22 | 605.28 | 483.00 | 0.32 | 0.03 | 0.270 |
| VC_0767 | 28.46 | 0.64 | 347.09 | 344.00 | 0.01 | 0.05 | 0.601 |
| VC_0766 | 31.65 | 0.48 | 548.05 | 431.00 | 0.34 | 0.03 | 0.482 |
| VC_0765 | 8.00 | 0.00 | 62.89 | 88.00 | -0.51 | 0.19 | 0.516 |
| VC_0764 | 30.34 | 1.08 | 396.95 | 286.00 | 0.47 | 0.03 | 0.416 |
| VC_0763 | 0.68 | 0.47 | 1.53 | 1.00 | 0.47 | 0.29 | 1.000 |
| VC_0762 | 1.00 | 0.00 | 1.00 | 3.00 | -1.58 | 0.00 | 1.000 |
| VC_0761 | 9.85 | 0.36 | 23.16 | 478.00 | -4.43 | 4.68 | 0.023 |
| VC_0760 | 2.92 | 0.27 | 43.87 | 6.00 | 2.83 | 0.02 | 0.204 |
| VC_0759 | 0.96 | 0.20 | 3.17 | 1.00 | 1.20 | 0.28 | 1.000 |
| VC_0758 | 13.60 | 0.49 | 175.58 | 134.00 | 0.38 | 0.06 | 0.371 |
| VC_0757 | 32.23 | 0.63 | 687.90 | 746.00 | -0.12 | 0.04 | 0.768 |
| VC_0756 | 7.59 | 0.49 | 147.06 | 50.00 | 1.55 | 0.03 | 0.189 |
| VC_0755 | 27.00 | 0.00 | 499.40 | 418.00 | 0.25 | 0.04 | 0.521 |
| VC_0754 | 1.00 | 0.00 | 28.32 | 1.00 | 4.78 | 0.01 | 1.000 |
| VC_0753 | 1.00 | 0.00 | 8.41 | 1.00 | 2.78 | 0.11 | 1.000 |
| VC_0752 | 5.61 | 0.49 | 4.65 | 8.00 | -1.29 | 1.93 | 0.377 |
| VC_0751 | 0.94 | 0.24 | 3.16 | 1.00 | 1.27 | 0.26 | 1.000 |
| VC_0750 | 0.00 | 0.00 | 1.00 | 1.00 | #VALEUR! | NaN | NaN |
| VC_0749 | 1.00 | 0.00 | 6.90 | 3.00 | 1.02 | 0.21 | 1.000 |
| VC_0748 | 1.97 | 0.17 | 9.73 | 1.00 | 3.11 | 0.05 | 0.353 |
| VC_0747 | 13.94 | 0.24 | 329.25 | 397.00 | -0.27 | 0.06 | 0.389 |
| VC_0746 | 16.00 | 0.00 | 429.53 | 396.00 | 0.11 | 0.05 | 0.907 |
| VC_0745 | 16.97 | 0.17 | 107.61 | 149.00 | -0.48 | 0.13 | 0.433 |
| VC_0744 | 21.84 | 0.37 | 487.08 | 314.00 | 0.63 | 0.03 | 0.340 |
| VC_0743 | 49.93 | 0.26 | 771.96 | 624.00 | 0.31 | 0.03 | 0.272 |
| VC_0742 | 6.95 | 0.22 | 58.95 | 5.00 | 3.53 | 0.01 | 0.020 |
| VC_0741 | 31.88 | 0.33 | 587.54 | 330.00 | 0.83 | 0.02 | 0.509 |
| VC_0740 | 7.00 | 0.00 | 80.59 | 179.00 | -1.17 | 0.28 | 0.440 |
| VC_0739 | 16.93 | 0.26 | 561.38 | 378.00 | 0.57 | 0.03 | 0.356 |
| VC_0738 | 4.00 | 0.00 | 75.56 | 124.00 | -0.74 | 0.23 | 0.594 |
| VC_0737 | 6.00 | 0.00 | 93.78 | 131.00 | -0.50 | 0.14 | 0.477 |

| | | | | | | | |
|---|---|---|---|---|---|---|---|
| VC_0736 | 29.15 | 0.74 | 541.55 | 352.00 | 0.62 | 0.03 | 0.299 |
| VC_0735 | 3.00 | 0.00 | 69.24 | 36.00 | 0.92 | 0.07 | 0.794 |
| VC_0734 | 26.18 | 1.07 | 374.40 | 604.00 | -0.69 | 0.08 | 0.904 |
| VC_0733 | 3.96 | 0.20 | 9.49 | 7.00 | 0.24 | 0.42 | 0.874 |
| VC_0732 | 27.71 | 0.50 | 461.68 | 364.00 | 0.34 | 0.03 | 0.859 |
| VC_0731 | 9.92 | 0.27 | 379.60 | 75.00 | 2.34 | 0.01 | 0.050 |
| VC_0730 | 14.56 | 0.50 | 181.84 | 288.00 | -0.67 | 0.12 | 0.042 |
| VC_0729 | 5.00 | 0.00 | 184.09 | 105.00 | 0.80 | 0.04 | 0.924 |
| VC_0728 | 20.55 | 0.58 | 494.45 | 209.00 | 1.24 | 0.02 | 0.160 |
| VC_0727 | 16.15 | 0.73 | 222.87 | 207.00 | 0.10 | 0.05 | 0.648 |
| VC_0726 | 25.58 | 0.54 | 231.89 | 310.00 | -0.43 | 0.09 | 0.230 |
| VC_0725 | 31.91 | 0.85 | 215.71 | 233.00 | -0.12 | 0.07 | 0.167 |
| VC_0724 | 37.81 | 0.85 | 303.66 | 360.00 | -0.25 | 0.06 | 0.887 |
| VC_0723 | 56.38 | 0.58 | 1226.04 | 869.00 | 0.50 | 0.02 | 0.373 |
| VC_0722 | 33.67 | 0.51 | 239.46 | 594.00 | -1.32 | 0.16 | 0.109 |
| VC_0721 | 20.00 | 0.00 | 346.03 | 384.00 | -0.15 | 0.06 | 0.854 |
| VC_0720 | 30.22 | 1.07 | 513.63 | 268.00 | 0.94 | 0.02 | 0.020 |
| VC_0719 | 18.89 | 0.31 | 439.26 | 633.00 | -0.53 | 0.07 | 0.760 |
| VC_0718 | 17.13 | 0.77 | 200.87 | 162.00 | 0.30 | 0.06 | 0.314 |
| VC_0717 | 33.56 | 0.54 | 827.06 | 981.00 | -0.25 | 0.04 | 0.753 |
| VC_0716 | 7.31 | 0.68 | 115.93 | 94.00 | 0.29 | 0.08 | 0.647 |
| VC_0715 | 26.83 | 0.89 | 346.36 | 348.00 | -0.01 | 0.05 | 0.248 |
| VC_0714 | 13.60 | 0.49 | 65.10 | 83.00 | -0.38 | 0.18 | 0.161 |
| VC_0713 | 6.00 | 0.00 | 32.28 | 22.00 | 0.51 | 0.12 | 0.711 |
| VC_0712 | 4.00 | 0.00 | 12.47 | 36.00 | -1.74 | 1.87 | 0.165 |
| VC_0711 | 53.88 | 1.22 | 940.66 | 664.00 | 0.50 | 0.02 | 0.268 |
| VC_0710 | 14.00 | 0.00 | 118.17 | 262.00 | -1.16 | 0.21 | 0.163 |
| VC_0709 | 22.93 | 0.26 | 472.92 | 168.00 | 1.49 | 0.02 | 0.359 |
| VC_0708 | 5.00 | 0.00 | 65.34 | 61.00 | 0.08 | 0.11 | 0.642 |
| VC_0707 | 3.00 | 0.00 | 24.26 | 46.00 | -1.01 | 0.51 | 0.580 |
| VC_0706 | 7.58 | 0.50 | 95.16 | 71.00 | 0.40 | 0.09 | 0.908 |
| VC_0705 | 29.46 | 0.66 | 353.03 | 398.00 | -0.18 | 0.06 | 0.646 |
| VC_0704 | 38.67 | 0.49 | 1123.31 | 976.00 | 0.20 | 0.03 | 0.755 |
| VC_0703 | 75.31 | 1.04 | 1253.46 | 1224.00 | 0.03 | 0.03 | 0.130 |
| VC_0702 | 15.92 | 0.27 | 167.13 | 182.00 | -0.13 | 0.09 | 0.604 |
| VC_0701 | 12.43 | 0.66 | 230.90 | 142.00 | 0.69 | 0.04 | 0.233 |
| VC_0700 | 63.80 | 0.90 | 1015.96 | 693.00 | 0.55 | 0.02 | 0.076 |
| VC_0699 | 4.00 | 0.00 | 11.49 | 13.00 | -0.34 | 0.54 | 0.770 |
| VC_0698 | 40.95 | 0.22 | 1302.68 | 1054.00 | 0.30 | 0.02 | 0.216 |
| VC_0697 | 17.83 | 0.38 | 522.46 | 593.00 | -0.19 | 0.05 | 0.932 |

| | | | | | | | |
|---|---|---|---|---|---|---|---|
| VC_0696 | 21.85 | 0.36 | 456.41 | 662.00 | -0.54 | 0.08 | 0.482 |
| VC_0695 | 22.15 | 0.73 | 277.81 | 274.00 | 0.02 | 0.05 | 0.588 |
| VC_0694 | 42.99 | 0.10 | 560.34 | 858.00 | -0.62 | 0.06 | 0.403 |
| VC_0693 | 14.96 | 0.20 | 323.82 | 182.00 | 0.83 | 0.03 | 0.435 |
| VC_0692 | 23.96 | 0.83 | 232.70 | 323.00 | -0.48 | 0.10 | 0.363 |
| VC_0691 | 22.87 | 0.34 | 344.52 | 493.00 | -0.52 | 0.09 | 0.671 |
| VC_0690 | 16.00 | 0.00 | 303.81 | 207.00 | 0.55 | 0.04 | 0.874 |
| VC_0689 | 34.13 | 0.85 | 532.99 | 443.00 | 0.26 | 0.04 | 0.198 |
| VC_0688 | 13.97 | 0.17 | 258.87 | 165.00 | 0.64 | 0.04 | 0.291 |
| VC_0687 | 45.54 | 0.58 | 824.03 | 942.00 | -0.19 | 0.04 | 0.449 |
| VC_0686 | 2.00 | 0.00 | 30.58 | 1.00 | 4.87 | 0.01 | 0.620 |
| VC_0685 | 1.00 | 0.00 | 13.63 | 1.00 | 3.67 | 0.02 | 1.000 |
| VC_0684 | 7.93 | 0.26 | 69.10 | 109.00 | -0.68 | 0.19 | 0.696 |
| VC_0683 | 1.00 | 0.00 | 1.60 | 15.00 | -3.52 | 4.23 | 1.000 |
| VC_0682 | 0.00 | 0.00 | 1.00 | 1.00 | #VALEUR! | NaN | NaN |
| VC_0681 | 2.00 | 0.00 | 13.05 | 17.00 | -0.57 | 0.59 | 1.000 |
| VC_0680 | 0.00 | 0.00 | 1.00 | 1.00 | #VALEUR! | NaN | NaN |
| VC_0679 | 0.00 | 0.00 | 1.00 | 1.00 | #VALEUR! | NaN | NaN |
| VC_0678 | 12.96 | 0.20 | 246.10 | 172.00 | 0.51 | 0.05 | 0.463 |
| VC_0677 | 32.59 | 0.53 | 439.66 | 326.00 | 0.43 | 0.04 | 0.657 |
| VC_0676 | 36.60 | 0.49 | 611.84 | 532.00 | 0.20 | 0.03 | 0.714 |
| VC_0675 | 1.00 | 0.00 | 1.00 | 39.00 | -5.29 | 0.00 | 1.000 |
| VC_0674 | 2.90 | 0.30 | 58.65 | 65.00 | -0.17 | 0.15 | 0.642 |
| VC_0673 | 24.48 | 0.64 | 378.50 | 582.00 | -0.62 | 0.08 | 0.348 |
| VC_0672 | 60.41 | 1.10 | 1103.29 | 1499.00 | -0.44 | 0.04 | 0.575 |
| VC_0671 | 11.40 | 0.72 | 94.75 | 149.00 | -0.67 | 0.16 | 0.849 |
| VC_0670 | 6.96 | 0.20 | 63.01 | 32.00 | 0.95 | 0.07 | 0.528 |
| VC_0669 | 2.97 | 0.17 | 31.02 | 1.00 | 4.90 | 0.01 | 0.418 |
| VC_0668 | 12.92 | 0.27 | 182.61 | 89.00 | 1.03 | 0.04 | 0.244 |
| VC_0667 | 34.45 | 0.66 | 484.24 | 802.00 | -0.73 | 0.08 | 0.909 |
| VC_0666 | 13.33 | 0.71 | 209.03 | 436.00 | -1.07 | 0.15 | 0.443 |
| VC_0665 | 36.82 | 0.88 | 1027.88 | 790.00 | 0.38 | 0.02 | 0.539 |
| VC_0664 | 1.00 | 0.00 | 1.00 | 22.00 | -4.46 | 0.00 | 1.000 |
| VC_0663 | 1.00 | 0.00 | 1.00 | 2.00 | -1.00 | 0.00 | 1.000 |
| VC_0662 | 40.00 | 0.00 | 753.88 | 580.00 | 0.38 | 0.02 | 0.246 |
| VC_0661 | 19.96 | 0.20 | 125.47 | 157.00 | -0.34 | 0.11 | 0.743 |
| VC_0660 | 9.92 | 0.27 | 18.73 | 34.00 | -0.96 | 0.56 | 0.534 |
| VC_0659 | 34.39 | 0.58 | 339.14 | 658.00 | -0.96 | 0.11 | 0.120 |
| VC_0658 | 55.83 | 0.87 | 635.30 | 930.00 | -0.55 | 0.07 | 0.349 |
| VC_0657 | 16.00 | 0.00 | 333.31 | 128.00 | 1.38 | 0.02 | 0.037 |

| | | | | | | | |
|---|---|---|---|---|---|---|---|
| VC_0656 | 0.00 | 0.00 | 1.00 | 1.00 | #VALEUR! | NaN | NaN |
| VC_0655 | 11.29 | 0.73 | 127.66 | 141.00 | -0.16 | 0.11 | 0.834 |
| VC_0654 | 12.70 | 0.46 | 146.95 | 164.00 | -0.17 | 0.08 | 0.147 |
| VC_0653 | 27.13 | 0.76 | 271.58 | 108.00 | 1.32 | 0.02 | 0.000 |
| VC_0652 | 22.93 | 0.29 | 414.94 | 310.00 | 0.42 | 0.04 | 0.655 |
| VC_0651 | 24.17 | 0.74 | 269.50 | 229.00 | 0.23 | 0.05 | 0.411 |
| VC_0650 | 47.09 | 0.77 | 1081.99 | 691.00 | 0.65 | 0.02 | 0.150 |
| VC_0649 | 8.83 | 0.38 | 124.50 | 47.00 | 1.39 | 0.04 | 0.320 |
| VC_0648 | 28.81 | 0.39 | 442.66 | 319.00 | 0.47 | 0.04 | 0.372 |
| VC_0647 | 16.72 | 0.49 | 43.45 | 76.00 | -0.84 | 0.30 | 0.237 |
| VC_0646 | 0.95 | 0.22 | 3.17 | 1.00 | 1.29 | 0.26 | 1.000 |
| VC_0645 | 26.37 | 0.68 | 388.05 | 740.00 | -0.94 | 0.10 | 0.480 |
| VC_0644 | 2.00 | 0.00 | 25.91 | 37.00 | -0.57 | 0.29 | 0.730 |
| VC_0643 | 2.00 | 0.00 | 43.25 | 1.00 | 5.40 | 0.00 | 0.333 |
| VC_0642 | 6.52 | 0.59 | 14.55 | 86.00 | -2.71 | 2.60 | 0.153 |
| VC_0641 | 1.00 | 0.00 | 1.00 | 1.00 | 0.00 | 0.00 | 1.000 |
| VC_0640 | 3.55 | 0.50 | 6.59 | 28.00 | -2.49 | 4.85 | 0.409 |
| VC_0639 | 2.00 | 0.00 | 6.69 | 2.00 | 1.46 | 0.22 | 1.000 |
| VC_0638 | 3.68 | 1.10 | 4.32 | 1.00 | 1.58 | 0.25 | 0.312 |
| VC_0637 | 1.00 | 0.00 | 1.00 | 1.00 | 0.00 | 0.00 | 1.000 |
| VC_0636 | 17.37 | 0.72 | 170.04 | 278.00 | -0.72 | 0.14 | 0.569 |
| VC_0635 | 8.00 | 0.00 | 142.84 | 69.00 | 1.04 | 0.04 | 0.320 |
| VC_0634 | 11.00 | 0.00 | 181.67 | 224.00 | -0.31 | 0.10 | 0.415 |
| VC_0633 | 39.27 | 0.84 | 576.62 | 498.00 | 0.21 | 0.04 | 0.209 |
| VC_0632 | 57.82 | 0.39 | 508.27 | 618.00 | -0.28 | 0.05 | 0.802 |
| VC_0631 | 1.00 | 0.00 | 1.54 | 4.00 | -1.65 | 1.10 | 1.000 |
| VC_0630 | 55.42 | 0.64 | 810.92 | 732.00 | 0.15 | 0.04 | 0.621 |
| VC_0629 | 88.31 | 1.14 | 1261.90 | 1797.00 | -0.51 | 0.04 | 0.224 |
| VC_0628 | 14.36 | 0.67 | 194.40 | 111.00 | 0.80 | 0.04 | 0.137 |
| VC_0627 | 2.00 | 0.00 | 45.53 | 4.00 | 3.48 | 0.01 | 0.333 |
| VC_0626 | 1.89 | 0.31 | 20.13 | 1.00 | 4.25 | 0.01 | 0.407 |
| VC_0625 | 5.64 | 0.48 | 87.11 | 49.00 | 0.81 | 0.07 | 0.502 |
| VC_0624 | 32.97 | 0.17 | 659.40 | 717.00 | -0.12 | 0.04 | 0.862 |
| VC_0623 | 34.62 | 0.53 | 521.99 | 277.00 | 0.91 | 0.03 | 0.016 |
| VC_0622 | 113.48 | 1.05 | 1932.47 | 1703.00 | 0.18 | 0.02 | 0.089 |
| VC_0621 | 11.87 | 0.34 | 193.74 | 126.00 | 0.61 | 0.05 | 0.494 |
| VC_0620 | 62.54 | 0.54 | 1285.35 | 906.00 | 0.50 | 0.02 | 0.229 |
| VC_0619 | 28.99 | 0.10 | 858.34 | 1261.00 | -0.56 | 0.05 | 0.935 |
| VC_0618 | 26.45 | 0.64 | 485.00 | 561.00 | -0.21 | 0.05 | 0.184 |
| VC_0617 | 30.98 | 0.14 | 555.29 | 422.00 | 0.39 | 0.03 | 0.620 |

| | | | | | | | |
|---|---|---|---|---|---|---|---|
| VC_0616 | 26.61 | 0.58 | 776.03 | 707.00 | 0.13 | 0.03 | 0.363 |
| VC_0615 | 36.20 | 0.71 | 623.76 | 588.00 | 0.08 | 0.04 | 0.711 |
| VC_0614 | 24.98 | 0.14 | 573.77 | 630.00 | -0.14 | 0.04 | 0.747 |
| VC_0613 | 52.65 | 0.48 | 396.47 | 1117.00 | -1.50 | 0.14 | 0.131 |
| VC_0612 | 66.80 | 1.01 | 1353.86 | 1483.00 | -0.13 | 0.03 | 0.850 |
| VC_0611 | 32.83 | 0.87 | 346.19 | 647.00 | -0.91 | 0.10 | 0.310 |
| VC_0610 | 22.79 | 0.46 | 377.21 | 447.00 | -0.25 | 0.06 | 0.759 |
| VC_0609 | 52.95 | 0.76 | 560.99 | 527.00 | 0.09 | 0.04 | 0.718 |
| VC_0608 | 29.54 | 0.52 | 781.83 | 576.00 | 0.44 | 0.03 | 0.889 |
| VC_0607 | 23.98 | 0.14 | 533.83 | 352.00 | 0.60 | 0.03 | 0.621 |
| VC_0606 | 6.00 | 0.00 | 43.92 | 32.00 | 0.42 | 0.13 | 0.494 |
| VC_0605 | 11.95 | 0.22 | 246.96 | 73.00 | 1.75 | 0.02 | 0.203 |
| VC_0604 | 68.80 | 0.90 | 653.82 | 413.00 | 0.66 | 0.03 | 0.237 |
| VC_0603 | 61.34 | 1.11 | 727.03 | 949.00 | -0.39 | 0.06 | 0.264 |
| VC_0602 | 51.93 | 0.86 | 681.52 | 906.00 | -0.41 | 0.05 | 0.853 |
| VC_0601 | 37.60 | 0.49 | 918.12 | 759.00 | 0.27 | 0.03 | 0.605 |
| VC_0600 | 22.46 | 0.67 | 324.24 | 239.00 | 0.44 | 0.04 | 0.762 |
| VC_0599 | 2.00 | 0.00 | 46.85 | 52.00 | -0.18 | 0.17 | 0.867 |
| VC_0598 | 4.00 | 0.00 | 84.08 | 27.00 | 1.62 | 0.04 | 0.133 |
| VC_0597 | 20.94 | 0.28 | 252.47 | 240.00 | 0.07 | 0.06 | 0.199 |
| VC_0596 | 3.00 | 0.00 | 7.72 | 5.00 | 0.45 | 0.28 | 0.988 |
| VC_0595 | 20.83 | 0.90 | 357.44 | 235.00 | 0.60 | 0.03 | 0.705 |
| VC_0594 | 25.59 | 0.53 | 462.04 | 312.00 | 0.56 | 0.03 | 0.091 |
| VC_0593 | 1.98 | 0.14 | 3.25 | 24.00 | -3.32 | 6.22 | 1.000 |
| VC_0592 | 1.83 | 0.38 | 9.71 | 22.00 | -1.33 | 0.87 | 1.000 |
| VC_0591 | 3.65 | 0.48 | 2.31 | 6.00 | -1.92 | 1.98 | 0.439 |
| VC_0590 | 26.50 | 0.59 | 410.49 | 598.00 | -0.55 | 0.08 | 0.810 |
| VC_0589 | 17.17 | 0.79 | 360.45 | 106.00 | 1.76 | 0.02 | 0.071 |
| VC_0588 | 5.64 | 0.48 | 103.31 | 39.00 | 1.39 | 0.04 | 0.888 |
| VC_0587 | 39.51 | 0.61 | 837.18 | 856.00 | -0.03 | 0.03 | 0.384 |
| VC_0586 | 24.17 | 0.73 | 500.76 | 223.00 | 1.16 | 0.02 | 0.300 |
| VC_0585 | 18.83 | 0.38 | 251.13 | 151.00 | 0.73 | 0.04 | 0.672 |
| VC_0584 | 4.00 | 0.00 | 120.78 | 162.00 | -0.43 | 0.12 | 0.205 |
| VC_0583 | 18.92 | 0.27 | 270.01 | 318.00 | -0.24 | 0.08 | 0.834 |
| VC_0582 | 26.54 | 0.59 | 246.95 | 466.00 | -0.92 | 0.12 | 0.912 |
| VC_0581 | 30.66 | 0.48 | 36.48 | 312.00 | -3.15 | 1.79 | 0.000 |
| VC_0580 | 12.85 | 0.39 | 399.53 | 156.00 | 1.35 | 0.02 | 0.058 |
| VC_0579 | 13.90 | 0.30 | 237.62 | 98.00 | 1.27 | 0.03 | 0.126 |
| VC_0578 | 13.99 | 0.10 | 282.01 | 85.00 | 1.72 | 0.02 | 0.155 |
| VC_0577 | 14.83 | 0.40 | 120.45 | 279.00 | -1.22 | 0.21 | 0.196 |

| | | | | | | | |
|---|---|---|---|---|---|---|---|
| VC_0576 | 14.27 | 0.79 | 92.88 | 63.00 | 0.54 | 0.08 | 0.578 |
| VC_0575 | 21.99 | 0.10 | 231.46 | 352.00 | -0.61 | 0.11 | 0.353 |
| VC_0574 | 45.63 | 0.58 | 1044.09 | 490.00 | 1.09 | 0.01 | 0.251 |
| VC_0573 | 15.84 | 0.37 | 169.48 | 255.00 | -0.60 | 0.13 | 0.460 |
| VC_0572 | 2.72 | 0.47 | 10.39 | 3.00 | 1.64 | 0.11 | 0.341 |
| VC_0571 | 0.00 | 0.00 | 1.00 | 1.00 | #VALEUR! | NaN | NaN |
| VC_0570 | 0.00 | 0.00 | 1.00 | 1.00 | #VALEUR! | NaN | NaN |
| VC_0569 | 0.00 | 0.00 | 1.00 | 1.00 | #VALEUR! | NaN | NaN |
| VC_0568 | 44.00 | 0.82 | 492.52 | 583.00 | -0.25 | 0.05 | 0.436 |
| VC_0567 | 13.08 | 0.69 | 45.14 | 265.00 | -2.59 | 0.95 | 0.050 |
| VC_0566 | 29.10 | 0.78 | 345.99 | 500.00 | -0.54 | 0.10 | 0.444 |
| VC_0565 | 3.00 | 0.00 | 80.80 | 129.00 | -0.69 | 0.18 | 0.543 |
| VC_0564 | 0.00 | 0.00 | 1.00 | 1.00 | #VALEUR! | NaN | NaN |
| VC_0563 | 0.00 | 0.00 | 1.00 | 1.00 | #VALEUR! | NaN | NaN |
| VC_0562 | 0.00 | 0.00 | 1.00 | 1.00 | #VALEUR! | NaN | NaN |
| VC_0561 | 0.00 | 0.00 | 1.00 | 1.00 | #VALEUR! | NaN | NaN |
| VC_0560 | 1.00 | 0.00 | 1.00 | 7.00 | -2.81 | 0.00 | 1.000 |
| VC_0559 | 23.74 | 0.80 | 453.90 | 240.00 | 0.92 | 0.02 | 0.385 |
| VC_0558 | 41.33 | 0.70 | 505.27 | 549.00 | -0.12 | 0.05 | 0.806 |
| VC_0557 | 13.98 | 0.14 | 60.87 | 224.00 | -1.91 | 0.54 | 0.040 |
| VC_0556 | 36.16 | 0.72 | 246.01 | 338.00 | -0.46 | 0.08 | 0.207 |
| VC_0555 | 4.98 | 0.14 | 21.42 | 63.00 | -1.63 | 0.77 | 0.661 |
| VC_0554 | 66.68 | 0.91 | 1120.28 | 1389.00 | -0.31 | 0.04 | 0.592 |
| VC_0553 | 15.88 | 0.36 | 271.41 | 119.00 | 1.18 | 0.03 | 0.032 |
| VC_0552 | 28.66 | 0.57 | 517.72 | 478.00 | 0.11 | 0.04 | 0.111 |
| VC_0551 | 28.96 | 0.20 | 549.18 | 432.00 | 0.34 | 0.03 | 0.584 |
| VC_0550 | 39.53 | 0.61 | 927.58 | 788.00 | 0.23 | 0.03 | 0.559 |
| VC_0549 | 6.98 | 0.14 | 113.69 | 25.00 | 2.17 | 0.03 | 0.012 |
| VC_0548 | 2.00 | 0.00 | 45.65 | 16.00 | 1.48 | 0.06 | 0.383 |
| VC_0547 | 35.75 | 0.48 | 484.06 | 358.00 | 0.43 | 0.04 | 0.814 |
| VC_0546 | 7.00 | 0.00 | 63.08 | 122.00 | -0.97 | 0.24 | 0.188 |
| VC_0545 | 1.00 | 0.00 | 16.23 | 20.00 | -0.40 | 0.37 | 1.000 |
| VC_0544 | 14.94 | 0.24 | 149.38 | 191.00 | -0.36 | 0.11 | 0.714 |
| VC_0543 | 24.17 | 0.77 | 359.05 | 322.00 | 0.15 | 0.05 | 0.504 |
| VC_0542 | 8.00 | 0.00 | 69.61 | 139.00 | -1.02 | 0.23 | 0.333 |
| VC_0541 | 20.67 | 0.57 | 146.43 | 145.00 | 0.00 | 0.09 | 0.590 |
| VC_0540 | 25.33 | 0.64 | 219.97 | 159.00 | 0.46 | 0.06 | 0.855 |
| VC_0539 | 18.55 | 0.59 | 86.29 | 115.00 | -0.43 | 0.14 | 0.099 |
| VC_0538 | 23.19 | 0.76 | 463.65 | 308.00 | 0.59 | 0.03 | 0.116 |
| VC_0537 | 24.95 | 0.22 | 121.92 | 364.00 | -1.59 | 0.26 | 0.034 |

| | | | | | | | |
|---|---|---|---|---|---|---|---|
| VC_0536 | 2.00 | 0.00 | 40.83 | 30.00 | 0.41 | 0.13 | 0.800 |
| VC_0535 | 66.39 | 0.63 | 824.12 | 1567.00 | -0.93 | 0.06 | 0.194 |
| VC_0534 | 19.84 | 0.37 | 154.45 | 649.00 | -2.08 | 0.34 | 0.195 |
| VC_0533 | 40.64 | 0.95 | 522.78 | 842.00 | -0.69 | 0.07 | 0.639 |
| VC_0532 | 10.88 | 0.33 | 179.87 | 436.00 | -1.28 | 0.18 | 0.413 |
| VC_0531 | 20.51 | 0.52 | 411.31 | 588.00 | -0.52 | 0.06 | 0.835 |
| VC_0530 | 25.35 | 0.59 | 281.12 | 270.00 | 0.05 | 0.06 | 0.303 |
| VC_0529 | 0.96 | 0.20 | 3.56 | 1.00 | 1.47 | 0.22 | 1.000 |
| VC_0528 | 2.86 | 0.35 | 2.04 | 14.00 | -3.18 | 4.28 | 0.412 |
| VC_0527 | 0.00 | 0.00 | 1.00 | 1.00 | #VALEUR! | NaN | NaN |
| VC_0526 | 21.30 | 0.73 | 414.36 | 307.00 | 0.43 | 0.04 | 0.771 |
| VC_0525 | 12.00 | 0.00 | 153.94 | 224.00 | -0.55 | 0.11 | 0.446 |
| VC_0524 | 0.00 | 0.00 | 1.00 | 1.00 | #VALEUR! | NaN | NaN |
| VC_0523 | 10.74 | 0.44 | 324.53 | 182.00 | 0.83 | 0.03 | 0.716 |
| VC_0522 | 23.66 | 0.54 | 163.55 | 584.00 | -1.85 | 0.31 | 0.010 |
| VC_0521 | 1.00 | 0.00 | 10.92 | 13.00 | -0.41 | 0.53 | 1.000 |
| VC_0520 | 0.83 | 0.38 | 2.16 | 1.00 | 0.80 | 0.31 | 1.000 |
| VC_0519 | 6.76 | 0.45 | 9.09 | 53.00 | -2.68 | 2.40 | 0.307 |
| VC_0518 | 2.00 | 0.00 | 6.23 | 4.00 | 0.36 | 0.52 | 1.000 |
| VC_0517 | 0.00 | 0.00 | 1.00 | 1.00 | #VALEUR! | NaN | NaN |
| VC_0516 | 57.27 | 0.76 | 729.17 | 782.00 | -0.10 | 0.04 | 0.580 |
| VC_0515 | 19.85 | 1.24 | 117.84 | 75.00 | 0.64 | 0.06 | 0.273 |
| VC_0514 | 45.42 | 0.73 | 344.43 | 255.00 | 0.43 | 0.04 | 0.709 |
| VC_0513 | 8.62 | 0.97 | 18.76 | 43.00 | -1.28 | 0.63 | 0.232 |
| VC_0512 | 82.67 | 0.85 | 1548.64 | 1532.00 | 0.01 | 0.02 | 0.428 |
| VC_0511 | 7.90 | 0.30 | 86.19 | 103.00 | -0.28 | 0.15 | 0.874 |
| VC_0510 | 11.94 | 0.24 | 496.28 | 371.00 | 0.42 | 0.03 | 0.616 |
| VC_0509 | 13.00 | 0.00 | 306.11 | 240.00 | 0.35 | 0.05 | 0.300 |
| VC_0508 | 19.43 | 0.57 | 446.32 | 212.00 | 1.07 | 0.03 | 0.195 |
| VC_0507 | 3.00 | 0.00 | 71.36 | 62.00 | 0.19 | 0.10 | 0.982 |
| VC_0506 | 16.89 | 0.31 | 331.21 | 312.00 | 0.08 | 0.06 | 0.720 |
| VC_0505 | 10.00 | 0.00 | 197.56 | 301.00 | -0.61 | 0.11 | 0.471 |
| VC_0504 | 8.91 | 0.29 | 88.57 | 82.00 | 0.09 | 0.11 | 0.141 |
| VC_0503 | 51.44 | 1.04 | 755.92 | 691.00 | 0.13 | 0.03 | 0.804 |
| VC_0502 | 25.78 | 0.92 | 481.83 | 452.00 | 0.09 | 0.04 | 0.447 |
| VC_0501 | 30.97 | 0.17 | 475.09 | 304.00 | 0.64 | 0.03 | 0.730 |
| VC_0500 | 7.00 | 0.00 | 166.19 | 267.00 | -0.69 | 0.13 | 0.589 |
| VC_0499 | 24.75 | 0.44 | 716.26 | 490.00 | 0.55 | 0.02 | 0.487 |
| VC_0498 | 16.58 | 0.50 | 198.19 | 358.00 | -0.86 | 0.12 | 0.669 |
| VC_0497 | 9.00 | 0.00 | 358.14 | 339.00 | 0.08 | 0.05 | 0.802 |

| | | | | | | | |
|---|---|---|---|---|---|---|---|
| VC_0496 | 29.80 | 0.45 | 506.50 | 320.00 | 0.66 | 0.03 | 0.197 |
| VC_0495 | 21.63 | 0.49 | 197.58 | 164.00 | 0.26 | 0.06 | 0.638 |
| VC_0494 | 29.47 | 0.67 | 485.33 | 286.00 | 0.76 | 0.03 | 0.004 |
| VC_0493 | 28.55 | 0.61 | 224.60 | 263.00 | -0.23 | 0.08 | 0.463 |
| VC_0492 | 58.71 | 0.46 | 509.35 | 611.00 | -0.27 | 0.05 | 0.894 |
| VC_0491 | 12.92 | 0.91 | 33.23 | 104.00 | -1.69 | 0.63 | 0.477 |
| VC_0490 | 83.55 | 1.40 | 1044.89 | 778.00 | 0.42 | 0.02 | 0.109 |
| VC_0489 | 60.26 | 0.77 | 1010.85 | 1107.00 | -0.13 | 0.04 | 0.441 |
| VC_0488 | 31.98 | 0.14 | 767.23 | 566.00 | 0.44 | 0.03 | 0.770 |
| VC_0487 | 0.00 | 0.00 | 1.00 | 1.00 | #VALEUR! | NaN | NaN |
| VC_0486 | 14.96 | 0.20 | 66.56 | 115.00 | -0.82 | 0.25 | 0.075 |
| VC_0485 | 19.44 | 0.69 | 431.09 | 190.00 | 1.18 | 0.02 | 0.329 |
| VC_0484 | 6.62 | 0.49 | 49.56 | 77.00 | -0.66 | 0.24 | 0.646 |
| VC_0483 | 22.99 | 0.10 | 637.50 | 360.00 | 0.82 | 0.02 | 0.607 |
| VC_0482 | 29.28 | 0.71 | 302.26 | 453.00 | -0.59 | 0.10 | 0.019 |
| VC_0481 | 18.84 | 0.37 | 220.34 | 208.00 | 0.08 | 0.07 | 0.498 |
| VC_0480 | 23.33 | 0.71 | 489.78 | 309.00 | 0.66 | 0.03 | 0.306 |
| VC_0479 | 4.84 | 0.37 | 15.81 | 21.00 | -0.52 | 0.41 | 0.751 |
| VC_0478 | 2.00 | 0.00 | 47.78 | 15.00 | 1.64 | 0.05 | 1.000 |
| VC_0477 | 1.00 | 0.00 | 1.00 | 1.00 | 0.00 | 0.00 | 1.000 |
| VC_0476 | 39.06 | 0.80 | 1434.62 | 1500.00 | -0.07 | 0.03 | 0.199 |
| VC_0475 | 66.47 | 0.61 | 1713.42 | 1301.00 | 0.40 | 0.02 | 0.729 |
| VC_0474 | 17.00 | 0.00 | 142.79 | 224.00 | -0.66 | 0.13 | 0.897 |
| VC_0473 | 49.00 | 0.84 | 849.00 | 1295.00 | -0.61 | 0.06 | 0.285 |
| VC_0472 | 2.00 | 0.00 | 16.06 | 1.00 | 3.90 | 0.02 | 0.333 |
| VC_0471 | 12.62 | 0.51 | 362.28 | 173.00 | 1.06 | 0.03 | 0.301 |
| VC_0470 | 13.88 | 0.33 | 234.27 | 465.00 | -0.99 | 0.12 | 0.293 |
| VC_0469 | 16.24 | 0.68 | 313.34 | 357.00 | -0.19 | 0.07 | 0.718 |
| VC_0468 | 25.94 | 0.24 | 295.99 | 299.00 | -0.02 | 0.06 | 0.571 |
| VC_0467 | 16.97 | 0.17 | 223.06 | 259.00 | -0.22 | 0.08 | 0.416 |
| VC_0466 | 0.00 | 0.00 | 1.00 | 1.00 | #VALEUR! | NaN | NaN |
| VC_0465 | 26.94 | 0.24 | 650.59 | 808.00 | -0.31 | 0.05 | 0.661 |
| VC_0464 | 17.72 | 0.47 | 385.04 | 437.00 | -0.19 | 0.06 | 0.611 |
| VC_0463 | 20.66 | 0.48 | 241.38 | 407.00 | -0.76 | 0.11 | 0.141 |
| VC_0462 | 27.41 | 0.64 | 411.48 | 349.00 | 0.23 | 0.04 | 0.372 |
| VC_0461 | 17.70 | 0.48 | 313.65 | 298.00 | 0.07 | 0.05 | 0.912 |
| VC_0460 | 12.00 | 0.00 | 91.95 | 118.00 | -0.38 | 0.15 | 0.888 |
| VC_0459 | 11.97 | 0.17 | 29.59 | 76.00 | -1.41 | 0.49 | 0.335 |
| VC_0458 | 9.00 | 0.00 | 251.75 | 192.00 | 0.38 | 0.05 | 0.294 |
| VC_0457 | 16.52 | 0.63 | 185.88 | 152.00 | 0.28 | 0.06 | 0.645 |

| | | | | | | | |
|---|---|---|---|---|---|---|---|
| VC_0456 | 10.00 | 0.00 | 126.39 | 209.00 | -0.73 | 0.13 | 0.059 |
| VC_0455 | 25.19 | 0.81 | 343.34 | 362.00 | -0.08 | 0.06 | 0.506 |
| VC_0454 | 19.72 | 0.47 | 241.59 | 432.00 | -0.84 | 0.11 | 0.571 |
| VC_0453 | 9.00 | 0.00 | 179.35 | 103.00 | 0.79 | 0.04 | 0.747 |
| VC_0452 | 22.97 | 0.17 | 432.78 | 348.00 | 0.31 | 0.04 | 0.762 |
| VC_0451 | 3.99 | 0.10 | 67.21 | 38.00 | 0.80 | 0.08 | 0.665 |
| VC_0450 | 38.77 | 0.47 | 541.52 | 412.00 | 0.39 | 0.04 | 0.819 |
| VC_0449 | 47.85 | 0.39 | 930.58 | 963.00 | -0.05 | 0.03 | 0.452 |
| VC_0448 | 4.89 | 0.31 | 21.99 | 24.00 | -0.18 | 0.23 | 0.690 |
| VC_0447 | 16.84 | 0.37 | 218.80 | 149.00 | 0.55 | 0.04 | 0.669 |
| VC_0446 | 4.52 | 0.50 | 20.53 | 26.00 | -0.44 | 0.38 | 0.378 |
| VC_0445 | 1.00 | 0.00 | 1.00 | 3.00 | -1.58 | 0.00 | 1.000 |
| VC_0444 | 0.00 | 0.00 | 1.00 | 1.00 | #VALEUR! | NaN | NaN |
| VC_0443 | 2.93 | 0.29 | 8.45 | 4.00 | 0.81 | 0.41 | 0.520 |
| VC_0442 | 8.68 | 0.47 | 238.20 | 167.00 | 0.51 | 0.04 | 0.864 |
| VC_0441 | 25.67 | 0.60 | 214.53 | 330.00 | -0.63 | 0.11 | 0.492 |
| VC_0440 | 2.72 | 0.45 | 10.11 | 27.00 | -1.60 | 1.47 | 0.567 |
| VC_0439 | 16.48 | 0.61 | 122.00 | 273.00 | -1.17 | 0.18 | 0.109 |
| VC_0438 | 24.35 | 0.67 | 339.80 | 383.00 | -0.18 | 0.05 | 0.552 |
| VC_0437 | 7.00 | 0.00 | 50.60 | 101.00 | -1.03 | 0.33 | 0.445 |
| VC_0436 | 0.00 | 0.00 | 1.00 | 1.00 | #VALEUR! | NaN | NaN |
| VC_0435 | 0.00 | 0.00 | 1.00 | 1.00 | #VALEUR! | NaN | NaN |
| VC_0434 | 5.00 | 0.00 | 50.94 | 100.00 | -1.00 | 0.30 | 0.531 |
| VC_0433 | 44.32 | 0.66 | 525.89 | 719.00 | -0.45 | 0.05 | 0.491 |
| VC_0432 | 31.82 | 0.39 | 630.73 | 489.00 | 0.37 | 0.03 | 0.423 |
| VC_0431 | 19.98 | 0.14 | 177.61 | 331.00 | -0.91 | 0.14 | 0.657 |
| VC_0430 | 32.64 | 0.48 | 451.65 | 409.00 | 0.14 | 0.05 | 0.736 |
| VC_0429 | 75.15 | 0.87 | 922.43 | 1260.00 | -0.45 | 0.05 | 0.657 |
| VC_0428 | 13.83 | 0.84 | 182.04 | 247.00 | -0.45 | 0.11 | 0.517 |
| VC_0427 | 3.00 | 0.00 | 5.01 | 83.00 | -4.32 | 10.12 | 0.199 |
| VC_0426 | 12.66 | 0.48 | 529.99 | 218.00 | 1.28 | 0.02 | 0.027 |
| VC_0425 | 16.60 | 0.51 | 457.56 | 294.00 | 0.63 | 0.03 | 0.285 |
| VC_0424 | 12.59 | 0.55 | 131.42 | 227.00 | -0.80 | 0.15 | 0.501 |
| VC_0423 | 30.19 | 0.80 | 663.05 | 448.00 | 0.56 | 0.03 | 0.307 |
| VC_0422 | 31.23 | 0.76 | 407.75 | 647.00 | -0.67 | 0.09 | 0.085 |
| VC_0421 | 21.63 | 0.49 | 367.50 | 330.00 | 0.15 | 0.04 | 0.650 |
| VC_0420 | 88.51 | 1.22 | 1539.82 | 1184.00 | 0.38 | 0.02 | 0.368 |
| VC_0419 | 24.95 | 0.89 | 501.33 | 426.00 | 0.23 | 0.04 | 0.326 |
| VC_0418 | 11.97 | 0.17 | 140.35 | 145.00 | -0.06 | 0.09 | 0.854 |
| VC_0417 | 0.00 | 0.00 | 1.00 | 1.00 | #VALEUR! | NaN | NaN |

| | | | | | | | |
|---|---|---|---|---|---|---|---|
| VC_0416 | 1.99 | 0.10 | 6.33 | 1.00 | 2.26 | 0.15 | 0.440 |
| VC_0415 | 0.70 | 0.46 | 1.39 | 1.00 | 0.38 | 0.27 | 1.000 |
| VC_0414 | 140.62 | 1.41 | 2210.61 | 1737.00 | 0.35 | 0.02 | 0.076 |
| VC_0413 | 13.00 | 0.00 | 234.98 | 157.00 | 0.58 | 0.04 | 0.039 |
| VC_0412 | 34.92 | 0.88 | 388.27 | 485.00 | -0.32 | 0.06 | 0.088 |
| VC_0411 | 25.58 | 0.55 | 527.49 | 397.00 | 0.41 | 0.03 | 0.877 |
| VC_0410 | 18.86 | 0.35 | 522.47 | 346.00 | 0.59 | 0.03 | 0.857 |
| VC_0409 | 32.00 | 0.00 | 451.60 | 540.00 | -0.26 | 0.07 | 0.186 |
| VC_0408 | 18.22 | 0.72 | 233.45 | 115.00 | 1.02 | 0.03 | 0.728 |
| VC_0407 | 14.00 | 0.00 | 304.06 | 267.00 | 0.18 | 0.05 | 0.656 |
| VC_0406 | 29.31 | 0.77 | 416.86 | 330.00 | 0.33 | 0.04 | 0.896 |
| VC_0405 | 41.21 | 0.73 | 815.64 | 765.00 | 0.09 | 0.04 | 0.772 |
| VC_0404 | 27.49 | 0.59 | 476.47 | 716.00 | -0.59 | 0.07 | 0.168 |
| VC_0403 | 16.92 | 0.27 | 473.78 | 425.00 | 0.15 | 0.04 | 0.738 |
| VC_0402 | 60.48 | 0.58 | 1062.04 | 799.00 | 0.41 | 0.03 | 0.624 |
| VC_0401 | 12.68 | 0.47 | 328.33 | 181.00 | 0.86 | 0.03 | 0.835 |
| VC_0400 | 18.95 | 0.22 | 344.06 | 253.00 | 0.44 | 0.04 | 0.336 |
| VC_0399 | 38.14 | 0.78 | 833.33 | 851.00 | -0.03 | 0.03 | 0.639 |
| VC_0398 | 67.68 | 1.05 | 1279.20 | 1532.00 | -0.26 | 0.03 | 0.232 |
| VC_0397 | 1.00 | 0.00 | 8.69 | 3.00 | 1.26 | 0.30 | 1.000 |
| VC_0396 | 15.68 | 0.47 | 262.34 | 193.00 | 0.44 | 0.04 | 0.260 |
| VC_0395 | 18.95 | 0.22 | 113.02 | 190.00 | -0.76 | 0.17 | 0.795 |
| VC_0394 | 71.71 | 0.99 | 1146.12 | 1873.00 | -0.71 | 0.05 | 0.023 |
| VC_0393 | 80.12 | 0.78 | 1434.57 | 1460.00 | -0.03 | 0.03 | 0.365 |
| VC_0392 | 23.84 | 0.37 | 511.93 | 376.00 | 0.44 | 0.03 | 0.854 |
| VC_0391 | 31.39 | 0.63 | 720.58 | 1093.00 | -0.60 | 0.05 | 0.916 |
| VC_0390 | 93.84 | 0.83 | 1899.76 | 2029.00 | -0.10 | 0.02 | 0.899 |
| VC_0389 | 47.14 | 0.82 | 529.00 | 879.00 | -0.74 | 0.07 | 0.129 |
| VC_0388 | 5.65 | 0.48 | 14.63 | 22.00 | -0.67 | 0.42 | 0.792 |
| VC_0387 | 2.00 | 0.00 | 19.03 | 64.00 | -1.82 | 0.83 | 0.383 |
| VC_0386 | 28.55 | 0.98 | 429.28 | 250.00 | 0.78 | 0.03 | 0.021 |
| VC_0385 | 39.18 | 0.73 | 816.62 | 372.00 | 1.13 | 0.02 | 0.150 |
| VC_0384 | 49.15 | 0.88 | 979.16 | 917.00 | 0.09 | 0.03 | 0.069 |
| VC_0383 | 5.00 | 0.00 | 83.98 | 41.00 | 1.02 | 0.05 | 0.443 |
| VC_0382 | 7.00 | 0.00 | 54.96 | 69.00 | -0.36 | 0.18 | 0.562 |
| VC_0381 | 24.00 | 0.00 | 358.54 | 418.00 | -0.23 | 0.06 | 0.803 |
| VC_0380.1 | 10.81 | 0.39 | 417.06 | 509.00 | -0.29 | 0.06 | 0.937 |
| VC_0380 | 1.90 | 0.30 | 26.87 | 1.00 | 4.70 | 0.01 | 0.400 |
| VC_0379 | 22.00 | 0.00 | 347.61 | 439.00 | -0.34 | 0.07 | 0.559 |
| VC_0378 | 13.56 | 0.50 | 255.08 | 131.00 | 0.95 | 0.04 | 0.208 |

| | | | | | | | |
|---|---|---|---|---|---|---|---|
| VC_0377 | 10.92 | 0.27 | 96.62 | 106.00 | -0.15 | 0.11 | 0.941 |
| VC_0376 | 40.65 | 0.52 | 707.87 | 829.00 | -0.23 | 0.04 | 0.287 |
| VC_0375 | 3.64 | 0.48 | 13.42 | 27.00 | -1.15 | 0.77 | 0.681 |
| VC_0374 | 34.50 | 0.56 | 333.12 | 340.00 | -0.03 | 0.06 | 0.775 |
| VC_0373 | 14.96 | 0.20 | 346.09 | 522.00 | -0.60 | 0.08 | 0.622 |
| VC_0372 | 3.00 | 0.00 | 42.09 | 3.00 | 3.78 | 0.01 | 0.106 |
| VC_0371 | 1.00 | 0.00 | 62.77 | 19.00 | 1.70 | 0.04 | 1.000 |
| VC_0370 | 34.58 | 1.08 | 259.90 | 477.00 | -0.88 | 0.12 | 0.252 |
| VC_0369 | 2.51 | 0.61 | 10.54 | 1.00 | 3.25 | 0.04 | 0.240 |
| VC_0368 | 2.00 | 0.00 | 25.96 | 20.00 | 0.30 | 0.20 | 0.947 |
| VC_0367 | 0.00 | 0.00 | 1.00 | 1.00 | #VALEUR! | NaN | NaN |
| VC_0366 | 0.00 | 0.00 | 1.00 | 1.00 | #VALEUR! | NaN | NaN |
| VC_0365 | 16.29 | 0.70 | 139.00 | 190.00 | -0.46 | 0.10 | 0.425 |
| VC_0364 | 8.00 | 0.00 | 209.85 | 59.00 | 1.82 | 0.02 | 0.192 |
| VC_0363 | 2.00 | 0.00 | 1.30 | 23.00 | -4.33 | 5.19 | 0.403 |
| VC_0362 | 47.93 | 0.81 | 800.59 | 438.00 | 0.87 | 0.02 | 0.231 |
| VC_0361 | 0.00 | 0.00 | 1.00 | 1.00 | #VALEUR! | NaN | NaN |
| VC_0360 | 0.00 | 0.00 | 1.00 | 1.00 | #VALEUR! | NaN | NaN |
| VC_0359 | 0.00 | 0.00 | 1.00 | 1.00 | #VALEUR! | NaN | NaN |
| VC_0358 | 1.66 | 0.48 | 1.45 | 2.00 | -0.71 | 0.53 | 1.000 |
| VC_0357 | 1.00 | 0.00 | 1.46 | 25.00 | -4.35 | 6.63 | 1.000 |
| VC_0356 | 0.00 | 0.00 | 1.00 | 1.00 | #VALEUR! | NaN | NaN |
| VC_0355 | 19.80 | 0.40 | 327.96 | 149.00 | 1.13 | 0.02 | 0.065 |
| VC_0354 | 16.45 | 0.59 | 142.99 | 311.00 | -1.13 | 0.18 | 0.231 |
| VC_0353 | 24.94 | 0.24 | 401.41 | 473.00 | -0.24 | 0.06 | 0.933 |
| VC_0352 | 5.64 | 0.48 | 102.78 | 22.00 | 2.21 | 0.02 | 0.222 |
| VC_0351 | 8.00 | 0.00 | 141.84 | 222.00 | -0.66 | 0.15 | 0.784 |
| VC_0350 | 28.54 | 0.56 | 238.40 | 233.00 | 0.03 | 0.07 | 0.119 |
| VC_0349 | 30.36 | 1.12 | 339.29 | 347.00 | -0.04 | 0.06 | 0.825 |
| VC_0348 | 42.53 | 0.56 | 723.54 | 605.00 | 0.26 | 0.03 | 0.834 |
| VC_0347 | 4.00 | 0.00 | 3.99 | 20.00 | -2.67 | 3.73 | 0.310 |
| VC_0346 | 25.65 | 0.48 | 326.92 | 311.00 | 0.07 | 0.06 | 0.622 |
| VC_0345 | 54.38 | 1.03 | 1076.01 | 1243.00 | -0.21 | 0.03 | 0.455 |
| VC_0344 | 17.62 | 0.49 | 166.41 | 125.00 | 0.41 | 0.06 | 0.575 |
| VC_0343 | 5.00 | 0.00 | 12.39 | 32.00 | -1.49 | 0.91 | 0.343 |
| VC_0342 | 32.65 | 0.50 | 394.33 | 376.00 | 0.06 | 0.05 | 0.207 |
| VC_0341 | 1.00 | 0.00 | 26.78 | 17.00 | 0.58 | 0.17 | 1.000 |
| VC_0340 | 4.23 | 0.72 | 19.38 | 37.00 | -1.05 | 0.65 | 0.559 |
| VC_0339 | 1.00 | 0.00 | 95.57 | 15.00 | 2.65 | 0.02 | 1.000 |
| VC_0338 | 37.95 | 0.22 | 1036.45 | 897.00 | 0.21 | 0.03 | 0.545 |

| | | | | | | | |
|---|---|---|---|---|---|---|---|
| VC_0337 | 23.00 | 0.00 | 409.18 | 465.00 | -0.19 | 0.05 | 0.277 |
| VC_0336 | 1.00 | 0.00 | 115.79 | 42.00 | 1.45 | 0.04 | 1.000 |
| VC_0335 | 25.96 | 0.20 | 232.68 | 474.00 | -1.03 | 0.15 | 0.623 |
| VC_0334 | 29.17 | 0.74 | 647.18 | 656.00 | -0.02 | 0.04 | 0.321 |
| VC_0333 | 15.94 | 0.24 | 437.81 | 242.00 | 0.85 | 0.02 | 0.232 |
| VC_0332 | 2.68 | 0.47 | 58.66 | 23.00 | 1.32 | 0.06 | 0.391 |
| VC_0331 | 20.98 | 0.14 | 691.36 | 787.00 | -0.19 | 0.04 | 0.928 |
| VC_0330 | 16.59 | 0.49 | 168.54 | 280.00 | -0.74 | 0.11 | 0.543 |
| VC_0329 | 1.99 | 0.10 | 5.03 | 8.00 | -0.95 | 1.12 | 1.000 |
| VC_0328 | 0.00 | 0.00 | 1.00 | 1.00 | #VALEUR! | NaN | NaN |
| VC_0327 | 0.00 | 0.00 | 1.00 | 1.00 | #VALEUR! | NaN | NaN |
| VC_0326 | 0.00 | 0.00 | 1.00 | 1.00 | #VALEUR! | NaN | NaN |
| VC_0325 | 1.00 | 0.00 | 6.42 | 2.00 | 1.32 | 0.26 | 1.000 |
| VC_0324 | 0.00 | 0.00 | 1.00 | 1.00 | #VALEUR! | NaN | NaN |
| VC_0323 | 5.22 | 0.72 | 2.25 | 12.00 | -2.87 | 3.82 | 0.139 |
| VC_0322 | 0.74 | 0.44 | 1.38 | 1.00 | 0.33 | 0.26 | 1.000 |
| VC_0321 | 50.72 | 0.79 | 968.11 | 729.00 | 0.41 | 0.02 | 0.722 |
| VC_0320 | 9.00 | 0.00 | 21.68 | 68.00 | -1.73 | 0.82 | 0.045 |
| VC_0319 | 4.43 | 0.64 | 43.10 | 8.00 | 2.40 | 0.03 | 0.293 |
| VC_0318 | 0.54 | 0.50 | 1.35 | 1.00 | 0.42 | 0.28 | 1.000 |
| VC_0317 | 10.26 | 0.75 | 89.68 | 81.00 | 0.13 | 0.10 | 0.913 |
| VC_0316 | 0.59 | 0.49 | 1.32 | 1.00 | 0.33 | 0.27 | 1.000 |
| VC_0315 | 0.99 | 0.10 | 6.76 | 1.00 | 2.44 | 0.12 | 1.000 |
| VC_0314 | 6.61 | 0.49 | 31.70 | 23.00 | 0.41 | 0.15 | 0.710 |
| VC_0313 | 2.00 | 0.00 | 20.36 | 71.00 | -1.87 | 0.76 | 0.403 |
| VC_0312 | 30.57 | 0.57 | 395.09 | 576.00 | -0.55 | 0.08 | 0.320 |
| VC_0311 | 12.22 | 0.68 | 291.31 | 353.00 | -0.28 | 0.08 | 0.862 |
| VC_0309 | 14.91 | 0.89 | 322.06 | 196.00 | 0.71 | 0.03 | 0.003 |
| VC_0308 | 30.66 | 0.48 | 562.87 | 459.00 | 0.29 | 0.04 | 0.903 |
| VC_0307 | 4.79 | 0.43 | 8.70 | 9.00 | -0.23 | 0.45 | 0.800 |
| VC_0306 | 12.00 | 0.00 | 337.94 | 86.00 | 1.97 | 0.02 | 0.169 |
| VC_0305 | 35.98 | 0.14 | 793.13 | 362.00 | 1.13 | 0.02 | 0.072 |
| VC_0304 | 41.10 | 0.80 | 679.97 | 642.00 | 0.08 | 0.03 | 0.853 |
| VC_0303 | 94.96 | 0.92 | 2385.14 | 1957.00 | 0.28 | 0.02 | 0.397 |
| VC_0302 | 30.43 | 0.66 | 397.11 | 706.00 | -0.83 | 0.08 | 0.257 |
| VC_0301 | 6.00 | 0.00 | 53.43 | 229.00 | -2.12 | 0.58 | 0.273 |
| VC_0300 | 33.69 | 0.46 | 838.11 | 797.00 | 0.07 | 0.03 | 0.209 |
| VC_0299 | 12.88 | 0.33 | 380.96 | 144.00 | 1.40 | 0.02 | 0.315 |
| VC_0298 | 58.32 | 0.82 | 1538.20 | 1921.00 | -0.32 | 0.04 | 0.599 |
| VC_0297 | 10.92 | 0.27 | 242.94 | 213.00 | 0.18 | 0.06 | 0.587 |

| | | | | | | | |
|---|---|---|---|---|---|---|---|
| VC_0296 | 3.96 | 0.20 | 18.60 | 5.00 | 1.82 | 0.07 | 0.321 |
| VC_0295 | 0.00 | 0.00 | 1.00 | 1.00 | #VALEUR! | NaN | NaN |
| VC_0294 | 2.00 | 0.00 | 30.83 | 45.00 | -0.60 | 0.31 | 0.623 |
| VC_0293 | 18.00 | 0.00 | 385.26 | 249.00 | 0.63 | 0.03 | 0.042 |
| VC_0292 | 1.92 | 0.27 | 6.91 | 2.00 | 1.40 | 0.29 | 0.497 |
| VC_0291 | 16.80 | 0.84 | 158.87 | 235.00 | -0.57 | 0.11 | 0.584 |
| VC_0290 | 6.55 | 0.56 | 78.28 | 20.00 | 1.95 | 0.03 | 0.502 |
| VC_0289 | 17.00 | 0.00 | 574.29 | 304.00 | 0.91 | 0.02 | 0.553 |
| VC_0288 | 33.92 | 0.27 | 656.84 | 823.00 | -0.33 | 0.05 | 0.888 |
| VC_0287 | 9.91 | 0.29 | 47.24 | 162.00 | -1.81 | 0.57 | 0.824 |
| VC_0286 | 28.00 | 0.00 | 470.31 | 402.00 | 0.22 | 0.04 | 0.425 |
| VC_0285 | 12.00 | 0.00 | 317.14 | 226.00 | 0.48 | 0.04 | 0.564 |
| VC_0284 | 65.00 | 0.00 | 1643.60 | 1231.00 | 0.42 | 0.02 | 0.122 |
| VC_0283 | 14.00 | 0.00 | 251.38 | 277.00 | -0.15 | 0.07 | 0.895 |
| VC_0282 | 23.89 | 0.31 | 426.84 | 770.00 | -0.85 | 0.08 | 0.805 |
| VC_0281 | 60.74 | 1.07 | 1589.47 | 1401.00 | 0.18 | 0.02 | 0.699 |
| VC_0280 | 31.76 | 0.47 | 610.88 | 694.00 | -0.19 | 0.04 | 0.893 |
| VC_0279 | 2.00 | 0.00 | 5.17 | 3.00 | 0.35 | 0.58 | 0.537 |
| VC_0278 | 43.35 | 0.66 | 1109.13 | 679.00 | 0.71 | 0.02 | 0.064 |
| VC_0277 | 5.00 | 0.00 | 44.36 | 18.00 | 1.27 | 0.06 | 0.532 |
| VC_0276 | 29.75 | 0.50 | 704.76 | 809.00 | -0.20 | 0.04 | 0.551 |
| VC_0275 | 21.65 | 0.58 | 434.98 | 465.00 | -0.10 | 0.05 | 0.901 |
| VC_0274 | 28.97 | 0.17 | 353.94 | 367.00 | -0.06 | 0.06 | 0.778 |
| VC_0273 | 6.57 | 0.50 | 146.86 | 45.00 | 1.70 | 0.03 | 0.567 |
| VC_0272 | 3.89 | 0.31 | 31.60 | 14.00 | 1.13 | 0.09 | 0.654 |
| VC_0271 | 29.98 | 0.14 | 635.02 | 1186.00 | -0.90 | 0.08 | 0.106 |
| VC_0270 | 48.18 | 0.72 | 833.98 | 993.00 | -0.25 | 0.04 | 0.094 |
| VC_0269 | 47.95 | 1.19 | 736.95 | 920.00 | -0.32 | 0.05 | 0.890 |
| VC_0268 | 18.74 | 0.44 | 466.45 | 591.00 | -0.34 | 0.06 | 0.720 |
| VC_0267 | 19.52 | 0.59 | 298.92 | 330.00 | -0.15 | 0.07 | 0.106 |
| VC_0266 | 13.84 | 0.37 | 407.54 | 339.00 | 0.26 | 0.04 | 0.204 |
| VC_0265 | 29.44 | 0.66 | 414.79 | 575.00 | -0.47 | 0.07 | 0.485 |
| VC_0264 | 24.82 | 0.39 | 450.71 | 424.00 | 0.08 | 0.05 | 0.722 |
| VC_0263 | 12.62 | 0.49 | 13.56 | 165.00 | -3.71 | 3.88 | 0.002 |
| VC_0262 | 38.44 | 1.02 | 463.65 | 495.00 | -0.10 | 0.05 | 0.476 |
| VC_0261 | 4.75 | 0.44 | 46.67 | 81.00 | -0.83 | 0.27 | 0.668 |
| VC_0260 | 68.92 | 0.80 | 588.34 | 1176.00 | -1.00 | 0.08 | 0.041 |
| VC_0259 | 35.80 | 1.21 | 167.13 | 193.00 | -0.22 | 0.10 | 0.829 |
| VC_0258 | 14.75 | 0.46 | 85.57 | 33.00 | 1.36 | 0.04 | 0.242 |
| VC_0257 | 44.05 | 1.14 | 308.03 | 272.00 | 0.18 | 0.05 | 0.774 |

| | | | | | | | |
|---|---|---|---|---|---|---|---|
| VC_0256 | 16.63 | 0.51 | 95.78 | 50.00 | 0.92 | 0.05 | 0.277 |
| VC_0255 | 5.45 | 0.61 | 29.31 | 52.00 | -0.87 | 0.30 | 0.504 |
| VC_0254 | 43.31 | 0.69 | 765.49 | 708.00 | 0.11 | 0.04 | 0.783 |
| VC_0253 | 4.00 | 0.00 | 58.70 | 32.00 | 0.85 | 0.07 | 0.993 |
| VC_0252 | 24.36 | 1.05 | 107.56 | 233.00 | -1.13 | 0.21 | 0.587 |
| VC_0251 | 109.87 | 1.54 | 1114.99 | 1260.00 | -0.18 | 0.04 | 0.451 |
| VC_0250 | 47.14 | 0.79 | 344.18 | 559.00 | -0.70 | 0.09 | 0.239 |
| VC_0249 | 50.16 | 1.40 | 248.55 | 220.00 | 0.17 | 0.06 | 0.578 |
| VC_0248 | 2.00 | 0.00 | 1.00 | 3.00 | -1.58 | 0.00 | 0.333 |
| VC_0247 | 8.56 | 0.57 | 3.16 | 23.00 | -3.30 | 6.07 | 0.034 |
| VC_0246 | 11.01 | 0.88 | 7.42 | 39.00 | -2.60 | 2.63 | 0.079 |
| VC_0245 | 26.62 | 1.33 | 106.68 | 53.00 | 0.99 | 0.05 | 0.062 |
| VC_0244 | 52.54 | 0.91 | 498.11 | 771.00 | -0.63 | 0.07 | 0.033 |
| VC_0243 | 57.04 | 0.84 | 655.38 | 602.00 | 0.12 | 0.03 | 0.726 |
| VC_0242 | 51.40 | 1.20 | 307.76 | 265.00 | 0.21 | 0.04 | 0.052 |
| VC_0241 | 64.87 | 0.80 | 700.57 | 1152.00 | -0.72 | 0.06 | 0.004 |
| VC_0240 | 17.27 | 0.74 | 10.27 | 184.00 | -4.36 | 9.79 | 0.000 |
| VC_0239 | 40.50 | 0.52 | 179.74 | 418.00 | -1.22 | 0.16 | 0.000 |
| VC_0238 | 7.31 | 0.71 | 14.65 | 70.00 | -2.35 | 1.43 | 0.494 |
| VC_0237 | 12.00 | 0.00 | 3.32 | 105.00 | -5.46 | 29.41 | 0.000 |
| VC_0236 | 19.77 | 0.49 | 37.43 | 115.00 | -1.66 | 0.58 | 0.004 |
| VC_0235 | 36.49 | 0.59 | 654.48 | 488.00 | 0.42 | 0.03 | 0.353 |
| VC_0234 | 41.44 | 0.73 | 1284.16 | 647.00 | 0.99 | 0.01 | 0.106 |
| VC_0233 | 1.00 | 0.00 | 1.00 | 6.00 | -2.58 | 0.00 | 1.000 |
| VC_0232 | 15.00 | 0.00 | 322.24 | 204.00 | 0.65 | 0.04 | 0.819 |
| VC_0231 | 63.04 | 0.96 | 706.30 | 867.00 | -0.30 | 0.05 | 0.081 |
| VC_0230 | 24.39 | 0.58 | 434.38 | 261.00 | 0.73 | 0.03 | 0.360 |
| VC_0229 | 25.45 | 0.69 | 317.40 | 275.00 | 0.20 | 0.04 | 0.840 |
| VC_0228 | 15.28 | 0.68 | 70.87 | 35.00 | 0.99 | 0.07 | 0.316 |
| VC_0227 | 3.64 | 0.48 | 15.79 | 2.00 | 2.86 | 0.04 | 0.308 |
| VC_0226 | 2.00 | 0.00 | 36.42 | 1.00 | 5.15 | 0.00 | 0.333 |
| VC_0225 | 16.00 | 0.00 | 16.13 | 187.00 | -3.65 | 4.06 | 0.000 |
| VC_0224 | 17.89 | 0.31 | 123.57 | 279.00 | -1.19 | 0.21 | 0.016 |
| VC_0223 | 19.00 | 0.00 | 76.78 | 265.00 | -1.81 | 0.42 | 0.009 |
| VC_0222 | 1.00 | 0.00 | 1.00 | 45.00 | -5.49 | 0.00 | 1.000 |
| VC_0221 | 17.90 | 0.85 | 393.74 | 636.00 | -0.70 | 0.08 | 0.811 |
| VC_0220 | 15.78 | 0.46 | 311.21 | 85.00 | 1.87 | 0.02 | 0.073 |
| VC_0219 | 0.86 | 0.35 | 2.21 | 1.00 | 0.79 | 0.31 | 1.000 |
| VC_0218 | 0.00 | 0.00 | 1.00 | 1.00 | #VALEUR! | NaN | NaN |
| VC_0217 | 17.00 | 0.00 | 353.96 | 544.00 | -0.62 | 0.08 | 0.365 |

| | | | | | | | |
|---|---|---|---|---|---|---|---|
| VC_0216 | 46.87 | 0.84 | 788.54 | 793.00 | -0.01 | 0.04 | 0.864 |
| VC_0215 | 1.00 | 0.00 | 7.11 | 7.00 | -0.33 | 0.98 | 1.000 |
| VC_0214 | 11.20 | 0.77 | 115.21 | 93.00 | 0.29 | 0.08 | 0.145 |
| VC_0213 | 2.00 | 0.00 | 1.00 | 68.00 | -6.09 | 0.00 | 0.333 |
| VC_0212 | 18.82 | 0.39 | 173.60 | 321.00 | -0.90 | 0.17 | 0.451 |
| VC_0211 | 18.87 | 0.34 | 492.79 | 234.00 | 1.07 | 0.02 | 0.246 |
| VC_0210 | 15.87 | 0.34 | 216.16 | 292.00 | -0.44 | 0.08 | 0.501 |
| VC_0209 | 10.28 | 0.73 | 148.57 | 76.00 | 0.96 | 0.04 | 0.850 |
| VC_0208 | 16.96 | 0.20 | 191.25 | 264.00 | -0.47 | 0.09 | 0.612 |
| VC_0207 | 28.68 | 0.51 | 560.79 | 511.00 | 0.13 | 0.04 | 0.846 |
| VC_0206 | 18.49 | 0.54 | 489.05 | 559.00 | -0.20 | 0.05 | 0.706 |
| VC_0205 | 4.00 | 0.00 | 17.56 | 50.00 | -1.64 | 1.26 | 0.180 |
| VC_0204 | 14.00 | 0.00 | 330.91 | 195.00 | 0.76 | 0.03 | 0.145 |
| VC_0203 | 36.78 | 0.42 | 622.21 | 756.00 | -0.28 | 0.06 | 0.772 |
| VC_0202 | 19.92 | 0.27 | 533.09 | 240.00 | 1.15 | 0.02 | 0.403 |
| VC_0201 | 13.82 | 0.39 | 173.71 | 157.00 | 0.14 | 0.07 | 0.642 |
| VC_0200 | 74.13 | 0.80 | 1929.26 | 1448.00 | 0.41 | 0.02 | 0.157 |
| VC_0199 | 41.60 | 0.49 | 1396.86 | 1441.00 | -0.05 | 0.03 | 0.919 |
| VC_0198 | 14.00 | 0.00 | 211.10 | 261.00 | -0.31 | 0.09 | 0.328 |
| VC_0197 | 5.57 | 0.50 | 92.34 | 30.00 | 1.60 | 0.04 | 0.649 |
| VC_0196 | 42.36 | 0.66 | 871.27 | 938.00 | -0.11 | 0.04 | 0.924 |
| VC_0195 | 23.46 | 0.66 | 615.64 | 550.00 | 0.16 | 0.04 | 0.515 |
| VC_0194 | 30.98 | 0.85 | 459.86 | 685.00 | -0.58 | 0.08 | 0.122 |
| VC_0193 | 0.00 | 0.00 | 1.00 | 1.00 | #VALEUR! | NaN | NaN |
| VC_0192 | 23.65 | 0.48 | 254.38 | 501.00 | -0.98 | 0.12 | 0.177 |
| VC_0191 | 24.82 | 0.41 | 666.71 | 421.00 | 0.66 | 0.03 | 0.797 |
| VC_0190 | 46.41 | 1.10 | 472.20 | 493.00 | -0.07 | 0.05 | 0.763 |
| VC_0189 | 6.00 | 0.00 | 30.57 | 33.00 | -0.17 | 0.23 | 0.406 |
| VC_0188 | 45.18 | 0.72 | 1006.45 | 1432.00 | -0.51 | 0.05 | 0.307 |
| VC_0187 | 24.33 | 0.65 | 339.29 | 582.00 | -0.78 | 0.09 | 0.834 |
| VC_0186 | 29.24 | 0.99 | 529.85 | 303.00 | 0.80 | 0.03 | 0.441 |
| VC_0185 | 52.61 | 1.06 | 499.07 | 529.00 | -0.09 | 0.06 | 0.603 |
| VC_0184 | 59.22 | 1.09 | 622.66 | 531.00 | 0.23 | 0.03 | 0.483 |
| VC_0183 | 72.27 | 1.31 | 635.30 | 812.00 | -0.36 | 0.05 | 0.862 |
| VC_0182 | 23.33 | 0.67 | 100.55 | 271.00 | -1.45 | 0.30 | 0.275 |
| VC_0181 | 18.28 | 0.70 | 226.66 | 443.00 | -0.97 | 0.13 | 0.699 |
| VC_0180 | 98.55 | 1.07 | 1933.89 | 2195.00 | -0.18 | 0.03 | 0.240 |
| VC_0179 | 62.34 | 0.64 | 1050.26 | 1098.00 | -0.07 | 0.03 | 0.896 |
| VC_0178 | 58.68 | 0.47 | 674.62 | 754.00 | -0.16 | 0.05 | 0.419 |
| VC_0177 | 18.27 | 0.75 | 116.84 | 204.00 | -0.82 | 0.16 | 0.157 |

| | | | | | | | |
|---|---|---|---|---|---|---|---|
| VC_0176 | 14.61 | 0.49 | 206.07 | 165.00 | 0.32 | 0.05 | 0.163 |
| VC_0175 | 71.78 | 0.92 | 678.34 | 869.00 | -0.36 | 0.05 | 0.485 |
| VC_0174 | 15.52 | 0.52 | 249.04 | 193.00 | 0.36 | 0.05 | 0.551 |
| VC_0173 | 20.94 | 0.24 | 172.32 | 354.00 | -1.05 | 0.16 | 0.017 |
| VC_0172 | 26.60 | 0.49 | 574.18 | 590.00 | -0.04 | 0.04 | 0.862 |
| VC_0171 | 45.64 | 0.48 | 655.73 | 630.00 | 0.06 | 0.04 | 0.900 |
| VC_0170 | 43.56 | 0.54 | 868.80 | 1195.00 | -0.46 | 0.05 | 0.448 |
| VC_0169 | 2.62 | 0.53 | 4.21 | 5.00 | -0.66 | 1.06 | 0.964 |
| VC_0168 | 15.61 | 0.57 | 217.56 | 367.00 | -0.76 | 0.12 | 0.436 |
| VC_0167 | 3.99 | 0.10 | 14.48 | 7.00 | 0.95 | 0.15 | 0.891 |
| VC_0166 | 18.62 | 0.49 | 353.64 | 377.00 | -0.10 | 0.05 | 0.451 |
| VC_0165 | 37.61 | 0.53 | 727.18 | 598.00 | 0.28 | 0.03 | 0.442 |
| VC_0164 | 81.56 | 1.30 | 1911.38 | 1311.00 | 0.54 | 0.02 | 0.122 |
| VC_0163 | 3.00 | 0.00 | 6.77 | 33.00 | -2.61 | 4.36 | 0.254 |
| VC_0162 | 46.24 | 0.71 | 783.62 | 793.00 | -0.02 | 0.04 | 0.257 |
| VC_0161 | 25.62 | 0.49 | 433.43 | 399.00 | 0.12 | 0.05 | 0.558 |
| VC_0160 | 6.00 | 0.00 | 29.36 | 22.00 | 0.35 | 0.17 | 0.827 |
| VC_0159 | 12.99 | 0.10 | 145.11 | 135.00 | 0.10 | 0.07 | 0.314 |
| VC_0158 | 2.00 | 0.00 | 1.00 | 10.00 | -3.32 | 0.00 | 0.333 |
| VC_0157 | 57.61 | 0.49 | 1174.93 | 1725.00 | -0.56 | 0.05 | 0.175 |
| VC_0156 | 99.96 | 0.95 | 1958.40 | 1873.00 | 0.06 | 0.02 | 0.654 |
| VC_0154 | 34.65 | 0.48 | 939.28 | 700.00 | 0.42 | 0.03 | 0.524 |
| VC_0153 | 12.00 | 0.00 | 120.05 | 281.00 | -1.24 | 0.22 | 0.020 |
| VC_0152 | 10.50 | 0.58 | 241.52 | 171.00 | 0.49 | 0.05 | 0.621 |
| VC_0151 | 44.68 | 0.47 | 599.78 | 617.00 | -0.04 | 0.04 | 0.124 |
| VC_0150 | 0.00 | 0.00 | 1.00 | 1.00 | #VALEUR! | NaN | NaN |
| VC_0149 | 30.97 | 0.17 | 525.15 | 594.00 | -0.18 | 0.05 | 0.480 |
| VC_0148 | 17.95 | 0.22 | 270.15 | 530.00 | -0.98 | 0.13 | 0.266 |
| VC_0147 | 1.00 | 0.00 | 1.00 | 6.00 | -2.58 | 0.00 | 1.000 |
| VC_0146 | 15.75 | 0.46 | 144.51 | 163.00 | -0.18 | 0.09 | 0.891 |
| VC_0145 | 5.83 | 0.38 | 114.06 | 178.00 | -0.65 | 0.15 | 0.886 |
| VC_0144 | 22.96 | 0.20 | 403.48 | 279.00 | 0.53 | 0.04 | 0.100 |
| VC_0143 | 0.93 | 0.26 | 2.96 | 1.00 | 1.14 | 0.29 | 1.000 |
| VC_0142 | 12.31 | 0.66 | 152.27 | 133.00 | 0.19 | 0.07 | 0.796 |
| VC_0141 | 3.68 | 0.47 | 20.52 | 33.00 | -0.74 | 0.32 | 0.770 |
| VC_0140 | 10.00 | 0.00 | 466.21 | 314.00 | 0.57 | 0.03 | 0.821 |
| VC_0139 | 12.99 | 0.10 | 118.40 | 77.00 | 0.61 | 0.07 | 0.064 |
| VC_0138 | 3.00 | 0.00 | 52.71 | 38.00 | 0.44 | 0.10 | 0.997 |
| VC_0137 | 39.00 | 0.00 | 1092.46 | 799.00 | 0.45 | 0.02 | 0.849 |
| VC_0136 | 24.57 | 0.50 | 568.86 | 648.00 | -0.19 | 0.05 | 0.933 |

| | | | | | | | |
|---|---|---|---|---|---|---|---|
| VC_0135 | 38.28 | 0.70 | 906.58 | 804.00 | 0.17 | 0.03 | 0.406 |
| VC_0134 | 20.84 | 0.37 | 392.79 | 553.00 | -0.50 | 0.08 | 0.260 |
| VC_0133 | 3.00 | 0.00 | 19.37 | 27.00 | -0.56 | 0.42 | 0.793 |
| VC_0132 | 41.99 | 0.10 | 399.81 | 1290.00 | -1.69 | 0.18 | 0.000 |
| VC_0131 | 13.00 | 0.00 | 135.22 | 142.00 | -0.08 | 0.09 | 0.578 |
| VC_0130 | 51.36 | 0.99 | 1203.06 | 966.00 | 0.32 | 0.02 | 0.876 |
| VC_0129 | 22.00 | 0.00 | 320.87 | 467.00 | -0.55 | 0.09 | 0.620 |
| VC_0128 | 24.18 | 0.74 | 165.05 | 246.00 | -0.58 | 0.12 | 0.725 |
| VC_0127 | 18.56 | 0.59 | 326.02 | 291.00 | 0.16 | 0.05 | 0.878 |
| VC_0126 | 3.00 | 0.00 | 7.01 | 26.00 | -2.18 | 2.84 | 0.195 |
| VC_0125 | 46.62 | 0.49 | 650.62 | 605.00 | 0.10 | 0.04 | 0.547 |
| VC_0124 | 8.00 | 0.00 | 369.73 | 403.00 | -0.13 | 0.05 | 0.944 |
| VC_0123 | 3.86 | 0.35 | 76.04 | 189.00 | -1.33 | 0.31 | 0.732 |
| VC_0122 | 62.21 | 1.09 | 600.99 | 717.00 | -0.26 | 0.05 | 0.512 |
| VC_0121 | 5.93 | 0.26 | 44.72 | 29.00 | 0.59 | 0.10 | 0.173 |
| VC_0120 | 3.00 | 0.00 | 18.62 | 13.00 | 0.43 | 0.20 | 0.790 |
| VC_0119 | 6.79 | 0.43 | 85.66 | 61.00 | 0.47 | 0.08 | 0.277 |
| VC_0118 | 21.60 | 0.57 | 154.87 | 322.00 | -1.06 | 0.16 | 0.139 |
| VC_0117 | 28.52 | 0.73 | 692.00 | 542.00 | 0.35 | 0.03 | 0.752 |
| VC_0116 | 49.05 | 0.85 | 1496.86 | 793.00 | 0.92 | 0.02 | 0.051 |
| VC_0115 | 14.99 | 0.10 | 494.55 | 233.00 | 1.08 | 0.02 | 0.532 |
| VC_0114 | 13.35 | 0.63 | 245.76 | 212.00 | 0.21 | 0.06 | 0.898 |
| VC_0113 | 26.79 | 0.52 | 521.45 | 768.00 | -0.56 | 0.07 | 0.700 |
| VC_0112 | 29.58 | 0.54 | 803.71 | 1052.00 | -0.39 | 0.04 | 0.772 |
| VC_0111 | 1.00 | 0.00 | 1.00 | 28.00 | -4.81 | 0.00 | 1.000 |
| VC_0110 | 4.21 | 0.69 | 15.33 | 94.00 | -2.72 | 1.89 | 0.320 |
| VC_0109 | 1.71 | 0.46 | 5.25 | 1.00 | 2.00 | 0.18 | 0.527 |
| VC_0108 | 10.39 | 0.99 | 95.98 | 7.00 | 3.76 | 0.01 | 0.001 |
| VC_0107 | 3.00 | 0.00 | 1.39 | 5.00 | -2.04 | 1.23 | 0.355 |
| VC_0106 | 14.95 | 0.22 | 177.87 | 88.00 | 1.00 | 0.04 | 0.039 |
| VC_0105 | 9.49 | 0.89 | 42.68 | 5.00 | 3.06 | 0.02 | 0.015 |
| VC_0104 | 2.00 | 0.00 | 150.53 | 20.00 | 2.90 | 0.01 | 0.333 |
| VC_0103 | 23.00 | 0.00 | 390.92 | 423.00 | -0.12 | 0.06 | 0.290 |
| VC_0102 | 11.99 | 0.10 | 186.16 | 139.00 | 0.41 | 0.05 | 0.747 |
| VC_0101 | 4.00 | 0.00 | 29.56 | 33.00 | -0.21 | 0.23 | 0.947 |
| VC_0100 | 13.69 | 0.46 | 338.96 | 253.00 | 0.42 | 0.05 | 0.443 |
| VC_0099 | 30.81 | 0.39 | 532.85 | 367.00 | 0.54 | 0.03 | 0.565 |
| VC_0098 | 42.69 | 1.09 | 774.68 | 771.00 | 0.01 | 0.04 | 0.879 |
| VC_0097 | 17.23 | 0.69 | 257.06 | 141.00 | 0.86 | 0.04 | 0.721 |
| VC_0096 | 6.70 | 0.46 | 99.27 | 55.00 | 0.84 | 0.05 | 0.775 |

| | | | | | | | |
|---|---|---|---|---|---|---|---|
| VC_0095 | 25.99 | 0.10 | 252.32 | 298.00 | -0.25 | 0.07 | 0.791 |
| VC_0094 | 16.02 | 0.72 | 115.20 | 32.00 | 1.83 | 0.03 | 0.059 |
| VC_0093 | 59.06 | 0.78 | 1167.99 | 1296.00 | -0.15 | 0.03 | 0.665 |
| VC_0092 | 2.00 | 0.00 | 6.04 | 7.00 | -0.57 | 0.91 | 0.943 |
| VC_0091 | 17.62 | 0.49 | 217.14 | 203.00 | 0.09 | 0.06 | 0.719 |
| VC_0090 | 40.38 | 0.68 | 720.03 | 719.00 | 0.00 | 0.04 | 0.879 |
| VC_0089 | 31.19 | 0.85 | 484.99 | 584.00 | -0.27 | 0.06 | 0.858 |
| VC_0088 | 21.92 | 0.27 | 266.07 | 306.00 | -0.21 | 0.07 | 0.919 |
| VC_0087 | 7.66 | 0.48 | 85.74 | 42.00 | 1.01 | 0.05 | 0.447 |
| VC_0086 | 7.00 | 0.00 | 131.76 | 87.00 | 0.59 | 0.06 | 0.596 |
| VC_0085 | 9.47 | 1.18 | 27.73 | 12.00 | 1.16 | 0.09 | 0.009 |
| VC_0084 | 4.91 | 0.29 | 22.67 | 3.00 | 2.87 | 0.03 | 0.144 |
| VC_0083 | 6.99 | 1.12 | 13.78 | 11.00 | 0.22 | 0.24 | 0.076 |
| VC_0082 | 35.60 | 0.49 | 641.50 | 662.00 | -0.05 | 0.04 | 0.134 |
| VC_0081 | 17.98 | 0.14 | 136.72 | 160.00 | -0.24 | 0.10 | 0.718 |
| VC_0080 | 29.10 | 0.82 | 583.62 | 710.00 | -0.29 | 0.05 | 0.469 |
| VC_0079 | 14.64 | 0.48 | 516.14 | 305.00 | 0.76 | 0.02 | 0.260 |
| VC_0078 | 17.91 | 0.87 | 403.39 | 400.00 | 0.01 | 0.05 | 0.881 |
| VC_0077 | 5.00 | 0.00 | 185.65 | 67.00 | 1.46 | 0.03 | 0.767 |
| VC_0076 | 12.87 | 0.37 | 307.37 | 241.00 | 0.35 | 0.04 | 0.089 |
| VC_0075 | 30.10 | 0.75 | 321.25 | 381.00 | -0.25 | 0.07 | 0.680 |
| VC_0074 | 6.50 | 0.52 | 54.36 | 24.00 | 1.16 | 0.06 | 0.856 |
| VC_0073 | 16.68 | 0.47 | 229.71 | 260.00 | -0.19 | 0.08 | 0.843 |
| VC_0072 | 69.09 | 0.84 | 1000.03 | 1665.00 | -0.74 | 0.06 | 0.129 |
| VC_0071 | 16.00 | 0.78 | 223.97 | 297.00 | -0.41 | 0.10 | 0.804 |
| VC_0070 | 10.65 | 0.48 | 316.15 | 160.00 | 0.98 | 0.03 | 0.329 |
| VC_0069 | 43.27 | 0.57 | 843.77 | 838.00 | 0.01 | 0.03 | 0.918 |
| VC_0068 | 34.70 | 0.46 | 500.70 | 559.00 | -0.16 | 0.05 | 0.798 |
| VC_0067 | 43.00 | 0.00 | 1168.05 | 1034.00 | 0.17 | 0.03 | 0.772 |
| VC_0066 | 30.72 | 0.45 | 587.13 | 367.00 | 0.68 | 0.03 | 0.342 |
| VC_0065 | 15.85 | 0.77 | 107.91 | 80.00 | 0.42 | 0.07 | 0.356 |
| VC_0064 | 7.00 | 0.00 | 127.32 | 54.00 | 1.22 | 0.05 | 0.884 |
| VC_0063 | 28.11 | 0.75 | 311.99 | 436.00 | -0.49 | 0.08 | 0.690 |
| VC_0062 | 48.72 | 0.94 | 697.97 | 735.00 | -0.08 | 0.04 | 0.906 |
| VC_0061 | 79.44 | 1.00 | 1478.52 | 1087.00 | 0.44 | 0.02 | 0.065 |
| VC_0060 | 11.98 | 0.14 | 69.40 | 168.00 | -1.30 | 0.30 | 0.263 |
| VC_0059 | 4.00 | 0.00 | 11.55 | 14.00 | -0.45 | 0.68 | 0.666 |
| VC_0058 | 21.61 | 0.49 | 439.66 | 522.00 | -0.25 | 0.06 | 0.878 |
| VC_0057 | 6.70 | 0.46 | 42.59 | 89.00 | -1.09 | 0.31 | 0.737 |
| VC_0056 | 32.57 | 0.52 | 667.54 | 530.00 | 0.33 | 0.03 | 0.149 |

| | | | | | | | |
|---|---|---|---|---|---|---|---|
| VC_0055 | 3.68 | 0.47 | 5.32 | 9.00 | -1.17 | 1.72 | 0.644 |
| VC_0054 | 16.97 | 0.17 | 531.20 | 517.00 | 0.04 | 0.05 | 0.900 |
| VC_0053 | 3.66 | 0.48 | 30.45 | 70.00 | -1.24 | 0.38 | 0.403 |
| VC_0052 | 13.98 | 0.14 | 258.39 | 197.00 | 0.39 | 0.05 | 0.708 |
| VC_0051 | 33.95 | 0.22 | 585.20 | 740.00 | -0.34 | 0.05 | 0.316 |
| VC_0050 | 15.98 | 0.14 | 874.70 | 371.00 | 1.24 | 0.01 | 0.314 |
| VC_0049 | 8.00 | 0.00 | 87.84 | 203.00 | -1.23 | 0.31 | 0.322 |
| VC_0048 | 35.98 | 0.14 | 545.38 | 463.00 | 0.23 | 0.03 | 0.859 |
| VC_0047 | 34.53 | 0.66 | 766.99 | 810.00 | -0.08 | 0.04 | 0.880 |
| VC_0046 | 14.66 | 0.48 | 294.65 | 324.00 | -0.14 | 0.06 | 0.823 |
| VC_0045 | 1.00 | 0.00 | 8.53 | 1.00 | 2.82 | 0.10 | 1.000 |
| VC_0044 | 27.36 | 0.70 | 567.13 | 348.00 | 0.70 | 0.03 | 0.653 |
| VC_0043 | 2.53 | 0.50 | 1.45 | 8.00 | -2.73 | 2.12 | 0.402 |
| VC_0042 | 40.23 | 0.71 | 622.87 | 800.00 | -0.36 | 0.05 | 0.490 |
| VC_0041 | 15.98 | 0.14 | 221.80 | 307.00 | -0.48 | 0.10 | 0.855 |
| VC_0040 | 26.94 | 0.93 | 432.26 | 605.00 | -0.49 | 0.06 | 0.279 |
| VC_0039 | 21.70 | 0.92 | 289.52 | 382.00 | -0.40 | 0.08 | 0.203 |
| VC_0038 | 1.87 | 0.34 | 5.45 | 17.00 | -2.02 | 2.84 | 1.000 |
| VC_0037 | 11.97 | 0.17 | 215.00 | 59.00 | 1.86 | 0.02 | 0.017 |
| VC_0036 | 38.57 | 0.59 | 720.26 | 658.00 | 0.13 | 0.04 | 0.312 |
| VC_0035 | 30.59 | 0.55 | 637.27 | 495.00 | 0.36 | 0.03 | 0.555 |
| VC_0034 | 23.60 | 0.51 | 129.14 | 370.00 | -1.53 | 0.27 | 0.034 |
| VC_0033 | 28.65 | 0.48 | 937.95 | 563.00 | 0.74 | 0.02 | 0.156 |
| VC_0032 | 30.00 | 0.82 | 495.97 | 391.00 | 0.34 | 0.04 | 0.845 |
| VC_0031 | 26.00 | 0.00 | 1041.14 | 883.00 | 0.24 | 0.03 | 0.201 |
| VC_0030 | 7.95 | 0.22 | 73.53 | 107.00 | -0.56 | 0.18 | 0.634 |
| VC_0029 | 25.99 | 0.10 | 376.71 | 466.00 | -0.31 | 0.07 | 0.887 |
| VC_0028 | 42.98 | 0.14 | 1269.01 | 1539.00 | -0.28 | 0.04 | 0.875 |
| VC_0027 | 30.75 | 0.46 | 591.78 | 455.00 | 0.38 | 0.03 | 0.877 |
| VC_0026 | 24.77 | 0.45 | 525.01 | 599.00 | -0.19 | 0.05 | 0.477 |
| VC_0025 | 7.58 | 0.50 | 38.93 | 349.00 | -3.21 | 1.63 | 0.011 |
| VC_0024 | 1.00 | 0.00 | 7.61 | 1.00 | 2.67 | 0.10 | 1.000 |
| VC_0023 | 11.00 | 0.00 | 269.54 | 227.00 | 0.24 | 0.06 | 0.529 |
| VC_0022 | 21.92 | 0.27 | 396.78 | 261.00 | 0.60 | 0.03 | 0.640 |
| VC_0021 | 3.64 | 0.48 | 14.38 | 39.00 | -1.56 | 0.95 | 0.272 |
| VC_0020 | 0.00 | 0.00 | 1.00 | 1.00 | #VALEUR! | NaN | NaN |
| VC_0019 | 32.30 | 0.69 | 567.44 | 491.00 | 0.21 | 0.04 | 0.393 |
| VC_0018 | 9.88 | 0.33 | 170.61 | 90.00 | 0.91 | 0.04 | 0.572 |
| VC_0017 | 1.00 | 0.00 | 14.58 | 1.00 | 3.75 | 0.02 | 1.000 |
| VC_0016 | 17.95 | 0.22 | 579.91 | 340.00 | 0.77 | 0.02 | 0.242 |

| | | | | | | | |
|---|---|---|---|---|---|---|---|
| VC_0015 | 2.00 | 0.00 | 23.57 | 22.00 | 0.04 | 0.20 | 1.000 |
| VC_0014 | 31.57 | 0.59 | 303.22 | 285.00 | 0.08 | 0.06 | 0.880 |
| VC_0013 | 0.92 | 0.27 | 2.97 | 1.00 | 1.32 | 0.23 | 1.000 |
| VC_0012 | 0.00 | 0.00 | 1.00 | 1.00 | #VALEUR! | NaN | NaN |
| VC_0011 | 7.00 | 0.00 | 45.05 | 47.00 | -0.10 | 0.18 | 0.779 |
| VC_0010 | 23.98 | 0.14 | 325.14 | 296.00 | 0.13 | 0.06 | 0.589 |
| VC_0009 | 16.57 | 0.50 | 217.77 | 204.00 | 0.09 | 0.06 | 0.361 |
| VC_0008 | 12.21 | 0.69 | 270.90 | 180.00 | 0.58 | 0.04 | 0.474 |
| VC_0007 | 0.00 | 0.00 | 1.00 | 1.00 | #VALEUR! | NaN | NaN |
| VC_0006 | 1.00 | 0.00 | 1.00 | 3.00 | -1.58 | 0.00 | 1.000 |
| VC_0005 | 8.27 | 0.65 | 55.55 | 54.00 | 0.02 | 0.13 | 0.389 |
| VC_0004 | 3.00 | 0.00 | 1.35 | 17.00 | -3.84 | 4.05 | 0.162 |
| VC_0003 | 31.03 | 1.22 | 217.68 | 203.00 | 0.09 | 0.06 | 0.695 |
| VC_0002 | 7.69 | 1.09 | 41.18 | 28.00 | 0.52 | 0.11 | 0.343 |
| VC_0001 | 3.00 | 0.00 | 5.87 | 3.00 | 0.70 | 0.34 | 0.400 |
