# Supplementary material for: A Vibrio cholerae BolA-Like Protein Is Required for Proper Cell Shape and Cell Envelope Integrity
Source: mBio. 2019 Jul 9;10(4):e00790-19. doi: 10.1128/mBio.00790-19 (PMC6747721; doi:10.1128/mBio.00790-19)

Table S2. Total proteins identified by mass spectrometry

Unique peptides	Total peptides	reference	Gene Symbol	MW(kDa)	Predicted function
42	73	sp|Q9KV29|RPOC_VIBCH	rpoC	154.93	DNA-directed RNA polymerase subunit beta
40	62	sp|Q9KV30|RPOB_VIBCH	rpoB	149.36	DNA-directed RNA polymerase subunit beta
33	256	sp|Q9KPM5|EFG2_VIBCH	fusA2	76.43	elongation factor
29	105	sp|O34241|DNAK_VIBCH	dnaK	68.72	chaperone
28	65	tr|Q9KQT3|Q9KQT3_VIBCH	VC_1915	61.01	30 s ribosomal protein s1
27	169	sp|Q9KV37|EFTU1_VIBCH	tufA	43.16	elongation factor
24	39	tr|Q9KPF4|Q9KPF4_VIBCH	VC_2414	98.95	acetyl coa metabolism pyruvate dehydrogenase E1 component
24	28	tr|Q9KPJ4|Q9KPJ4_VIBCH	VC_2373	168.83	glutamate synthase large subunit
23	44	sp|Q9KVX3|GYRB_VIBCH	gyrB	89.46	DNA gyrase subunit B
21	71	tr|Q9KQG5|Q9KQG5_VIBCH	VC_2033	96.17	Fe-ADH aldehyde-alcohol dehydrogenase
21	44	sp|Q9KT08|PTA_VIBCH	pta	76.84	phosphate acetyltransferase acetyl coa metabolism
21	37	sp|P22359|HTPG_VIBCH	htpG	72.15	chaperone protein heat shock
18	33	tr|Q9KV99|Q9KV99_VIBCH	VC_0251	91.77	biosynthesis o antigen Acyl protein synthase/acyl-CoA reductase RfbN
18	23	tr|Q9KUB8|Q9KUB8_VIBCH	VC_0604	93.53	bind 4Fe-4S cluster aconitate hydratase b
17	18	tr|Q9KQB3|Q9KQB3_VIBCH	VC_2087	105.25	2-oxoglutarate dehydrogenase, E1 component
16	29	tr|Q9KQS8|Q9KQS8_VIBCH	lon	87.93	protease stress response
16	28	sp|Q9KU76|PNP_VIBCH	pnp	76.54	Polyribonucleotide nucleotidyltransferase
16	21	tr|Q9KSJ8|Q9KSJ8_VIBCH	gyrA	98.96	DNA gyrase subunit A
16	19	tr|Q9KRZ1|Q9KRZ1_VIBCH	VC_1492	183.44	glutamate dehydrogenase
15	110	sp|Q9KNR7|CH601_VIBCH	groL1	57.12	chaperone protein
15	21	sp|Q9KPH4|SECA_VIBCH	secA	102.41	protein translocase
15	17	sp|Q9KTN2|PUR4_VIBCH	purL	141.33	purine biosynthetic pathway
14	16	tr|Q9KU31|Q9KU31_VIBCH	VC_0698	62.45	
13	29	tr|Q9KL05|Q9KL05_VIBCH	VC_A0945	43.78	maldotextrin binding protein ABC transporter
13	27	sp|Q9KU80|IF2_VIBCH	infB	98.64	tranlation initiation factor
13	19	tr|Q9KNJ4|Q9KNJ4_VIBCH	VC_2744	67.14	gtp binding protein TypA BipA
13	14	sp|Q9KUZ7|EFG1_VIBCH	fusA1	76.88	elongation factor
13	14	tr|Q9KSK0|Q9KSK0_VIBCH	VC_1256	85.80	dna replication oxydoreductase
13	13	sp|Q9KP73|SYV_VIBCH	valS	108.10	valine tRNA ligase
12	23	tr|Q9KQY1|Q9KQY1_VIBCH	VC_1866	87.73	phosphoenolpyruvate carboxykinase
12	18	sp|Q9KNK0|PCKA_VIBCH	pckA	59.81	
12	17	sp|Q9KSU0|SYD_VIBCH	aspS	65.73	aspartate t rna ligase
12	12	sp|Q9KQC6|SYR_VIBCH	argS	63.84	arginine t RNA ligase
11	18	tr|Q9KU81|Q9KU81_VIBCH	nusA	54.91	transcription termination
11	18	tr|Q9KLJ7|Q9KLJ7_VIBCH	VC_A0747	59.88	Anaerobic glycerol-3-phosphate dehydrogenase, subunit A
11	14	tr|Q9KNS5|Q9KNS5_VIBCH	VC_2656	66.36	frda Fumarate reductase flavoprotein subunit
11	13	sp|Q9KTA6|SYQ_VIBCH	glnS	64.05	glutamine trna ligase
11	13	tr|Q9KTD7|Q9KTD7_VIBCH	VC_0965	63.09	Phosphoenolpyruvate-protein phosphotransferase
11	12	tr|Q9KQT0|Q9KQT0_VIBCH	VC_1918	68.45	Peptidylprolyl isomerase
11	12	sp|Q9KU18|CLPB_VIBCH	clpB	95.77	chaperone protein heat shock
11	11	tr|Q9KR74|Q9KR74_VIBCH	VC_1769	90.70	dna methylase HsdM
11	11	tr|Q9KTB2|Q9KTB2_VIBCH	VC_0991	62.32	Asparagine synthetase B, glutamine-hydrolyzing
10	31	sp|Q9KNY5|RL4_VIBCH	rplD	21.85	50S ribosomal protein L4
10	23	sp|Q9KPC5|ENO_VIBCH	eno	45.78	enolase phosphoenolpyruvate pathway
10	15	tr|Q9KQU3|Q9KQU3_VIBCH	VC_1905	39.81	alanine dehydrogenase pyruvate pathway
10	14	sp|Q9KUY9|RL9_VIBCH	rplI	15.60	50S ribosomal protein L9
10	12	sp|P0C6Q3|PGK_VIBCH	pgk	40.95	phosphoglycerate kinase pyruvate pathway
10	11	tr|Q9KSW5|Q9KSW5_VIBCH	VC_1141	80.48	isocitrate dehydrogenase
10	11	sp|Q9KPB0|LEPA_VIBCH	lepA	65.99	elongation factor 4
10	10	tr|Q9KSW2|Q9KSW2_VIBCH	VC_1144	83.97	chaperone clpA ATP-dependent Clp protease, ATP-binding subunit ClpA
10	10	tr|Q9KPH1|Q9KPH1_VIBCH	ftsZ	41.50	
10	10	sp|Q9KNY1|RNR_VIBCH	rnr	93.28	ribonuclease r
9	101	tr|Q9KP61|Q9KP61_VIBCH	VC_2515	9.66	ibag
9	16	sp|Q9KU60|SYK_VIBCH	lysS	58.04	lysine t rna ligase
9	15	sp|Q9KLR4|NAPA_VIBCH	napA	93.05	nitrate reductase binds 4FE-4S cluster
9	14	sp|Q9KTM7|SYP_VIBCH	proS	63.21	proline t rna ligase
9	13	sp|Q9KUP3|METK_VIBCH	metK	42.04	S-adenosylmethionine synthase
9	13	sp|Q9KNJ2|GLN1B_VIBCH	glnAv	51.72	glutamine synthetase
9	12	tr|Q9KNL6|Q9KNL6_VIBCH	VC_2716	84.77	
9	10	sp|Q9KU97|GSA_VIBCH	hemL	46.15	Glutamate-1-semialdehyde 2,1-aminomutase
9	10	sp|Q9KQS7|CLPX_VIBCH	clpX	46.46	chaperone clpx
9	9	tr|Q9KQB4|Q9KQB4_VIBCH	VC_2086	44.06	2-oxoglutarate dehydrogenase
9	9	sp|Q9KUP2|TKT1_VIBCH	tkt1	71.57	
8	38	sp|P0C6Q6|OMPU_VIBCH	ompU	36.62	
8	15	sp|O34242|DNAJ_VIBCH	dnaJ	40.80	chaperone protein
8	14	tr|Q9KQB1|Q9KQB1_VIBCH	VC_2089	64.47	Succinate dehydrogenase flavoprotein subunit
8	12	sp|Q9KMN7|SYT_VIBCH	thrS	73.40	threonine t rna ligase
8	12	sp|P45383|RECA_VIBCH	recA	38.23	
8	10	sp|Q9KNH3|ATPA_VIBCH	atpA	55.62	atp synthase subunit alpha

8	10	sp|Q9KTE6|SYL_VIBCH	leuS	96.56	leucine t rna ligase
8	9	sp|Q9KU47|SYI_VIBCH	ileS	105.33	isoleucine t rna ligase
8	8	sp|Q9KRA3|FABV1_VIBCH	fabV	44.03	Enoyl-[acyl-carrier-protein] reductase [NADH] 1
8	8	sp|Q9X4Q8|NQRF_VIBCH	nqrF	45.04	Na(+)-translocating NADH-quinone reductase subunit F
8	8	sp|Q9KTY2|ISCS_VIBCH	iscS	44.78	
8	8	sp|Q9KVW8|SYGB_VIBCH	glyS	75.88	glycine t rna ligase
7	19	sp|Q06952|GM4D_VIBCH	gmd	42.03	GDP-mannose 4,6-dehydratase
7	16	tr|Q9KTZ9|Q9KTZ9_VIBCH	VC_0731	22.85	Antioxidant, AhpC/Tsa family
7	15	tr|Q9KPF5|Q9KPF5_VIBCH	VC_2413	66.03	Acetyltransferase component of pyruvate dehydrogenase complex
7	12	tr|Q9KPD9|Q9KPD9_VIBCH	parE	69.42	DNA topoisomerase 4 subunit B
7	12	sp|Q9KT88|ETCC_VIBCH	VC_1015	83.45	rfnc Ion-translocating oxidoreductase complex subunit C
7	12	tr|Q9KT14|Q9KT14_VIBCH	VC_1091	61.10	Oligopeptide ABC transporter, periplasmic oligopeptide-binding protein
7	11	sp|Q9KSN6|SYFB_VIBCH	pheT	86.08	phenylalanine t rna ligase
7	10	tr|Q9KNF1|Q9KNF1_VIBCH	VC_A0013	92.51	Alpha-1,4 glucan phosphorylase
7	10	sp|P0C6C6|FLAD_VIBCH	flaD	39.88	Flagellin D
7	10	tr|Q9KUN0|Q9KUN0_VIBCH	VC_0485	50.41	pyruvate kinase
7	10	sp|Q9KP08|RPOA_VIBCH	rpoA	36.39	DNA-directed RNA polymerase subunit alpha
7	9	sp|Q9KV34|RL11_VIBCH	rplK	14.77	50S ribosomal protein L11
7	9	sp|Q9KNZ9|RL6_VIBCH	rplF	18.80	50S ribosomal protein L6
7	9	tr|Q9KSF2|Q9KSF2_VIBCH	VC_1304	54.74	fumarate hydratae
7	8	sp|Q56648|SYA_VIBCH	alaS	93.77	alanine t rna ligase
7	8	sp|Q9KTW2|GUAA_VIBCH	guaA	57.73	gmp synthase
7	8	sp|Q9KPY7|UPP_VIBCH	upp	22.68	Uracil phosphoribosyltransferase
7	8	sp|Q9KQB5|SUCC_VIBCH	sucC	41.37	succinate coA ligase
7	8	tr|Q9KNQ0|Q9KNQ0_VIBCH	VC_2681	46.08	malate oxydoreductase
7	8	sp|Q9KV22|GPMI_VIBCH	gpmI	55.33	2,3-bisphosphoglycerate-independent phosphoglycerate mutase
7	8	tr|Q9KTJ1|Q9KTJ1_VIBCH	VC_0911	64.80	Trehalose-6-phosphate hydrolase
7	7	tr|Q9KQA5|Q9KQA5_VIBCH	VC_2095	59.31	phosphoglucomutase
7	7	sp|Q9KNZ6|RL5_VIBCH	rplE	20.15	50S ribosomal protein L7
7	7	tr|Q9KPE0|Q9KPE0_VIBCH	parC	84.95	DNA topoisomerase 4 subunit A
7	7	tr|Q9KQ20|Q9KQ20_VIBCH	ychF	41.71	ribosome binding atpase
7	7	tr|Q9KVN3|Q9KVN3_VIBCH	polA	103.81	DNA polymerase 1
7	7	sp|Q9KNT4|CAPP_VIBCH	ppc	98.25	Phosphoenolpyruvate carboxylase
7	7	sp|Q9KRC8|MUKB_VIBCH	mukB	169.86	chromosome partition mukB
6	11	sp|Q9KT07|ACKA1_VIBCH	ackA1	42.82	acetate kinase
6	10	tr|Q9KQD7|Q9KQD7_VIBCH	VC_2063	84.04	chemotaxis protein chea
6	9	sp|Q9KUM8|GLMS_VIBCH	glmS	67.06	Glutamine--fructose-6-phosphate aminotransferase
6	9	tr|Q9KUK1|Q9KUK1_VIBCH	rpoD	71.12	RNA polymerase sigma factor RpoD
6	8	tr|Q9KUN7|Q9KUN7_VIBCH	VC_0478	38.89	Fructose-bisphosphate aldolase, class II
6	7	sp|Q9KPV5|RRF_VIBCH	frr	20.63	Ribosome-recycling factor
6	7	sp|Q9KU89|GREA_VIBCH	greA	17.44	Transcription elongation factor GreA
6	6	sp|Q9KQ30|5NTD_VIBCH	nutA	60.86	5'-nucleotidase
6	6	sp|Q9KUW9|METH_VIBCH	metH	135.71	methionine synthase
6	6	tr|Q9KU86|Q9KU86_VIBCH	ftsH	71.66	ATP-dependent zinc metalloprotease FtsH
6	6	tr|Q9KRQ7|Q9KRQ7_VIBCH	VC_1579	63.09	Enterobactin synthetase component F-related protein
6	6	tr|Q9KP17|Q9KP17_VIBCH	VC_2562	74.98	2`,3`-cyclic-nucleotide 2`-phosphodiesterase
6	6	sp|Q9KP03|RL15_VIBCH	rplO	14.94	50S ribosomal protein L15
5	13	sp|Q9KQS5|TIG_VIBCH	tig	47.92	Trigger factor
5	13	sp|Q9KTE0|MIAB_VIBCH	miaB	53.58	tRNA-2-methylthio-N(6)-dimethylallyladenosine synthase
5	8	tr|Q9KU13|Q9KU13_VIBCH	VC_0717	52.44	protease
5	7	tr|H9L4Q1|H9L4Q1_VIBCH	VC_0249	52.54	rfbL
5	7	tr|Q9KLE2|Q9KLE2_VIBCH	deaD	73.74	ATP-dependent RNA helicase DeaD
5	7	sp|P0C6C3|FLAA_VIBCH	flaA	40.36	flagelin a
5	7	tr|Q9KNF0|Q9KNF0_VIBCH	VC_A0014	81.33	4-alpha-glucanotransferase
5	6	tr|H9L4Q4|H9L4Q4_VIBCH	VC_0252	20.00	Acetyltransferase RfbO, CysE/LacA/LpxA/NodL family
5	6	sp|Q9KP01|RS5_VIBCH	rpsE	17.57	30S ribosomal protein S5
5	6	sp|Q9KU64|RF3_VIBCH	prfC	59.59	Peptide chain release factor 3
5	5	sp|Q9KUY4|G6PI_VIBCH	pgi	60.65	Glucose-6-phosphate isomerase
5	5	tr|Q9KSG3|Q9KSG3_VIBCH	VC_1293	44.90	Aminotransferase
5	5	sp|Q9KUF1|RL13_VIBCH	rplM	15.98	50S ribosomal protein L13
5	5	tr|Q9KPJ1|Q9KPJ1_VIBCH	VC_2376	163.40	Glutamate synthase, large subunit
5	5	sp|Q9KLE1|RNB_VIBCH	rnb	76.10	exoribonuclease
5	5	tr|Q9KQG8|Q9KQG8_VIBCH	rne	117.45	ribonuclease
5	5	tr|Q9KKW5|Q9KKW5_VIBCH	VC_A0985	105.38	Oxidoreductase/iron-sulfur cluster-binding protein
5	5	sp|Q9KP62|MURA_VIBCH	murA	44.66	
5	5	tr|Q9KVF8|Q9KVF8_VIBCH	VC_0188	76.99	oligopeptidase a
5	5	sp|Q9KP07|RS4_VIBCH	rpsD	23.34	30S ribosomal protein S4
5	5	tr|Q9KP92|Q9KP92_VIBCH	VC_2481	44.21	D-3-phosphoglycerate dehydrogenase
4	9	sp|Q9KPF6|DLDH_VIBCH	lpd	50.96	Dihydrolipoyl dehydrogenase
4	7	sp|Q9KV33|RL1_VIBCH	rplA	24.60	50S ribosomal protein L1

4	7	sp|Q9KNH5|ATPB_VIBCH	atpD	50.49	ATP synthase subunit beta
4	6	sp|Q9KNX8|PURA_VIBCH	purA	46.79	Adenylosuccinate synthetase
4	5	sp|Q9KMT5|ACKA2_VIBCH	ackA2	42.80	Acetate kinase 2
4	5	tr|Q9KUG1|Q9KUG1_VIBCH	ffh	50.34	Signal recognition particle protein
4	5	sp|Q9KSA5|RLMI_VIBCH	rlmI	44.57	Ribosomal RNA large subunit methyltransferase I
4	5	sp|Q9KP34|IPYR_VIBCH	ppa	19.59	Inorganic pyrophosphatase
4	5	sp|Q9KS33|POTA_VIBCH	potA	42.67	Spermidine/putrescine import ATP-binding protein PotA
4	5	tr|Q9KQV8|Q9KQV8_VIBCH	VC_1890	47.50	NADH dehydrogenase
4	4	sp|Q9KPH8|CARA_VIBCH	carA	40.90	Carbamoyl-phosphate synthase small chain
4	4	tr|Q9KKW3|Q9KKW3_VIBCH	VC_A0987	88.43	Phosphoenolpyruvate synthase
4	4	sp|Q9KRB2|TOP1_VIBCH	topA	97.85	DNA topoisomerase 1
4	4	tr|Q9KT62|Q9KT62_VIBCH	VC_1043	46.77	Long-chain fatty acid transport protein
4	4	tr|Q9KQA8|Q9KQA8_VIBCH	VC_2092	48.56	Citrate synthase
4	4	sp|Q9KPA4|NADB_VIBCH	nadB	59.83	L-aspartate oxidase
4	4	tr|Q9KNW6|Q9KNW6_VIBCH	VC_2614	23.62	Cyclic AMP receptor protein
4	4	sp|Q9KRL3|CANSD_VIBCH	VC_1624	45.92	Carboxynorspermidine synthase
4	4	tr|Q9KVA5|Q9KVA5_VIBCH	VC_0244	41.07	Perosamine synthase
4	4	sp|Q9KVD5|PYRE_VIBCH	pyrE	23.39	Orotate phosphoribosyltransferase
4	4	sp|Q9KTX0|SYH_VIBCH	hisS	46.90	histidine t rna ligase
4	4	tr|Q9KPI2|Q9KPI2_VIBCH	VC_2386	62.64	Phosphomannomutase, putative
4	4	tr|Q9KKR1|Q9KKR1_VIBCH	VC_A1041	62.02	
4	4	sp|Q9KTX1|ISPG_VIBCH	ispG	40.62	
3	7	sp|Q9KV32|RL10_VIBCH	rplJ	17.32	50S ribosomal protein L10
3	7	sp|Q56652|LAMB_VIBCH	lamB	44.51	Maltoporin
3	6	sp|Q9KSR8|MAO1_VIBCH	maeA	62.11	NAD-dependent malic enzyme
3	5	tr|Q9KV50|Q9KV50_VIBCH	rho	46.66	Transcription termination factor Rho
3	4	sp|Q9KV09|HFLK_VIBCH	hflK	43.79	
3	4	sp|Q9KPV3|EFTS_VIBCH	tsf	29.83	Elongation factor Ts
3	4	sp|Q9KNY4|RL3_VIBCH	rplC	22.34	50S ribosomal protein L3
3	4	tr|Q9KL61|Q9KL61_VIBCH	kbl	43.24	2-amino-3-ketobutyrate coenzyme A ligase
3	4	sp|Q9KKW6|LLDD_VIBCH	lldD	41.26	L-lactate dehydrogenase
3	4	sp|Q9KQG2|DHAS1_VIBCH	asd1	40.47	Aspartate-semialdehyde dehydrogenase 1
3	4	sp|Q9KUZ8|RS7_VIBCH	rpsG	17.66	30S ribosomal protein S7
3	4	sp|Q9KR11|TOLB_VIBCH	tolB	49.61	Tol-Pal system protein TolB
3	4	tr|Q9KTT2|Q9KTT2_VIBCH	VC_0806	58.86	
3	4	tr|Q9KR21|Q9KR21_VIBCH	VC_1826	65.58	PTS system, fructose-specific IIABC component
3	3	sp|Q9KSG8|OPGG_VIBCH	opgG	60.84	Glucans biosynthesis protein G
3	3	tr|Q9KL25|Q9KL25_VIBCH	VC_A0924	73.68	
3	3	sp|Q9KQD8|CHEB1_VIBCH	cheB1	40.35	Protein-glutamate methylesterase/protein-glutamine glutaminase 1
3	3	tr|Q9KP90|Q9KP90_VIBCH	VC_2483	62.82	Acetolactate synthase
3	3	tr|Q9KVN6|Q9KVN6_VIBCH	VC_0105	38.51	Delta-aminolevulinic acid dehydratase
3	3	sp|Q9KUG4|LUXS_VIBCH	luxS	19.07	
3	3	sp|Q9KP70|RAPA_VIBCH	rapA	109.37	RNA polymerase-associated protein RapA
3	3	sp|Q9KQ25|RF1_VIBCH	prfA	40.20	Peptide chain release factor 1
3	3	sp|P23247|DHAS2_VIBCH	asd2	37.35	Aspartate-semialdehyde dehydrogenase 2
3	3	sp|Q9KNH1|ATPF_VIBCH	atpF	17.65	ATP synthase subunit b
3	3	sp|Q9K2Y1|TOLC_VIBCH	tolC	47.72	
3	3	sp|P66367|RS11_VIBCH	rpsK	13.88	30S ribosomal protein S11
3	3	tr|Q9KUE5|Q9KUE5_VIBCH	VC_0576	24.26	Stringent starvation protein A
3	3	tr|Q9KTF5|Q9KTF5_VIBCH	VC_0947	42.86	D-alanyl-D-alanine carboxypeptidase
3	3	sp|Q9KTZ1|QUEA_VIBCH	queA	39.43	S-adenosylmethionine:tRNA ribosyltransferase-isomerase
3	3	tr|Q9KR20|Q9KR20_VIBCH	VC_1827	43.25	Mannose-6-phosphate isomerase
3	3	tr|Q9KS85|Q9KS85_VIBCH	VC_1374	68.17	DnaK-related protein
3	3	sp|Q9KPC4|PYRG_VIBCH	pyrG	59.85	CTP synthase
3	3	tr|Q9KSP3|Q9KSP3_VIBCH	VC_1213	23.74	Transcriptional regulator, LuxR family
3	3	sp|Q9KQT2|KCY_VIBCH	cmk	24.45	Cytidylate kinase
3	3	tr|Q9KVH5|Q9KVH5_VIBCH	VC_0171	60.58	Peptide ABC transporter, periplasmic peptide-binding protein
3	3	sp|Q9KLD1|SPEA_VIBCH	speA	72.20	Biosynthetic arginine decarboxylase
3	3	sp|Q9KPB3|ERA_VIBCH	era	37.04	GTPase Era
3	3	tr|Q9KQ66|Q9KQ66_VIBCH	VC_2137	54.96	Sigma-54 dependent transcriptional activator
3	3	tr|Q9KRZ7|Q9KRZ7_VIBCH	VC_1486	72.05	ABC transporter, ATP-binding protein
3	3	tr|Q9KNP3|Q9KNP3_VIBCH	VC_2688	36.04	Fructose-1,6-bisphosphatase
3	3	sp|Q9KTY9|TGT_VIBCH	tgt	43.23	Queuine tRNA-ribosyltransferase
3	3	tr|Q9KPT3|Q9KPT3_VIBCH	VC_2279	58.67	Aminoacyl-histidine dipeptidase
3	3	tr|Q9KT66|Q9KT66_VIBCH	VC_1039	75.24	AsmA protein
2	10	sp|Q9KNY7|RL2_VIBCH	rplB	29.81	50S ribosomal protein L2
2	5	sp|Q9KNL2|NFUA_VIBCH	nfuA	21.09	Fe/S biogenesis protein NfuA
2	5	tr|Q9KR12|Q9KR12_VIBCH	VC_1835	18.48	Pal Peptidoglycan-associated protein
2	4	sp|Q9KTX8|HSCA_VIBCH	hscA	65.80	Chaperone protein HscA homolog
2	3	sp|Q9KNY9|RL22_VIBCH	rplV	12.19	50S ribosomal protein L22

2	3	tr|Q9KTW3|Q9KTW3_VIBCH	guaB	51.91	Inosine-5'-monophosphate dehydrogenase
2	3	tr|Q9KMW6|Q9KMW6_VIBCH	VC_A0200	42.02	
2	2	tr|Q9KM70|Q9KM70_VIBCH	VC_A0518	42.59	PTS system, fructose-specific IIA/FPR component
2	2	sp|Q9KQV6|Y1892_VIBCH	VC_1892	20.75	UPF0227 protein VC_1892
2	2	sp|Q9KTG1|GLYA1_VIBCH	glyA1	45.38	Serine hydroxymethyltransferase 1
2	2	tr|Q9KSK1|Q9KSK1_VIBCH	VC_1255	45.05	Ribonucleoside-diphosphate reductase, beta subunit
2	2	tr|Q9KRZ8|Q9KRZ8_VIBCH	VC_1485	60.53	
2	2	tr|Q9KPR8|Q9KPR8_VIBCH	VC_2298	22.45	Lipoprotein, putative
2	2	sp|Q9KU92|SYY2_VIBCH	tyrS2	44.05	tyrosine t rna ligase
2	2	sp|Q9KVL7|DCDA_VIBCH	lysA	46.25	Diaminopimelate decarboxylase
2	2	tr|Q9KPK5|Q9KPK5_VIBCH	VC_2362	46.20	threonine synthase
2	2	sp|P0C6C5|FLAC_VIBCH	flaC	39.88	flagelin c
2	2	tr|Q9KLZ7|Q9KLZ7_VIBCH	VC_A0592	21.63	MutT/nudix family protein
2	2	tr|Q9KSS8|Q9KSS8_VIBCH	VC_1178	23.14	
2	2	tr|Q9KUF3|Q9KUF3_VIBCH	zapE	42.47	cell division
2	2	tr|Q9KT65|Q9KT65_VIBCH	VC_1040	22.43	Corrinoid adenosyltransferase
2	2	tr|Q9KPW0|Q9KPW0_VIBCH	bamA	89.87	Outer membrane protein assembly factor BamA
2	2	sp|Q9KPY2|GMHA_VIBCH	gmhA	20.63	Phosphoheptose isomerase
2	2	sp|Q9KSX8|MNMA_VIBCH	mnmA	41.82	tRNA-specific 2-thiouridylase MnmA
2	2	tr|Q9KSV7|Q9KSV7_VIBCH	VC_1149	61.65	Glutamate decarboxylase, putative
2	2	sp|Q9KPT8|PROB_VIBCH	proB	40.13	Glutamate 5-kinase
2	2	sp|Q9KTL3|DXS_VIBCH	dxs	68.30	1-deoxy-D-xylulose-5-phosphate synthase
2	2	sp|Q9KP09|RL17_VIBCH	rplQ	14.40	50S ribosomal protein L17
2	2	tr|Q9KUH1|Q9KUH1_VIBCH	VC_0550	64.75	Oxaloacetate decarboxylase, alpha subunit
2	2	sp|Q9KRL4|NSPC_VIBCH	nspC	43.07	Carboxynorspermidine/carboxyspermidine decarboxylase
2	2	tr|Q9KTJ2|Q9KTJ2_VIBCH	VC_0910	50.82	PTS system, trehalose-specific IIBC component
2	2	sp|Q9KSR6|PUR7_VIBCH	purC	41.58	Phosphoribosylaminoimidazole-succinocarboxamide synthase
2	2	sp|Q9KVD1|COABC_VIBCH	coaBC	42.62	Coenzyme A biosynthesis bifunctional protein CoaBC
2	2	sp|Q9KV80|PUR9_VIBCH	purH	57.29	Bifunctional purine biosynthesis protein PurH
2	2	tr|Q9KKM8|Q9KKM8_VIBCH	VC_A1075	43.03	
2	2	tr|Q9KL28|Q9KL28_VIBCH	VC_A0921	42.36	
2	2	tr|Q9KQL3|Q9KQL3_VIBCH	VC_1985	62.81	Long-chain-fatty-acid--CoA ligase
2	2	tr|Q9KNP7|Q9KNP7_VIBCH	VC_2684	88.44	Bifunctional aspartokinase/homoserine dehydrogenase
2	2	sp|Q9KNS8|SECB_VIBCH	secB	17.02	Protein-export protein SecB
2	2	sp|P32557|DSBA_VIBCH	dsbA	22.55	Thiol:disulfide interchange protein DsbA
2	2	tr|Q9KVA7|Q9KVA7_VIBCH	VC_0239	67.07	
2	2	tr|Q9KTE1|Q9KTE1_VIBCH	VC_0961	41.56	PhoH family protein
2	2	sp|Q9KNW0|RIMO_VIBCH	rimO	52.45	Ribosomal protein S12 methylthiotransferase RimO
2	2	sp|Q9KLC6|CH602_VIBCH	groL2	56.16	chaperonine
2	2	sp|Q9KUY6|ALR1_VIBCH	alr1	39.32	Alanine racemase 1
2	2	sp|Q9KSE5|ALR2_VIBCH	alr2	42.47	Alanine racemase 2
2	2	tr|Q9KSJ2|Q9KSJ2_VIBCH	VC_1264	47.33	Iron-regulated protein A, putative
2	2	sp|Q9KTX5|PEPB_VIBCH	pepB	46.73	peptidase B
2	2	tr|Q9KNX2|Q9KNX2_VIBCH	VC_2608	75.53	ABC transporter, ATP-binding protein
2	2	tr|Q9KLJ5|Q9KLJ5_VIBCH	VC_A0749	46.12	Anaerobic glycerol-3-phosphate dehydrogenase, subunit C
2	2	tr|Q9KSF6|Q9KSF6_VIBCH	VC_1300	49.22	L-serine dehydratase 1
2	2	tr|Q9KV62|Q9KV62_VIBCH	VC_0295	49.43	Biotin carboxylase
2	2	tr|Q9KL41|Q9KL41_VIBCH	VC_A0907	20.29	Maltose/maltodextrin import ATP-binding protein MalK
2	2	sp|Q9KL04|MALK_VIBCH	malK	41.38	
2	2	tr|Q9KLP1|Q9KLP1_VIBCH	VC_A0702	41.96	Iron-containing alcohol dehydrogenase
1	5	tr|Q9KQJ8|Q9KQJ8_VIBCH	VC_2000	35.26	Glyceraldehyde-3-phosphate dehydrogenase
1	4	tr|Q9KKP6|Q9KKP6_VIBCH	VC_A1056	84.32	Methyl-accepting chemotaxis protein
1	4	tr|Q9KMC3|Q9KMC3_VIBCH	VC_A0441	35.61	
1	3	tr|Q9KSB9|Q9KSB9_VIBCH	VC_1339	42.04	
1	3	sp|Q9KSF9|SYN_VIBCH	asnS	52.42	asparagine t rna ligase
1	3	tr|Q9KSH5|Q9KSH5_VIBCH	VC_1281	11.14	PTS system, cellobiose-specific IIB component
1	2	sp|Q9KNX6|SLYD_VIBCH	slyD	21.23	FKBP-type peptidyl-prolyl cis-trans isomerase SlyD
1	2	tr|Q9KRY7|Q9KRY7_VIBCH	VC_1496	74.73	Tail-specific protease
1	2	sp|Q9KP05|RS13_VIBCH	rpsM	13.26	30S ribosomal protein S13
1	2	tr|Q9KRV7|Q9KRV7_VIBCH	VC_1527	65.88	Molybdopterin biosynthesis MoeA protein
1	2	sp|Q06951|RFBB_VIBCH	rfbB	51.81	Phosphomannomutase
1	2	tr|Q9KKM6|Q9KKM6_VIBCH	VC_A1077	67.08	
1	2	sp|Q9KV31|RL7_VIBCH	rplL	12.24	50S ribosomal protein L7/L12
1	2	sp|Q9KNS1|EFP_VIBCH	efp	20.56	Elongation factor P
1	2	sp|Q9KNU5|PBPA_VIBCH	mrcA	91.93	Penicillin-binding protein 1A
1	2	sp|Q9KUC7|GLUQ_VIBCH	gluQ	33.97	Glutamyl-Q tRNA(Asp) synthetase
1	2	sp|Q9KU04|PSTB1_VIBCH	pstB1	30.57	Phosphate import ATP-binding protein PstB 1
1	2	tr|Q9KNP0|Q9KNP0_VIBCH	VC_2691	18.56	Periplasmic protein cpxP, putative
1	2	tr|Q9KRM4|Q9KRM4_VIBCH	VC_1612	26.63	Fimbrial biogenesis and twitching motility protein, putative
1	2	sp|Q9KPH9|CARB_VIBCH	carB	117.84	Carbamoyl-phosphate synthase large chain

1	1	tr|Q9KLA3|Q9KLA3_VIBCH	VC_A0843	55.64	Glyceraldehyde 3-phosphate dehydrogenase
1	1	sp|Q9KUS8|OBG_VIBCH	cgtA	43.02	GTPase Obg/CgtA
1	1	tr|Q9KPY9|Q9KPY9_VIBCH	VC_2223	38.13	Pseudouridine synthase
1	1	sp|Q9KU96|ERPA_VIBCH	erpA	12.01	Iron-sulfur cluster insertion protein ErpA
1	1	tr|Q9KQB2|Q9KQB2_VIBCH	VC_2088	26.37	Succinate dehydrogenase iron-sulfur subunit
1	1	sp|Q9KNB2|DEOD2_VIBCH	deoD2	25.62	Purine nucleoside phosphorylase DeoD-type 2
1	1	sp|Q9KUF7|RL19_VIBCH	rplS	13.24	50S ribosomal protein L19
1	1	sp|Q9KPL8|TYPH_VIBCH	deoA	47.81	Thymidine phosphorylase
1	1	tr|Q9KVI1|Q9KVI1_VIBCH	VC_0165	40.42	
1	1	sp|Q9KV35|NUSG_VIBCH	nusG	20.64	Transcription termination/antitermination protein NusG
1	1	sp|Q9KPW2|LPXD_VIBCH	lpxD	36.99	UDP-3-O-acylglucosamine N-acyltransferase
1	1	sp|Q9KVC8|RL28_VIBCH	rpmB	8.98	50S ribosomal protein L28
1	1	sp|Q9KPT9|PROA_VIBCH	proA	44.48	Gamma-glutamyl phosphate reductase
1	1	sp|Q9KVU3|DEF1_VIBCH	def1	19.14	Peptide deformylase 1
1	1	sp|Q9KUW2|SSB_VIBCH	ssb	19.66	Single-stranded DNA-binding protein
1	1	sp|Q9KU84|GLMM_VIBCH	glmM	47.82	Phosphoglucosamine mutase
1	1	sp|Q9KPV8|DXR_VIBCH	dxr	43.66	1-deoxy-D-xylulose 5-phosphate reductoisomerase
1	1	sp|Q9KSJ7|Y1259_VIBCH	VC_1259	37.14	
1	1	sp|Q9KQH9|FABF_VIBCH	fabF	43.18	3-oxoacyl-[acyl-carrier-protein] synthase 2
1	1	sp|Q9KQ85|AROC_VIBCH	aroC	39.08	Chorismate synthase
1	1	tr|Q9KPD2|Q9KPD2_VIBCH	VC_2440	58.66	
1	1	tr|Q9KQM1|Q9KQM1_VIBCH	VC_1977	45.19	Aspartate aminotransferase, putative
1	1	tr|Q9KRX1|Q9KRX1_VIBCH	VC_1512	21.90	Formate dehydrogenase, iron-sulfur subunit
1	1	tr|H9L4Q5|H9L4Q5_VIBCH	VC_0409	18.43	MSHA pilin protein MshA
1	1	tr|Q9KSX6|Q9KSX6_VIBCH	VC_1130	15.15	DNA-binding protein
1	1	tr|Q9KSR1|Q9KSR1_VIBCH	VC_1195	23.52	Lipoprotein, putative
1	1	sp|P0C6C8|FUR_VIBCH	fur	16.90	
1	1	tr|Q9KLV4|Q9KLV4_VIBCH	VC_A0637	24.06	Oxygen-insensitive NAD(P)H nitroreductase
1	1	tr|Q9KRS3|Q9KRS3_VIBCH	VC_1563	36.80	
1	1	tr|Q9KP11|Q9KP11_VIBCH	VC_2568	22.23	Peptidyl-prolyl cis-trans isomerase
1	1	sp|Q9KL83|VOLA_VIBCH	volA	83.51	Lysophospholipase VolA
1	1	tr|Q9KQ36|Q9KQ36_VIBCH	VC_2168	42.11	
1	1	sp|P52022|DPO3A_VIBCH	dnaE	129.97	DNA polymerase III subunit alpha
1	1	sp|Q9KPU4|RISB_VIBCH	ribH	16.42	6,7-dimethyl-8-ribityllumazine synthase
1	1	tr|Q9KPJ3|Q9KPJ3_VIBCH	VC_2374	53.40	Glutamate synthase, small subunit
1	1	tr|Q9KKJ2|Q9KKJ2_VIBCH	VC_A1115	46.36	ParA family protein
1	1	tr|Q9KS77|Q9KS77_VIBCH	VC_1382	149.46	ATP-dependent helicase HrpA
1	1	sp|Q9KSN7|SYFA_VIBCH	pheS	37.03	phenylalanine t rna ligase
1	1	tr|Q9KNP8|Q9KNP8_VIBCH	VC_2683	42.11	Cystathionine gamma-synthase
1	1	sp|Q9KNV1|AROK_VIBCH	aroK	19.61	Shikimate kinase
1	1	tr|Q9KS37|Q9KS37_VIBCH	VC_1424	38.97	Putrescine-binding periplasmic protein
1	1	sp|O30862|GRPE_VIBCH	grpE	22.65	Protein GrpE
1	1	tr|Q9KT02|Q9KT02_VIBCH	VC_1103	28.72	ABC transporter, ATP-binding protein
1	1	sp|Q9KTD1|DNLJ_VIBCH	ligA	73.25	dna ligase
1	1	sp|Q9KNR6|CH101_VIBCH	groS1	10.29	chaperonine
1	1	sp|Q9KTB7|KAD_VIBCH	adk	23.26	Adenylate kinase
1	1	tr|Q9KU34|Q9KU34_VIBCH	VC_0695	39.70	Phospho-2-dehydro-3-deoxyheptonate aldolase
1	1	tr|Q9KTB0|Q9KTB0_VIBCH	VC_0993	43.86	N-acetylglucosamine repressor
1	1	tr|Q9KLX4|Q9KLX4_VIBCH	VC_A0617	47.31	Molybdopterin biosynthesis MoeA protein
1	1	sp|Q9KQH7|FABG_VIBCH	fabG	25.55	3-oxoacyl-[acyl-carrier-protein] reductase FabG
1	1	tr|Q9KVS2|Q9KVS2_VIBCH	VC_0067	66.57	Aminopeptidase P
1	1	sp|Q9KPS1|NQRA_VIBCH	nqrA	48.59	Na(+)-translocating NADH-quinone reductase subunit A
1	1	sp|Q9KPL9|DEOB_VIBCH	deoB	44.13	Phosphopentomutase
1	1	tr|Q9KTY1|Q9KTY1_VIBCH	VC_0749	13.65	Iron-sulfur cluster assembly scaffold protein IscU
1	1	sp|Q9KTW7|DER_VIBCH	der	55.60	gtpase
1	1	sp|Q9KPN7|DAPD_VIBCH	dapD	35.63	2,3,4,5-tetrahydropyridine-2,6-dicarboxylate N-succinyltransferase
1	1	tr|Q9KUV9|Q9KUV9_VIBCH	VC_0402	60.10	MSHA biogenesis protein MshL
1	1	sp|Q9KST9|KITH_VIBCH	tdk	21.64	Thymidine kinase
1	1	tr|Q9KNL1|Q9KNL1_VIBCH	VC_2721	23.15	MutT/nudix family protein
1	1	sp|Q9KTK8|THII_VIBCH	thiI	54.29	tRNA sulfurtransferase
1	1	tr|Q9KVB5|Q9KVB5_VIBCH	VC_0231	59.81	
1	1	tr|Q9KUU6|Q9KUU6_VIBCH	VC_0419	55.26	Cytoplasmic axial filament protein
1	1	sp|Q9KLJ3|FABH2_VIBCH	fabH2	39.00	3-oxoacyl-[acyl-carrier-protein] synthase 3 protein 2
1	1	sp|Q9KNZ0|RS3_VIBCH	rpsC	25.60	30S ribosomal protein S3
1	1	tr|Q9KUH4|Q9KUH4_VIBCH	VC_0547	42.44	Aspartokinase
1	1	sp|Q9KQH8|ACP_VIBCH	acpP	8.66	Acyl carrier protein
1	1	sp|Q9KNF8|Y2806_VIBCH	VC_A0006	26.34	Probable transcriptional regulatory protein VC_A0006
1	1	tr|Q9KQ69|Q9KQ69_VIBCH	VC_2133	63.81	Flagellar M-ring protein
1	1	tr|Q9KSE4|Q9KSE4_VIBCH	VC_1313	59.13	Methyl-accepting chemotaxis protein
1	1	tr|Q9KTD8|Q9KTD8_VIBCH	VC_0964	18.01	PTS system, glucose-specific IIA component
1	1	tr|Q9KQD2|Q9KQD2_VIBCH	VC_2068	55.40	Flagellar biosynthetic protein FlhF, putative
1	1	sp|Q9KT67|URK_VIBCH	udk	24.21	uridine kinase
1	1	sp|Q9KMP4|GLYA2_VIBCH	glyA2	46.88	Serine hydroxymethyltransferase 2
1	1	sp|Q9KUT0|RL21_VIBCH	rplU	11.50	50S ribosomal protein L21
1	1	sp|Q9KUU7|Y418_VIBCH	VC_0418	20.48	Maf-like protein VC_0418
1	1	tr|Q9KLH9|Q9KLH9_VIBCH	VC_A0765	37.30	L-allo-threonine aldolase

Lipid category					
Unique peptides	Total peptides	reference	Gene Symbol	MW(kDa)	Predicted function
18	33	tr|Q9KV99|Q9KV99_VIBCH	VC_0251	91.8	RfbN
8	8	sp|Q9KRA3|FABV1_VIBCH	fabV	44.0	Enoyl-[acyl-carrier-protein] reductase [NADH] 1
5	7	tr|H9L4Q1|H9L4Q1_VIBCH	VC_0249	52.5	RfbL
5	6	tr|H9L4Q4|H9L4Q4_VIBCH	VC_0252	20.0	Acetyltransferase RfbO, CysE/LacA/LpxA/NodL family
4	4	tr|Q9KT62|Q9KT62_VIBCH	VC_1043	46.8	Long-chain fatty acid transport protein
2	2	tr|Q9KPR8|Q9KPR8_VIBCH	VC_2298	22.5	Lipoprotein, putative
2	2	tr|Q9KQL3|Q9KQL3_VIBCH	VC_1985	62.8	Long-chain-fatty-acid--CoA ligase
1	2	sp|Q06951|RFBB_VIBCH	rfbB	51.8	Phosphomannomutase
1	1	tr|Q9KSR1|Q9KSR1_VIBCH	VC_1195	23.5	Lipoprotein, putative
1	1	sp|Q9KQH9|FABF_VIBCH	fabF	43.2	3-oxoacyl-[acyl-carrier-protein] synthase 2
1	1	sp|Q9KQH7|FABG_VIBCH	fabG	25.6	3-oxoacyl-[acyl-carrier-protein] reductase FabG
1	1	sp|Q9KLJ3|FABH2_VIBCH	fabH2	39.0	3-oxoacyl-[acyl-carrier-protein] synthase 3 protein 2
1	1	sp|Q9KL83|VOLA_VIBCH	volA	83.5	Lysophospholipase VolA

Cell wall and envelope category					
Unique peptides	Total peptides	reference	Gene Symbol	MW(kDa)	Predicted function
3	4	sp|Q9KR11|TOLB_VIBCH	tolB	49.6	TolB
3	3	sp|Q9K2Y1|TOLC_VIBCH	tolC	47.7	TolC
2	5	tr|Q9KR12|Q9KR12_VIBCH	VC_1835	18.5	Pal Peptidoglycan-associated protein

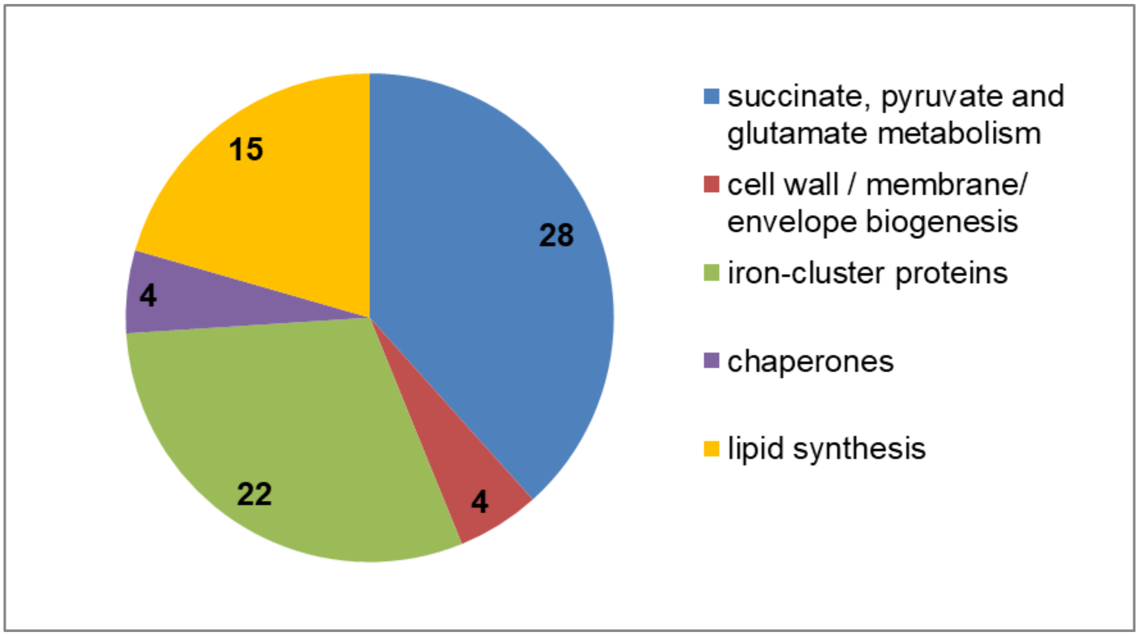

Supplement: TABLE S2 [file mBio.00790-19-st002.pdf]
