# Supplementary material for: A Vibrio cholerae BolA-Like Protein Is Required for Proper Cell Shape and Cell Envelope Integrity
Source: mBio. 2019 Jul 9;10(4):e00790-19. doi: 10.1128/mBio.00790-19 (PMC6747721; doi:10.1128/mBio.00790-19)
Supplement: TABLE S3 [file mBio.00790-19-st003.pdf]

Table S3. Strains, plasmids and oligos

Strain	Relevant genotype	Source
***V. cholerae* N16961**		
**AF01**	ΔibaG (vc2515)	this study
**AF02**	ΔbolA (vc2296)	this study
**AF03**	pBAD33 bolA	this study
**AF04**	pBAD33 ibaG	this study
**AF05**	ΔibaG (vc2515) pBAD33 ibaG	this study
**AF06**	N16961 lacz-	Dorr *et al* ., 2014
**AF07**	N16961 ibaG-TAP-TAG	this study
Escherichia coli		this study
SM10 λpir	Δ(ara-leu)7697 ΔlacX74 ΔphoA PvuII phoR araD139 ahpC galE galK rpsL (DE3) F'[lac+ lacIq pro] gor522::Tn10 trxB pLysSRARE (CamR, StrR, TetR)	
DH5 alpha λpir	F- endA1 glnV44 thi-1 recA1 relA1 gyrA96 deoR nupG Φ80d*lacZ* ΔM15 Δ(*lacZYA-argF*)U169, hsdR17(rK- mK+), λpir	
BTH101	F-, cya-99, araD139, galE15, galK16, rpsL1 (Str r), hsdR2, mcrA1, mcrB1	
**AF08**	SM10 λpir pCVD442 ΔbolA	deletion plasmid for bolA, this study
**AF09**	SM10 λpir pCVD442 ΔibaG	deletion plasmid for ibaG, this study
**Plasmids**		
pCVD442	carbR, ori R6K gamma, *sacB*	Donnenberg and Kaper 1991
pBAD33	CmR, arabinose inducible pBAD promoter	Guzman *et al* ., 1995
pAF001	pCVD442 ΔbolA	this study
pAF002	pCVD442 ΔibaG	this study
pAF003	pBAD33 ibaG	this study
pAF004	pBAD33 bolA	this study
pAF005	pKT25 ibaG	this study
pAF006	pKNT25 ibaG	this study
pAF007	pUT18 ibaG	this study
pAF008	pUT18c ibaG	this study
pAF009	pKT25 ispG	this study
pAF010	pKNT25 ispG	this study
pAF011	pUT18 ispG	this study
pAF012	pUT18c ispG	this study
pAF013	pKT25 grx4	this study
pAF014	pKNT25 grx4	this study
pAF015	pUT18 grx4	this study
pAF016	pUT18c grx4	this study

**Primers**			
**Name**	**Description**	**Sequence**	**Comment**
p01	XbaI-ibag-Nterm	TATTCTAGAGgtggatagcgcacaagtccaac	For bacterial two hybrid assay
p02	KpnI-ibag-stop-Cterm	TATGGTACCCGttaaagggacatcaacttcttattg	For bacterial two hybrid assay
p03	KpnI-ibag-Cterm	TATGGTACCCGaagggacatcaacttcttattgcgt	For bacterial two hybrid assay
p04	XbaI-grx4-Nterm	TATTCTAGAGatggaaactattgacaaaatcaaac	For bacterial two hybrid assay
p05	KpnI-grx4-stop-Cterm	TATGGTACCCGttactcttggcttgctgagcgagcagc	For bacterial two hybrid assay
p06	KpnI-grx4-Cterm	TATGGTACCCGctcttggcttgctgagcgagcagc	For bacterial two hybrid assay
p07	BamHI-ispg-Nterm	TATGGATCCCatgtctatgcaacatgagtctc	For bacterial two hybrid assay
p08	SacI-ispg-stop-Cterm	TATGAGCTCGGttaatcttgttctacgtgcttg	For bacterial two hybrid assay
p09	SacI-ispg-Cterm	TATGGTACCGGatcttgttctacgtgcttgata	For bacterial two hybrid assay
p10	KpnI-ispg-stop-Cterm	TATGGTACCCGttaatcttgttctacgtgcttg	For bacterial two hybrid assay
p11	qpcr-ibag-fw	gatagcgcacaagtccaaca	For RT qPCR
p12	qpcr-ibag-rev	tgtgaccgcgtgaatatcat	For RT qPCR
p13	pCVD442fw-bolA-up	aggtatatgtgatgggttaaaaaggatcgatcctCAAACAGATTCAAGGTTGATGTATGAATCTCCT	To construct ∆bolA in pcvd442
p14	linkerrev-bolA-up	TTATCATTACTCGAGTGCGGCCGCAtgaTAAATTGCTTTCCTTGTTGAACACTTGAGTGTCG	To construct ∆bolA in pcvd442
p15	linkerfw-bolA-down	TTAtcaTGCGGCCGCACTCGAGTAATGATAATAGGTTTGATACCTATAACGCTTGATTGG	To construct ∆bolA in pcvd442
p16	pCVD442rev-bolA-down	ccgggagagctcgatatcgcatgcggtacctctagCACTTCAATCACTACAGACTGAAGAACGC	To construct ∆bolA in pcvd442
p17	pCVD442fw-ibag-up	aggtatatgtgatgggttaaaaaggatcgatcctTGCGCCAAGAGAAATCGGGTGAGTGG	To construct ∆ibaG in pcvd442
p18	linkerrev-ibag-up	TTATCATTACTCGAGTGCGGCCGCAtgaTAACTATCCACAATTCACCTCTGCTGGTTTGGC	To construct ∆ibaG in pcvd442
p19	linkerfw-ibag-down	TTAtcaTGCGGCCGCACTCGAGTAATGATAACCCTTTAAGGTTTTTATGGAAAAGTTTCGAG	To construct ∆ibaG in pcvd442
p20	pCVD442rev-ibag-down	ccgggagagctcgatatcgcatgcggtacctctagCCACGCTCACTTTATCCATCACAATATGCGCGCC	To construct ∆ibaG in pcvd442
p21	pBAD33-ibaG-fw	gtttttttgggctagcgaattcgaggtggatagcgcacaagtccaaca	To overexpress ibaG in a pBAD33 cut by Sac1(Ecl136II)
p22	pBAD33-ibaG-rev	ctctagaggatccccgggtaccgagttaaagggacatcaacttcttattgcg	To overexpress ibaG in a pBAD33 cut by Sac1(Ecl136II)
p23	Rev-IbaG-Tap	CTTCTCTTTTCCATGGAAAGGGACATCAACTTCTTATTGCGTGCCCACTCTTGTGGGGTAAA	To construct ibaG fused to TAP TAG
p24	Fw-IbaG-Tap	TTTACCCCACAAGAGTGGGCACGCAATAAGAAGTTGATGTCCCTTTCCATGGAAAAGAGAAG	To construct ibaG fused to TAP TAG
p25	Tap-rev	tcaggttgacttccccgcggaattcgcgtc	To construct ibaG fused to TAP TAG
p26	Tap-IbaG-down	gacgcgaattccgcggggaagtcaacctgaGGTTTTTATGGAAAAGTTTCGAGTTATTGG	To construct ibaG fused to TAP TAG
